# Supplementary material for: Single cell transcriptional perturbome in pluripotent stem cell models
Source: Mol Syst Biol. 2025 Dec 10;22(2):179–227. doi: 10.1038/s44320-025-00172-8 (PMC12864791; doi:10.1038/s44320-025-00172-8)
Supplement: Supplementary file 1 — Appendix [file 44320_2025_172_MOESM1_ESM.pdf]

Appendix for Single Cell Transcriptional Perturbome in Pluripotent Stem Cell Models

- Appendix Text - Page 2
- Appendix Figure S1. Optimization of molecular biology and genome editing steps for iPS2-seq - Page 4
- Appendix Figure S2. Optimization of iPS2-sci-seq - Page 7
- Appendix Methods - Page 11
- Appendix Table S1. Disease relevance of genes selected for loss of function perturbations - Page 13
- Appendix Table S2. Sanger sequencing QC of pooled cloning step 1 optimization experiments - Page 14
- Appendix Table S3. Key figures of iPS2-seq NGS experiments - Page 14
- Appendix Table S4. Number of cells per perturbation - Page 14
- Appendix Table S5. Mean expression of perturbation targets - Page 15
- Appendix Table S6. Pathway and gene ontology enrichment analyses of gene modules - Page 16
- Appendix Table S7. Copy number variation analysis - Page 17
- Appendix Protocols Overview. - Page 18
- Appendix Protocol 1. Generation of iPS2-seq plasmids and hiPSCs - Page 19
- Appendix Protocol 2. iPS2-sci-seq library preparation - Page 36
- Appendix Protocol 3. iPS2-10X-seq library preparation - Page 42
- Appendix Protocol 4. iPS2-seq design and analysis with *catcheR* - Page 45

Appendix Text

The implementation of iPS2-seq described in the main text and in the supplemental protocols was the result of extensive trial-and-error iterations. We summarize here the main findings from these optimization experiments as a resource for those that may decide to tinker with the methodology or related approaches.

Optimization of molecular cloning and genome editing

In a pilot experimental run, we cloned PCR-amplified shRNA and UCI-BC sequences in our original *AAVS1* targeting vector (pAAV-Puro_siKD; Bertero et al. (2016)). After reconstituting the tet-ON system through re-insertion of CAG-OPTtetR, we targeted hiPSCs with the resulting plasmid pool and selected genome-edited clones with puro. Throughout this experiment we performed the same quality controls described in the main text for Figures EV1-B-EV1-D, and made the following key observations that informed subsequent troubleshooting:

- In the first pooled cloning step, amplification of shRNAs by PCR using Q5 High-Fidelity DNA Polymerase introduced mutations, barcode swaps, and representation biases. Despite appearing at first ~97% efficient by colony PCR (Appendix Figure S1A), careful observation of band size variations suggested frequent indels which were confirmed by Sanger sequencing (Appendix Figure S1B and Appendix Table S2): ~45% of tested plasmids carried mutations, including deletions of up to 13 base pairs (Appendix Figure S1C). ~16% of plasmids also showed a swap between the shRNA and a BC designed to be associated to a different perturbation (Appendix Figure S1C and Appendix Table S2). shRNA representation was highly variable and significantly different from the anticipated Gaussian distribution ($P < 1 \times 10^{-5}$; Appendix Figure S1B), with one shRNA against *SMAD2* representing approximately one third of tested bacterial clones and seven sequences dropping out. Of note, for this experiment each oligonucleotide had been manually pooled at equimolar amounts, ruling out inefficient ssDNA synthesis. Collectively, these observations suggested a combination of selective amplification as well as intra- and inter-molecular template switching by the otherwise high-fidelity DNA polymerase. We speculated that this arises from the difficulties posed during PCR by the strong secondary structure of shRNAs. We thus tested the following alternative approaches:

  1. Direct Gibson assembly of ssDNA oligonucleotides. This strategy was extremely inefficient (~6% by colony PCR; Appendix Figure S1A), suggesting that shRNA secondary structure posed a near-insurmountable barrier to the activity of high-fidelity DNA polymerase at the temperature required for the activity of the other enzymes (50°C).
  2. Annealing of complementary pairs of ssDNA oligonucleotides, followed by ligation in a restriction enzyme-digested vector, namely a pooled version of the established strategy we had developed for individual shRNA cloning (Bertero et al. 2018b). While this strategy had a ~92% cloning efficiency by colony PCR (Appendix Figure S1A), ~12.5% of plasmids carried large indels affecting the H1 promoter and/or shRNA. Unexpectedly, we observed shRNA BC swaps in 22% of plasmids despite not having introduced exogenous DNA polymerases (Appendix Table S2): this suggests that bacterial enzymes can also interfere with the process, possibly as a result of mis-annealed dsDNA that triggers DNA repair mechanisms by exposing bulges. shRNA representation was improved compared to the pilot experiment (Appendix

Figure S1B), but still not ideal. Of note, this strategy would have forced us to forego the use of UCIs given the impossibility of designing complementary sequences to random stretches of DNA. In all we dropped this avenue.

3. Amplification of ssDNA oligonucleotides by PCR using a DNA polymerase with strand displacement activity, followed by Gibson assembly. After testing several polymerases we focused on Deep Vent and Deep Vent(exo-), and we optimized protocols to obtain sufficient amounts of dsDNA from ssDNA oligonucleotides following only 10 cycles and 5 cycles of PCR, respectively (the exo- polymerase has a higher processivity at the expense of lower fidelity). Gibson assembly was extremely efficient: ~100% and ~97% for Deep Vent and Deep Vent(exo-), respectively (Appendix Figure S1A). While Deep Vent(exo-) resulted in a high rate of mutations, ~80% of plasmids obtained using Deep Vent carried the expected fragments (Appendix Table S2). This is marginally better than the fidelity of the strategy we ultimately adopted. Moreover, the representation of shRNAs in this condition was comparatively as good, being statistically in line with a Gaussian distribution with the expected median (Appendix Figure S1B). However, this comes at the expense of ~1.6-fold higher rate of barcode swap.
4. Second strand synthesis of ssDNA oligonucleotides using a DNA polymerase with strong strand displacement activity, followed by Gibson assembly. This strategy was designed to minimize shRNA amplification biases and barcode swapping arising from even a handful of PCR cycles. Having tested various enzymes we settled on a protocol based on Bst3.0, a DNA polymerase extensively used in isothermal methods due to a very strong strand displacement activity and a temperature optimum between 60-72°C. As described in the main text and shown in Figure 1B and Figure EV1B, this strategy proved both effective and sufficiently faithful, and is our preferred method due to the lower rate of shRNA BC swapping.

- Pooled cloning step 1 and/or 2 resulted in a high rate of aberrant plasmids, which being smaller integrated very efficiently during genome editing, outcompeting the correct vectors. Genotyping of hiPSC clones from the pilot experiment indicated that nearly half of them had integrated an unexpected sequence, as indicated by a ~2.7 kbp band in a PCR designed to amplify the wild type *AAVS1* locus (1692 bp band) or result in no amplification for alleles correctly targeted with the tet-ON system (due to the high GC content of the CAG promoter; Appendix Figure S1D). Sanger sequencing of the ~2.7 kbp band revealed that it contained only the puro resistance gene trap and lacked all elements of the tet-ON system (Appendix Figure S1E).

  We speculated that this aberrant sequence resulted from a plasmid whereby the two identical bovine growth hormone (bGH) pA signals downstream of the puro resistance and OPTtetR genes had recombined (Appendix Figure S1F). This hypothesis was supported by observations from cloning step 1, as negative control Gibson assemblies containing only the backbone resulted in a large number of ampicillin resistant bacterial clones, particularly after prolonged incubation times. Restriction digestion and Sanger sequencing analyses confirmed that these plasmids were consistent with the hypothesized recombination.

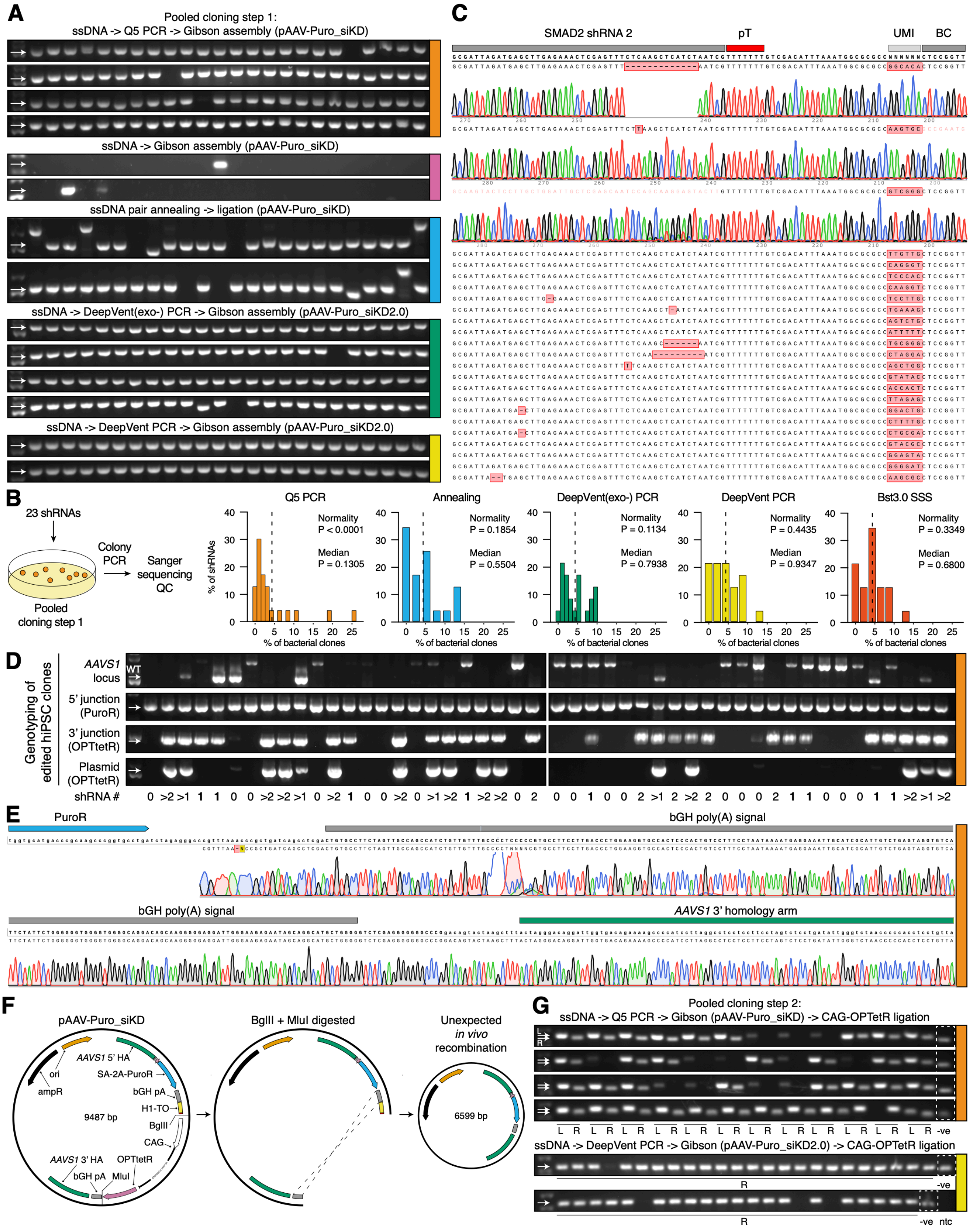

Appendix Figure S1. Optimization of molecular biology and genome editing steps for iPS2-seq
Legend on the next page

Appendix Figure S1. Optimization of molecular biology and genome editing steps for iPS2-seq (continued)

(A) Efficiency of pooled cloning step 1 following the indicated, color-coded strategies. Barcoded shRNA integration was measured in individual bacterial clones through colony PCR (refer to Figure EV1A); arrow = expected amplicon.
(B) Quantification of shRNA representation by Sanger sequencing of the bacterial colony PCR amplicons from panel A. The normality of distributions was tested by D'Agostino & Pearson test while the deviation of medians from the expected value (dashed lines) was tested by Wilcoxon signed-ranked test. N = 38-94 clones (Appendix Table S2)
(C) Sequence alignments for colony PCR amplicons associated to *SMAD2* shRNA 2 after "orange" pooled cloning step 1. Exemplar electropherograms indicate a large deletion and shRNA barcode swaps.
(D) Genotyping of hiPSC clones generated using plasmid pools obtained through "orange" pooled cloning steps 1 and 2. Distinctly from Appendix Figure 1A, only these targeting vectors were delivered, and genome edited cells were selected with puromycin. PCR strategies assayed the *AAVS1* locus (loss-of-allele implies biallelic targeting of the unamplifiable CAG promoter; a ~2.7 kbp amplicon indicates an unexpected sequence integration), the expected targeting cassette junctions (PuroR and OPTtetR confirm targeting of both ends of the inducible barcoded shRNA cassette), and random shRNA plasmid integrations; the number of shRNAs expressed by each clone is reported.
(E) Sanger sequencing results of the ~2.7 kbp amplicon from the *AAVS1* locus PCR from panel D; the sequence is aligned to the unexpected *in vivo* recombination plasmid described in panel F
(F) Schematic of the recombination between identical bovine growth hormone poly(A) (bGH pA) sequences in pAAV-Puro siKD prepared for pooled cloning step 1 by restriction enzyme digestion. A similar event can involve the intermediate plasmids prepared for pooled cloning step 2.
(G) As for panel A, but to measure the efficiency of pooled cloning step 2. For the "orange" strategy, two PCRs were performed: L: which amplifies also the parental vector, and R, which amplifies only the correct product; pAAV-Puro_siKD amplified with R (negative control, -ve) leads to a smaller amplicon.

Surprisingly, the same recombinant plasmids could be obtained with high frequency also after direct transformation of only the digested plasmid backbones from either cloning step 1 or 2, indicating that bacterial DNA repair mechanisms could also induce homology-mediated recombination *in vivo*. We note that such an event was not anticipated to happen at significant frequency given that the bGH pA signal downstream of the puro resistance is 227 bp and 288 bp away from the double strand break induced by restriction digestion in cloning steps 1 and 2, respectively. Nevertheless, ~27% of bacterial clones from cloning step 2 contained plasmids consistent with this bGH pA recombination (Appendix Figure S1G). It is not surprising that these smaller plasmids outcompeted the ones containing the larger tet-ON cassette during genome editing.

To overcome this problem, we modified the *AAVS1* targeting plasmid by substituting the bGH pA signal downstream of the puro resistance with the structurally unrelated SV40 pA signal. As described in the main text and shown in Figures EV1B and EV1C, cloning steps 1 and 2 using this plasmid, pAAV-Puro_siKD2.0 – which also includes additional modifications described below – no longer result in significant background transformation; accordingly, subsequent genome editing does not result in the aberrant sequences (Figure EV1D).

- Targeting of the *AAVS1* locus is so efficient that too many resulting clones were homozygous and thus likely to express more than one shRNA. The genotyping results of the pilot experiment also indicated that in ~85% of tested clones both alleles of the *AAVS1* locus had been edited (Appendix Figure S1D). Despite the competition represented by the smaller aberrant vector described above, approximately one third of clones had integrated two, presumably distinct, shRNAs. This clashed with our goal of expressing a single isogenic perturbation in most cells: while it is possible to filter out cells carrying two or more shRNAs bioinformatically, an excess proportion is a waste of resources invested in scRNA-seq and NGS.

  To increase the rate of hemizygous targeting of inducible barcoded shRNAs, we modified the genome editing protocol to include cotargeting of the second *AAVS1* allele with a filler plasmid car-

rying a second selection marker to enable coselection of biallelic targeted clones. As described in the main text and shown in Figure EV1D, this strategy is quite effective. We note that neo selection is tricky because of the narrow concentration range that enables selection of unedited cells while minimally interfering with the fitness of edited ones. We thus optimized a coselection protocol that uses a low dose of neo, and, accordingly, does not fully select biallelic editing. While this was not needed for our experiments, a more stringent neo selection can be employed if this is desired. Alternatively, other selection markers could be employed (such as blasticidin resistance and/or a fluorescent protein to enable to enable sorting of targeted clones).

- High rate of random integration of targeting plasmids further increase the chances of expressing more than one shRNA. One last important finding from the genotyping results of the pilot experiment was that approximately one third of clones carried targeting vector random integrations (Appendix Figure S1D). Since at least some of these integrations would result in transcriptionally competent shRNA cassettes, this also clashed with our goal of maximizing the number of cells carrying a single functional shRNA.

  We reasoned that this issue could be mitigated by introducing a negative selection cassette outside of the *AAVS1* homology arms in the targeting vector, so that clones carrying random integrations of the tet-ON system cassette and its backbone could be selected out. We modified our targeting vector accordingly: the backbone of pAAV-Puro_siKD2.0 contains a TK gene under the transcriptional control of the constitutive PGK promoter (Figure EV1A), to enable negative selection with FIAU. As described in the main text and shown in Figure EV1D, this strategy is effective. We note that this modification added ~1.7 kbp to the targeting vector to a total size of ~11 kbp. While our experiments demonstrate that this plasmid can be successfully transfected, a marginal improvement in transfection efficiency could be achieved by removing up to ~2.3 kbp of non essential sequences from the vector backbone outside of the *AAVS1* homology arms

Optimization of shRNA barcode detection in sci-RNA-seq

At the culmination of the iPS2-seq pilot described in the previous section, we performed an experiment using the standard, two-level indexing sci-RNA-seq protocol (Cao et al. 2017) on PFA-fixed hiPSC nuclei obtained after 4 days of tet treatment, to verify the efficiency of shRNA UMI-BCs detection from the unbiased transcriptome library. As it could be anticipated, only a small fraction of reads contained the reference sequence matching to the OPTtetR 3' UTR (1,796 out of 152 million, in a NGS run reaching ~95% saturation; data not shown). This likely resulted from the combination of three factors: (1) the intrinsic zero inflated nature of scRNA-seq, which results in a sizeable fraction of null transcript counts also for genes that are reasonably well expressed; (2) the particularly low sensitivity of 2-level indexing sci-RNA-seq, compared to the more recently optimized 3-level indexing protocol (Martin et al. 2023); (3) the specific location of the UCI-BC within the OPTtetR 3' UTR (Figure EV1E): this is ~130 bp away from the cleavage and polyadenylation site; thus, the UCI-BC is not sufficiently proximal to the start of read 2 in a large fraction of a typical sci-RNA-seq library (average insert size of ~250 bp).

We reasoned that we could increase the detection rate of UCI-BCs by enriching for a specific portion of the OPTtetR 3' UTR at two steps (Figure EV1E): (1) during RT, by adding a primer specific to the region at the 3' of UCI-BCs but otherwise identical to the conventional polythymidine (pT) oligo used in sci-RNA-seq; and (2) during PCR, by adding a primer specific to the region at the 5' of UCI-BCs and carrying both the Nextera Tn5 mosaic end (ME) and the conventional P7 primers used in sci-RNA-seq,

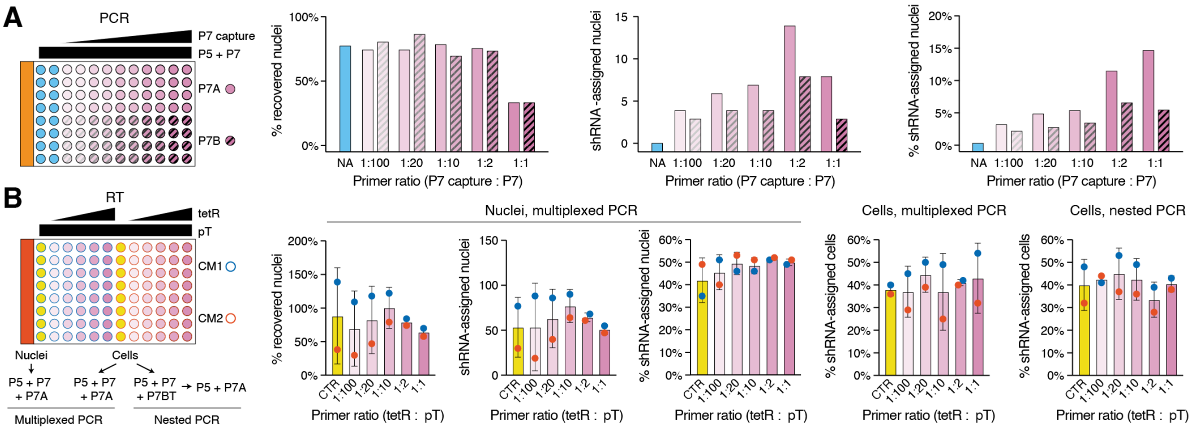

Appendix Figure S2. Optimization of iPS2-sci-seq

(A) Optimization of UCI-BC enrichment during PCR. Increasing amounts of one of two OPTtetR-specific primers were spiked in on top of standard primers in a sci-RNA-seq experiment in nuclei from the hiPSC pool described in Appendix Figure S1D. The proportion of nuclei recovered after transcriptome QC, the number of nuclei assigned to a single shRNA by *catcheR_scicatch* (UMI count threshold >2), and the proportion of recovered nuclei assigned to a unique shRNA are reported. (B) Optimization of UCI-BC enrichment during RT. Increasing amounts of a OPTtetR-specific primer were spiked in on top of standard pT in a sci-RNA-seq experiment with two batches of hiPSC-CMs (CM1-2) from the hiPSC pool described in Figure EV1D. PFA-fixed nuclei were compared to methanol-fixed cells; for cells, besides the multiplexed PCR method described in Figure EV1E, a nested PCR based on P7BT was also tested. The same metrics described for panel A are reported, but the UMI count threshold was >5. Error bars: SD..

so as to reconstitute the Nextera read 2 primer in inserts not modified by Tn5 transposase. Not only these strategies would be expected to increase the fraction of reads containing the UCI-BCs, but they would also place them in a precise position close to the start of read 2, ensuring their efficient sequencing even in a short read format and easing their precise bioinformatic identification (Figure EV1F).

We first set out to optimize PCR enrichment. We designed different primers with annealing portions specific to the OPTtetR cDNA at increasing distances from the 5' of UCI-BCs, and we tested them for their ability to efficiently amplify this region from Tn5-treated cDNA generated from tet-treated hiPSC nuclei. The two closest primers to the UCI-BCs proved the most efficient (data not shown), and were selected for further optimization. We denominated these primers P7A and P7B to distinguish them from the P7 primer used in conventional sci-RNA-seq (Figure EV1E). We performed a complete sci-RNA-seq experiment in which we tested various concentrations of P7A or P7B in subsets of PCR wells, adding them to the standard amounts of P5 and P7 primers (Appendix Figure S2A). Importantly, the indexes carried by each P7 primer (i7) were matched to those of P7A or P7B primers, to ensure that reads arising from OPTtetR and the rest of the transcriptome could be matched *in silico*. First, we checked whether addition of P7A or P7B negatively impacted the quality of standard sci-RNA-seq data, by examining the percentage of nuclei combinatorial barcodes passing quality filters. Molar ratios of P7A:P7 or P7B:P7 of 1:100 through 1:2 did not interfere, while an equimolar ratio resulted in a marked performance loss (Appendix Figure S2A). We then utilized *catcheR_scicatch* to match transcriptomes with unique shRNA perturbations using the same thresholds described in the main text to filter UCI-BCs, except for a less stringent UMI count cutoff of higher than 2 (since at this point the enrichment was not strong enough). This analysis confirmed that standard sci-RNA-seq is not sufficient to identify shRNA perturbations, but that addition of P7A or P7B improves this aspect, with the best result observed at a 1:2 ratio of P7A:P7 or P7B:P7 (Appendix Figure S2A). Since P7 outperformed P7B, we selected this primer and used it at a 1:2 ratio to P7 in our subsequent experiments. Of note, while only ~15% of quality-passing

nuclei could be assigned to a single shRNA (Appendix Figure S2A), this actually exceeded the minimal estimated fraction of hiPSC clones expressing a single shRNA in the clonal pool used for this optimization experiment (~8%; Appendix Figure S1D).

We then tested whether adding a second enrichment step during RT would further boost the capture rate of UCI-BCs. For this experiment we relied on the hiPSC clonal pool generated with the optimized cloning and gene editing protocol (Figure EV1D). In order to test the method beyond the iPSC state, we generated two biological replicates of hiPSC-CMs treated with tet from the onset of differentiation. These two batches of hiPSC-CMs are the same that we ultimately analyzed in the final experiment presented in Appendix Figure 1. From these cultures we generated both PFA-fixed nuclei and methanol-fixed cells in order to also compare the performance in these two established sample types (Cao et al. 2017). We designed an RT primer annealing at the 3' of the UCI-BCs, and obtained 96 copies with the same indexes of the sci-RNA-seq pT oligos; we denominated the OPTtetR-specific RT primer "tetR" (Figure EV1E). We then performed a complete sci-RNA-seq experiment on PFA-fixed nuclei in which subsets of RT wells were spiked in with various concentrations of index-matched tetR on top of the standard amount of pA; all subsequent PCR wells contained the previously optimized 1:2 ratio of P7A:P7, so that the only resulting variable would be the RT enrichment (Appendix Figure S2B). We then repeated the same assessments described for Appendix Figure S2A, except that to filter UCI-BCs we applied the more stringent UMI count cutoff of higher than 5. Molar ratios of tetR:pT up to 1:1 did not interfere with the quality of standard sci-RNA-seq data, while a 1:10 molar ratio proved optimal to improve the matching of transcriptomes to unique UCI-BCs (Appendix Figure S2B). In this condition, ~51% of nuclei passing quality filters could be assigned to a single shRNA (Appendix Figure S2B), an acceptable performance considering the estimated maximal fraction of hiPSC clones expressing a single shRNA in this clonal pool (~81%; Figure EV1D).

We also explored whether sci-RNA-seq on methanol-fixed cells, would result in improved UCI-BC detection, possibly as a result of increased transcript counts in whole cells compared to nuclei. We performed the same RT enrichment optimization experiment starting from this sample type, but the resulting assignment of transcriptomes to unique shRNA was both quite variable and on average less efficient than what could be achieved using nuclei (Appendix Figure S2B). Considering that in our hands methanol-fixed hiPSC-CMs were quite fragile during RT, complicating FACS and increasing the risk of mRNA cross-contamination, we decided against using this sample type in our final experiment. Nevertheless, these results demonstrate that the strategy we developed can also be applied in whole cells, which may prove advantageous for other cell types less sensitive than hiPSC-CMs.

Lastly, we evaluated whether a nested PCR strategy would improve the enrichment of UCI-BCs. For this, we repeated the RT enrichment optimization experiment, but instead of performing the multiplexed P5-P7 and P5-P7A PCR, we generated a separate UCI-BC library in two steps: (1) multiplexed PCR with P5, P7, and truncated variant of P7B (P7BT) without any Nextera sequences, so as to generate a standard sci-RNA-seq library containing non-sequenceable fragments with the UCI-BCs; and (2) re-amplifying the output of the previous reaction with P5 and P7A, using well-matched indexes with the original P7, so as to perform a nested PCR on the fragments containing UCI-BCs. This strategy was effective, but it did not lead to an improvement in the assignment of transcriptomes to unique shRNAs (Appendix Figure S2B). As the nested PCR requires more effort and reagents, we decided against adopting it for our subsequent experiments.

In summary, for the "iPS2-sci-seq" experiment described in the main text we selected the optimal dual enrichment protocol during RT (1:10 of tetR:pT) and multiplexed PCR (1:2 of P7A:P7) using PFA-fixed nuclei as starting material.

Optimization of shRNA barcode detection in 10X Genomics sc-RNA-seq

In parallel to the second optimization round of iPS2-sci-seq described in the previous section (Appendix Figures S2A and S2B), we pursued the alternative strategy of utilizing microfluidics sc-RNA-seq to increase overall UMI count, with the expectation that this would ease the reliable measurement of UCI-BCs. Specifically, we leveraged on the 10X Genomics Next GEM 3' v3.1 chemistry, which tags the 3' ends of all mRNAs from a given cell with the same 18 bp unique barcode.

We analyzed the same two batches of tet-treated day 23 hiPSC-CMs (Figure EV1G), as well as the two matching pools of genome edited hiPSCs (subjected to four days of tet treatment in pluripotency media); in both cases, we pooled equal amounts of cells from each batch in a single microfluidics reaction with 16,000 cells in total. Having obtained standard gene expression libraries following the manufacturer's protocol, we tested two avenues to obtain separate libraries enriched for UCI-BCs starting from pre-amplified, cell-barcoded cDNA: (1 - nested) a three-step PCR involving a two-step nested PCR with outer and inner primers specific for the OPTtetR 3' end (paired to the same reverse primer on the i5 TruSeq adapter inserted during RT), followed by NGS library prep; and (2 - direct) a two-step strategy that omits the first PCR with the outer primer (Figure EV2A). In both cases the inner primer carries the i7 TruSeq adapter that is otherwise inserted by the manufacturer's protocol through ligation, to enable library prep using the standard manufacturer's dual indexed P5 and P7 primers. The nested strategy was inspired by the one employed in an optimized CROP-seq method (Hill et al. 2018), while the direct approach was tested as a potential simpler alternative with lower PCR duplication rates.

We identified optimal annealing temperatures for outer and inner PCRs, and the minimal cycle number that resulted in a clean amplicon at each step (data not shown; Appendix Protocol 3). We then sequenced equal amounts of the resulting "nested" and "direct" UCI-BC libraries in parallel to the standard gene expression ones (pooled at a 1:100 ratio), and analyzed the results using the *catcheR_10Xcatch* pipeline using the same thresholds for UCI-BC quantification employed for iPS2-sci-seq except for a more stringent UMI count cutoff of higher than 10. Both enrichment strategies supported the assignment of ~60% of cells to at least one shRNA in both hiPSCs and hiPSC-CMs with a high degree of confidence (mean UMI of valid UCI-BCs of ~90 and ~180, respectively; Figure EV2B). The separation between UCI-BCs associated to single shRNA integrations and background noise or multiple integration was even clearer than what observed for iPS2-sci-seq (compare Figure EV2C to Appendix Figure 1E), and the fraction of cells assigned to a single shRNA was marginally lower (~75% *vs.* ~80%; compare Figure EV2D to Appendix Figure 1F), still well in line with our estimation based on genotyping of a subset of hiPSC clones from these genome edited pools (Figure EV1D). As both nested and direct UCI-BC libraries were of good quality, we merged them and re-run *catcheR_10Xcatch*, identifying a total of 3,672 hiPSCs and 2,670 hiPSC-CMs associated to a single shRNA (out of a total of 6,297 and 4,336 cells, respectively; Figure EV2E). These included all clones previously identified by iPS2-sci-seq (Appendix Figure 1G). Since the fraction of cells assigned to individual shRNAs did not increase further by merging nested and direct UCI-BC libraries (presumably since they captured the same, limiting number of OPTtetR mRNAs), for subsequent experiments we only utilized the simpler direct strategy, which is our reference protocol for "iPS2-10X-seq".

Identification of cells expressing no shRNA

While exploring the NGS results from the UCI-BC libraries of iPS-10X-seq we noticed that besides the expected 23 bp "reference" sequence at the start of read 2, matching to the region upstream of the UCI-BC, a small but sizeable number of reads contained a similar sequence that differed only in the last two

nucleotides (Figure EV2F). We noticed that such "wrong reference" sequence aligns to the unmodified parental pAAV-Puro_siKD2.0. Accordingly, nearly all of the putative 14 bp UCI-BC that followed the wrong reference continued to match to pAAV-Puro_siKD2.0 (instead of containing the expected 6 random bases followed by an 8 bp shRNA barcode). We concluded that a small proportion of cells had integrated a cassette without any shRNA (presumably due to a small contamination of pAAV-Puro_siKD2.0 during gel extraction of the CAG-OPTtetR sequence in pooled cloning step 2; Figure EV1A).

Cells not expressing an shRNA can be a useful additional control for potential non-specific effects of expressing any shRNA (i.e., interference with the processing of endogenous miRNA). Thus, we developed *catcheR_10Xnocatch*, which takes the cells discarded by *catcheR_10Xcatch* as having no valid integration of UCI-BCs, and checks whether they instead express high levels of the wrong reference matching to pAAV-Puro_siKD2.0. If so, these cells are rescued and assigned the "empty" label, so they can be analyzed in parallel to cells expressing individual shRNAs. In our experiment in hiPSCs and hiPSC-CMs, we found ~7% and ~9% empty cells, respectively. This corresponded to ~1.26% of plasmids from cloning pooled step 2 carrying this sequence, as could be readily assessed using *catcheR_step2QC* function.

Of note, while a small proportion of empty cells can be a useful feature, a larger fraction can be wasteful. Our cloning strategy has a built in safeguard for this eventuality, namely digesting the plasmid pool from cloning step 2 with BglII and/or MluI: these enzymes only cut pAAV-Puro_siKD2.0 but not the plasmids that correctly integrated an shRNA and UCI-BC after pooled cloning step 1 (Figure EV1A and Appendix Protocol 1).

Appendix Methods

Plasmids

Several alternative approaches for the first cloning step were tested (Appendix Figures S1A–S1C). First, PCR of ssDNA oligo pools with Q5 Hot Start High-Fidelity DNA polymerase (10 fmol template and 50 µM iPS2-seq_dsDNA_F/R primers, 1 U of enzyme in 1X reaction buffer, 20 cycles annealing at 60 °C for 30 s and extending at 72 °C for 30 s), gel extraction (2% agarose TBE), and assembly with NEBuilder HiFi DNA Assembly Master Mix (10 fmol plasmid and 0.2 pmol dsDNA, 15 min at 50 °C). Second, direct Gibson assembly of ssDNA oligo pool (10 fmol plasmid and 0.2 pmol dsDNA, 60 min at 50 °C). Third, annealing of two pairs of complementary ssDNA oligo pools designed, annealed, phosphorylated, and ligated as previously described (Bertero et al. 2018b); these encoded the same sequence except for lacking random UCIs. Fourth, PCR of ssDNA oligo pools with Deep Vent polymerase (5 fmol template and 50 µM iPS2-seq_dsDNA_F/R primers, 1 U of enzyme in 1X reaction buffer with 6 mM $MgSO_4$, 10 cycles, annealing at 55 °C and extending at 72 °C for 30 s), gel extraction (2% agarose TBE), and NEBuilder HiFi DNA Assembly Master Mix (10 fmol plasmid and 0.2 pmol dsDNA, 15 min at 50 °C).

Genome editing

For our pilot experiment (Appendix Figure S1D), we followed a protocol similar to the one described in the main text, except that only the pool of pAAV-Puro_siKD with the barcoded shRNAs was co-transfected with ZFN plasmids, and that clones were selected only with $1\,\mu g\,mL^{-1}$ puromycin. Clones were picked and genotyped according to the same protocol except that the *AAVS1* locus PCR was extended for 3 min to allow amplification of an unexpected ~2.7 kbp fragment, which was PCR purified and sequenced using primer 5'-TGGTGCATGACCCGCAA-3'.

iPS2-sci-seq

Optimization experiments that led to the final protocol tested multiple variables. First, two OPTtetR-specific primers were evaluated for multiplexed library PCR, P7A and P7B, which were either omitted (only P5 and P7) or spiked in, separately, at increasing concentrations: 5 nM, 25 nM, 50 nM, 250 nM, or 500 nM (same concentration of P7). This 96 x 96 indexes experiment was performed using hiPSC-CM nuclei from the gene editing pilot experiment, and did not include the tetR primer during RT. Second, the tetR primer was either omitted or added at varying concentrations during RT: 0.05 µM, 0.25 µM, 0.5 µM, 2.5 µM, or 5 µM (same concentration of pT). This 96 x 96 indexes experiment was performed using hiPSC-CMs from the two final genome-edited pools, and included the P7A primer during PCR. The same experiment tested two additional variables: cells *versus* nuclei, and multiplexed *versus* nested library prep PCR. For this, cells were fixed with ice-cold methanol for 15 min, washed, and then processed in parallel to PFA-fixed nuclei to generate a comparable multiplexed library PCR. In parallel, the cells were subjected to a different multiplexed library PCR whereby P5 and P7 were supplemented with a variant of P7B lacking the Illumina adapter sequence, to obtain a gene expression library with pre-amplified, but not sequenceable, UCI-BCs. 5 µL of each PCR was then transferred to a new plate and treated with 10 U exonuclease I and 1 U recombinant shrimp alkaline phosphatase to remove the old primers (30 min at 37 °C and 15 min at 80 °C to heat inactivate). 1 µL of cleaned up PCR was transferred to a new plate and used as template for UCI-BC library prep PCR using 500 nM P5 and 500 nM P7A (using i7 indexes that matched those of the original gene expression library PCR, well by well, and different i5 indexes).

iPS2-10X-seq

During the optimization experiment, a second strategy was tested: a nested PCR with an outer primer followed by the same inner primer PCR. Both amplicon types were utilized for library prep PCR with dual indexed primers.

Appendix Tables

Gene	CHD involvement	shRNA ID	Sequence ID	Potency (KD)	Target sequence	Barcode(s)	ssDNA for pooled cloning step 1	Clones in pool
GATA4	ASD, VSD, AVSD, PVS, TOF	GATA4.1	TRCN0000020424	94%	CCAGAGATTCTGCAACACGAA	TCTCGCCT; GCCGAATG	AGTTCCCTATCAGTGATAGAGATCCCGCCAGAGATTCTGCAACACGAA-<br>CTCGAGTTCGTGTTGCAGAATCTCTGGTTTTTTTGTCGACATTTAAAT-<br>GGCGCGCCNNNNNNTCTCGCCTGTAGCTCGCTGATCAGC	10
		GATA4.2	TRCN0000329713	91%	GGACATAATCACTGCGTAATC	GCTGAAGA	AGTTCCCTATCAGTGATAGAGATCCCGGACATAATCACTGCGTAATC-<br>CTCGAGGATTACGCAGTGATTATGTCCTTTTTTTGTCGACATTTAAAT-<br>GGCGCGCCNNNNNNGCTGAAGAGTAGCTCGCTGATCAGC	0
		GATA4.3	TRCN0000329769	80%	CGAGGAGATGCGTCCCATCAA	TATCTGTG	AGTTCCCTATCAGTGATAGAGATCCCGCGAGGAGATGCGTCCCATCAA-<br>CTCGAGTTGATGGGACGCATCTCCTCGTTTTTTTGTCGACATTTAAAT-<br>GGCGCGCCNNNNNNTATCTGTGGTAGCTCGCTGATCAGC	4
NKX2-5	ASD, AVCD, TOF, HLHS	NKX2-5.1	TRCN0000013736	97%	GCCTCAATCCCTACGGTTATA	TACTGCAG; CATGTTGA	AGTTCCCTATCAGTGATAGAGATCCCGCCTCAATCCCTACGGTTATA-<br>CTCGAGTATAACCGTAGGGATTGAGGCTTTTTTTGTCGACATTTAAAT-<br>GGCGCGCCNNNNNNTACTGCAGGTAGCTCGCTGATCAGC	2
		NKX2-5.2	TRCN0000013734	71%	CGCCAACAACAACTTCGTGAA	GCATCACG	AGTTCCCTATCAGTGATAGAGATCCCGCGCCAACAACAACTTCGTGAA-<br>CTCGAGTTCACGAAGTTGTTGTTGGCGTTTTTTTGTCGACATTTAAAT-<br>GGCGCGCCNNNNNNGCATCACGGTAGCTCGCTGATCAGC	2
		NKX2-5.3	TRCN0000013733	70%	CGGCGATTATGCAGCGTGCAA	CTGAATCA	AGTTCCCTATCAGTGATAGAGATCCCGCGGCGATTATGCAGCGTGCAA-<br>CTCGAGTTGCACGCTGCATAATCGCCGTTTTTTTGTCGACATTTAAAT-<br>GGCGCGCCNNNNNNCTGAATCAGTAGCTCGCTGATCAGC	5
SMAD2	DORV, CAVC, ASD, VSD, PDA, HLHS	SMAD2.1	TRCN0000010477	81%	CAAGTACTCCTTGCTGGATTG	GGCTCTTG	AGTTCCCTATCAGTGATAGAGATCCCGCAAGTACTCCTTGCTGGATTG-<br>CTCGAGCAATCCAGCAAGGAGTACTTGTTTTTTTGTCGACATTTAAAT-<br>GGCGCGCCNNNNNNGGCTCTTGGTAGCTCGCTGATCAGC	1
		SMAD2.2	TRCN0000040036	78%	CGATTAGATGAGCTTGAGAAA	CTCCGGTT	AGTTCCCTATCAGTGATAGAGATCCCGCGATTAGATGAGCTTGAGAAA-<br>CTCGAGTTTCTCAAGCTCATCTAATCGTTTTTTTGTCGACATTTAAAT-<br>GGCGCGCCNNNNNNCTCCGGTTGTAGCTCGCTGATCAGC	1
		SMAD2.3	TRCN0000040037	77%	CCTAAGTGATAGTGCAATCTT	CGAACTTC	AGTTCCCTATCAGTGATAGAGATCCCGCCTAAGTGATAGTGCAATCTT-<br>CTCGAGAAGATTGCACTATCACTTAGGTTTTTTTGTCGACATTTAAAT-<br>GGCGCGCCNNNNNNCGAACTTCGTAGCTCGCTGATCAGC	2
		SMAD2.4	TRCN0000040035	73%	GCGTTGCTCAAGCATGTCATA	AGTTCCGC	AGTTCCCTATCAGTGATAGAGATCCCGCGTTGCTCAAGCATGTCATA-<br>CTCGAGTATGACATGCTTGAGCAACGCTTTTTTTGTCGACATTTAAAT-<br>GGCGCGCCNNNNNNAGTTCCGCGTAGCTCGCTGATCAGC	3
KMT2D	Kabuki syndrome, CoA, BAV, VSD, TOF, TGA, HLHS	KMT2D.1	TRCN0000013138	61%	CCCACCTGAATCATCACCTTT	GAGATTGT	AGTTCCCTATCAGTGATAGAGATCCCGCCCACCTGAATCATCACCTTT-<br>CTCGAGAAAGGTGATGATTCAGGTGGGTTTTTTTGTCGACATTTAAAT-<br>GGCGCGCCNNNNNNGAGATTGTGTAGCTCGCTGATCAGC	2
		KMT2D.2	TRCN0000013140	51%	CCTCGCCTCAAGAAATGGAAA	TGCTCCGA	AGTTCCCTATCAGTGATAGAGATCCCGCCTCGCCTCAAGAAATGGAAA-<br>CTCGAGTTTCCATTTCTTGAGGCGAGGTTTTTTTGTCGACATTTAAAT-<br>GGCGCGCCNNNNNNTGCTCCGAGTAGCTCGCTGATCAGC	2
		KMT2D.3	TRCN0000235743	Unknown	CGTAGAAGAGGACCTACTAAT	ATACGGAT	AGTTCCCTATCAGTGATAGAGATCCCGCGTAGAAGAGGACCTACTAAT-<br>CTCGAGATTAGTAGGTCCTCTTCTACGTTTTTTTGTCGACATTTAAAT-<br>GGCGCGCCNNNNNNATACGGATGTAGCTCGCTGATCAGC	1
		KMT2D.4	TRCN0000235745	Unknown	AGCACATGGAGTGCGAAATTA	ATGGTCTC	AGTTCCCTATCAGTGATAGAGATCCCAGCACATGGAGTGCGAAATTA-<br>CTCGAGTAATTTCGCACTCCATGTGCTTTTTTTGTCGACATTTAAAT-<br>GGCGCGCCNNNNNNATGGTCTCGTAGCTCGCTGATCAGC	1
CHD7	CHARGE syndrome; TOF, PDA, DORV, AVSD, VSD	CHD7.1	TRCN0000234550	Unknown	GAGAGTTCCAGGGAGTATAAA	GAAAGAAG	AGTTCCCTATCAGTGATAGAGATCCCGAGAGTTCCAGGGAGTATAAA-<br>CTCGAGTTTATACTCCCTGGAACTCTCTTTTTTTGTCGACATTTAAAT-<br>GGCGCGCCNNNNNNGAAAGAAGGTAGCTCGCTGATCAGC	1
		CHD7.2	TRCN0000234553	Unknown	AGCGGAAAGGGAAGCTATTAT	TTGAGTAC	AGTTCCCTATCAGTGATAGAGATCCCAGCGGAAAGGGAAGCTATTAT-<br>CTCGAGATAATAGCTTCCCTTTCCGCTTTTTTTGTCGACATTTAAAT-<br>GGCGCGCCNNNNNNTTGAGTACGTAGCTCGCTGATCAGC	0
		CHD7.3	TRCN0000234552	Unknown	GCCTATCAGCGCAGCTATAAA	ATGACGAA	AGTTCCCTATCAGTGATAGAGATCCCGCCTATCAGCGCAGCTATAAA-<br>CTCGAGTTTATAGCTGCGCTGATAGGCTTTTTTTGTCGACATTTAAAT-<br>GGCGCGCCNNNNNNATGACGAAGTAGCTCGCTGATCAGC	1
		CHD7.4	TRCN0000234554	Unknown	GTATTGTGCAGTGCATTATTT	TGAGAGAT	AGTTCCCTATCAGTGATAGAGATCCCGTATTGTGCAGTGCATTATTT-<br>CTCGAGAAATAATGCACTGCACAATACTTTTTTTGTCGACATTTAAAT-<br>GGCGCGCCNNNNNNTGAGAGATGTAGCTCGCTGATCAGC	2
Controls	NA	SCR	SHC016	NA	GCGCGATAGCGCTAATAATTT	ACACAGGC	AGTTCCCTATCAGTGATAGAGATCCCGCGCGATAGCGCTAATAATTT-<br>CTCGAGAAATTATTAGCGCTATCGCGCTTTTTTTGTCGACATTTAAAT-<br>GGCGCGCCNNNNNNACACAGGCGTAGCTCGCTGATCAGC	1
		EGFP	SHC005	95%	TACAACAGCCACAACGTCTAT	AGCCTACT	AGTTCCCTATCAGTGATAGAGATCCCGTACAACAGCCACAACGTCTAT-<br>CTCGAGATAGACGTTGTGGCTGTTGTATTTTTTTGTCGACATTTAAAT-<br>GGCGCGCCNNNNNNAGCCTACTGTAGCTCGCTGATCAGC	0
		B2M	SHC008	80%	CAGCAGAGAATGGAAAGTCAA	CAGTTCCA	AGTTCCCTATCAGTGATAGAGATCCCGCAGCAGAGAATGGAAAGTCAA-<br>CTCGAGTTGACTTTCCATTCTCTGCTGTTTTTTTGTCGACATTTAAAT-<br>GGCGCGCCNNNNNNCAGTTCCAGTAGCTCGCTGATCAGC	2

Appendix Table S1. Disease relevance of genes selected for loss of function perturbations
Sequence IDs and potency of knockdown (KD) from the TRC data portal (Moffat et al. 2006) and the Sigma-Aldrich shRNA database. CHD acronyms: ASD, atrial septal defect; AVCD, atrioventricular conduction delay; AVSD, atrioventricular septal defect; BAV, bicuspid aortic valve; CAVC, complete atrioventricular canal defect; CHARGE, coloboma, heart defects, choanal atresia, retarded growth and development, genital anomalies, and ear anomalies; CoA, coarctation of the aorta; DORV, double-outlet right ventricle; HLHS, hypoplastic left heart syndrome; PDA, patent ductus arteriosus; PVS, pulmonary stenosis; TGA, transposition of great arteries; TOF, tetralogy of Fallot; VSD, ventricular septal defect.

	ssDNA -> Q5 PCR-> Gibson assembly		ssDNA pair annealing -> ligation		ssDNA -> DeepVent(exo-) PCR-> Gibson assembly		ssDNA -> DeepVent PCR-> Gibson assembly		ssDNA -> Bst3.0 SSS -> Gibson assembly	
	Clones	% of parent	Clones	% of parent	Clones	% of parent	Clones	% of parent	Clones	% of parent
All screened	96		48		96		48		48	
A - No shRNA	2	2%	4	8%	2	2%	0	0%	1	2%
B - shRNA	94	98%	44	92%	94	98%	48	100%	47	98%
B.1 - Inconclusive sequencing	2	2%	8	18%	25	27%	2	4%	1	2%
B.2 - Conclusive sequencing	92	98%	36	82%	69	73%	46	96%	46	98%
B.2.1 - Barcode swap	15	16%	8	22%	9	13%	8	17%	5	11%
B.2.2 - Missing barcode	0	0%	0	0%	0	0%	0	0%	0	0%
B.2.2 - Mutated barcode	0	0%	1	3%	2	3%	0	0%	0	0%
B.2.4 - Mutated shRNA	41	45%	8	22%	17	25%	1	2%	7	15%
B.2.5 - Correct fragment	36	39%	19	53%	41	59%	37	80%	34	74%

Appendix Table S2. Sanger sequencing QC of pooled cloning step 1 optimization experiments

Quantification of results from Sanger sequencing of colony PCRs reported in Figure EV1B and Figure S1A.

Protocol	Sample	Total reads, GEX (M)	All cells	Average GEX reads/cell	Median UMI per cell	Dup. rate	Mean genes per cell	Total reads, UCI-BC (M)	UMI threshold for UCI-BC	Mean UMI BC	Cells with 1 or more shRNA	Cells with 1 shRNA	Empty cells	Total usable cells	% Usable cells	% Cells with 2 or more shRNA
iPS2-sci-seq	Day 23 CMs	368	6872	53480	1769	90.3%	1018	/	5	15	3971	3465	104	3569	52%	7%
iPS2-10X-seq	hiPSCs	421	6297	66793	35601	14.2%	6923	10	10	156	5255	3716	433	4149	66%	24%
iPS2-10X-seq	Day 23 CMs	531	4336	122571	51715	42.3%	6083	11	10	74	3355	2690	386	3076	71%	15%
iPS2-10X-seq	Day 0 iPSCs	342	12299	27770	10747	36.1%	3425	11	10	63	9654	7181	852	8033	65%	20%
iPS2-10X-seq	Day 2 MES	322	11926	27005	8442	39.4%	2968	10	10	55	10215	7585	552	8137	68%	22%
iPS2-10X-seq	Day 6 CPs	290	13755	21049	6196	38.7%	2720	10	10	40	7787	5783	734	6517	47%	15%
iPS2-10X-seq	Day 12 CMs	306	11877	25724	6763	38.6%	3004	8	10	79	7336	5500	852	6352	53%	15%
iPS2-10X-seq	CTR cardioid	242	3734	30419	16874	37.43%	4634	22	10	56	2673	2600	242	2842	76%	2%
iPS2-10X-seq	TET cardioid		3918	32833			4881		10	50	2883	2814	204	3018	77%	2%
iPS2-10X-seq	CTR neurons	286	8628	21130	6138	31.47%	2182	17	10	34	4301	3840	140	3980	46%	5%
iPS2-10X-seq	TET neurons		5578	26919			2509		10	41	3873	3563	65	3628	65%	6%
iPS2-multi-seq	hiPSCs	452	6051	74817	16105	52.2%	4977	76	6	20	3208	1522	140	1662	27%	28%
iPS2-CITE-seq	B2M, SMAD2 CTR	365	6060	23358	11545	35.2%	4127	42	10	59	4902	4166	0	4166	69%	12%
iPS2-CITE-seq	B2M, SMAD2 KD		5395	22062			3914		10	58	3921	3309	0	3309	61%	11%

Appendix Table S3. Key figures of iPS2-seq NGS experiments

Protocol	iPS2-sci-seq	iPS2-10X-seq	iPS2-10X-seq	iPS2-10X-seq	iPS2-10X-seq	iPS2-10X-seq	iPS2-10X-seq	iPS2-10X-seq	iPS2-10X-seq	iPS2-10X-seq	iPS2-10X-seq	iPS2-multi-seq
	Day 23 CMs	hiPSCs	Day 23 CMs	Day 0 iPSCs	Day 2 MES	Day 6 CPs	Day 12 CMs	CTR cardioid	TET cardioid	CTR neurons	TET neurons	hiPSCs
Per gene												
B2M	52	74	46	189	201	143	127	55	43	26	12	13
CHD7	113	279	95	477	373	268	242	165	315	157	604	57
GATA4	1238	1607	1083	2919	3312	2616	2550	1141	1295	1581	983	274
KMT2D	357	456	253	805	647	611	454	245	194	306	166	52
NKX2-5	661	520	406	1007	1094	1029	1067	452	454	909	1547	75
SCR	121	164	208	293	420	379	317	141	125	126	108	33
SMAD2	923	616	551	1491	1547	738	767	499	455	735	143	98
Per shRNA												
B2M	52	74	46	189	201	143	127	55	43	26	12	13
CHD7.1	90	85	60	125	173	139	147	66	50	0	84	19
CHD7.3	3	18	10	41	37	14	18	3	12	0	40	5
CHD7.4	20	176	25	311	163	115	77	96	253	146	480	33
GATA4.1	441	841	452	1469	1551	1188	1220	489	657	750	569	184
GATA4.3	797	766	631	1450	1761	1428	1330	652	638	831	414	90
KMT2D.1	272	190	170	411	348	340	260	166	142	258	133	24
KMT2D.2	61	108	72	152	163	145	110	53	41	47	33	16
KMT2D.3	23	144	6	201	123	111	66	21	10	1	0	12
KMT2D.4	1	14	5	41	13	15	18	5	1	0	0	0
NKX2-5.1	18	39	8	89	51	36	27	17	18	5	5	4
NKX2-5.2	605	243	355	534	899	812	932	311	383	735	1120	26
NKX2-5.3	38	238	43	384	144	181	108	124	53	169	422	45
SCR	121	164	208	293	420	379	317	141	125	126	108	33
SMAD2.1	9	32	6	74	40	6	7	17	21	4	0	5
SMAD2.2	100	124	85	227	410	164	137	56	142	84	68	47
SMAD2.3	22	34	6	56	37	20	29	4	6	7	3	6
SMAD2.4	792	426	454	1134	1060	548	594	422	286	640	72	40

Appendix Table S4. Number of cells per perturbation

iPS2-10X-seq													
**Timepoint**	**Cluster**	***NKX2-5***		***GATA4***		***B2M***		***SMAD2***		***KMT2D***		***CHD7***	
		**CTR (SCR)**	**TET**	**CTR (SCR)**	**TET**	**CTR (SCR)**	**TET**	**CTR (SCR)**	**TET**	**CTR (SCR)**	**TET**	**CTR (SCR)**	**TET**
Day 0 iPSC	iPSC	0.0000	0.0009	0.0000	0.0006	0.3500	0.1811	0.2230	0.2252	0.0576	0.0715	0.3432	0.1562
	iPSC-neuro	0.0000	0.0128	0.0000	0.0011	0.3945	0.1419	0.4294	0.2303	0.0676	0.0520	0.4859	0.2159
Day 2 mesoderm	Mesoderm	0.0000	0.0011	0.2872	0.2653	0.2792	0.1030	0.3176	0.2478	0.0754	0.0774	0.3977	0.2187
	Primitive streak	0.0000	0.0008	0.0826	0.0876	0.2880	0.0949	0.2305	0.2051	0.0786	0.0645	0.3671	0.2071
Day 6 cardiac progenitors	Cardiac stroma	0.1026	0.0000	0.3632	0.2122	0.6328	0.3071	0.2476	0.1845	0.0000	0.0000	0.2822	NA
	CP early	0.1098	0.0599	0.4796	0.4424	0.5256	0.2831	0.5164	0.4504	0.0952	0.1093	0.4466	0.3594
	CP late	0.0920	0.0665	0.3434	0.3397	0.6149	0.3785	0.3066	0.2506	0.0388	0.0710	0.2581	0.1151
Day 12 cardiomyocytes	Cardiac stroma	0.1568	0.1432	0.5779	0.5461	0.6249	0.2825	0.3934	0.3714	0.0773	0.0791	0.3366	0.2472
	CP early	0.1341	0.1517	0.3011	0.3986	0.6330	0.2823	0.3853	0.4138	0.0432	0.1677	0.4144	0.3201
	CP late	0.2550	0.0615	0.1387	0.0820	0.6440	NA	0.0000	0.2673	0.0000	0.0000	0.0000	0.4660
	CP proliferating	0.0000	0.1343	0.0000	0.0040	0.4340	0.1396	0.2230	0.2582	0.0395	0.0000	0.5666	0.4509
	CM	0.4840	0.4682	0.7012	0.6472	0.5863	0.3295	0.4920	0.4183	0.0911	0.0908	0.3024	0.2601
Day 23 cardiomyocytes	Cardiac stroma	0.3243	0.2023	0.6553	0.4868	0.9127	0.3567	0.2933	0.2973	0.0702	0.1073	0.2594	0.3060
	CP early	0.0000	0.1765	0.0000	0.1858	0.5006	0.7284	0.3206	0.3010	0.0848	0.0643	0.5555	NA
	CP late	0.7096	0.6516	0.3212	0.2391	0.3769	0.0000	0.0000	0.1410	0.0000	0.0000	0.1726	NA
	CP proliferative	0.0000	0.0407	0.0461	0.0401	0.6632	0.2017	0.3064	0.3214	0.0986	0.0560	0.5241	NA
	CM	0.7436	0.7337	0.8375	0.8210	0.7154	0.5217	0.2749	0.2455	0.1028	0.0971	0.2840	0.2433

iPS2-sci-seq													
**Timepoint**	**Cluster**	***NKX2-5***		***GATA4***		***B2M***		***SMAD2***		***KMT2D***		***CHD7***	
		**CTR (SCR)**	**TET**	**CTR (SCR)**	**TET**	**CTR (SCR)**	**TET**	**CTR (SCR)**	**TET**	**CTR (SCR)**	**TET**	**CTR (SCR)**	**TET**
Day 23 cardiomyocytes	Cardiac stroma	0.2191	0.0155	0.3016	0.2055	0.0511	0.0546	0.1064	0.0596	0.0000	0.0000	0.0437	0.2658
	CM1	0.1103	0.0841	0.2330	0.2866	0.0338	0.0393	0.0824	0.0441	0.0084	0.0093	0.0467	0.0099
	CM2	0.1054	0.1059	0.2218	0.2417	0.0814	0.0850	0.0550	0.0324	0.0000	0.0118	0.0471	0.0199
	CM3	0.1568	0.1139	0.2952	0.2972	0.0428	0.0463	0.0848	0.0501	0.0078	0.0309	0.0464	0.0956
	CM proliferating	0.0854	0.1225	0.2615	0.2417	0.1909	0.1940	0.0390	0.0511	0.0157	0.0622	0.0344	0.0135

iPS2-10X-seq													
**Timepoint**	**Cluster**	***NKX2-5***		***GATA4***		***B2M***		***SMAD2***		***KMT2D***		***CHD7***	
		**CTR**	**TET**	**CTR**	**TET**	**CTR**	**TET**	**CTR**	**TET**	**CTR**	**TET**	**CTR**	**TET**
Day 7.5 Cardioids	ME	0.5248	0.4911	0.7427	0.6957	0.7895	0.5183	0.2211	0.0997	0.0220	0.0779	0.2123	0.1004
	CM-P	0.2166	0.2692	0.7916	0.7542	0.6525	0.1934	0.3360	0.3463	0.1399	0.0725	0.4148	0.4006
	CM-E	0.5458	0.5216	0.9246	0.8430	0.4756	0.2147	0.3966	0.3891	0.1021	0.1047	0.4744	0.3388
	CM-L	0.8025	0.7841	0.6149	0.5704	0.8495	0.4287	0.2461	0.1907	0.0643	0.0742	0.2319	0.1924
	CP	0.5202	0.4704	0.9458	0.8522	0.5164	0.4135	0.4526	0.4120	0.1277	0.1199	0.4605	0.3069
	MES	0.5365	0.5086	0.7039	0.6051	0.7935	0.5522	0.3681	0.3590	0.1448	0.1274	0.0000	0.2987
	CF	0.1604	0.1607	0.8285	0.7873	0.7580	0.5454	0.3290	0.3614	0.1089	0.0599	0.4248	0.3647
	EC	0.0949	0.1232	0.8685	0.7649	0.1030	0.4327	0.3679	0.3440	0.1025	0.1428	0.3853	0.5201
	ENDO	0.0580	0.0178	0.5946	0.3677	NA	0.4280	0.6563	NA	0.0000	0.1360	0.7235	0.3398

iPS2-10X-seq													
**Timepoint**	**Cluster**	***NKX2-5***		***GATA4***		***B2M***		***SMAD2***		***KMT2D***		***CHD7***	
		**CTR**	**TET**	**CTR**	**TET**	**CTR**	**TET**	**CTR**	**TET**	**CTR**	**TET**	**CTR**	**TET**
Day 30 Neurons	NC	NA	NA	0.0000	0.0000	NA	NA	0.1431	0.1943	0.0000	0.0550	0.3018	0.1669
	aRG	NA	NA	0.0000	0.0000	0.0000	0.2468	0.1276	0.0936	0.0171	0.0717	0.2149	0.3575
	Glut-Prog	NA	NA	0.0000	0.0000	NA	0.2402	0.0716	0.1271	0.0000	0.0000	0.7737	0.0000
	Ctx-Prog	NA	NA	0.0000	0.0000	0.1527	0.4592	0.1557	0.0893	0.0220	0.0305	0.1938	0.1770
	Fib	NA	NA	0.0000	0.0050	NA	NA	0.2460	0.0416	0.0000	0.0523	0.1005	0.1213
	pmNC	NA	NA	0.0000	0.0000	NA	NA	0.0000	0.1190	NA	0.0471	0.2540	0.3081
	Ctx-Neur	NA	NA	0.0000	0.0000	NA	NA	0.1210	0.1987	NA	0.0380	0.5219	0.4072
	Mes-Prog	NA	NA	NA	0.0122	NA	NA	NA	0.2519	NA	0.0413	0.3313	0.1624
	pRPC	NA	NA	0.0000	0.0056	0.2895	0.3127	0.2084	0.0000	0.0411	0.0000	0.1945	NA
	REP	NA	NA	0.0000	0.0000	0.2098	0.0000	0.1852	0.1950	0.0337	0.0000	0.1778	0.3450
	RPE	NA	NA	0.0028	0.0000	0.3555	0.4220	0.1481	0.1124	0.0000	0.0000	0.1985	0.0000
	subC-Neur	NA	NA	0.0000	0.0000	NA	0.0000	0.2684	0.1355	0.0278	NA	NA	NA

Appendix Table S5. Mean expression of perturbation targets

ID	Description	Genes	q-value	Module
GO:0015980	energy derivation by oxidation of organic compounds	61/338	1.23E-40	1
GO:0045333	cellular respiration	60/338	4.05E-48	1
GO:0009060	aerobic respiration	57/338	7.62E-50	1
GO:0006119	oxidative phosphorylation	52/338	7.62E-50	1
GO:0019693	ribose phosphate metabolic process	48/338	1.25E-20	1
GO:0042692	muscle cell differentiation	19/285	3.08E-02	2
GO:0051146	striated muscle cell differentiation	15/285	3.98E-02	2
GO:1990542	mitochondrial transmembrane transport	9/285	3.08E-02	2
GO:0086064	cell communication by electrical coupling involved in cardiac conduction	5/285	3.46E-02	2
GO:0042074	cell migration involved in gastrulation	4/285	3.98E-02	2
GO:0050808	synapse organization	19/197	1.94E-04	16
GO:0050804	modulation of chemical synaptic transmission	19/197	3.02E-04	16
GO:0099177	regulation of trans-synaptic signaling	19/197	3.02E-04	16
GO:0098742	cell-cell adhesion via plasma-membrane adhesion molecules	17/197	1.71E-05	16
GO:0030198	extracellular matrix organization	16/197	1.13E-04	16
GO:0007264	small GTPase mediated signal transduction	15/187	3.35E-02	20
GO:0050804	modulation of chemical synaptic transmission	15/187	3.35E-02	20
GO:0099177	regulation of trans-synaptic signaling	15/187	3.35E-02	20
GO:0043410	positive regulation of MAPK cascade	15/187	3.35E-02	20
GO:0003018	vascular process in circulatory system	11/187	3.35E-02	20
GO:0030198	extracellular matrix organization	14/166	7.94E-04	30
GO:0043062	extracellular structure organization	14/166	7.94E-04	30
GO:0045229	external encapsulating structure organization	14/166	7.94E-04	30
GO:0043217	myelin maintenance	4/166	1.01E-02	30
GO:0007015	actin filament organization	12/149	3.43E-02	41
GO:0051607	defense response to virus	11/149	1.08E-02	41
GO:0140546	defense response to symbiont	11/149	1.08E-02	41
GO:0034341	response to type II interferon	10/149	3.87E-04	41
GO:0032963	collagen metabolic process	7/149	1.08E-02	41
GO:0015980	energy derivation by oxidation of organic compounds	30/111	1.79E-24	59
GO:0045333	cellular respiration	29/111	8.08E-27	59
GO:0009060	aerobic respiration	27/111	8.65E-27	59
GO:0006119	oxidative phosphorylation	23/111	5.26E-24	59
GO:0022900	electron transport chain	22/111	5.90E-21	59
GO:0060562	epithelial tube morphogenesis	9/95	2.62E-02	62
GO:0001763	morphogenesis of a branching structure	7/95	2.62E-02	62
GO:0007584	response to nutrient	6/95	3.41E-02	62
GO:0048565	digestive tract development	6/95	2.62E-02	62
GO:0060576	intestinal epithelial cell development	3/95	2.62E-02	62
GO:0006936	muscle contraction	13/108	5.09E-05	64
GO:0003012	muscle system process	13/108	5.28E-04	64
GO:0042692	muscle cell differentiation	12/108	8.88E-04	64
GO:0031032	actomyosin structure organization	10/108	8.06E-05	64
GO:0055001	muscle cell development	10/108	6.04E-05	64
GO:0060537	muscle tissue development	10/108	1.08E-02	64
GO:0006887	exocytosis	8/65	3.43E-02	72
GO:0001505	regulation of neurotransmitter levels	6/65	3.66E-02	72
GO:0048278	vesicle docking	4/65	3.66E-02	72

Appendix Table S6. Pathway and gene ontology enrichment analyses of gene modules

Top 5 Gene Ontology Biological Processes significantly enriched in gene modules described in Figure EV5J.

Cell line	Chr. #	Base start	Base end	CNV length	Copy #
wild type	1	24945676	25968690	1023014	2
SMAD2		24945676	25968690	1023014	2
B2M		24945676	25968690	1023014	2
wild type	2	192753035	198143381	5390346	2
SMAD2		192753035	198143381	5390346	2
B2M		192753035	198143381	5390346	2
wild type	6	26362643	28433720	2071077	2
SMAD2		26362643	28433720	2071077	2
B2M		26362643	28433720	2071077	2
wild type	7	72044658	72358150	313492	3
SMAD2		72003201	72708505	705304	3
B2M		72083747	72731769	648022	3
wild type	7	69297662	70156217	858555	1
SMAD2		69297662	70158495	860833	1
B2M		69297662	70156217	858555	1
wild type	8	87824751	89855463	2030712	2
SMAD2		87824751	89855463	2030712	2
B2M		87824751	89855463	2030712	2
SMAD2	8	170692	30427531	30256839	1*
wild type	12	39184041	41724001	2539960	2
SMAD2		39184041	41724001	2539960	2
B2M		39132428	41724001	2591573	2
SMAD2	12	190980	32559693	32368713	3*
B2M	20	29625934	30869926	1243992	3*
wild type	Y	6630931	9170545	2539614	0
SMAD2		6190214	9170545	2980331	0
B2M		6190214	9170545	2980331	0

Appendix Table S7. Copy number variation analysis

CNVs identified in wild type WTC-11 and homozygous edited *SMAD2* or *B2M* iPS2-seq genome-edited hiPSCs. An asterisk indicates cell line unique CNVs.

Appendix Protocols Overview

iPS-optimized inducible Postranscriptional Silencing deconvoluted by single cell sequencing (iPS2-seq) enables robust phenotype-agnostic screens in human induced pluripotent stem cells (hiPSCs) and their derivatives. The method is based on loss-of-function perturbations that are mRNA-depleting (bypassing genotoxicity), clonally traced (controlling genetic and epigenetic variability), single cell aware (accounting for asynchronous and heterogeneous differentiation), isogenic engineered (preventing silencing), inducible and reversible (enabling stage-specific studies and robust control-treatment paired analyses). iPS2-seq is compatible with both commercial microfluidics and homebrew split-pool scRNA-seq protocols, enabling screens with different scales, costs, and input materials. A dedicated bioinformatics pipeline, *catcheR*, supports experimental design and data analysis. The following Supplementary Protocols cover the different procedures of a typical iPS2-seq experiment:

- Appendix Protocol 1. Generation of iPS2-seq plasmids and hiPSCs
- Appendix Protocol 2. iPS2-sci-seq library preparation
- Appendix Protocol 3. iPS2-10X-seq library preparation
- Appendix Protocol 4. iPS2-seq design and analysis with *catcheR*

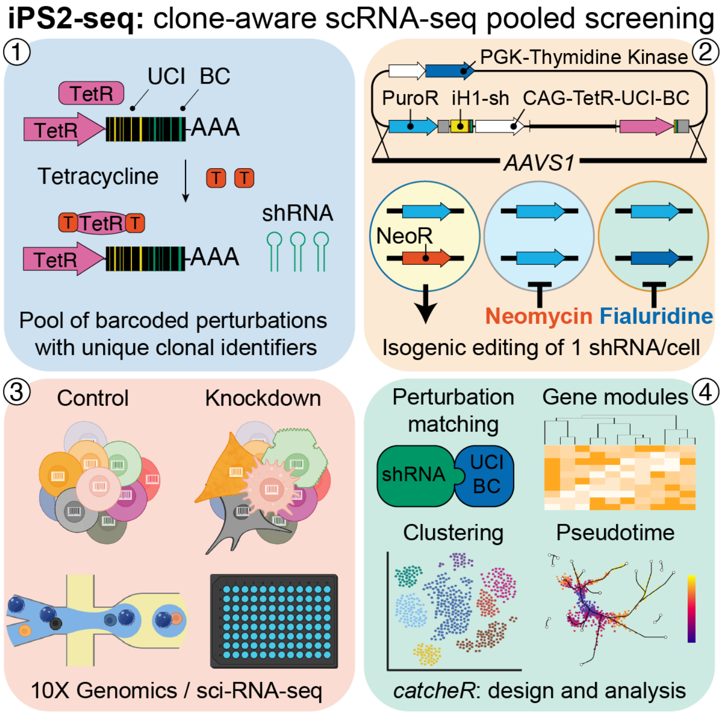

Appendix Protocol 1

Generation of iPS2-seq plasmids and hiPSCs

The iPS2-seq pipeline starts with a two-step cloning procedure for a barcoded pool of shRNAs into an *AAVS1* locus targeting plasmid to generate all-in-one tet inducible cassette (Figure EV1A).

This begins with designing single stranded DNA (ssDNA) oligonucleotides each carrying an shRNA with a polythymidine (pT) Pol III terminator sequence, a uniquely matched barcode (BC), and a random sequence serving as a unique clonal identifier (UCI). The shRNA and UCI-BC sequences are separated by a short multicloning site (SalI, SwaI, AscI) that is needed at a later stage, and are flanked by short regions of homology to the destination vector to enable Gibson assembly. The oligos are synthesized, pooled, and converted to double stranded DNA (dsDNA) through a single round of isothermal primer extension from the 3' end using a mesophilic DNA polymerase with strong strand displacement activity. This is essential to overcome the secondary structure of shRNAs in the oligos and minimize representation biases due to differential hairpin strength (Appendix Figures S1A-S1C).

dsDNA fragments are then cloned through Gibson assembly into an *AAVS1* targeting plasmid downstream of an inducible H1 Pol III promoter and upstream of a pA signal. The vector contains a puromycin (puro) resistance gene trap and is based on our previously reported plasmid (Bertero et al. 2016), but was modified to remove repetitive sequences that led to recombination errors in the next cloning step (Figures S1D–S1G). The plasmid also includes a novel thymidine kinase (TK) cassette outside of the *AAVS1* homology arms. This allows subsequent negative selection of cells carrying random, off-target integrations of the whole plasmid (Appendix Figure S1D), as they become sensitive to fialuridine (FIAU, a thymidine analog preferable to ganciclovir (Yusa 2013)).

The resulting intermediate plasmids are then modified in pool to insert a CAG-OPTtetR cDNA cassette between the shRNA and UCI-BC sequence (Figure EV1A). This second cloning step reconstitutes the tet-ON system while marking the 3' UTR of the OPTtetR with the UCI-BC sequence matched to a given shRNA. This is performed *via* restriction digestion and ligation, as the CAG promoter is too GC-rich for efficient and reliable PCR amplification. An optional strategy built into the system allows depletion of the intermediate plasmid from the final population through restriction digestion with SwaI.

The final plasmids are utilized to engineer pools of hPSCs enriched for clones expressing single inducible shRNAs. Plasmids are transfected alongside vectors encoding for obligate heterodimer zinc finger nucleases (ZFNs) (Bertero et al. 2016) to induce double-strand breaks specifically at the *AAVS1* locus and facilitate homology-directed repair (HDR) of the inducible barcoded shRNA cassette. A second *AAVS1* targeting plasmid is also co-delivered: this is a filler vector that carries no relevant cargo but has a neomycin (neo) resistance gene trap that allows enrichment of cells that integrated an shRNA on one *AAVS1* allele and the filler sequence on the other allele; these biallelic edited cells are co-selected with puro and neo. Additional negative selection with FIAU kills clones that carry randomly integrated shRNAs. Collectively, this genome editing strategy is designed to reduce the frequency of double shRNA integrations and maximise the fraction of cells expressing a single shRNA from the *AAVS1* locus.

Of note, the first cloning step can result in a low rate of swap between the shRNA and its expected BC based on oligonucleotide design and synthesis. This issue likely arises from DNA polymerase template switching (Potapov & Ong 2017) during dsDNA synthesis and/or Gibson assembly. The problem can be efficiently countered by next generation sequencing (NGS) analysis of Illumina-compatible libraries spanning the shRNA and UCI-BC region from the intermediate plasmid pool: this enables re-assignment of swapped shRNAs to the observed barcode, leveraging the fact that each plasmid also carries a UCI (also essential for tracking hPSC clones downstream). Similar NGS-based quality controls can be implemented to verify the diversity of the final plasmid pool in both bacteria and genome edited iPSCs.

In summary, this Supplemental Protocol describes the following procedures:

1. Plan the screening
2. Design shRNA oligonucleotides
3. Pooled cloning step 1: obtain the intermediate plasmid pool
4. Pooled cloning step 2: obtain the final plasmid pool
5. OPTIONAL: prepare NGS libraries for quality control of plasmid pools
6. Genome editing: obtain hPSC clonal pools

Plan the screening

Before initiating a screening experiment, it is essential to define three key elements: screening objectives, available budget, and overall feasibility. Effective screening design should begin with:

1. Target gene selection — Define the genes to perturb and decide how many shRNAs per gene will be used. We recommend a minimum of 3 shRNAs per gene, and ideally 5, to buffer against off-target effects or poorly performing constructs. Include at least 10% non-targeting control shRNAs.
2. Cell editing scale — Estimate the number of cells to genome-edit in order to achieve robust clonal representation for downstream iPS2-10X-seq analysis. An example calculation is provided in Figure SP1.1, accounting for both bacterial clone yield and the number of hiPSC clones required for sequencing.
3. Sequencing depth — Determine how many cells need to be profiled to capture biological variability and achieve statistical power. Depending on experimental goals and budget, controls may include non-targeting shRNAs (e.g., *B2M*, SCR), tet-untreated cells, or both. We strongly recommend including tet-untreated cells to control for clonal variability.

The flow chart below summarizes key parameters for optimizing molecular cloning, genome editing, and sequencing efficiency, followed by a worked example: a screen targeting 18 genes with 5 shRNAs per gene plus 10% control constructs.

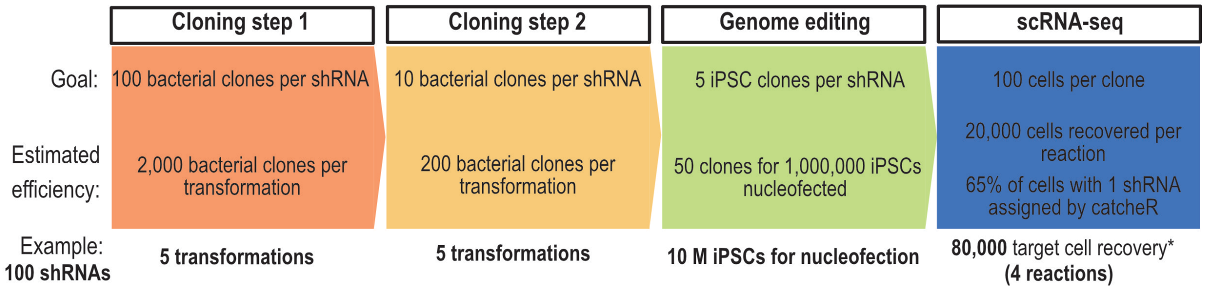

Figure SP1.1. Screening design example
Flow chart illustrating how to estimate the number of cells required for sufficient iPS2-seq coverage, accounting for dropout rates from cloning to transfection and single-cell sequencing. The top section defines the screening goal and provides an example estimation based on our protocol; the lower section shows a worked example with 100 shRNAs. scRNA-seq calculations are based the maximum loading capacity per reaction for either the Chromium Next GEM Single Cell 3' HT GEM Kit v3.1 or the Chromium GEM-X Universal 3' v4 Kit.

Design shRNA oligonucleotides

ssDNA oligonucleotides for iPS2-seq can be designed using *catcheR_design* as described in Appendix Protocol 4 - Oligonucleotides design. This function automatically performs the following steps, reducing the chance of manual errors when designing multiple oligos.

1. Identify validated shRNAs or design appropriate sequences. In our lab we use the Broad Institute TRC library, available at the GPP Web Portal. When using this resource take the ~58 bp forward oligos and remove the sequences required for cloning in pLKO vectors (typically the first 4 nucleotides and the last 6 nucleotides)

2. If an shRNA does not start with A or a G, add an initial G (required for efficient transcription by the H1 Pol III promoter)

3. Construct the cloning oligos by adding the following sequences to each shRNA, in order:

   (a) At the 5', the Gibson homology region 5'-AGTTCCCTATCAGTGATAGAGATCCC-3'
   (b) At the 3', the polythymidine (pT) Pol III terminator sequence 5'-TTTTTTT-3'
   (c) At the 3', SalI, SwaI, and AscI restriction sites 5'-GTCGACATTTAAATGGCGCGCC-3'
   (d) At the 3', a random sequence (UCI; i.e., 5'-NNNNNN-3')
   (e) At the 3', an shRNA-specific barcode (BC; i.e., 5'-CAGTTCCA-3')
   (f) At the 3', the Gibson homology region 5'-GTAGCTCGCTGATCAGC-3'

   The resulting oligos should look like Figure SP1.2

   Note: These sequences are passed as arguments to *catcheR_design*. The recommended minimum length for UCIs and BCs is 6 and 8 bp, respectively. Tools such as BARCOSEL can be used to generate and select BCs with optimal nucleotide distribution

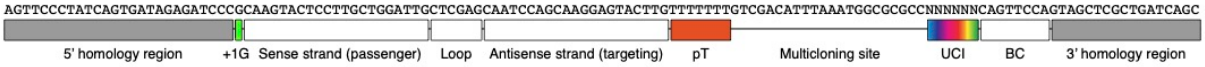

Figure SP1.2. Example of iPS2-seq ssDNA oligo
Annotated SMAD2.1 shRNA sequence extracted from TRC ID:TRCN0000010477 and modified by *catcheR_design*.

4. Modify or replace any cloning oligo that contains BglII (5'-AGATCT-3') or MluI (5'-ACGCGT-3') restriction sites that would interfere with subsequent cloning steps

5. Synthesize oligos as high quality ssDNA. In our lab we use the oPools Oligo Pools service from Integrated DNA Technologies

Pooled cloning step 1: obtain the intermediate plasmid pool

This section outlines the pooled conversion of barcoded shRNA-containing ssDNA oligos into dsDNA by second-strand synthesis (SSS), and the insertion of such fragments in pAAV-Puro_siKD2.0.

1. Prepare the oligo pool

   (a) Spin down the lyophilized ssDNA and resuspend it at 50 µM in nuclease-free water
   (b) Dilute the 50 µM stock 1:10 in nuclease-free water to make a 5 µM working stock

2. Convert ssDNA to dsDNA using Bst3.0 DNA polymerase

   (a) Denature and snap cool the ssDNA oligo pool

      i. Mix the following for 8 reactions in a single tube on ice:

Appendix Table SP1.1. SSS reaction mix 1

Reagent	One reaction (µL)	Eight reactions (µL)	Final conc.
ssDNA oligo pool (5 µM)	5	40	0.5 µM
iPS2-seq_dsDNA_R primer (10 µM)	2.5	20	0.5 µM
dNTP solution mix (10 mM)	2	16	400 µM
Nuclease-free water	15.5	124	
Total volume	25	200	

      ii. Distribute 25 µL of the reaction mix into 8 PCR tubes

      iii. Place PCR tubes in a thermocycler and run the following cycling conditions:

Appendix Table SP1.2. Cycling conditions for ssDNA denaturation and snap cooling

Steps	Temperature	Time	Cycles
Denaturation	98 °C	5 min	1
Hold (rapid ramp down)	4 °C	Hold	

   (b) Perform SSS

      i. Mix the following for 8 reactions in a single tube on ice:

Appendix Table SP1.3. SSS reaction mix 2

Reagent	One reaction (µL)	Eight reactions (µL)	Final conc.
MgSO4 (100 mM)	1	8	2 mM
Isothermal amplification buffer II (10X)	5	40	1X
Bst 3.0 DNA polymerase (8 U/µL)	2	16	0.32 U/µL
Nuclease-free water	17	136	
Total volume	25	200	

      ii. Add 25 µL of this reaction mix to each of the 8 PCR tubes from step 2(a)iii

      iii. Place PCR tubes in a thermocycler and run the following cycling conditions:

Appendix Table SP1.4. Cycling conditions for SSS

Step	Temperature	Time	Cycles
Annealing	55 °C	5 min	1
Extension	72 °C	15 min	1
Inactivation	80 °C	5 min	1
Slow ramp down	75 °C	-0.1 °C/s	1
Hold	75°C	4 min	1
Slow ramp down	70 °C	-0.1 °C/s	1
Hold	70°C	4 min	1
Slow ramp down	10 °C	-0.1 °C/s	1
Hold	10°C	Hold	

   (c) Remove residual ssDNA

      i. Add 4 µL of exonuclease I (20 U/µL) to each PCR tube and pipette mix

      ii. Place PCR tubes in a thermocycler and run the following cycling conditions:

Appendix Table SP1.5. Cycling conditions for ssDNA removal

Step	Temperature	Time	Cycles
Digestion	37 °C	30 min	1
Inactivation	80 °C	20 min	1
Slow ramp down	75 °C	-0.1 °C/s	1
Hold	75°C	4 min	1
Slow ramp down	70 °C	-0.1 °C/s	1
Hold	70°C	4 min	1
Slow ramp down	10 °C	-0.1 °C/s	1
Hold	10 °C	Hold	

(d) Clean up and concentrate the dsDNA

i. Pool all 8 reactions into a single tube

ii. Column purify using QIAquick PCR Purification Kit, following its manual

iii. Elute in 30 µL elution buffer
Note: the dsDNA shRNA pool can be stored at -20 °C for at least one week

3. Prepare the plasmid backbone

(a) Prepare a digestion mix and an undigested control mix following the table below:

Appendix Table SP1.6. pAAV-Puro_siKD2.0 restriction digestion mix

Reagent	Digestion reaction (µL)	Undigested control (µL)	Final conc.
pAAV-Puro_siKD2.0 plasmid	Variable	Variable	5 µg
FastDigest Green Buffer (10X)	15	1.5	1X
FastDigest MluI	5	0	
FastDigest BglII	5	0	
FastAP (1 U/µL)	5	0.5	0.03 U/µL
Nuclease-free water	Top up to 150	Top up to 15	
Total volume	150	15	

(b) Incubate at 37 °C overnight (~16 h), followed by 10 min at 80 °C for heat inactivation

4. Perform electrophoretic separation of the insert (step 2(d)iii) and backbone (step 3b) using 2% and 0.8% (w/v) agarose gels in TBE, respectively. Cast gels including SYBR Safe DNA gel stain to visualize DNA, preferably using a blue light transilluminator (avoid UV light if possible). Figure SP1.3 shows exemplary electrophoreses

Notes:

(a) Use 50 bp and 1 kbp DNA ladders for the insert and backbone, respectively

(b) Run 10% of each sample in a separate well and use this to monitor the electrophoretic separation, so as to avoid unnecessary exposure to high-energy light of the gel extracted DNA (particularly important if using UV light by necessity)

(c) Use FastDigest Green Buffer (10X) as loading dye for the insert: commonly used DNA loading dyes containing bromophenol blue and/or xylene cyanol FF can interfere with visualization of the small shRNA pool fragment

(d) If a smear is present in the insert, continue electrophoresis until the 135 bp band is clearly separated

(e) Run 90% of the digested empty plasmid (~140 µL) in a single well as this will facilitate gel extraction: merge multiple wells using autoclave tape if needed

(f) Load the plasmid undigested control next to 10% of the digestion reaction and continue the electrophoretic separation until the 8715 bp backbone band is completely separated from the undigested control (this can take several hours: use a low voltage and/or change buffer to prevent gel overheating). Incomplete separation can result in contamination of undigested or partially digested plasmid, which can dramatically increase transformation background

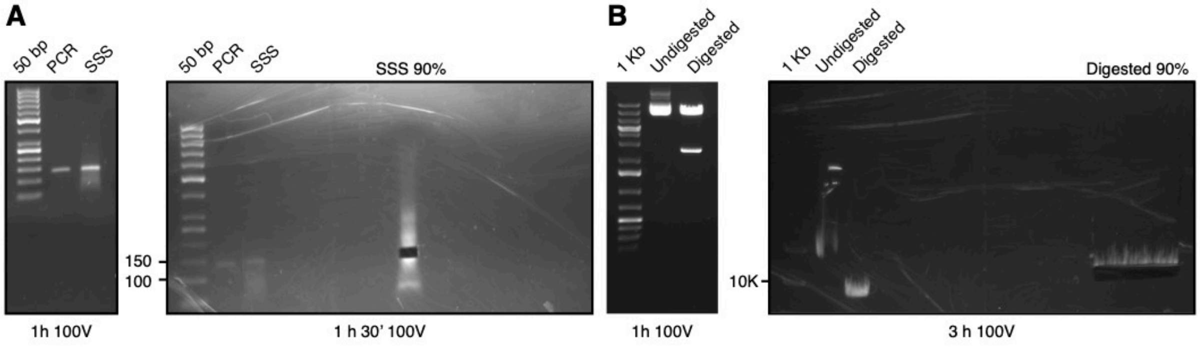

Figure SP1.3. Electrophoretic separation and gel extraction during cloning step 1
(A) Separation of shRNA pool insert (SSS); a PCR-amplified fragment indicates the expected molecular weight.
(B) Separation of digested plasmid backbone.

5. Use a clean scalpel to excise the DNA bands corresponding to the shRNA pool insert (135 bp) and plasmid backbone (~8.7 kbp) and place them in a microcentrifuge tube
6. Proceed with gel extraction using the QIAEX II Gel Extraction Kit following the manual and eluting in 20 µL EB; quantify the DNA concentration using NanoDrop
7. Setup the desired assembly alongside control reactions following the table below:

Appendix Table SP1.7. Pooled cloning step 1 assembly mix

Reagent	Desired assembly (µL)	Backbone-only control (µL)	No assembly control (µL)	Final conc.
Gel extracted insert (135 bp)	Variable	0	0	14.34 ng (172.4 fmol)
Gel extracted backbone (8715 bp)	Variable	Variable	Variable	50 ng (9.32 fmol)
NEBuilder HiFi DNA Assembly Master Mix (2X)	10	10	0	1X
Nuclease-free water	To 20	To 20	To 20	
Total volume	20	20	20	

(a) Incubate the reactions at 50 °C for 15 min, then transfer to ice
Note: avoid longer incubation times as they can increase background

8. Transform 2 µL of assembly or control reactions in 50 µL NEB 5-alpha competent *E. coli* (high-efficiency), following the manufacturer's instructions
9. Plate transformed bacteria
   (a) Pre-warm LB-agar plates containing 100 µg/mL ampicillin to 37 °C
   (b) To facilitate estimation of colony number and cloning efficiency, plate 10% of transformed bacteria on one dish, and the remaining 90% on a second dish
   (c) Incubate the plates at 37 °C overnight
10. Estimate the number of bacterial clones by counting the colonies in the plate containing 10% of the transformation of the desired assembly (>200 are to be expected)

Notes:

(a) the number of colonies in the matching backbone-only control and no assembly control plates should be at least 10-fold and 100-fold lower; it is advisable to repeat the procedure should a substantially higher background be observed

(b) bacterial plates can be stored at 4 °C for up to one week to allow for the subsequent optional quality controls

11. **OPTIONAL.** Confirm cloning efficiency by bacterial colony PCR

    (a) For each colony to be screened, aliquot in PCR plates a 15 µL PCR mix based on nuclease-free water and containing: iPS2-seq_step1QC_F Primer (200 nM), iPS2-seq_QC_R Primer (200 nM), dNTPs (200 µM), MgCl2 (2 mM), Colorless GoTaq Flexi buffer (1X), and GoTaq DNA polymerase (0.025 U/µL)

    (b) Pick an individual bacterial colony from the 10% plate, mix it by pipetting in 10 µL nuclease-free water aliquoted in a PCR plate, and transfer 5 µL of the suspension to the PCR mix for a final volume of 20 µL. The remaining 5 µL can be transferred to a PCR plate containing LB with 100 µg/mL ampicillin and stored at 4 °C for later use. Repeat for all colonies

    (c) Place the PCR plate in a thermocycler and run following cycling conditions: (1) 95 °C for 5 min; (2) 95 °C for 30 sec; (3) 60 °C for 30 sec; (4) 72 °C for 1 min; (5) go to step 2 for 34 times; (6) 72 °C for 2 min; (7) hold at 10 °C

    (d) Aliquot 5 µL of each colony PCR in a new plate, add 1 µL of 6X DNA loading dye, and perform an electrophoretic run on a 1.5% (w/v) agarose gel in TBE

    Note: clones with the correct insert deliver a PCR product of 543 bp (Figure EV1B)

12. **OPTIONAL.** Confirm cloning accuracy by Sanger sequencing

    (a) Aliquot 5 µL of each colony PCR in a new plate and add 1.5 µL of a mastermix containing 0.5 µL of exonuclease 1 (20 U/µL) and 1 µL of shrimp alkaline phosphatase (1 U/µL)

    (b) Incubate the reaction at 37 °C for 30 min, followed by heat inactivation for 15 min at 80 °C

    (c) Add 5 µL of iPS2-seq_QC_R primer (10 µM) to each well, and run Sanger sequencing

    Note: alignment of each electropherogram to the common sequence 5'-TTTTTTTGTCGAC-ATTTAAATGGCGCGCCNNNNNNNNNNNNNNNGTAGCTCGCTGATCAGC-3' simplifies extraction of the BC (last 8 Ns); the electropherogram can then be aligned to the expected oligo based on the BC, to verify the correct sequence of the associated shRNA

13. Collect all bacterial colonies from the 90% plate by washing and scraping the surface of LB-agar plates with 5 mL of LB broth with 100 µg/mL ampicillin

14. Inoculate the bacterial suspension in a clean Erlenmeyer flask with 45 mL LB broth containing 100 µg/mL ampicillin, and grow the bacterial cultures at 37 °C at 225 rpm for 16 h **Note**: inoculate bacteria in a sterile environment and use a sterile flask to minimize the chances of contamination. Do not grow bacteria for more than 16 h

15. Isolate the intermediate plasmid pool from bacterial cultures using the QIAGEN plasmid midiprep kit following the manual, and quantify the DNA concentration using NanoDrop

Pooled cloning step 2: obtain the final plasmid pool

This section outlines the generation of the final plasmid pool by re-inserting the CAG-OPTtetR into the intermediate plasmid pool.

1. Prepare the following restriction digestion reaction of the empty pAAV-Puro_siKD2.0 plasmid with SalI and MluI to extract the CAG-OPTtetR insert:

Appendix Table SP1.8. pAAV-Puro_siKD2.0 second restriction digestion mix

Reagent	Digestion reaction (µL)	Undigested control (µL)	Final conc.
pAAV-Puro_siKD2.0 plasmid	Variable	Variable	5 µg
FastDigest Green Buffer (10X)	10	1.5	1X
FastDigest SalI	5	0	
FastDigest MluI	5	0	
Nuclease-free water	To 100	To 15	
Total volume	100	15	

2. Prepare the following restriction digestion reaction of the intermediate plasmid pool from step 15 of the previous section with SalI and AscI to obtain the backbone:

Appendix Table SP1.9. Intermediate plasmid pool restriction digestion mix

Reagent	Digestion reaction (µL)	Undigested control (µL)	Final conc.
Intermediate iPS2-seq plasmid	Variable	Variable	5 µg
FastDigest Green Buffer (10X)	15	1.5	1X
FastDigest SalI	5	0	
FastDigest SgsI (AscI)	5	0	
FastAP (1 U/µL)	5	0.5	0.03 U/µL
Nuclease-free water	To 150	To 15	
Total volume	150	15	

3. Incubate at 37 °C overnight (~16 h), followed by 10 min at 80 °C for heat inactivation

4. Perform electrophoretic separation of all fragments using 0.8% (w/v) agarose gel in TBE supplemented with SYBR Safe. Figure SP1.4 shows an exemplary electrophoresis

   Note: follow the same general recommendations described for step 4 of the previous section, paying particular attention to separating the backbone from undigested plasmid

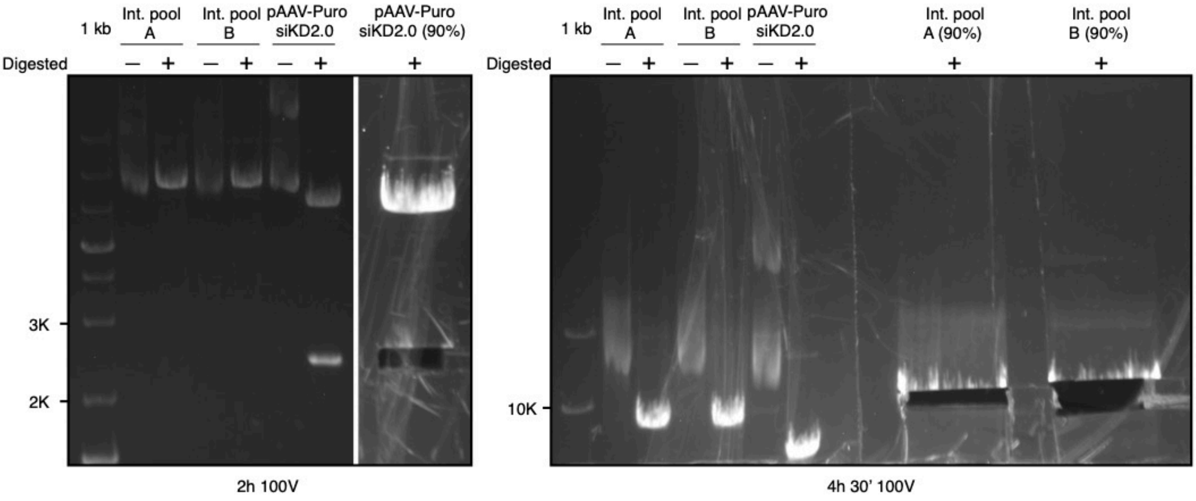

Figure SP1.4. Electrophoretic separation and gel extraction during cloning step 2 Separation of the CAG-OPTtetR insert and two digested intermediate plasmid pool backbones.

5. Use a clean scalpel to excise the ~8.8 kbp backbone from the digested intermediate plasmid pool and the ~2.4 kbp CAG-OPTtetR insert from pAAV-Puro_siKD2.0

6. Proceed with gel extraction using the QIAEX II Gel Extraction Kit following the manual and eluting in 20 µL EB; quantify the DNA concentrations using NanoDrop

7. Set up desired ligation alongside a control reaction following the table below:

Appendix Table SP1.10. Pooled cloning step 2 ligation mix

Reagent	Ligation (µL)	Backbone only control (µL)	Final conc.
Gel extracted CAG-OPTtetR fragment (2392 bp)	Variable	0	81.59 ng (55.38 fmol)
Gel extracted intermediate iPS2-seq pool (8795 bp)	Variable	Variable	100 ng (18.46 fmol)
Rapid Ligation Buffer (5X)	4	4	1X
T4 DNA Ligase (5 U/µL)	1	1	0.25 U/µL
Nuclease-free water	To 20	To 20	
Total volume	20	20	

   (a) Incubate the reaction at RT for 1 h

8. Transform 5 µL of ligation and control reaction in 50 µL NEB 5-alpha competent *E. coli* (high-efficiency), following the manufacturer's instructions

9. Plate and grow overnight transformed bacteria according to the general recommendations described for step 9 of the previous section, particularly regarding splitting the bacteria on two plates (10% and 90%)

10. Estimate the number of bacterial clones by counting the colonies in the plate containing 10% of the transformation of the desired assembly (>20 are to be expected)

    Notes:

    (a) the number of colonies in the backbone-only control should be at least 5-fold lower

    (b) bacterial plates can be stored at 4 °C for up to one week

11. **OPTIONAL**. Confirm cloning efficiency by bacterial colony PCR

    (a) Perform colony PCRs and electrophoresis as described for step 11 of the previous section except using primers iPS2-seq_step2QC_F and iPS2-seq_QC_R

    Notes:

    i. clones with the correct insert deliver a PCR product of 312 bp; intermediate plasmids are not amplified, while the parental pAAV-Puro_siKD2.0 plasmid gives a 295 bp band (Figure EV1C)

    ii. Sanger sequencing can also be performed as described for step 12 of the previous section, to confirm the distribution of shRNA BCs; however, at this final stage the shRNA and barcode cannot be co-amplified

12. Collect all bacterial colonies and inoculate them as described for steps 13-14 of the previous section. Be extra careful to avoid contaminations at this stage

13. Isolate the final plasmid pool from bacterial cultures using the QIAGEN plasmid midiprep kit with endotoxin-free buffers, and quantify the DNA concentration using Qubit

14. Perform a diagnostic restriction digestion using EcoRI, MluI, and SwaI: the molecular weight of expected bands is 5669 bp and 5518 bp

    Note: additional bands at 2002 bp or 4343 bp would indicate contamination with intermediate plasmids or pAAV-Puro_siKD2.0, respectively. These contaminants can be removed by digestion with SwaI or BglII and MluI, respectively, followed by re-transformation

OPTIONAL: prepare NGS libraries for quality control of plasmid pools

This section outlines the optional generation of Illumina-compatible next generation sequencing (NGS) libraries to determine the composition of intermediate and final plasmid pools (Figure SP1.5A, Figure SP1.6A). Data analysis relies on *catcheR_step1QC* and *catcheR_step2QC*, respectively, as described in Appendix Protocol 4 - Pooled cloning step 1 QC and Pooled cloning step 2 and hiPSC genome editing QC.

Intermediate plasmid pool QC

1. PCR #1: amplify the shRNA and UCI-BC region from the intermediate plasmid pool while adding the TruSeq read 2 adapter, a diversity index (DI, a random 12 bp sequence that ensures adequate clustering during NGS and can be leveraged to partially demultiplex reads to eliminate duplicates from the subsequent PCR), and partial TruSeq read 1 adapter

    (a) Prepare a PCR reaction according to the following calculations:

Appendix Table SP1.11. Intermediate plasmid pool QC, PCR #1 mix

Reagent	Volume (µL)	Final conc.
Intermediate iPS2-seq plasmid pool	Variable	150 ng
ThermoPol Reaction Buffer (10X)	2.5	1X
dNTP solution mix (10 mM)	0.5	200 µM
iPS2-seq_step1NGS_F Primer (10 µM)	1.25	0.5 µM
iPS2-seq_NGS_R Primer (10 µM)	1.25	0.5 µM
MgSO4 (100 mM)	1	4 mM
Deep Vent DNA Polymerase (2 U/µL)	0.25	0.02 U/µL
Nuclease-free water	To 25	
Total volume	25	

    (b) Place the PCR tube in a thermocycler and run the following cycling conditions:

Appendix Table SP1.12. Cycling conditions for PCR #1, intermediate plasmid pool QC

Steps	Temperature	Time	Cycles
Initial denaturation	95°C	2 min	1
Denaturation	95°C	30 sec	3 cycles
Annealing	54 - 59 °C (increase by 2.5 °C each cycle)	15 sec	
Extension	72 °C	30 sec	
Denaturation	95 °C	30 sec	9 cycles
Annealing	63 °C	15 sec	
Extension	72 °C	30 sec	
Final extension	72 °C	5 min	1
Hold (rapid ramp down)	4 °C	Hold	

    Note: a ”touch-up” strategy minimizes the annealing of shRNA hairpins during the second part of the reaction: once a sufficient number of molecules have integrated the TruSeq adapters, they anneal to the primers at higher temperatures

    (c) Add 25 µL of nuclease-free water to the reaction and perform a double-sided size selection using 0.6X & 1.2X volumes of SPRIselect beads, following the manufacturer’s instructions and eluting in 20 µL of elution buffer

    (d) Run 2 µL of the undiluted PCR product on a TapeStation High Sensitivity D1000 Screen Tape assay, following the manual. Expect a single peak of ~203 bp. An exemplary result is reported in (Figure SP1.5B)

    (e) Quantify the DNA concentration in the range 100-400 bp, and dilute it to 1 ng/µL

2. PCR #2: prepare dual-indexed Illumina TruSeq sequencing libraries

    (a) Prepare a PCR reaction according to the following calculations:

Appendix Table SP1.13. Intermediate plasmid pool QC, PCR #2 mix

Reagent	Volume (µL)	Final conc.
PCR #1 product (step 1e; 1 ng/µL)	2	2 ng
ThermoPol Reaction Buffer (10X)	2	1X
dNTP solution mix (10 mM)	0.4	200 µM
Dual Index primers Kit TT Set A	4	
MgSO4 (100 mM)	0.8	4 mM
Deep Vent DNA Polymerase (2 U/µL)	0.2	0.02 U/µL
Nuclease-free water	10.6	
Total volume	20	

    (b) Place the PCR tube in a thermocycler and run the following cycling conditions:

Appendix Table SP1.14. Cycling conditions for PCR #2, intermediate plasmid pool QC

Steps	Temperature	Time	Cycles
Initial denaturation	95°C	2 min	1
Denaturation	95°C	30 sec	
Annealing	45 - 47 °C (increase by 2.5 °C each cycle)	15 sec	3 cycles
Extension	72 °C	45 sec	
Denaturation	95°C	30 sec	
Annealing	63 °C	15 sec	9 cycles
Extension	72 °C	30 sec	
Final extension	72 °C	5 min	1
Hold (rapid ramp down)	4 °C	Hold	

    (c) Add 30 µL of nuclease-free water to the reaction and perform a double-sided size selection using 0.65X & 0.85X volumes of SPRIselect beads, following the manufacturer's instructions and eluting in 20 µL of elution buffer

    (d) Run 2 µL of the undiluted PCR product on a TapeStation High Sensitivity D1000 Screen Tape assay, following the manual. Expect a single peak of ~287 bp

3. Perform NGS using NextSeq 1000/2000 and 100 cycles reagents, with the following settings: read 1 - 118 cycles; index 1 - 10 cycles; index 2 - 10 cycles (>100,000 reads/shRNA)

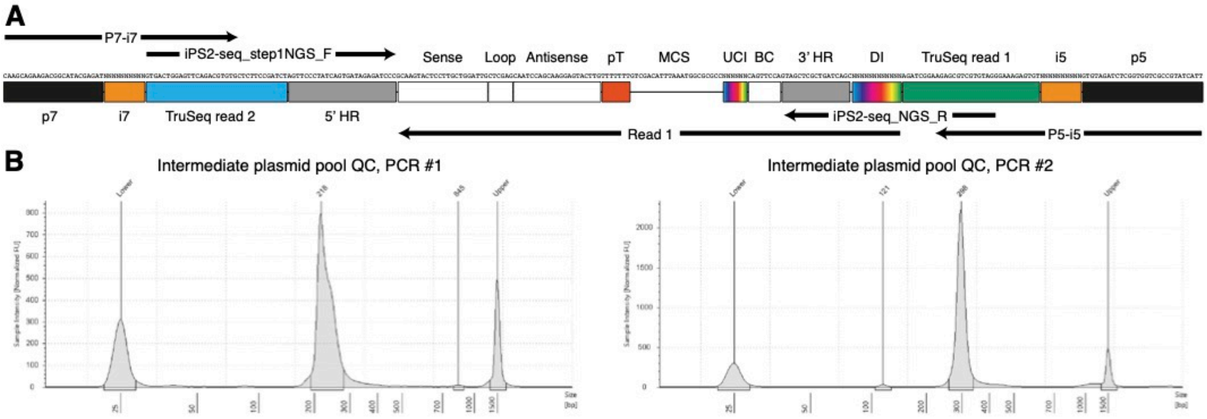

Figure SP1.5. NGS library generation from intermediate plasmid pools
(A) Library structure and primer sequences used for PCR #1 and PCR #2; read 1 is also indicated.
(B) Exemplary TapeStation High Sensitivity D1000 Screen Tape assays of size-selected DNA from PCRs #1 and #2.

Final plasmid pool QC

1. PCR #1: amplify UCI-BCs from the final plasmid pool, adding TruSeq adapters and DIs

Appendix Table SP1.15.
Final plasmid pool QC, PCR #1 mix

Reagent	Volume (µL)	Final conc.
Final iPS2-seq plasmid pool	Variable	25 ng
Q5 Reaction Buffer (5X)	5	1X
dNTP solution mix (10 mM)	0.5	200 µM
iPS2-seq_step2NGS_Primer (10 µM)	1.25	0.5 µM
iPS2-seq_NGS_R Primer (10 µM)	1.25	0.5 µM
Q5 Hot Start High-Fidelity DNA Polymerase (2 U/µL)	0.25	0.02 U/µL
Nuclease-free water	To 25	
Total volume	25	

Appendix Table SP1.16.
Cycling conditions for PCR #1

Steps	Temperature	Time	Cycles
Initial Denaturation	98 °C	30 sec	1
Denaturation	98 °C	10 sec	9 cycles
Annealing	67 °C	30 sec	
Extension	72 °C	20 sec	
Final extension	72°C	2 min	1
Hold (rapid ramp down)	4 °C	Hold	

   (a) Purify, quantify, and dilute the PCR product following steps 1c, 1d, and 1e of the previous section, except for using 0.6X & 1.5X volumes of SPRIselect beads for double-sided size selection. Expect a single peak of ~144 bp (Figure SP1.6B)

2. PCR #2: prepare dual-indexed Illumina TruSeq sequencing libraries

Appendix Table SP1.17.
Final plasmid pool QC, PCR #2 mix

Reagent	Volume (µL)	Final conc.
PCR 1 product (step 1a; 1 ng/µL)	1	1 ng
Dual Index primers Kit TT Set A	4	
NEBNext high fidelity 2X PCR master mix	10	1X
Nuclease-free water	5	
Total volume	20	

Appendix Table SP1.18.
Cycling conditions for PCR #2

Steps	Temperature	Time	Cycles
Initial Denaturation	98 °C	30 sec	1
Denaturation	98 °C	10 sec	8 cycles
Annealing	58 °C	20 sec	
Extension	72 °C	20 sec	
Final extension	72 °C	30 sec	1
Hold (rapid ramp down)	4 °C	Hold	

   (a) Purify and quantify the PCR product following steps 2c and 2d of the previous section, except for using 0.65X & 1.5X volumes of SPRIselect beads for double-sided size selection. Expect a single peak of ~228 bp (Figure SP1.6B)

3. Perform NGS using NextSeq 1000/2000 and 50 cycles reagents, with the following settings: read 1 - 68 cycles; index 1 - 10 cycles; index 2 - 10 cycles (>10,000 reads/shRNA)

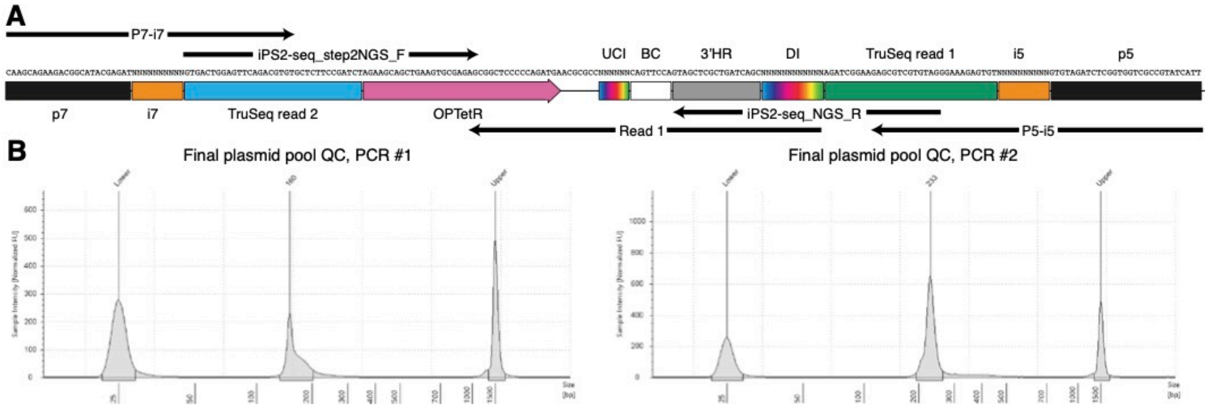

Figure SP1.6. NGS library generation from final plasmid pools
(A) Library structure and primer sequences used for PCR #1 and PCR #2; read 1 is also indicated.
(B) Exemplary TapeStation High Sensitivity D1000 Screen Tape assays of size-selected DNA from PCRs #1 and #2.

Genome editing: obtain hPSC clonal pools

This section outlines the generation of hPSCs genome edited at the *AAVS1* locus with the final iPS2-seq plasmid pool, and the optional genotyping of individual clones as quality control (Figure SP1.1).

1. Preparatory steps
   (a) Determine the number of nucleofections required to obtain the total target number of hPSC clones, based on the number of shRNAs, the target number of clones per shRNA, and the efficiency of genome editing
   Notes:
      i. The protocol describes plasmid delivery by nucleofection as this is most efficient, but if the necessary instrumentation is not available plasmids can instead be transfected as described in the Methods
      ii. While our standard protocol uses ZFNs to target the *AAVS1* locus, alternative programmable nucleases such as TALENs and CRISPR/Cas9 can also be used. We opted for ZFNs because we previously demonstrated >95% on-target integration efficiency in hPSCs. That said, our targeting vector is fully compatible with other systems, including TALENs and widely used CRISPR/Cas9 reagents (e.g., Addgene #59025, #59026, #129726). Besides plasmid delivery, in side-by-side comparisons, we observed comparable integration efficiencies using 200,000 cells per nucleocuvette strip co-electroporated with the iPS2-seq vector and either 1 µg ZFN plasmids or Cas9 RNPs (1 µg Cas9 protein + 200 ng sgRNA)
      iii. Perform a pilot genome editing experiment to empirically determine the efficiency of genome editing in an hPSC line used for iPS2-seq for the first time
   (b) Prepare the following plasmids using the QIAGEN plasmid midiprep kit with endotoxin-free buffers, diluting them to 1 µg/µL (2.5 µg/nucleofection):
      i. Pool of pAAV-Puro_siKD2.0 with the barcoded shRNAs (step 13 from Pooled cloning step 2: obtain the final plasmid pool)
      ii. pAAV-Neo_CAG
      iii. pZFN-AAVS1_ELD
      iv. pZFN-AAVS1_KKR
   (c) Prepare Geltrex-coated 24-well culture dishes (1 well per nucleofection)
   (d) Culture hPSCs in Essential 8 at ~40% confluency (1 x 10^6/nucleofection)

2. 16 h before nucleofection, refresh the hPSC culture media to Essential 8 supplemented with 2 µM Thiazovivin. Do not add antibiotics until step 11

3. Prepare hPSC Essential 8 supplemented with CEPT [50 nM Chroman 1; 5 µM Emricasan; 0.1% Polyamine Supplement; 0.7 µM trans-ISRIB] (1.5 mL/nucleofection)

4. Remove the coating solution from the pre-coated culture dish, add 1 mL of Essential 8 with CEPT per well, and place the dish in the incubator to acclimatize to 37 °C

5. Prepare a mix of equal mass of the four plasmids from step 1b (10 µg/transfection) and nucleofection buffers (18 µL P3 Supplement & 82 µL Nucleofector Solution per reaction)

6. Obtain a single cell suspension by treating hPSCs with StemPro Accutase, following the manufacturer's instructions, aliquot 1 x 10^6 hPSCs/nucleofection in a conical tube, and pellet the cells at 100 g for 5 min

7. Remove the supernatant, gently resuspend the pellet with the nucleofection mix from step 5, transfer 110 µL/nucleofection in a Nucleocuvette Vessel (avoiding bubbles), place the cuvette into the 4D-Nucleofector System and run program CA137

8. Add 500 µL of Essential 8 with CEPT to each cuvette and use the suction pipette to gently transfer the cell suspension to the 24-well plate from step 4. Place in the incubator

9. 24 h post nucleofection, refresh media with Essential 8, and prepare Geltrex-coated 100 mm dishes (1 per nucleofection)

10. 48 h post nucleofection, dissociate cells as small clumps with 0.5 mM EDTA in DPBS, and replate in Essential 8 with 2 µM Thiazovivin (using one 100 mm dish per well of 24-wp)

11. Refresh media daily with Essential 8. Antibiotics can be added from now on

12. When hPSCs are ~50% confluent (~5-6 days post nucleofection), perform dual positive drug selection for 4 days by supplementing Essential 8 with 0.5 µg/mL puromycin and 25 µg/mL Geneticin, refreshing media daily; include 2 µM Thiazovivin for the first 2 days

    Note: to optimize the procedure for a specific hPSC line, perform a kill curve with 0.25-2 µg/mL puromycin and 12.5-100 µg/mL Geneticin to identify the minimal concentration of each drug that in combination, eliminates all unedited hPSCs within 72 h

13. When hPSCs colonies are ready for passaging (~1-2 mm in diameter), count the number of colonies (clones), dissociate cells as small clumps with 0.5 mM EDTA in DPBS, and replate in Essential 8 with 2 µM Thiazovivin at a density of ~1 clones per cm^2

14. From 24 h post passaging until 5 days post passaging, refresh media daily with Essential 8 supplemented with 200 nM fialuridine (FIAU) to perform the negative selection

15. hPSC clonal pools are now ready for screening experiments and/or cryopreservation

    Notes:

    (a) Minimize the number of passages before the induction of gene knockdown, to minimize the expansion of hPSC clones with a genetic/epigenetic growth advantage

    (b) To account for the possible emergence of hPSC clones with neuroectodermal bias ("iPSC-neuro" clones), ensure sufficient clonal representation per perturbation. iPS2-seq enables robust retrospective control for this source of variability; however, it may still be helpful to screen for expression of early neuroectodermal markers such as *ZIC1* or *UNC5D*. If a given pool contains an overrepresentation of iPSC-neuro clones, consider repeating the targeting or depleting biased clones using, for example, a UNC5D antibody for FACS or MACS

    (c) For hPSC growth and differentiation protocols involving media containing FBS and/or BSA, batch-test these reagents for the absence of tetracycline contamination (i.e., by generating a clonal line expressing a validated inducible shRNA, to confirm that shRNA expression is not leaky in the absence of exogenously added tetracycline (Bertero et al. 2016))

    (d) To induce knockdown, add 1 ng/mL tetracycline, refreshing the media daily or every 48 h, when compatible for the specific differentiation protocol

hiPSC pool QC

This procedure aims to determine clonal diversity and shRNA representation in the genome-edited hiPSC pool by generating Illumina-compatible NGS libraries of UCI-BCs that can be analyzed using *catcheR_step2QC* as described in Appendix Protocol 4 - Pooled cloning step 2 and hiPSC genome editing QC.

1. After hPSC passaging, collect the cells from a confluent 6-well plate, and pellet the cells at 100 g for 5 min

   Note: Cell pellets can be frozen at -20 °C if needed

2. Obtain purified genomic DNA (gDNA) of the genome-edited hiPSCs with the Monarch Spin gDNA Extraction Kit

3. Elute samples with 50-100 µL of elution buffer and assess DNA purity and quantity by NanoDrop and Qubit broad range dsDNA

4. Use 500 ng of gDNA per PCR reaction, performing as many reactions as needed to achieve coverage of at least 100-fold the estimated number of clones

   Note: One nanogram of human gDNA contains approximately 152 genome equivalents; therefore, 500 ng corresponds to ~76,000 potential UCI-BC templates at the *AAVS1* locus

5. Using the same primers as the Final plasmid pool QC, perform a PCR to amplify UCI-BCs from the genomic DNA, adding TruSeq adapters and DI, according to the adjusted calculations and cycling conditions:

Appendix Table SP1.19.
iPS2-seq transfected cells QC, PCR #1 mix

Reagent	Volume (µL)	Final conc.
gDNA iPS2-seq pool	Variable	500 ng
Q5 Reaction Buffer (5X)	10	1X
dNTP solution mix (10 mM)	1	200 µM
iPS2-seq_step2NGS_Primer (10 µM)	2.50	0.5 µM
iPS2-seq_NGS_R Primer (10 µM)	2.50	0.5 µM
Q5 Hot Start High-Fidelity DNA Polymerase (2 U/µL)	1	0.04 U/µL
Nuclease-free water	To 50	
Total volume	50	

Appendix Table SP1.20.
Cycling conditions for PCR #1

Steps	Temperature	Time	Cycles
Initial Denaturation	98 °C	30 sec	1
Denaturation	98 °C	10 sec	26 cycles
Annealing	64 °C	30 sec	
Extension	72 °C	20 sec	
Final extension	72°C	2 min	1
Hold (rapid ramp down)	4 °C	Hold	

6. Purify the expected ~144 bp amplification product and purify it using SPRIselect beads with a double side selection using 0.6X & 1.5X bead ratios to remove primer dimers and small fragments

7. Dilute the PCR product to 0.1 ng/µL in EB buffer and perform indexing PCR according to Appendix Table SP1.17 and Appendix Table SP1.18

8. An indexed library product of ~228 bp is expected. Purify the reaction using a double-sided SPRIselect bead cleanup (0.65X & 1.5X ratios)

9. Run 2 µL of the undiluted and 1:10 final library on a TapeStation High Sensitivity D1000 Screen Tape assay, following the manual.

   Note: This is the same amplicon described for the final plasmid QC (Figure SP1.6B), but adjusted to work on genomic DNA material

10. Perform NGS using MiSeq or NextSeq 1000/2000 with the following settings: read 1 - 68 cycles; index 1 - 10 cycles; index 2 - 10 cycles (>10,000 reads/clone)

OPTIONAL: hiPSC clone genotyping

This optional procedure can be implemented to validate the efficiency of biallelic *AAVS1* genome editing on a subset of hPSC clones, or to isolate homozygous-edited clones for validation studies. Genotyping leverages various genomic PCRs to determine on-site integration, copy number, and random integrations (Figure SP1.7)

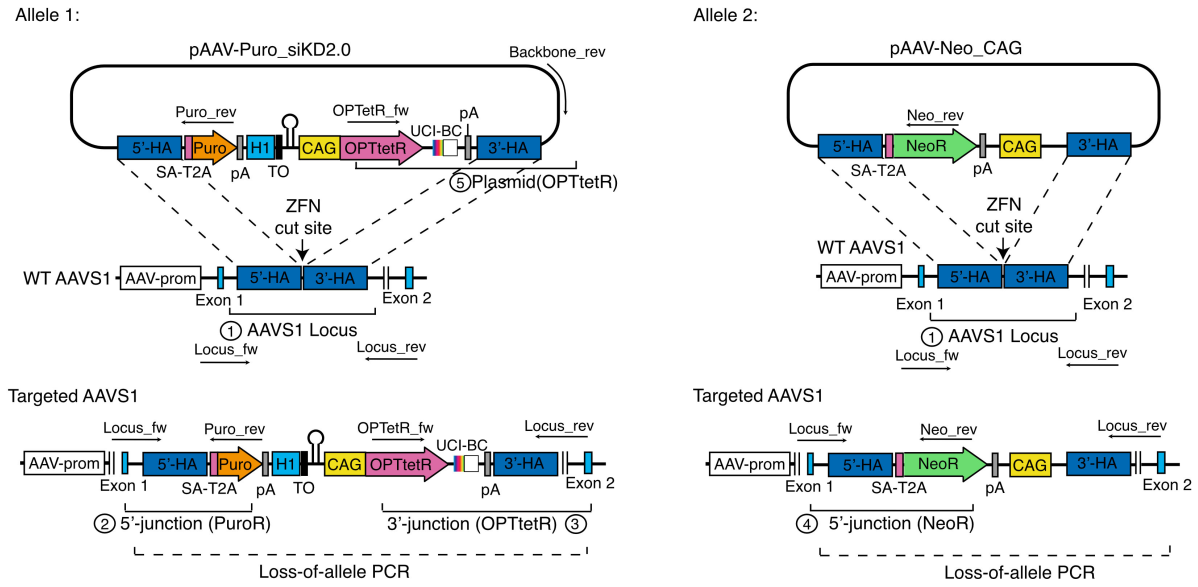

Figure SP1.7. iPS2-seq clone genotyping strategy

Schematic of the genotyping strategies to monitor integration of *pAAV-Puro_siKD2.0* in allele 1 and *pAAV-Neo_CAG* in allele 2. Primer binding sites and directions are shown on the plasmid or integrated cassettes. PCR types and products are numbered as in Appendix Table SP1.23.

1. After step 13 of Genome editing: obtain hPSC clonal pools, pick and expand individual hPSC clones.

   Note: treat clones with 200 nM FIAU to test for random integrations.

2. Prepare a culture dish with 2-5 x 10^5 cells, and extract gDNA using the Monarch Spin gDNA Extraction Kit, diluting to a concentration of 50-200 ng/µL

3. Perform PCR reactions according to the following calculations and cycling conditions, employing the primer pairs listed in Appendix Table SP1.23. Include the following controls: wild type hPSCs; pAAV-Puro_siKD2.0; no template

Appendix Table SP1.21.
Genotyping PCR mix

Reagent	Volume/reaction (µL)	Final conc.
Genomic DNA	1	50-200 ng
LongAmp Taq Reaction Buffer (5X)	2	1X
dNTP solution mix (10 mM)	0.3	300 µM
Forward primer (10 µM)	0.5	0.5 µM
Reverse primer (10 µM)	0.5	0.5 µM
DMSO	0,2	10
LongAmp Taq DNA Polymerase (2.5 U/µL)	0.4	0.1 U/µL
Nuclease-free water	5.1	
Total volume	10	

Appendix Table SP1.22.
Cycling conditions for genotyping

Steps	Temperature	Time	Cycles
Initial Denaturation	94 °C	5 min	1
Denaturation	94 °C	15 sec	
Annealing	Appendix Table SP1.23	30 sec	35 cycles
Extension	65 °C	Appendix Table SP1.23	
Final extension	65 °C	5 min	1
Hold (rapid ramp down)	4 °C	Hold	

4. Run half the PCR products on 0.8% (w/v) agarose gels and determine the genotype based on Appendix Table SP1.24

Appendix Table SP1.23. Genotyping strategies for iPS2-seq clone genotyping
[a]Result of PCR on wild type *AAVS1* allele
[b]Result of PCR on iPS2-seq-targeted *AAVS1* allele
[c]Result of PCR on pAAV-Puro_siKD2.0 (positive control for off-target plasmid integration)
[d]Variable parameter in PCR protocol (Appendix Table SP1.22)

PCR type NB	PCR type	Primer	Primer name	Primer location	Amplicon wild type[a]	Amplicon target[b]	Amplicon plasmid[c]	Annealing temp.[d]	Extension time[d]
1	AAVS1 locus	FW	Locus_fw	Genomic, 5' to 5' HA	1692 bp	No band	no band	65 °C	1' 30"
		REV	Locus_rev	Genomic, 3' to 3' HA					
2	5' junction (PuroR)	FW	Locus_fw	Genomic, 5' to 5' HA	no band	991 bp	no band	65 °C	1'
		REV	Puro_rev	Puro resistance					
3	3' junction (OPTetR)	FW	OPTtetR_fw	OPTtetR cDNA	no band	1463 bp	no band	60 °C	1' 30"
		REV	Locus_rev	Genomic, 3' to 3' HA					
4	5' junction (NeoR)	FW	Locus_fw	Genomic, 5' to 5' HA	no band	1032 bp	no band	60 °C	1'
		REV	Neo_rev	Neo resistance					
5	Plasmid (OPTtetR)	FW	OPTtetR_fw	OPTtetR cDNA	no band	no band	1819 bp	60 °C	2'
		REV	Backbone_rev	Backbone, 3' to 3' HA					

Appendix Table SP1.24. Interpretation of iPS2-seq clone genotyping

AAVS1 locus	5' junction (PuroR)	3' junction (OPTtetR)	5' junction (NeoR)	Plasmid (OPTtetR)	*AAVS1* genotype	FIAU sensitive?	Number of shRNAs
-	+	+	+	-	Compound heterozygous	No	1
-	+	+	+	+	Compound heterozygous	No	1
+	+	+	-	-	Heterozygous iPS2-seq	No	1
+	+	+	-	+	Heterozygous iPS2-seq	No	1
-	+	+	+	+	Compound heterozygous	Yes	>1
+	+	+	-	+	Heterozygous iPS2-seq	Yes	>1
-	+	+	-	-	Homozygous iPS2-seq	No	2
-	+	+	-	+	Homozygous iPS2-seq	No	2
-	+	+	-	+	Homozygous iPS2-seq	Yes	>2
+	-	-	-	-	Wild type	No	0
+/-	-	+/-	+/-	-	Incorrect iPS2-seq targeting	No	0
+/-	+/-	-	+/-	-	Incorrect iPS2-seq targeting	No	0
+/-	-	+/-	+/-	+	Incorrect iPS2-seq targeting	No	0
+/-	+/-	-	+/-	+	Incorrect iPS2-seq targeting	No	0
+/-	-	+/-	+/-	+	Incorrect iPS2-seq targeting	Yes	1 or more
+/-	+/-	-	+/-	+	Incorrect iPS2-seq targeting	Yes	1 or more

Appendix Protocol 2

iPS2-sci-seq library preparation

Decoding single cell transcriptional perturbomes in iPS2-seq relies on detecting the shRNA-associated UCI-BC within the 3' UTR of the OPTtetR transgene (Figure 1A). This can be in principle achieved with any scRNA-seq method that tags and counts the 3' ends of mRNAs, usually as a result of pA-primed RT.

iPS2-sci-seq is a home-brew adaptation of the 2-level indexing sci-RNA-seq protocol (Cao et al. 2017), that relies on 2 rounds of transcriptome barcoding through sequential pool-split of defined cell numbers so that the resulting combinatorial barcode in each cell is statistically most likely unique. This approach requires limited custom reagents and supports the analysis of 1,000-10,000 cells per experiment, a scale compatible with everyday needs of most laboratories. Minor adaptations of this protocol requiring some additional reagents can enable the 3-level indexing variant of sci-RNA-seq (sci-RNA-seq3), which supports the analysis of over ~1 million cells per experiment (Martin et al. 2023).

Since transcript counts in sci-RNA-seq are notoriously low and zero-inflated (i.e., genes may not be counted at least once in cells that express them), it is key to incorporate a strategy whereby the sequence containing the UCI-BC is specifically enriched, so as to detect it with higher sensitivity and specificity. Therefore, in the iPS2-sci-seq protocol, UCI-BCs are enriched at both transcriptome barcoding steps: first during RT, using a set of target-specific primers that share RT barcodes with standard pT primers, well by well, and secondly during PCR, using a set of target-specific primers that share i7 barcodes with standard P7 primers, once again well by well. The resulting multiplexed NGS library pools contain both single cell transcriptomes and UCI-BCs, which share library structure and carry identical combinatorial indexes, cell by cell (Figures EV1E and EV1F).

This Supplemental Protocol describes the following procedures:

1. Reagent setup
2. Nuclei preparation
3. Reverse transcription
4. Nuclei sorting
5. Second strand synthesis
6. Tagmentation
7. Indexing PCR and NGS

Reagent setup

1. Prepare the RT and PCR oligos using nuclease-free water:
   (a) Obtain master stocks of the 96 indexed iPS2-sci-seq_pT primers (250 µM) and iPS2-sci-seq_tetR primers (100 µM). Use the RT_indexes indicated in Appendix Table SP2.1
   (b) Prepare a working stock of multiplexed RT primers by diluting in the same plate iPS2-sci-seq_pT primers (25 µM) and iPS2-sci-seq_tetR primers (2.5 µM), matching well positions so that pooled primers share the same RT index
   (c) Obtain master stocks of indexed iPS2-sci-seq_P5, iPS2-sci-seq_P7, and iPS2-sci-seq_P7A (all at 100 µM). Use the i5 and i7 indexes indicated in Appendix Table SP2.1

   Note: it is not necessary to obtain 96 indexed oligos of each type, as i5 and i7 can be combined to generate unique combinations
   (d) Prepare a working stock of iPS2-sci-seq_P5 (10 µM)

(e) Prepare a working stock of multiplexed P7 primers by diluting in the same plate iPS2-sci-seq_P7 (10 µM) and iPS2-sci-seq_P7A (5 µM), matching well positions so that pooled primers share the same i7 index

2. Prepare the following buffers using nuclease-free water, and chill them on ice:

(a) Nuclei buffer (store at 4 °C for up to 1 month, 5 mL/sample): 10 mM Tris-HCl, pH 7.4, 10 mM NaCl, 3 mM MgCl2

(b) Nuclei lysis buffer (made fresh, 1 mL/sample): nuclei buffer with 0.1-0.3% IGEPAL CA-630, 1% SUPERase In RNase Inhibitor (20 U/µL) and 1% BSA (20 mg/mL)

Note: the optimal concentration of IGEPAL CA-630 for a given cell type should be determined in a pilot experiment. For reference, 0.1% works for hPSCs, but not for hPSC-CMs which are lysed appropriately only at 0.3%

(c) Nuclei suspension buffer (make fresh, 3 mL/sample): nuclei buffer with 1% SUPERase In RNase Inhibitor (20 U/µL) and 1% BSA (20 mg/mL)

(d) 4% PFA (store at 4 °C for up to 1 month, 10 mL/sample)

(e) DPBS without Ca2+ and Mg2+ (store at 4 °C for up to 1 month, 10 mL/sample)

Appendix Table SP2.1. RT and PCR indexes for iPS2-sci-seq

Well	RT_index (5'-3')	i7 for primer (5'-3')	i7 for sample sheet (5'-3')	i5 for primer & sample sheet, forward strand (5'-3')	i5 for sample sheet, reverse strand (5'-3')	Well	RT_index (5'-3')	i7 for primer (5'-3')	i7 for sample sheet (5'-3')	i5 for primer & sample sheet, forward strand (5'-3')	i5 for sample sheet, reverse strand (5'-3')
A01	TTCTCGCATG	CCGAATCCGA	TCGGATTCGG	CTCCATCGAG	CTCGATGGAG	E01	AGAGAAGGTT	TTCGTTCCAT	ATGGAACGAA	AGGCGAGAGC	GCTCTCGCCT
A02	TCCTACCAGT	ATAAGCCGGA	TCCGGCTTAT	TTGGTAGTCG	CGACTACCAA	E02	CATACTCCGA	TACCTAATCA	TGATTAGGTA	TCAAGATAGT	ACTATCTTGA
A03	GCGTTGGAGC	CCGGCGGCGA	TCGCCGCCGG	GGCCGTCAAC	GTTGACGGCC	E03	GCTAACTTGC	AAGTAATATT	AATATTACTT	TAATTGACCT	AGGTCAATTA
A04	GATCTTACGC	GGCTTGCCAA	TTGGCAAGCC	CCTAGACGAG	CTCGTCTAGG	E04	AATCCATCTT	AGCTAAGAAT	ATTCTTAGCT	CAGCCGGCTT	AAGCCGGCTG
A05	CTGATGGTCA	CCGCTAGCTG	CAGCTAGCGG	TCGTTAGAGC	GCTCTAACGA	E05	GGCTGAGCTC	GTCGAGGTAT	ATACCTCGAC	AGAACCGGAG	CTCCGGTTCT
A06	CCGAGAATCC	CTTATCCTAC	GTAGGATAAG	CGTTCTATCA	TGATAGAACG	E06	CCGATTCCTG	TTATTAGTAG	CTACTAATAA	GAGATGCATG	CATGCATCTC
A07	GCCGCAACGA	TGAGCTACTT	AAGTAGCTCA	CGGAATCTAA	TTAGATTCCG	E07	ACCGCCAACC	TGCGAAGATC	GATCTTCGCA	GATTACCGGA	TCCGGTAATC
A08	TGAGTCTGGC	TCAGGACTTA	TAAGTCCTGA	ATGACTGATC	GATCAGTCAT	E08	TGGCCTGAAG	AACTACGGCT	AGCCGTAGTT	TCGTAACGGT	ACCGTTACGA
A09	TGCGGACCTA	CCGCAGCCGC	GCGGCTGCGG	TCAATATCGA	TCGATATTGA	E09	AACCTCATTC	AACGGAACGC	GCGTTCCGTT	TGGCGACGGA	TCCGTCGCCA
A10	ACCTCGTTGA	TGCGCCTGGT	ACCAGGCGCA	GTAGACCTGG	CCAGGTCTAC	E10	ATAAGGAGCA	GATGCTACGA	TCGTAGCATC	AGTCATAGCC	GGCTATGACT
A11	ACGGAGGCGG	AATCATACGG	CCGTATGATT	TTATGACCAA	TTGGTCATAA	E11	CGAACGCCGG	ATCTGCCAAT	ATTGGCAGAT	GTCAAGTCCA	TGGACTTGAC
A12	TAGATCTACT	CGCCAATCAA	TTGATTGGCG	TTGGTCCGTT	AACGGACCAA	E12	GGTATGCTTG	ATCGTATCAA	TTGATACGAT	ATTCGGAAGT	ACTTCCGAAT
B01	AATTAAGACT	CAAGGCTTAG	CTAAGCCTTG	GGTACGTTAA	TTAACGTACC	F01	AACCTGCGTA	AACGCCTCTA	TAGAGGCGTT	GTCGGTAGTT	AACTACCGAC
B02	CCATTGCGTT	GCGCTCGACG	CGTCGAGCGC	CAATGAGTCC	GGACTCATTG	F02	GGCAGACGCC	ACGGCAACCA	TGGTTGCCGT	AGGACGGACG	CGTCCGTCCT
B03	TTATTCATTC	TCCAGCAATA	TATTGCTGGA	GATGCAGTTC	GAACTGCATC	F03	TAGCCGTCAT	CAGGCTAAGA	TCTTAGCCTG	CTCCTGGACC	GGTCCAGGAG
B04	ATCTCCGAAC	CATGAGAACT	AGTTCTCATG	CCATCGTTCC	GGAACGATGG	F04	CCTGGAAGAG	CGCAATATCA	TGATATTGCG	TAGCCTCGTT	AACGAGGCTA
B05	TTGACTTCAG	AACGTAATCT	AGATTACGTT	TTGAGAGAGT	ACTCTCTCAA	F05	GGAGGTTCTA	TTCGATAACC	GGTTATCGAA	GGTTGAACGT	ACGTTCAACC
B06	GGCAGGTATT	ATTCTCCTCT	AGAGGAGAAT	ACTGAGCGAC	GTCGCTCAGT	F06	CTAGTAGTCT	AACCTCAAGA	TCTTGAGGTT	AGGTCCTCGT	ACGAGGACCT
B07	AGAGCTATAA	TCTGCGCGTT	AACGCGCAGA	TGAGGAATCA	TGATTCCTCA	F07	ATCATCAACG	CAGGCGCCAT	ATGGCGCCTG	GGAAGTTATA	TATAACTTCC
B08	CTAAGAGAAG	GCTCATATGC	GCATATGAGC	CCTCCGACGG	CCGTCGGAGG	F08	ACGCGAGATT	AACTATTATA	TATAATAGTT	TGGTAATCCT	AGGATTACCA
B09	ACTCAATAGG	AGCGGTAACG	CGTTACCGCT	CATTGACGCT	AGCGTCAATG	F09	GAAGAGGCAT	AAGTTACCTA	TAGGTAACTT	AAGCTAGGTT	AACCTAGCTT
B10	CTTGCGCCGC	AATGAATAGT	ACTATTCATT	TCGTCCTTCG	CGAAGGACGA	F10	GGTATCCGCC	CGGCAGAGGA	TCCTCTGCCG	TCCGCGGACT	AGTCCGCGGA
B11	AATCGTAGCG	CCGTATCTGG	CCAGATACGG	TGATACTCAA	TTGAGTATCA	F11	AACTAGGCGC	GCCTCAATAA	TTATTGAGGC	TGCGGATAGT	ACTATCCGCA
B12	GGTACTGCCT	CCTTAGTCTG	CAGACTAAGG	TTCTACCTCA	TGAGGTAGAA	F12	TCGCTAAGCA	TTAACGCCGT	ACGGCGTTAA	TGGCAGCTCG	CGAGCTGCCA
C01	TAGAATTAAC	ACCTAGTTAG	CTAACTAGGT	TCGTCGGAAC	GTTCCGACGA	G01	TATATACTAA	CATACGATGC	GCATCGTATG	TGCTACGGTC	GACCGTAGCA
C02	GCCATTCTCC	ATAGGAGTAC	GTACTCCTAT	ATCGAGATGA	TCATCTCGAT	G02	ACTTGCTAGA	AAGCTGACCT	AGGTCAGCTT	GCGCAATGAC	GTCATTGCGC
C03	TGCCGGCAGA	CTACGACGAG	CTCGTCGTAG	TAGACTAGTC	GACTAGTCTA	G03	AACCATTGGA	GAGTCCTTAT	ATAAGGACTC	CTTAATCTTG	CAAGATTAAG
C04	TTACCGAGGC	AGTCGAGTTC	GAACTCGACT	GTCGAAGCAG	CTGCTTCGAC	G04	TCGCGGTTGG	CCTACGGCAA	TTGCCGTAGG	GGAGTTGCGT	ACGCAACTCC
C05	ATCATATTAG	TGGTCCAGTC	GACTGGACCA	AGGCGCTAGG	CCTAGCGCCT	G05	CGTAGTTACC	AATATTCGAA	TTCGAATATT	ACTCGTATCA	TGATACGAGT
C06	TGGTCAGCCA	ATCTAAGCAA	TTGCTTAGAT	AGATGCAACT	AGTTGCATCT	G06	TCCAATCATC	TTCAAGAATC	GATTCTTGAA	GGTAATAATG	CATTATTACC
C07	ACTATGCAAT	CGAATTCGTT	AACGAATTCG	AAGCCTACGA	TCGTAGGCTT	G07	AATCGATAAT	ATGCTCGCAA	TTGCGAGCAT	TCCTTATAGA	TCTATAAGGA
C08	CGACGCGACT	CAGCGATAGA	TCTATCGCTG	GTAGGCAATT	AATTGCCTAC	G08	CCATTATCTA	GGAGTAAGCC	GGCTTACTCC	CCGACTCCAA	TTGGAGTCGG
C09	GATACGGAAC	GGTCGCTATG	CATAGCGACC	TGCCAGTTGC	GCAACTGGCA	G09	TCAACGTAAG	TTATCGTATT	AATACGATAA	GCCAAGCTTG	CAAGCTTGGC
C10	TTATCCGGAT	ATCCGTTAGC	GCTAACGGAT	CTTAGGTATC	GATACCTAAG	G10	TCTAATAGTA	AAGTCTAATA	TATTAGACTT	CATATCCTAT	ATAGGATATG
C11	TAGAGTAATA	TCGCAATTAG	CTAATTGCGA	GAGACCTACC	GGTAGGTCTC	G11	AACCGCTGGT	CGGCTTACTA	TAGTAAGCCG	ACCTACGCCA	TGGCGTAGGT
C12	GCAGGTCCGT	GGCTGGCTAG	CTAGCCAGCC	ATTGACCGAG	CTCGGTCAAT	G12	GATCGCTTCT	GATATGGTCT	AGACCATATC	GGAATTCAGT	ACTGAATTCC
D01	TCGGCCTTAC	ACGGTCTTGC	GCAAGACCGT	GGAGGCGGCG	CGCCGCCTCC	H01	CTAACTAGAT	TAGTCGTCCA	TGGACGACTA	TGGCGTAGAA	TTCTACGCCA
D02	AGAACGTCTC	GCTCCATTCG	CGAATGGAGC	CCAGTACTTG	CAAGTACTGG	H02	GCTGGAACTT	TAGCTGCTAC	GTAGCAGCTA	ATTGCGGCCA	TGGCCGCAAT
D03	CCAGTTCCAA	ACGATAAGCG	CGCTTATCGT	GGTCTCGCCG	CGGCGAGACC	H03	AGGTTAGTTC	CTCTTCAAGC	GCTTGAAGAG	TTCAGCTTGG	CCAAGCTGAA
D04	GGCGTTAAGG	ACCATAGCGC	GCGCTATGGT	GGCGGAGGTC	GACCTCCGCC	H04	CATTCGACGG	ATGAACGCGC	GCGCGTTCAT	CCATCTGGCA	TGCCAGATGG
D05	ACTTAACCTT	CTCTTAGCGG	CCGCTAAGAG	TAGTTCTAGA	TCTAGAACTA	H05	CATTCAATCA	GTCGACGGAA	TTCCGTCGAC	CTTATAAGTT	AACTTATAAG
D06	CAACCGCTAA	TGATTCAACT	AGTTGAATCA	TTGGAGTTAG	CTAACTCCAA	H06	CGGATTAGAA	ACTAATTGAG	CTCAATTAGT	GATTAGATGA	TCATCTAATC
D07	GACCTTGATA	TATGGCCGCG	CGCGGCCATA	AGATCTTGGT	ACCAAGATCT	H07	ATCGGCTATC	CTTGCATAAT	ATTATGCAAG	TATAGGATCT	AGATCCTATA
D08	TCTGATACCA	AGAGGTCGCA	TGCGACCTCT	GTAATGATCG	CGATCATTAC	H08	CCTTGATCGT	TCCTTACCAA	TTGGTAAGGA	AGCTTATAGG	CCTATAAGCT
D09	GAAGATCGAG	AGGAGATTGA	TCAATCTCCT	CAGAGAGGTC	GACCTCTCTG	H09	ACGAAGTCAA	TGCAGCCTAC	GTAGGCTGCA	GTCTGCAATC	GATTGCAGAC
D10	AGGAGCGGTA	GGCTATATAG	CTATATAGCC	TTAATTAGCC	GGCTAATTAA	H10	TTACCTCGAC	GGAGCTGAGG	CCTCAGCTCC	CGCCTCTTAT	ATAAGAGGCG
D11	AAGAAGCTAG	TCGCGTACTT	AAGTACGCGA	CTCTAACTCG	CGAGTTAGAG	H11	GGAGGATAGC	GCAGCGGACT	AGTCCGCTGC	GTTGGATCTT	AAGATCCAAC
D12	TCCGGCCTCG	AATAATAATG	CATTATTATT	TACGATCATC	GATGATCGTA	H12	GGCTCTCTAT	CATCGCGCTC	GAGCGCGATG	GCGATTGCAG	CTGCAATCGC

Nuclei preparation

This is an exemplary protocol for one sample containing 1-5 x 10^6 starting cells. Perform all procedures on ice using chilled reagents and a refrigerated swing bucket centrifuge.

1. Obtain a single-cell suspension using a dissociation protocol that preserves >95% cell viability. Eliminate any cell clump using a 40-100 µm cell strainer

2. Extract and fix nuclei:

   - Centrifuge cells in a 15 mL conical at 150 g for 5 min, discard the supernatant, resuspend the pellet in 1 mL nuclei lysis buffer, and incubate for 5 min
   - Centrifuge nuclei at 300 g for 3 min, discard the supernatant, re-suspend the pellet in 100 µL nuclei suspension buffer , add 10 mL 4% PFA, mix and incubate for 15 min
   - Centrifuge fixed nuclei at 300 g for 5 min, discard the supernatant , and wash the pellet three times with 1 mL nuclei suspension buffer by repeating this step
   - After the final wash, resuspend the pellet in 300 µL nuclei suspension buffer

3. Count nuclei with a hemocytometer with YOYO-1 staining (Figure SP2.1), dilute them to 2,500 nuclei/µL , prepare single-use aliquots, and either proceed with the next section or snap-freeze nuclei in liquid nitrogen and store them at -80 °C for up to one month

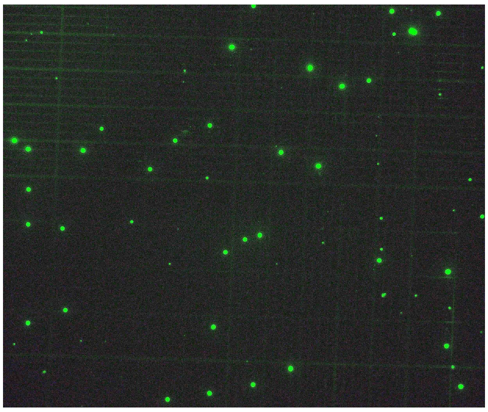

Figure SP2.1. Nuclear extraction QC
Exemplary picture of PFA-fixed nuclei stained with YOYO-1 visualized on a hemocytometer. Nuclei are round, not clumped, and there are minimal cell debris.

Reverse transcription

Clean the work area and instrumentation to prevent RNase contamination. Unless stated otherwise, keep samples on ice, perform centrifugations at 4 °C, use DNA LoBind 1.5 mL tubes and low-retention tips, and set the thermocycler heated lid at 105 °C to prevent evaporation.

1. Prepare fresh stop solution (40 mM EDTA and 1 mM spermidine; 600 µL/plate)

2. Thaw PFA-fixed nuclei in hand or at 37 °C thermal block, mix by flicking, then move to ice

3. Prepare a mix by combining nuclei suspension (2 µL, 5,000 nuclei, per well) and 10 mM dNTP (0.25 µL per well), and distribute 2.25 µL into each well of a 96-well LoBind plate

4. Add 1 µL of multiplexed RT primers to each well (step 1b of Reagent setup). Cap the plate and centrifuge it for 10 seconds at 100 g

5. Incubate the plate at 55 °C for 5 minutes and immediately place it on ice. After 3-5 minutes on ice, centrifuge the plate for 10 seconds at 100 g

6. Prepare the following RT mix, distribute 1.75 µL to each well without mixing, cap the plate, and centrifuge it for 10 seconds at 100 g

Appendix Table SP2.2. iPS2-sci-seq RT mix

Reagent	Volume per well (µL)	Volume per plate (µL)
5X Superscript IV First-strand buffer	1	105
100 mM DTT	0.25	26.25
Superscript IV reverse transcriptase	0.25	26.25
RNAseOUT Recombinant Ribonuclease Inhibitor	0.25	26.25

7. Incubate the plate at 55 °C for 10 minutes, immediately place it on ice, and add 5 µL of stop solution into each well to stop the reaction. After 3-5 minutes on ice, centrifuge the plate for 10 seconds at 100 g

8. Gently pool all wells into a FACS tube using wide bore tips, passing through a 40 µm cell strainer, and immediately proceed with the next procedure

Nuclei sorting

1. Prepare collection plate(s) by adding 5 µL of elution buffer to each well one or more 96-well LoBind plates, cover, and place on ice

2. Aliquot 50 µL of nuclei suspension (8 from Reverse transcription) in a separate tube to be used as unstained control; add 3 µM DAPI to the remaining volume to stain nuclei

3. Setup gating for single nuclei (Figure SP2.2), and sort 25 nuclei into each wells of collection plate (assuming 96 indexes in the RT and an accepted rate of ~10% index collisions)

   Note: use a cell sorter equipped with a 100 µm nozzle and minimize sample pressure, chill both sample and collection plate, and mix the sample while sorting

4. Centrifuge the plates for 1 minute at 900 g, and either proceed with the next step or store plates at -80 °C for up to one week

Second strand synthesis

1. Prepare reaction master mix, distribute 1 µL to each well, pipette mix, cap the plate, and centrifuge it for 10 seconds at 100 g

Appendix Table SP2.3. iPS2-sci-seq second strand synthesis mix

Reagent	Volume per well (µL)	Volume per plate (µL)
mRNA Second Strand Synthesis buffer	0.6	67.5
mRNA Second Strand Synthesis enzyme	0.3	33.75
Nuclease-free H2O	0.1	11.25

2. Carry out second strand synthesis at 16 °C for 180 minutes, and terminate the reaction by incubating at 75 °C for 20 minutes (lid kept at 80 °C for both steps)

3. Centrifuge the plate for 10 seconds at 100 g and either immediately proceed with the next section or store at 4 °C overnight

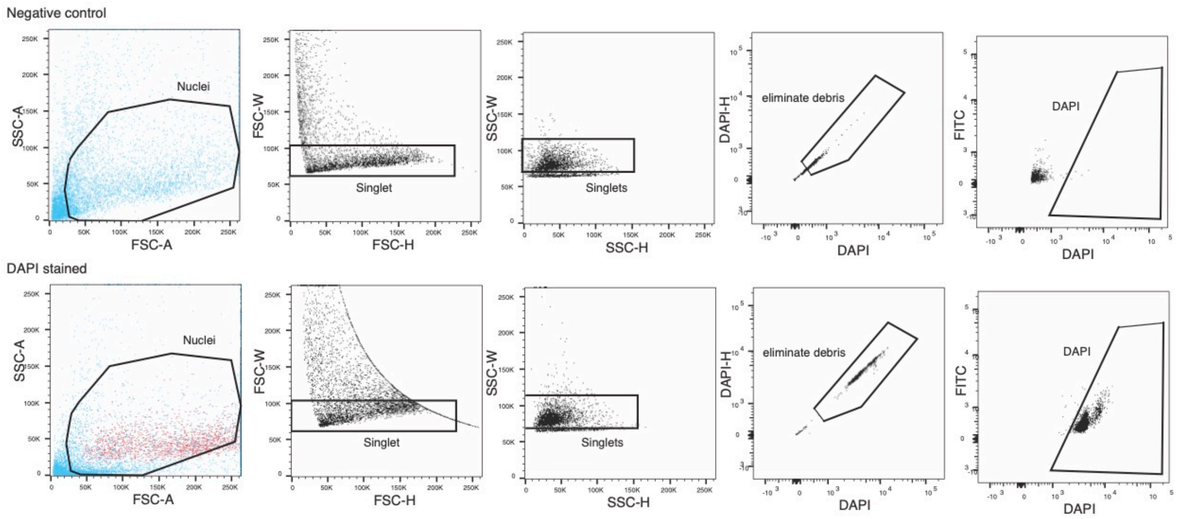

Figure SP2.2. Nuclei FACS
Exemplary gating strategy for single nuclei sorting. Set up the gates based on the negative control (top) and sort the DAPI+ population. DAPI+ single nuclei are back-gated in red; debris are in light blue.

Tagmentation

1. Prepare the tagmentation mix with the Nextera TD buffer (5.75 µL/well) and the TDE1 enzyme (0.01 µL/well), distribute 5.76 µL of mix to each well of the sorting plate, pipette mix, cap the plate, and centrifuge it for 10 seconds at 100 g

2. Incubate the plate at 55 °C for 5 min, immediately place it on ice, add 12 µL of Zymo DNA binding buffer to each well, and incubate at room temperature for 5 min.

3. Purify tagmented dsDNA using 1.5X volumes of SPRIselect beads (36 µL/well), following the manufacturer's instructions and eluting in 17 µL of elution buffer

4. Transfer 16 µL of eluted DNA to a new 96-well LoBind plate and either proceed with the next section or store at 4 °C overnight

Indexing PCR and NGS

1. Add 2 µL of 10 µM P5 primer and 2 µL of 10 µM multiplexed P7 primers (step 1e from Reagent setup) to each well, applying a unique combination of i5 and i7

2. Add 20 µL NEBNext High-Fidelity 2X PCR Master Mix to each well, pipette mix, cap the plate, and centrifuge it for 10 seconds at 100 g, and run the following PCR:

Appendix Table SP2.4. iPS2-sci-seq indexing PCR cycling conditions

Steps	Temperature	Time	Cycles
Initial denaturation	98 °C	30 sec	1
Denaturation	98 °C	10 sec	
Annealing	66 °C	30 sec	20
Extension	72 °C	1 min	
Final extension	72 °C	5 min	1

3. Pool all samples of a 96-well plate in a 15 mL Falcon tube and concentrate on 4 columns of the Zymo DNA Clean & Concentrator kit, following the manufacturer's instructions except without performing any washes and eluting in a total volume 25 µL of elution buffer

4. Further purify using 0.85X volumes of SPRIselect beads, according to the manufacturer's instructions and eluting in 50 µL of elution buffer

5. Run 2 µL of a 1:10 dilution of the NGS library on a TapeStation High Sensitivity 5000 Screen Tape assay, following the manual. Expect a main peak at ~350 bp (Figure SP2.3)

6. Perform NGS using NextSeq 1000/2000 and 50 cycles reagents: read 1 - 18 cycles; index 1 & index 2 - 10 cycles each; read 2 - 52 cycles (>25,000 reads/nuclei)

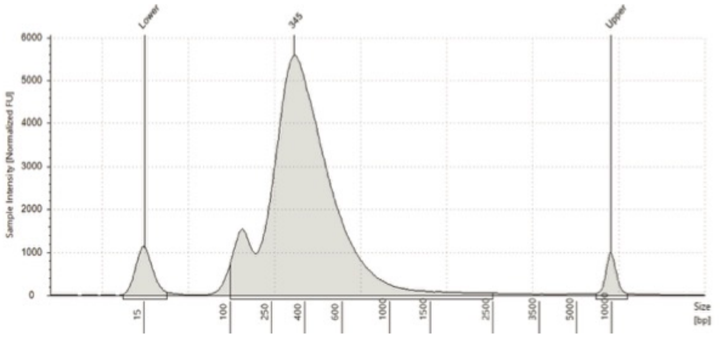

Figure SP2.3. iPS2-sci-seq NGS library QC
Exemplary TapeStation QC of iPS2-sci-seq NGS library (1:10 dilution).

Appendix Protocol 3

iPS2-10X-seq library preparation

iPS2-10X-seq relies on microfluidics partitioning-based scRNA-seq from 10X Genomics. Despite the higher costs, the ease and reproducibility of this commercial method have led to its widespread adoption. The procedure integrates the manufacturer's protocol to generate an additional NGS library containing UCI-BCs. This is sequenced in parallel to the standard gene expression library to enable *in silico* matching of UCI-BCs and single cell transcriptomes through their shared unique cell barcodes.

Of note, iPS2-10X-seq provides built-in support for the cost-saving strategy of reasonably overloading the microfluidics chip, since cell doublets are readily identified by the co-expression of two different UCI-BCs and filtered out as part of the standard *catcheR_10Xcatch* pipeline. Thus, it is possible to load the cell number recommended by the manufacturer for barcoded sample pools even in the absence of additional multiplexing barcodes.

Other cost-saving strategies include the use of cell multiplexing oligos (CMOs) or barcoded antibodies against housekeeping cell surface antigens to multiplex tet-untreated and tet-treated cells in a single microfluidics channel, enabling robust control versus knockdown experimental design without having to perform two separate reactions.

This Supplemental Protocol describes the following procedures:

1. Prepare the gene expression library
2. Prepare the shRNA barcode library
3. Library pooling and NGS

Prepare the gene expression library

This protocol was optimized using the Chromium Next GEM Single Cell 3' Reagent Kits v3.1 (Dual Index) from 10X Genomics and has been successfully validated with the next generation GEM-X Single Cell 3' Reagent Kits v4, without requiring changes to primer sequences. It must be used in conjunction with the appropriate 10X Genomics protocols: CG00315 Rev E (single sample), CG000388 Rev B (multiplexed samples), or CG000419 Rev D (high-throughput) for v3.1; and CG000731 Rev A for v4. Notably, v4 no longer supports cell multiplexing oligo (CMO)-based sample multiplexing but remains fully compatible with antibody-derived tag (ADT)–based strategies.

A first variation, iPS2-CITE-seq, enables surface protein profiling and ADT-based multiplexing using barcoded TotalSeq-B antibodies from BioLegend. When using antibodies, refer to protocol CG000149 Rev D (antibody labeling) in combination with CG000317 Rev E for v3.1 or CG000731 Rev A for v4.

A second variation, iPS2-multi-seq, adapts the protocol for use with the 10X Genomics Single Cell Multiome ATAC & Gene Expression kit. It should be paired with CG000365 Rev C (nuclei isolation) and CG000338 Rev F (library construction).

1. Obtain a single-cell suspension using a dissociation protocol that preserves >95% cell viability. Avoid the use of DNase, and remove any EDTA with at least three washes
2. **OPTIONAL**: Label multiple samples using Totalseq-B hashtags and/or other antibodies with ADTs, following the manufacturer's instructions (CG000149 Rev D)
3. **OPTIONAL**: Label multiple samples using cell multiplexing oligos (CMOs) in the 3' CellPlex Kit from 10X Genomics, following the manufacturer's instructions (CG000391 Rev B).

   Note: ADTs and CMOs can be combined if needed. After washing once post ADT incubation, start CMO labeling and follow the manufacturer's instructions (CG000390 Rev C)

4. **OPTIONAL**: Sort live cells after staining with Fixable Viability Dye eFluor 780

   Note: this step increases the fraction of cells that deliver high quality transcriptomes, and is particularly recommended for organoids that are difficult to dissociate. Avoid the use of other live/dead staining that can intercalate with DNA

5. Centrifuge cells/cell pools at 150 g for 5 min at 4°C, and resuspend in ice cold DPBS 1% BSA aiming for 1 x 10^6 cells/mL

6. Manually count cells, and calculate the cell volume needed to load the target cell number per channel of Chromium Next GEM Chip G

   Note: since iPS2-seq cells express shRNA barcodes, chips can be reasonably overloaded even without any CMO or hashtags (~49,500 cells/reaction at a ~24% doublet rate)

7. Follow the 10X Genomics protocols to obtain and quality control the gene expression library (GEX) and, when appropriate, the CMO library and/or the ADT library

8. Save a portion of the pre-amplified full-length cDNA for the next procedure

Prepare the shRNA barcode library

Perform a two-step PCR to enrich the UCI-BC region in the 3' UTR of the OPTtetR cDNA

1. Perform the first PCR according to the calculations and cycling conditions below:

Appendix Table SP2.1.
iPS2-10X-seq first PCR mix

Reagent	Volume (µL)
NEBNext® High-Fidelity 2X PCR Master Mix	10
iPS2-10X-seq_truseq (10 µM)	1
iPS2-10X-seq_inner (10 µM)	1
Preamplified 10X Genomics cDNA (50 ng)	Variable
Nuclease-free water	To 10
Total volume	20

Appendix Table SP2.2.
Cycling conditions for iPS2-10X-seq first PCR

Steps	Temperature	Time	Cycles
Initial denaturation	98 °C	30 sec	1
Denaturation	98 °C	10 sec	
Annealing	60 °C	20 sec	15
Extension	72 °C	20 sec	
Final extension	72 °C	30 sec	1

   Note: When performing iPS2-multi-seq, adjust this step by increasing the input material to 200 ng and reducing the cycle amplification to 11 cycles. Adaptations of the current protocol were essential to obtain clean UCI-BC library profiles

2. Purify the PCR with 1.8X volumes of SPRIselect beads, following the manufacturer's instructions and eluting in 20 µL of elution buffer

3. Run and quantify 2 µL of undiluted PCR on a TapeStation High Sensitivity 5000 Screen Tape assay, following the manual. Expect a main peak at ~285 bp (Figure SP3.1A)

4. Dilute the first PCR to 0.1 ng/µL and use it as template for the second PCR according to the calculations and cycling conditions below:

   Note: use a different index from the one utilized to prepare the GEX, CMO, and/or library, to allow subsequent demultiplexing of the UCI-BC library

Appendix Table SP2.3.
iPS2-10X-seq second PCR mix

Reagent	Volume (µL)
NEBNext® High-Fidelity 2X PCR Master Mix	10
Dual Index Kit TT Set A primers	4
First PCR (0.1 ng/µL)	1
Nuclease-free water	5
Total volume	20

Appendix Table SP2.4.
Cycling conditions for iPS2-10X-seq second PCR

Step	Temperature	Timing	Cycles
Initial denaturation	98 °C	30 sec	1
Denaturation	98 °C	10 sec	
Annealing	58 °C	20 sec	10
Extension	72 °C	20 sec	
Final extension	72 °C	30 sec	1

5. Purify and perform a double-sided size selection using 0.7X & 0.9X volumes of SPRIselect beads, following the manufacturer's instructions and eluting in 20 µL of elution buffer

6. Run 2 µL of undiluted PCR on a TapeStation High Sensitivity 5000 Screen Tape assay, following the manual. Expect a main peak at ~385 bp (Figure SP3.1B)

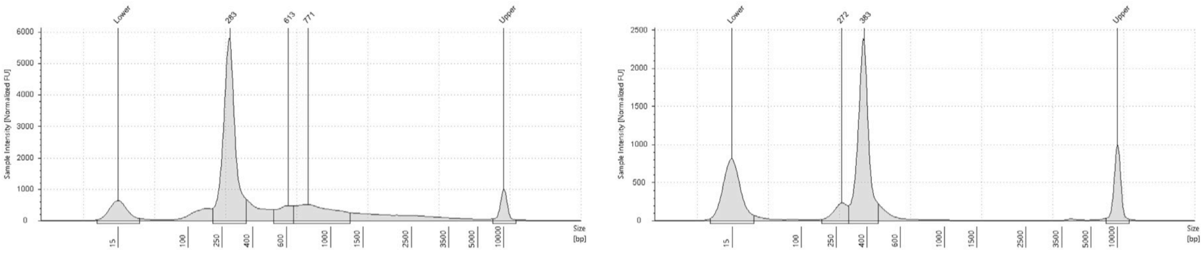

(A) (B)

Figure SP3.1. Exemplary TapeStation QC of iPS2-10X-seq first (A) and second (B) PCR.

Library pooling and NGS

1. Quantify the GEX and, when appropriate, CMO and/or ADT libraries (step 7 of Prepare the gene expression library), and the UCI-BC library (step 6 of Prepare the shRNA barcode library) with a Qubit dsDNA High Sensitivity kit

2. Pool GEX, CMO, ADT and UCI-BC libraries at a relative molar ratio of 60:10:10:3

3. Perform NGS using NextSeq 1000/2000 and 100 cycles reagents, with the following settings: read 1 - 28 cycles; index 1 - 10 cycles; index 2 - 10 cycles each; read 2 - 90 cycles (>30,000 reads/target cells)

Appendix Protocol 4

iPS2-seq design and analysis with *catcheR*

This Supplemental Protocol describes the following procedures:

1. Overview
2. Installation
3. Oligonucleotides design
4. Pooled cloning step 1 QC
5. Pooled cloning step 2 and hiPSC genome editing QC
6. iPS2-10X-seq perturbation deconvolution
7. iPS2-sci-seq perturbation deconvolution
8. Barcode reassignment
9. Perturbation effect analysis

Overview

catcheR is a comprehensive bioinformatic package for designing and analyzing iPS2-seq experiments. It comprises the following functions (Figure SP4.1):

1. *catcheR_design*, which designs oligonucleotides for Appendix Protocol 1 - Design shRNA oligonucleotides, facilitating shRNA library cloning
2. *catcheR_step1QC*, which analyzes the results of Appendix Protocol 1 - Intermediate plasmid pool QC, assessing pooled cloning step 1 plasmids for barcode swaps
3. *catcheR_step2QC*, which analyzes the results of Appendix Protocol 1 - Final plasmid pool QC or hiPSC pool QC, assessing pooled cloning step 2 or genome-edited hiPSC pools for shRNA representation
4. *catcheR_scicount*, which analyzes 2-level indexing sci-RNA-seq data, facilitating the generation of gene expression matrix for iPS2-sci-seq experiments
5. *catcheR_scicatch*, which assigns shRNA perturbations to single nuclei transcriptomes obtained by Appendix Protocol 2, enabling the primary analysis of iPS2-sci-seq
6. *catcheR_10Xcatch*, which assigns shRNA perturbations to single cell transcriptomes obtained by Appendix Protocol 3, enabling the primary analysis of iPS2-10X-seq
7. *catcheR_scicatchQC* and *catcheR_10XcatchQC*, which use the outputs of *catcheR_ scicatch* and *catcheR_10Xcatch*, respectively, to fine-tune shRNA assignment thresholds
8. *catcheR_filtercatch*, which leverages on the output of *catcheR_scicatchQC* and *catcheR_ 10XcatchQC* to filter single nuclei/cell transcriptomes expressing a single shRNA
9. *catcheR_sortcatch*, which quality controls the cell-by-gene matrix based on the results of *catcheR_step1QC*, reassigning hPSC clones with barcode swaps to the correct shRNA
10. *catcheR_scinocatch* and *catcheR_10Xnocatch*, which identify cells expressing no shRNA in iPS2-sci-seq and iPS2-10X-seq experiments, respectively, adding them to the cell-by-gene matrix to be used as additional controls

11. *catcheR_load*, which loads gene expression matrices annotated with shRNA perturbations into a Monocle object, preparing the dataset for downstream analysis

12. *catcheR_pseudotime*, which analyzes the effects of shRNA perturbations on pseudotime dynamics, highlighting shifts along differentiation trajectories (e.g., Figures 2J and 2K)

13. *catcheR_modules*, which assesses perturbation-induced changes in gene module expression, such as coordinated activation or repression of functional programs (e.g., Figures 5H–5J)

14. *catcheR_enrichment*, which quantifies differences in perturbation representation across experimental samples (e.g., Figures 5F and 5G) or cell clusters (e.g., Figure 5E)

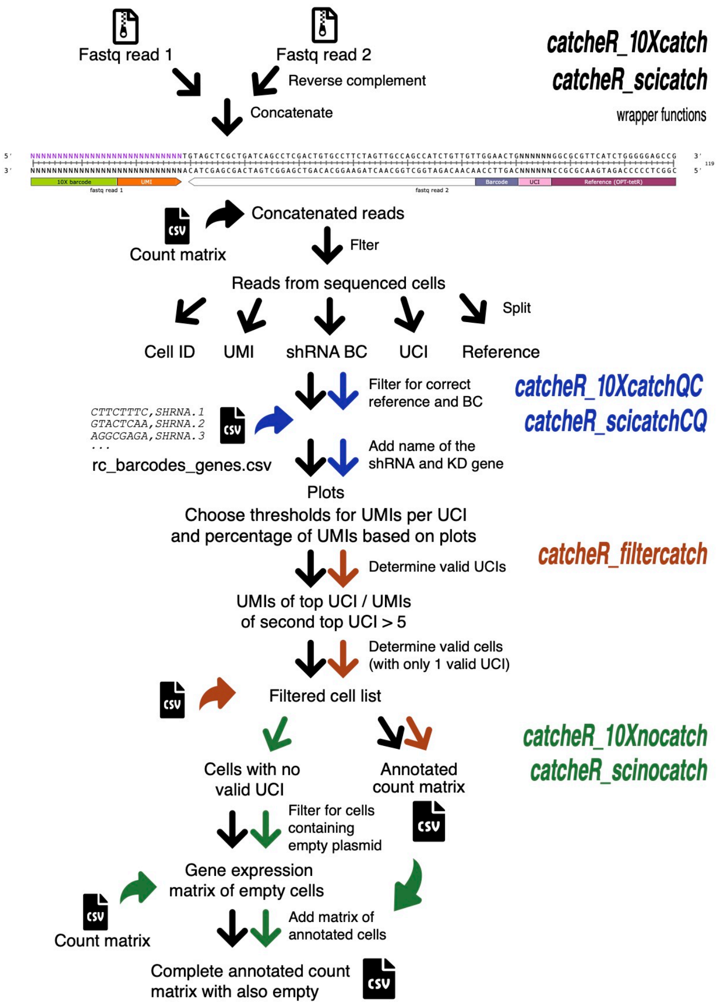

Figure SP4.1. Overview of *catcheR*

Logical relationship and main inputs/outputs for the *catcheR* analytical pipeline to assign perturbation in iPS2-seq experiments. Functions used for oligo design, plasmid/hiPSC QC, and perturbation effect analyses are not illustrated.

Installation

catcheR is available at https://github.com/alessandro-bertero/catcheR. The GitHub repository folder ”scripts” contains all the bash and R scripts that can be run independently. However, in order to ensure reproducible analyses, we strongly recommend to install *catcheR* package from GitHub, since its functions run all the analysis inside Docker containers.

1. Install Docker engine, following the instructions at https://docs.docker.com/engine/install/
2. Install *catcheR*
   (a) In R (≥ 3.0.2) install *devtools*, if not already present
   ```
   install.packages("devtools")
   ```
   (b) Install *catcheR* from GitHub
   ```
   install_github("alessandro-bertero/catcheR")
   ```
   (c) Install rrundocker from GitHub
   ```
   install_github("Reproducible-Bioinformatics/rrundocker")
   ```
   (d) Load *catcheR* and rrundocker in your R environment
   ```
   library(catcheR)
   library(rrundocker)
   ```

Oligonucleotides design

This step complements Appendix Protocol 1 - Design shRNA oligonucleotides.

1. In a new working folder, prepare the following files:
   (a) A comma-separated values (CSV) file with three columns listing: (1) the forward oligos from the TRC shRNA library; (2) the corresponding barcodes (BC); (3) the shRNA names. Below is an example for the shRNA described in Figure SP1.2:
   ```
   CCGGCAAGTACTCCTTGCTGGATTGCTCGAGCAATCCAGCAAGGAGTACTTGTTTTTG,CAGTTCCA,SMAD2.1
   ...
   ```
   (b) (Optional) - A txt file with a newline-separated list of 5’-3’ restriction sites, or other sequences, to be avoided in the shRNAs. By default these are SalI, SwaI and AscI:
   ```
   GTCGAC
   ATTTAAAT
   GGCGCGCC
   ```
2. Run *catcheR_design*:
   ```
   catcheR_design(
       group=c("docker","sudo"),
       folder,
       sequences,
       gibson.five = "AGTTCCCTATCAGTGATAGAGATCCC",
       gibson.three = "GTAGCTCGCTGATCAGC",
       fixed = "GTCGACATTTAAATGGCGCGCC",
       restriction.sites = NULL)
   ```

catcheR_design arguments:

(a) group: string with two options: sudo or docker, depending on the user group. For a detailed explanation of Docker user groups, see this page
(b) folder: string with the working folder path
(c) sequences: string with the CSV file name from step 1a
(d) gibson.five: (optional) string with the 5' Gibson homology
(e) gibson.three: (optional) string with the 3' Gibson homology
(f) fixed: (optional) character string with the multicloning site
(g) restriction.sites: (optional) string with the txt file name from 1b

Example usage:

```
catcheR_design(
    group = "docker",
    folder = "path/to/folder",
    sequences = "filename.csv",
    restriction.sites = "filename.txt")
```

catcheR_design outputs:

(a) "output.txt", with the oligo sequences to be used for synthesis (Figure SP1.2)
(b) "bad_oligos.txt", with the shRNAs with forbidden restriction enzyme sites (highlighted in lowercase characters); this information can be used to refine the shRNA list.
Example output for "bad_oligos.txt":

```
AGTTCCCTATCAGTGATAGAGATCCCGGACATAATCACTGCGTAATCCTCagatctTACGCAGTGATTATGTCCTTTTTTTGT-
CGACATTTAAATGGCGCGCCNNNNNNGCTGAAGAGTAGCTCGCTGATCAGC,GATA4
...
```

Pooled cloning step 1 QC

This step complements Appendix Protocol 1 - Intermediate plasmid pool QC.

1. In a new working folder, prepare the following files:

(a) Fastq/fq or fastq.gz files with demultiplexed read 1 from the NGS run
(b) A CSV file with the shRNA names and their full sequences

```
SMAD2.1,GCAAGTACTCCTTGCTGGATTGCTCGAGCAATCCAGCAAGGAGTACTTG
...
```

(c) (Optional) - A txt file with a newline-separated list of clones of interest as "BC_UCI"

```
CAAGAGCC_CATCGT
...
```

2. Run *catcheR_step1QC*:

```
catcheR_step1QC(
    group=c("docker","sudo"),
    folder,
    fastq.read1,
    DIs = 100,
    ratio = 10,
    plot.threshold = 2000,
    clones = NULL)
```

catcheR_step1QC arguments:

(a) group: string with two options: sudo or docker, depending on the user group (info)
(b) folder: string with the working folder path
(c) fastq.read1: string with the read 1 filename from step 1a
(d) DIs: integer of the minimum number of diversity indexes (DIs, pseudo-unique reads) of the most represented shRNA matching to a given UCI-BC; in combination with ”ratio”, it selects UCI-BCs for which it is possible to reliably assign an shRNA
(e) ratio: integer of the minimum ratio between the number of DIs of the most represented and second most represented shRNAs matching to a given UCI-BC
(f) plot.threshold: integer of the minimum number of DIs per UCI-BC for output 2b
(g) clones: (optional) a string with the txt file from step 1c

Example usage:

```
catcheR_step1QC(
    group = "docker",
    folder = "path/to/folder",
    fastq.read1 = "filename.fq",
    clones = "filename.txt")
```

catcheR_step1QC key outputs:

(a) ”reliable_clones_swaps.csv”, which lists UCI-BCs with reliable evidence of shRNA-barcode swap, and is used as input for Barcode reassignment
(b) Bar chart of the number of DIs for each clone above ”plot.threshold”
(c) Bar chart of the number of DIs associated to each BCs, shRNAs, and reliable swaps
(d) (If ”clones” argument is provided) - CSV files and bar charts with the number of DIs for each shRNA matching to each clone of interest (Figure SP4.2)

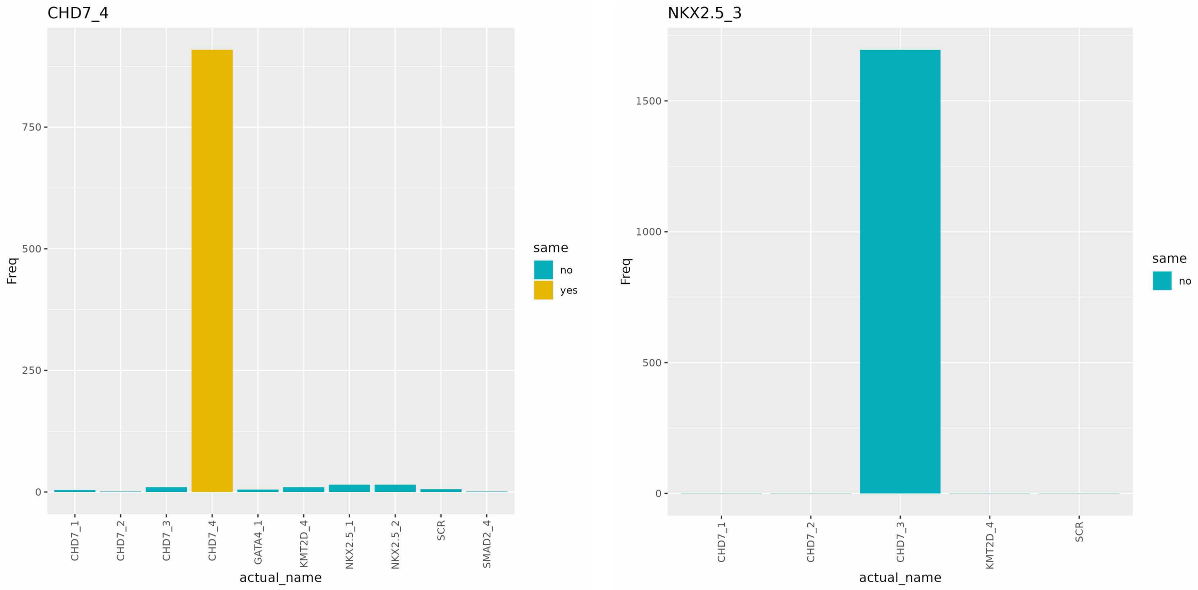

(A) **(B)**

Figure SP4.2. Example of quantification of DIs for bacterial clones carrying the expected shRNA (A) or with robust evidence of a barcode swap that can be reassigned (B; Figure 1B). These graphs allow visual evaluation of appropriate thresholds for ”DI” and ”ratio”.

Pooled cloning step 2 and hiPSC genome editing QC

This step complements Appendix Protocol 1 - Final plasmid pool QC and hiPSC pool QC.

1. In a new working folder, prepare the following files:

   (a) Fastq/fq or fastq.gz files with demultiplexed read 1 from the NGS run

   (b) ”rc_barcodes_genes.csv”, a CSV file with two columns: (1) the shRNA BCs; (2) the matching shRNA names in the format ”GENE.shRNAID”

   ```
   CAAGAGCC,SMAD2.1
   ...
   ```

   (c) (Optional) - A txt file with clones of interest (step 1c of Pooled cloning step 1 QC)

2. Run *catcheR_step2QC*:

   ```
   catcheR_step2QC(
       group=c("docker","sudo"),
       folder,
       fastq.read1,
       DIs = 1000,
       clones = NULL)
   ```

   catcheR_step2QC arguments:

   (a) group: string with two options: sudo or docker, depending on the user group (info)

   (b) folder: string with the working folder path

   (c) fastq.read1: string with the read 1 file from step 1c

   (d) DIs: integer of the minimum number of DIs for a given UCI-BC; it selects reliably-measured UCI-BCs

   (e) clones: (optional) a string with the txt file from step 1c

   Example usage:

   ```
   catcheR_step2QC(
       group= "docker",
       folder = "path/to/working/folder",
       fastq.read1 = "filename.fq",
       clones = "filename.txt")
   ```

   catcheR_step2QC key outputs:

   (a) Pie charts of DIs per shRNA and per gene targets associated to each clone

   (b) Text file listing all clones above the DI threshold

   (c) Bar chart of the number of DIs for each clone above the DI threshold (Figure SP4.3A)

   (d) Frequency histogram of the percentage of clones above the DI threshold for each shRNA (Figure SP4.3B)

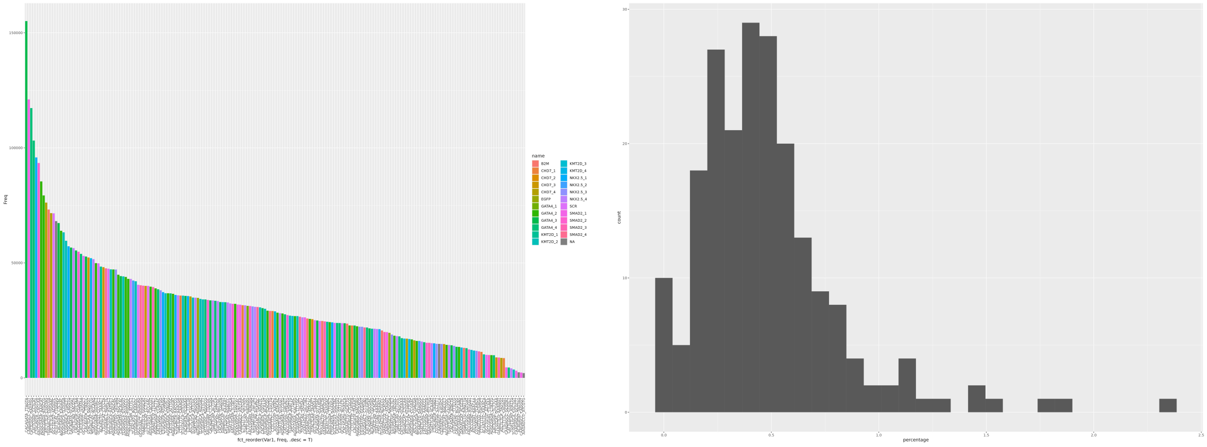

(A) **(B)**

Figure SP4.3. Example of quantification of DIs for bacterial clones represented by more than 1,000 DIs (A), and of the frequency of bacterial clones for each shRNA. These graphs confirm whether shRNAs are normally distributed in the final plasmid pool.

Obtain iPS2-10X-seq, iPS2-CITE-seq, or iPS2-multi-seq count matrices

1. Download and install *cellranger* (for iPS2-10X-seq and iPS2-CITE-seq) or *cellranger-arc* (for iPS2-multi-seq). Alternatively, Docker containers for *cellranger* v7, *cellranger* v9 (recommended for iPS2-CITE-seq), or *cellranger-arc* can be pulled from our Docker repository

2. For iPS2-10X-seq and iPS2-CITE-seq, demultiplex Illumina base call (BCL) files using *cellranger mkfastq*, following the standard 10X Genomics user guide. In the sample sheet CSV, include the index sequences used in Appendix Protocol 3 for the GEX and UCI-BC libraries, and, when applicable, CMO and/or ADT libraries

3. For iPS2-multi-seq, use *cellranger-arc mkfastq* to demultiplex GEX + ATAC dual-index libraries, following the Cell Ranger ARC user guide. Ensure the sample sheet format is compatible with dual-modality sequencing runs, and include the index sequences used for both gene expression and chromatin accessibility libraries

4. Run *FastQC* to assess the quality of the FASTQ files for each library type

5. Generate cell-by-gene count matrices using *cellranger count* (for single-sample experiments) or *cellranger multi* (for multiplexed experiments, including iPS2-CITE-seq). For iPS2-multi-seq, use *cellranger-arc count* to obtain both gene expression and chromatin accessibility matrices

   Note: In multiplexed experiments (e.g., using CMO or ADT barcodes in iPS2-CITE-seq), count matrices from individual samples can be aggregated using *cellranger aggr* to produce a unified dataset. This enables joint analysis in a single run of *catcheR_10Xcatch* by specifying the number of samples *via* the `samples` argument

6. Use *cellranger mat2csv* to convert the sparse matrix output into a dense CSV format for compatibility with downstream tools. For iPS2-multi-seq, run *cellranger-arc mat2csv* separately for GEX and ATAC outputs, if needed

After obtaining the count matrix, proceed to iPS2-10X-seq perturbation deconvolution.

Obtain iPS2-sci-seq count matrices

1. *catcheR_scicount* is a wrapper of the "bbi-sci" pipeline developed by the Brotman Baty Institute for Precision Medicine, which was implemented and dockerized in *catcheR* to be used on any operating system.

2. Demultiplex Illumina base calls to fastq files

   (a) Create a "SampleSheet.csv" file with as many sample rows as the PCR wells from Indexing PCR and NGS of Appendix Protocol 2, where "Sample_ID" is the well identified with format [A-H][01-12], and index and index2 are the i7 and i5 indexes used in the corresponding row and column (refer to Appendix Table SP2.1)

   (b) Run Illumina *bcl2fastq* following Illumina's manual

   (c) Run fastQC to confirm the quality of the fastq files

3. In a new working folder:

   (a) Create the subfolder "fastq", and copy all the demultiplexed fastq.gz files. Ensure that all file names begin with the well coordinate (e.g. A01) followed by an underscore

   (b) Create a tab separated txt file called "sci-RNA-seq-8.RT.oligos" with the association between RT wells and RT barcode sequences (refer to Appendix Table SP2.1)

   ```
   A01 TTCTCGCATG
   ...
   ```

   (c) Create the subfolder "GENOMES", and copy the annotated genome (i.e., GRCh38)

4. Run *catcheR_scicount*:

   ```
   catcheR_scicount(
       group=c("docker","sudo"),
       folder,
       sample.name,
       UMI.cutoff)
   ```

   catcheR_scicount arguments:

   (a) group: string with two options: sudo or docker, depending on the user group (info)

   (b) folder: string with the working folder path

   (c) sample.name: string with the name of the experiment

   (d) UMI.cutoff: integer of the minimum number of UMI per nucleus needed to consider the single cell transcriptome valid

   Example usage:

   ```
   catcheR_scicount(
       group = "docker",
       folder = "path/to/file",
       sample.name = "experiment",
       UMI.cutoff = 500)
   ```

   catcheR_scicount outputs, found in the final-output folder: knee-plot of UMI per cell (Figure EV1H), statistics files, sparse cell-by-gene count matrix, dense gene matrix "exp_mat.csv" and "exp_mat_no0.csv" (filtered to exclude genes with 0 counts in all cells), also in Rdata format

After obtaining the count matrix, proceed to iPS2-sci-seq perturbation deconvolution.

iPS2-10X-seq perturbation deconvolution

shRNA perturbations can be assigned to single cells using *catcheR_10Xcatch*, which identifies NGS reads containing UCI-BCs, matches them to the corresponding transcriptome based on their shared cellular barcodes, applies filters for background noise, and selects cells with robust evidence of a single integration. UCI-BC filtering involves: (1) filtering out UCI-BCs supported by less than a certain number of UMIs in a given transcriptome, to account for PCR and sequencing artifacts; (2) filtering out UCI-BCs that represent less than a certain fraction of all UMIs for UCI-BCs in a given transcriptome, to reduce noise arising from free mRNA; and (3) filtering out UCI-BCs whose UMI fraction in a given transcriptome is not several fold greater than the second most common UCI-BC, to eliminate cells that contain multiple UCI-BCs when the second most common one falls just below one of the first two thresholds. The resulting list of *bona fide* shRNA integrations per cell is then used to filter those expressing a single shRNA.

This section outlines a typical analysis workflow for iPS2-10X-seq, iPS2-CITE-seq or iPS2-multi-seq experiments, which follow analogous preprocessing and count matrix structures based on 10X Genomics technology. The pipeline begins with *catcheR_10Xcatch*, which performs a full analysis using automatic thresholding. If needed, these thresholds can be subsequently refined with *catcheR_10XcatchQC* and applied for re-filtering using *catcheR_filtercatch*. Finally, *catcheR_10Xnocatch* allows incorporation of cells that do not express any shRNA as additional negative controls in the final annotated cell-by-gene matrix.

1. In a new working folder:
   (a) Copy the fastq/fq or fastq.gz files with demultiplexed read 1 and read 2 of the UCI-BC library (step 3 of Obtain iPS2-10X-seq, iPS2-CITE-seq, or iPS2-multi-seq count matrices)
   (b) Copy the cell-by-gene count matrix in CSV format (step 6 of Obtain iPS2-10X-seq, iPS2-CITE-seq, or iPS2-multi-seq count matrices)
   (c) Create a CSV file named "rc_barcodes_genes.csv" with two columns: (1) the shRNA BCs; (2) the matching shRNA names in the format "GENE.shRNAID"

   ```
   CAAGAGCC,SMAD2.1
   ...
   ```

2. Run *catcheR_10Xcatch* to execute the complete analysis with automatic thresholding:

```
catcheR_10Xcatch(
    group=c("docker","sudo"),
    folder,
    fastq.read1,
    fastq.read2,
    expression.matrix,
    reference = "GGCGCGTTCATCTGGGGGAGCCG",
    UCI.length = 6,
    threads = 2,
    percentage = 15,
    mode = "bimodal",
    ratio = 5,
    samples = 1,
    x = 100,
    y = 400)
```

catcheR_10Xcatch arguments:

(a) group: string with two options: sudo or docker, depending on the user group (info)
(b) folder: string with the working folder path
(c) fastq.read1: string with the filename of read 1 fastq/fq or fastq.gz (step 1a)
(d) fastq.read2: string with the filename of read 2 fastq/fq or fastq.gz (step 1a)
(e) expression.matrix: string with the filename of the count matrix file CSV (step 1b)
(f) reference: string with the reverse complement of the sequence before the shRNA BC at the start of read 2 (default is the 3' end of the OPTtetR cDNA, optional argument; Figure EV2F)
(g) UCI.length: integer of the UCI length (default is 6, optional argument)
(h) threads: integer of the threads to be used for parallelization (default is 2, optional argument)
(i) percentage: integer of the percentage of UMIs supporting a given UCI over the total UMIs supporting all UCIs in a given cell; it is used as threshold to consider the UCI valid (Figure SP4.4D); the recommended default is 15 (optional argument)
(j) mode: string with two options: bimodal or noise, defining the mode for automatic thresholding the minimum number of UMIs per UCI to consider the UCI valid (optional argument)
    i. "bimodal" (default) sets the threshold at the valley of the bimodal UMIxUCI distribution (Figure SP4.4C)
    ii. "noise" sets the threshold at 1.35 * the number of UCIs supported by a single UMI; used if the UMIxUCI distribution is not bimodal
(k) ratio: the ratio between the number of UMIs supporting the most represented UCI and the number of UMIs supporting the second most represented UCI. The parameter is needed to identify the cell as a single integration cell. The default is 5 (optional argument)
(l) samples: the number of samples present in the same experiment (cells from different experiments will have the corresponding number after the cell ID in the gene expression matrix, so that multiple reactions of the same experiment can be unified). The default is 1 (optional argument)
(m) x: an integer indicating the upper limit on the x-axis of the cropped version of the plot "UMIxUCI". Default is 100 (optional argument)
(n) y: an integer indicating the upper limit on the y axis of the cropped version of the plot "UMIxUCI". Default is 400 (optional argument)

Example usage:

```
catcheR_10Xcatch(group = "docker",
    folder = "path/to/folder",
    fastq.read1 = "R1.fastq.gz",
    fastq.read2 = "R2.fastq.gz",
    expression.matrix = "filename.csv",
    threads = 12)
```

catcheR_10Xcatch key outputs (will be stored inside the ”Result” folder of each sample):

(a) ”log.txt” with the starting number of reads

(b) ”log2.txt” with the number of cells, UMIs, UCIs, and the calculated thresholds of UMI per UCI for both ”bimodal” and ”noise” (only one is chosen based on the ”mode” argument; the other one is provided for a potential reanalysis)

(c) Bar charts of UMI counts per shRNA and target gene (Figure SP4.4A and Figure SP4.4B)

(d) Frequency histograms of UCIs supported by a certain number of UMIs (UMIxUCI), to interpret the signal/noise of the experiment and possibly set a custom UMIxUCI threshold for subsequent reanalysis (Figure SP4.4C)

    Note: here and below, copies of the same UCI expressed by different cells are plotted and analyzed separately

(e) Frequency histogram of UCIs supported by a certain fraction of UMIs over the total number of UMIs supporting all UCIs in a given cell (UMIpercentagexUCI), to further assess signal/noise and possibly adjust the default threshold (Figure SP4.4D

(f) Dot plots that combine the UMIpercentagexUCI and UMIxUCI data on the x and y axes, respectively, with each dot representing one or more UCI (quantified by the dot size). Dot colors indicate either the number of valid UCIs (i.e., shRNAs) in the cell containing a given UCI (Figure SP4.4E), or whether said UCI is the only valid one in the relevant cell and is thus assigned to such cell (Figure SP4.4F)

(g) ”log_part3.txt” with how many cells were assigned to a single shRNA or were filtered out due to zero or multiple shRNAs

(h) ”silencing_matrix.csv” is the key output used for the secondary analyses: the cell-by-gene count matrix provided as input, filtered and annotated with the shRNA encoded in each cell. It is also provided in RDS format for easy loading into R. Cell names are modified as follows:

    `cellID_UMIxUCI_BC_GENE_UCI`

    Where:

    i. cellID is the original cell name (i.e., the 16 bp of the 10X RT barcode)
    ii. UMIsxUCI is the number of UMI associated with that perturbation
    iii. BC is the shRNA barcode
    iv. GENE is the shRNA target
    v. UCI is the Unique Clonal Identifier (shared by all cells originating from the same hPSC clone)

    Example of a cell name after the analysis:

    `TTCTAACCACAGTCGC_180_CGTGATGC_NKX2.5_ACAGTG`

The annotated matrix can be directly used with the functions implemented in *catcheR* (see Perturbation effect analysis) to perform a range of secondary analyses described in the manuscript. Additional scripts are available at our GitHub repository for customized or extended analyses, most of which are now incorporated into *catcheR*.

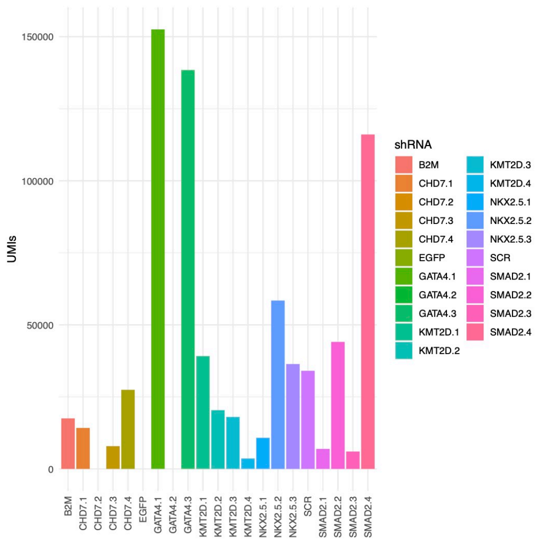

(A)

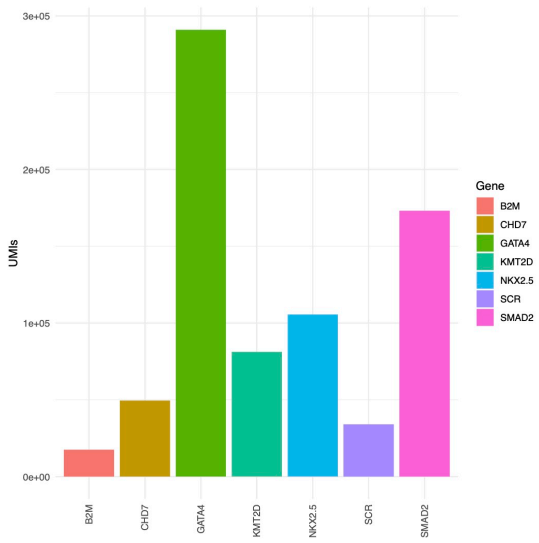

(B)

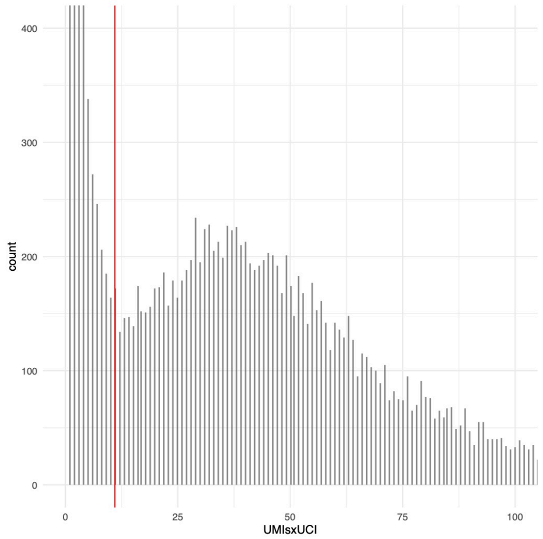

(C)

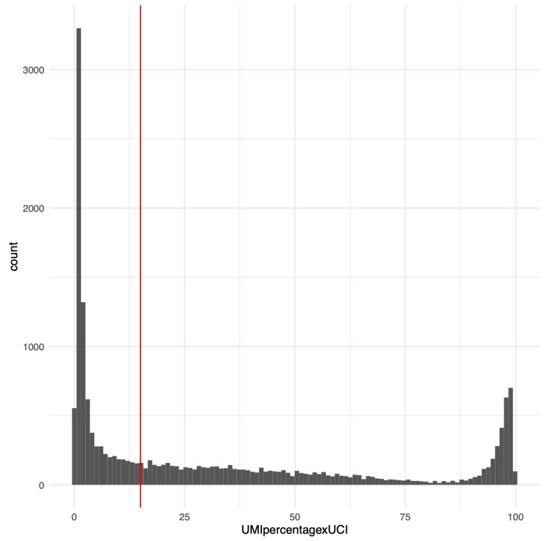

(D)

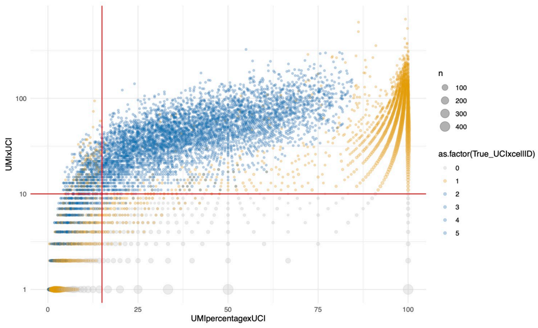

(E)

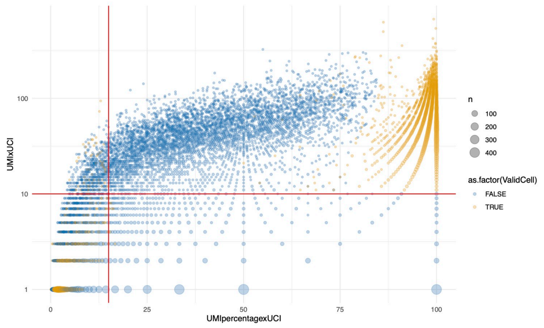

(F)

Figure SP4.4. Exemplary key outputs of a *catcheR_10Xcatch* analysis: ”barcode_distribution.pdf” (A); ”gene_distribution.pdf” (B); ”UMIxUCI_400_100.pdf” with the automatic UMIxUCI threshold based on ”bimodal” (C); ”percentage_of_UMIxUCI_dist.pdf” with the default 15% ”percentage” threshold (D); ”2D_percentage_of_UMIxUCI_UMI_count_trueorfalse.pdf” (E); and ”2D_percentage_of_UMIxUCI_UMI_ValidCells.pdf” (F).

OPTIONAL: fine tune iPS2-10X-seq perturbation assignment

1. In the same working folder used to run *catcheR_10Xcatch*, run *catcheR_10XcatchQC*, which uses the txt file outputs to regenerate the quality control plots described in Figure SP4.4 and suggest new thresholds to enable subsequent filtering

```
catcheR_10XcatchQC(
    group=c("docker","sudo"),
    folder,
    reference = "GGCGCGTTCATCTGGGGGAGCCG",
    mode = "bimodal",
    sample = 1
    x = 100,
    y = 400)
```

Example usage:

```
catcheR_10XcatchQC(
    group = "docker",
    folder = "path/to/working/folder",
    mode = "noise")
```

The function is to be run separately for each sample (specified by argument sample with default 1). *catcheR_10XcatchQC* arguments and outputs are the same as *catcheR_10Xcatch*

2. In the same working folder used to run *catcheR_10Xcatch*, run *catcheR_filtercatch*, which uses the output of *catcheR_10Xcatch* to filter cells based on custom thresholds

```
catcheR_filtercatch(
    group=c("docker","sudo"),
    folder,
    expression.matrix,
    UMI.count,
    percentage = 15,
    ratio = 5,
    sample = 1)
```

Example usage:

```
catcheR_filtercatch(group = "docker",
    folder = "path/to/working/folder",
    expression.matrix = "filename.csv",
    UMI.count = 5,
    sample = 1)
```

The function is to be run separately for each sample (specified by argument sample with default 1). *catcheR_filtercatch* argument *percentage* is the minimum percentage of UMI for a given UCI over the total UMIs of UCIs in that cell, to consider the UCI valid. *UMI.count* is an integer of the custom UMI threshold. *ratio* is the minimum ratio between UMIs of top UCI and UMIs of 2nd top UCI. The other arguments and the outputs are the same as *catcheR_10Xcatch*

OPTIONAL: identify cells expressing no shRNA in iPS2-10X-seq

1. In the same working folder used to run *catcheR_10Xcatch*, run *catcheR_10Xnocatch*:

```
catcheR_10Xnocatch(
    group=c("docker","sudo"),
    folder,
    expression.matrix,
    threshold,
    sample = 1,
    reference = "TACGCGTTCATCTGGGGGAGCCG")
```

Example usage:

```
catcheR_10Xnocatch(group = "docker",
folder = "path/to/folder",
expression.matrix = "filename.csv",
threshold = 5)
```

catcheR_10Xnocatch argument *threshold* is an integer of the minimal UMI count for the ”empty” reference (Figure EV2F) needed to confidently identify it as having integrated said plasmid. All other arguments are the same as *catcheR_10Xcatch*

catcheR_10Xnocatch output is an updated ”silencing_matrix.csv”, also available in RDS format, in which names of empty cells are modified as ”cellID_?_empty_NA_empty”

OPTIONAL: merge multiple samples

1. In case fine-tuning is applied after *catcheR_10Xcatch* and multiple samples are present, run *catcherR_merge* to aggregate the results in a single matrix. This step is done automatically when running *catcheR_10Xcatch*

```
catcheR_merge(
    group=c("docker","sudo"),
    folder,
    samples = 2,
    empty = T
    )
```

The argument ”empty” determines whether the cells identified by *catcheR_nocatch* should be added to the matrix

Example usage:

```
catcheR_merge(group = "docker",
folder = "path/to/folder",
samples = 4)
```

iPS2-sci-seq perturbation deconvolution

This section describes a typical analysis pipeline similar to the one described in the previous section, but leveraging on the *catcheR_scicatch*, *catcheR_scicatchQC*, *catcheR_filtercatch*, and *catcheR_scinocatch* functions.

1. In a new working folder:
   (a) Create a subfolder called ”fastq”, and copy all the demultiplexed fastq.gz files (step 2 of Obtain iPS2-sci-seq count matrices). Ensure that all file names begin with the well coordinate (e.g. A01)
   (b) Copy the cell-by-gene expression matrix CSV file obtained with *catcheR_scicount* function (step 4 of Obtain iPS2-sci-seq count matrices)
   (c) Create a CSV file called “rc_barcodes_genes.csv” with two columns: (1) the shRNA BCs; (2) the matching shRNA names in the format ”GENE.shRNAID”

   ```
   CAAGAGCC,SMAD2.1
   ...
   ```

   (d) Copy the txt file ”sci-RNA-seq-8.RT.oligos” used by *catcheR_scicount* (step 3b of Obtain iPS2-sci-seq count matrices)

2. Run *catcheR_scicatch* to execute the complete analysis with automatic thresholding; the arguments are the same as for *catcheR_10Xcatch* (step 2 of iPS2-10X-seq perturbation deconvolution), except that no filenames are provided since fastq files must be in the ”fastq” subfolder in the working directory:

   ```
   catcheR_scicatch(
       group=c("docker","sudo"),
       folder,
       expression.matrix,
       reference = "GGCGCGTTCATCTGGGGGAGCCG",
       UCI.length = 6,
       threads = 2,
       percentage = 15,
       ratio = 5,
       mode = "bimodal",
       x = 100,
       y = 400)
   ```

   Example usage:

   ```
   catcheR_scicatch(group = "docker",
       folder = "path/to/working/folder",
       expression.matrix = "filename.csv",
       threads = 12)
   ```

   catcheR_scicatch key outputs are the same as for *catcheR_10Xcatch* (step 2 of iPS2-10X-seq perturbation deconvolution and Figure SP4.4), except that in ”silencing_matrix.csv” ”cell ID” indicates the PCR well and RT barcode ID (which can be leveraged to identify nuclei from different samples pooled on the same RT plate)

   Example of cell name after the analysis:

   ```
   P24__RT_27_7_GCCTGTGT_SCR_ACGGTC
   ```

   In addition, *catcheR_scicatch* provides two quality control outputs to evaluate experimental biases during sci-RNA-seq library preparation: *demux* and *RT* detail about how many cells were identified from each row and column of the PCR and RT plates, respectively

OPTIONAL: fine tune iPS2-sci-seq perturbation assignment

1. Run *catcheR_scicatchQC* following the steps described for *catcheR_10XcatchQC* in OPTIONAL: fine tune iPS2-10X-seq perturbation assignment: the functions share the same arguments and outputs, but *catcheR_scicatchQC* used the output of *catcheR_scicatch*

   Example usage:

   ```
   catcheR_scicatchQC(
       group = "docker",
       folder = "path/to/working/folder",
       mode = "noise")
   ```

2. Run *catcheR_filtercatch* as described in OPTIONAL: fine tune iPS2-10X-seq perturbation assignment: this function also works on the output of *catcheR_scicatch* or *_scicatchQC*

OPTIONAL: identify cells expressing no shRNA in iPS2-sci-seq

1. Run *catcheR_scinocatch* following the steps described for *catcheR_10Xnocatch* in OPTIONAL: identify cells expressing no shRNA in iPS2-10X-seq: the functions share the same arguments and outputs, but *catcheR_scinocatch* uses the output of *catcheR_scicatch*

   Example usage:

```
catcheR_scinocatch(group = "docker",
    folder = "path/to/working/folder",
    expression.matrix = "filename.csv",
    threshold = 5,
    reference = "TACGCGTTCATCTGGGGGAG")
```

Barcode reassignment

catcheR_sortcatch is an optional function that corrects the annotated cell-by-gene count matrix obtained with *catcheR_10Xcatch* or *catcheR_scicatch* reassigning the perturbation of any cell belonging to a hPSC clone with reliable evidence of a shRNA-barcode swap, based on the results of *catcheR_step1QC*.

1. In a new working folder:
   (a) Copy the annotated count matrix (i.e., ”silencing_matrix.csv”)
   (b) Copy the CSV file with the list of UCI-BCs with reliable evidence of a shRNA-barcode swap (i.e., ”reliable_clones_swaps.csv”; output 2a of Pooled cloning step 1 QC)

2. Run *catcheR_sortcatch*:

```
catcheR_sortcatch(
    group=c("sudo","docker"),
    folder,
    expression.matrix,
    swaps)
```

   Example usage:

```
catcheR_sortcatch(
    group = "docker",
    folder = "path/to/working/folder",
    expression.matrix = "silencing_matrix.csv",
    swaps = "reliable_clones_swaps.csv")
```

   catcheR_sortcatch arguments:

   (a) group: string with two options: sudo or docker, depending on the user group (info)
   (b) folder: string with the working folder path
   (c) expression.matrix: string with the filename of the annotated count matrix CSV
   (d) swaps: a character string with the filename of the txt file (step 1b)

   catcheR_sortcatch output is an updated annotated gene expression matrix CSV file called ”silencing_matrix_updated.csv”, in which ”BC” and ”GENE” have been modified to reflect the actual shRNA encoded in each cell

Perturbation effect analysis

The second part of *catcheR* provides an exploratory analysis with visual and statistical summaries to highlight perturbation effects.

Annotation

Before proceeding, the gene expression matrix should be annotated with gene symbols using the *scannobyGtf* function from the R package rCASC. As part of quality control, we recommend evaluating the fraction of ribosomal and mitochondrial reads — e.g., using the *mitoRiboUmi* function from the same package — and considering the exclusion of cells with abnormally high proportions, which may indicate poor quality or stress.
Note: After this step, row names of the matrix (the genes) will have the following format:

```
GeneSymbol:EnsemblID
```

Example:

```
ENSG00000000003:TSPAN6
```

Data loading

This step loads data from *catcheR_10Xcatch* or *catcheR_scicatch* into a Monocle object after GTF-based annotation, adds experimental design, and performs normalization and clustering.

1. In a new working folder:
   (a) Copy the count matrix annotated with gene names during the previous step (filtered_annotated-_silencing_matrix_complete_all_samples.csv)
   (b) Copy the file rc_barcodes_genes.csv described in step 1a of Pooled cloning step 2 and hiPSC genome editing QC
   (c) Create a new-line separated file listing the control genes (e.g. SCR, *B2M*)
   (d) Create a new-line separated file listing the control samples, if any (e.g. 1,3). These are the sample names also used by *aggr* (see CSV file used as input for *aggr*)
   (e) Create a plain text file listing the sample replicate labels, one per line (e.g., `batch1`, `batch1`, `batch2`, `batch2`). The order of entries must match the order of samples in the input matrix. This file is required for downstream batch-aware analyses. If the dataset includes samples from different experiments or processing batches, batch correction is recommended
   (f) Create a CSV file listing each sample along with its corresponding annotation name, which will be used for display instead of the sample number (this file is mandatory). Example of CSV file to download on GitHub
   (g) Create a newline-separated file listing the genes of interest whose expression you wish to visualize on the UMAP (optional)

2. Run *catcheR_load*:

```
catcheR_load(
  group="docker",
  folder,
  expression.matrix,
  control_genes,
  control_samples = NULL,
  replicates = NULL,
  sample_names,
  resolution = 8e-4,
  genes = NULL)
```

Example usage:

```
catcheR_load(
  group="docker",
  folder="/path/to/working/folder/",
  expression.matrix =
   "annotated_silencing_matrix_complete_all_samples.csv",
  control_genes =  "controls.txt",
  control_samples = "noTET.txt",
  replicates = "replicates.txt",
  sample_names = "samples.csv",
  resolution = 8e-4,
  genes = "genelist.txt")
```

The argument *resolution* refers to the resolution parameter used by Monocle's *cluster_cells* function for clustering

catcheR_load outputs:

1. expression_data.csv and cell_metadata.csv, which can be used to create a Monocle Cell Data Set (CDS) and are also included in the ready-to-load R object starting_cds.Rdata
2. UMAP.pdf plots the dimensionality reduction and UMAP_gene_expression.pdf shows the gene expression on the UMAP of the genes provided by the argument "genes"
3. UMAP_clustering.pdf shows the clustering obtained on the UMAP with the provided resolution
4. processed_cds.RData is the Cell Data Set after normalization, dimensionality reduction, clustering and calculation of trajectories

At the end of this step, the data are stored in a format compatible with the `monocle3` package, which will be used for all the following steps of the standard iPS2-seq perturbation analysis with *catcheR*. However, you can switch to other single-cell analysis frameworks such as `Seurat` or `Scanpy` at any point in the workflow.

```
library(SeuratWrappers)
library(Seurat)
seurat <- as.Seurat(cds, assay = NULL)
scanpy_sce <- as.SingleCellExperiment(seurat)
```

The follow up analysis are: *catcheR_pseudotime*, *catcheR_modules* and *catcheR_enrichment*.

1. With the Pseudotime function, *catcheR* calculates the cumulative frequency of cells sharing the same gene, shRNA, or clone, compared to each control, along the pseudotime trajectory. *catcheR* can compare the cumulative frequency curves by computing directional Kolmogorov-Smirnov statistics between the target and control
2. With the Genes modules function, *catcheR* identifies gene modules within the dataset and computes the cumulative frequency of cells with the same gene, shRNA, or clone—relative to each control—based on the expression of each gene module. Also in this case, cumulative frequency curves are compared using a directional Kolmogorov-Smirnov test
3. With the Enrichment / depletion analysis function, *catcheR* calculates the enrichment of cells with the same gene, shRNA, or clone within clusters, in comparison to each control. *catcheR* can compare each target and control quantity and obtain statistics using the Fisher exact test

Pseudotime

Since Monocle pseudotime calculation requires the user to select the starting point interactively, the first step of the pseudotime analysis needs to be done separately within *monocle3*.

1. After loading the processed_cds.RData in R, calculate the pseudotime trajectory and plot it with the R script below:

   ```
   library(monocle3)
   cds <- order_cells(cds)
   pt = as.data.frame(pseudotime(cds))
   names(pt) = c("pseudotime")
   write.csv(pt, paste0(dir, "/pseudotime.csv"))

   plot_cells(cds,
              color_cells_by = "pseudotime",
              label_cell_groups=FALSE,
              label_leaves=FALSE,
              label_branch_points=FALSE,
              graph_label_size=1.5)
   ```

2. The working folder will contain the output of *catcheR_load* and the CSV file obtained after running the monocle pseudotime function (e.g. "pseudotime.csv")

3. Run *catcheR_pseudotime*:

   ```
   catcheR_pseudotime(
       group=c("docker","sudo"),
       folder,
       cds,
       pseudotime,
       all = FALSE)
   ```

   Example usage:

   ```
   catcheR_pseudotime(
       group="docker",
       folder="/path/to/working/folder/",
       cds = "processed_cds.RData",
       pseudotime = "pseudotime.csv")
   ```

   Argument "all" is a logical operator indicating whether to perform the Kolmogorov-Smirnov test against the controls together (all = F) or against each control separately (all = T). The default argument is false

   Below is a list of the *catcheR_pseudotime* outputs:

   (a) The "cumulative_frequency_pseudotime" plots show the number of cells at each given point of the pseudotime for different groups of cells at different comparison levels - gene, shRNA, and clone

   (b) The "ks_statistics" CSV files that include the Kolmogorov-Smirnov test results comparing cumulative the frequency based on the pseudotime between knockdown and different controls

   (c) The Volcano plots of the results of the Kolmogorov-Smirnov test show the significance and fold change between the pseudotime cumulative curves. Different controls and levels are used

   (d) The "correlated_pseudotime_gene_exp.pdf" showing the expression on the UMAP of the genes most correlated with the pseudotime

Genes modules

This function uses Monocle to find gene modules which expression varies in different perturbation groups, i.e., perturbed gene or shRNA or clones.

1. Run *catcheR_modules*:

   ```
   catcheR_modules(group=c("docker, "sudo"),
       folder,
       cds,
       resolution=1e-2)
   ```

   Example usage:

   ```
   catcheR_modules(group="docker",
       folder="/path/to/working /folder/",
       cds = "processed_cds.RData")
   ```

   The resolution parameter refers to the value used in Monocle's *find_gene_modules* function, which influences the number of gene modules identified and the number of genes included in each module (higher resolution typically results in more, smaller modules)

   Below are listed the outputs of *catcheR_modules*:

   (a) gene_modules.csv, listing the genes present in each module
   (b) Heatmap plots display the Z-scores of each module, either all modules or the top 10 most variable ones, across perturbation groups
   (c) The "modules_cells" folder contains tables with aggregated module expression values for each cell. These can be used as input for *catcheR_pseudotime* in place of the pseudotime CSV file, allowing the analysis of cumulative frequency based on module expression

2. Run *catcheR_pseudotime* on the CSV files stored in the "modules_cells" folder to use the data generated by *catcheR_modules* as described in the previous example in the Pseudotime section

Enrichment / depletion analysis

This function evaluates whether perturbation groups are enriched or depleted in cells (number) or in specific cell subpopulations (clusters).

1. Run *catcheR_enrichment*:

   ```
   catcheR_enrichment(
       group=c("docker","sudo"),
       folder,
       file,
       meta,
       timepoint = "PSC",
       control_gene = "SCR",
       min_cells_cluster = 70,
       min_cells_shRNA = 40)
   ```

Example usage:

```
catcheR_enrichment(group = "docker",
folder ="/path/to/working /folder/"
file = "processed_cds.RData",
meta = "cell_metadata.csv",
timepoint = "PSC",
control_gene = "SCR")
```

The required input is the cds file generated from *catcheR_load*

Timepoint refers to the baseline time point used as a control for statistical analysis. It is required when experiments include multiple time points and aim to assess enrichment or depletion across these time points *control_gene* specifies the control gene used as a reference for statistical comparisons

Below are the outputs of *catcheR_enrichment*:

(a) Plots of cells in each perturbation group

(b) Volcano plot showing enrichment or depletion of cell numbers in perturbation groups compared to the control, based on fold-change ($\log_2$ ratio) and statistical significance. A corresponding bar plot displays the $\log_2$ fold-change for each perturbation

(c) Barplots showing the distribution of cells from different perturbation groups in the clusters

(d) Volcano plot showing the results of Fisher's exact test, comparing the distribution of cells from perturbation groups across Monocle-derived clusters. The plot displays $-\log_{10}$ adjusted p-values *versus* $\log_2$ fold-changes relative to the control group

(e) Table with Fisher's statistics
