# Supplementary material for: Single cell transcriptional perturbome in pluripotent stem cell models
Source: Mol Syst Biol. 2025 Dec 10;22(2):179–227. doi: 10.1038/s44320-025-00172-8 (PMC12864791; doi:10.1038/s44320-025-00172-8)
Supplement: Supplementary file 5 — Source data Fig. 4 [file 44320_2025_172_MOESM5_ESM.zip › Figure4/4B/README.rtf]

Images were exported from the microscope without processing and were arranged by cropping the original picture, as shown. Full-size pictures are in the folders.Panel B CTR (top):in folder B_CTR_TOPMerged, Holechst (DAPI), FOXG1, PAX6 figures in tiff format _before_chop is the full-size figure (original high-resolution microscopic images)_BW is the figure with the signal in black and white and used for the panel figurePanel B TET (bottom):Merged, Holechst (DAPI), FOXG1, PAX6 figures in tiff format _before_chop is the full-size figure (original high-resolution microscopic images)_BW is the figure with the signal in black and white and used for the panel figure
