# Supplementary figures and images for: Single cell transcriptional perturbome in pluripotent stem cell models
Source: Mol Syst Biol. 2025 Dec 10;22(2):179–227. doi: 10.1038/s44320-025-00172-8 (PMC12864791; doi:10.1038/s44320-025-00172-8)

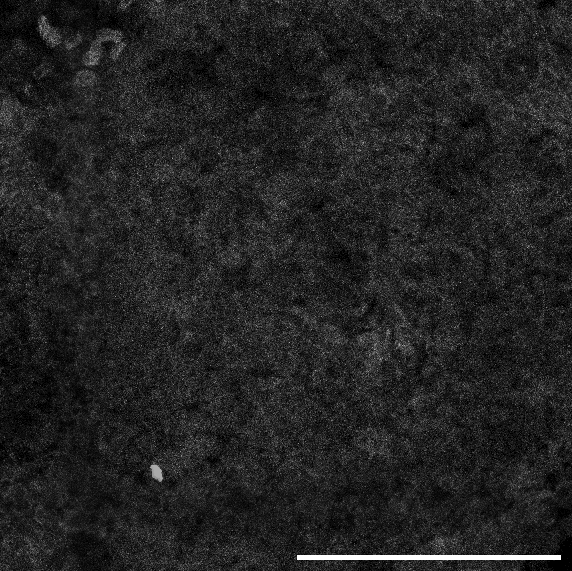

Supplement: Supplementary file 5 — Source data Fig. 4 [file 44320_2025_172_MOESM5_ESM.zip › Figure4/4B/B_TET_BOTTOM/foxg1_BW.tiff]

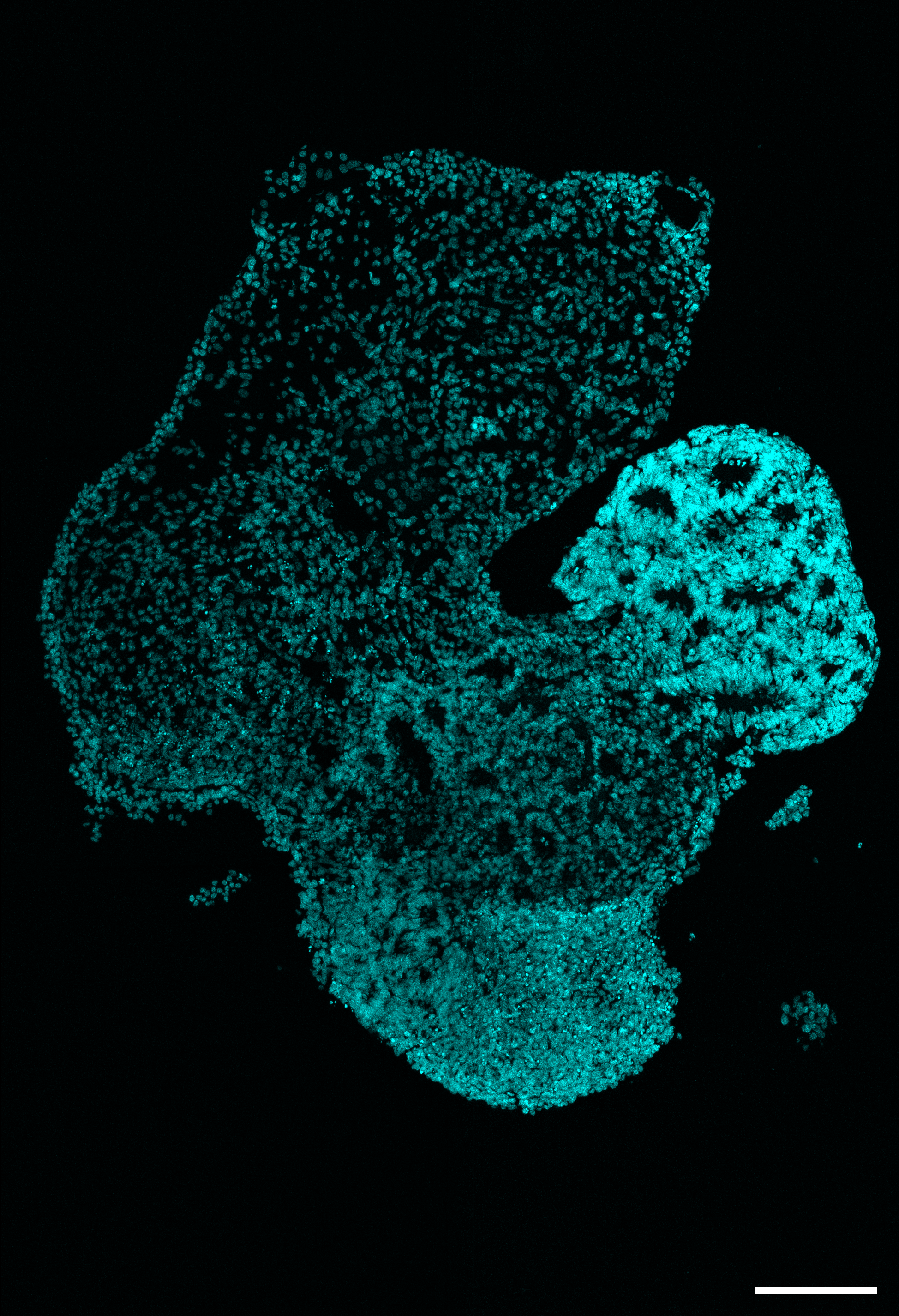

Supplement: Supplementary file 5 — Source data Fig. 4 [file 44320_2025_172_MOESM5_ESM.zip › Figure4/4B/B_TET_BOTTOM/Holechst_before_chrop.tiff]

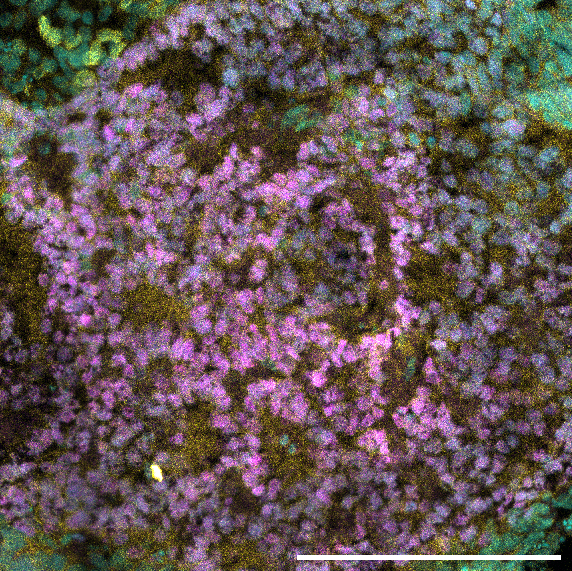

Supplement: Supplementary file 5 — Source data Fig. 4 [file 44320_2025_172_MOESM5_ESM.zip › Figure4/4B/B_TET_BOTTOM/Merged.tiff]

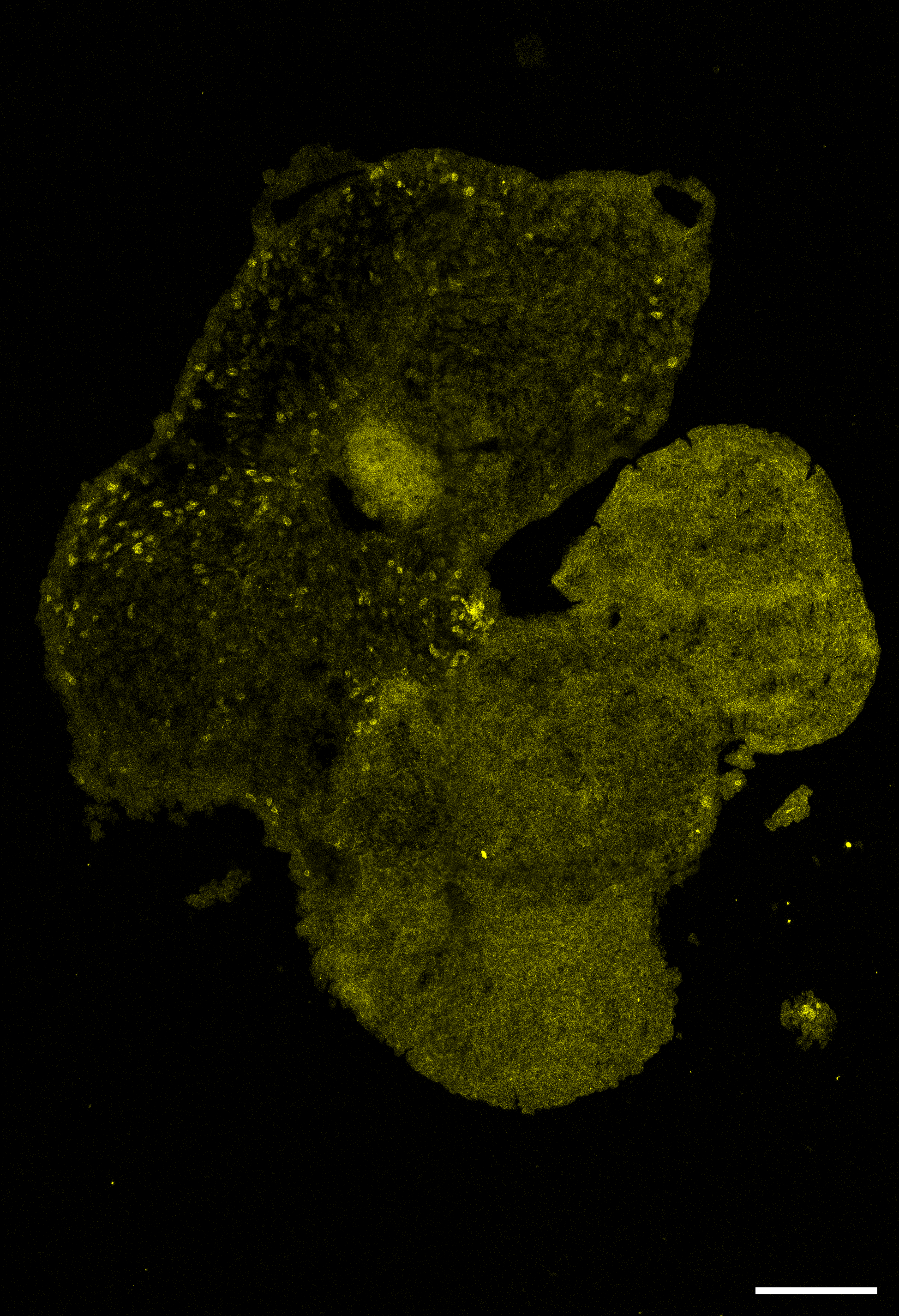

Supplement: Supplementary file 5 — Source data Fig. 4 [file 44320_2025_172_MOESM5_ESM.zip › Figure4/4B/B_TET_BOTTOM/foxg1_before_chrop.tiff]

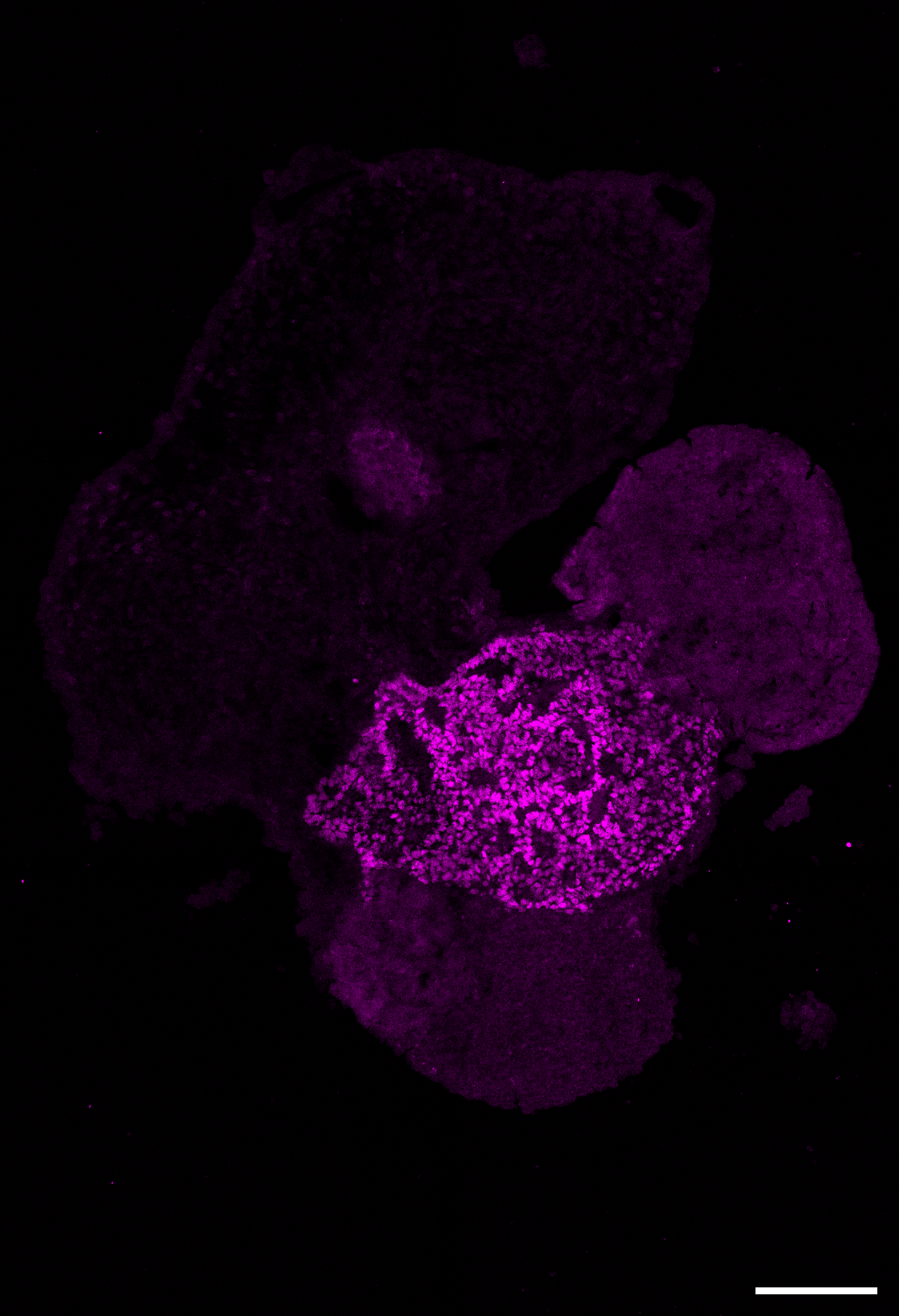

Supplement: Supplementary file 5 — Source data Fig. 4 [file 44320_2025_172_MOESM5_ESM.zip › Figure4/4B/B_TET_BOTTOM/pax6_before_chrop.tiff]

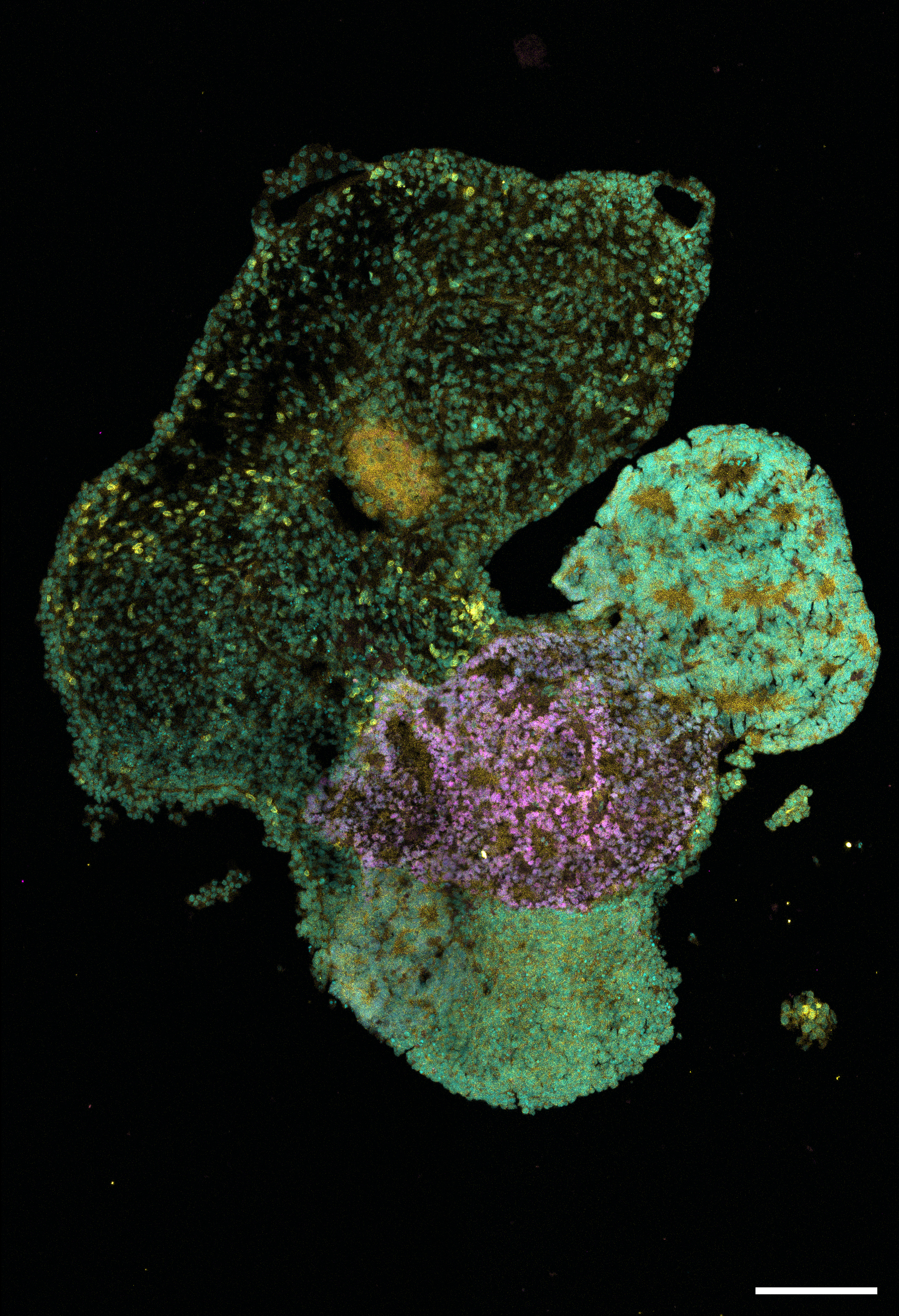

Supplement: Supplementary file 5 — Source data Fig. 4 [file 44320_2025_172_MOESM5_ESM.zip › Figure4/4B/B_TET_BOTTOM/Merged_main_before_chrop.tiff]

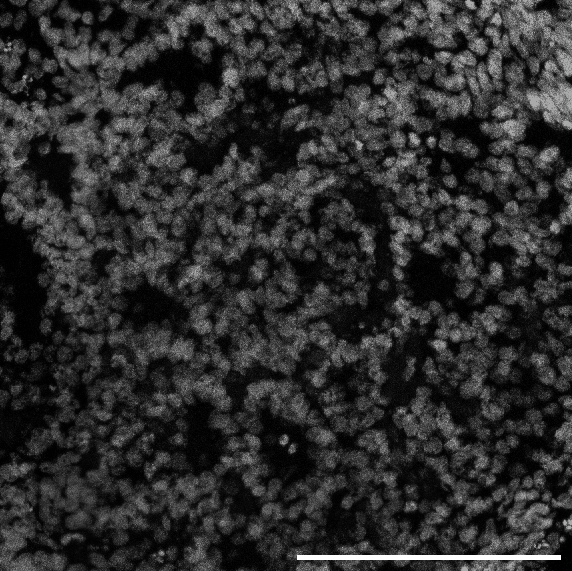

Supplement: Supplementary file 5 — Source data Fig. 4 [file 44320_2025_172_MOESM5_ESM.zip › Figure4/4B/B_TET_BOTTOM/Holechst_BW.tiff]

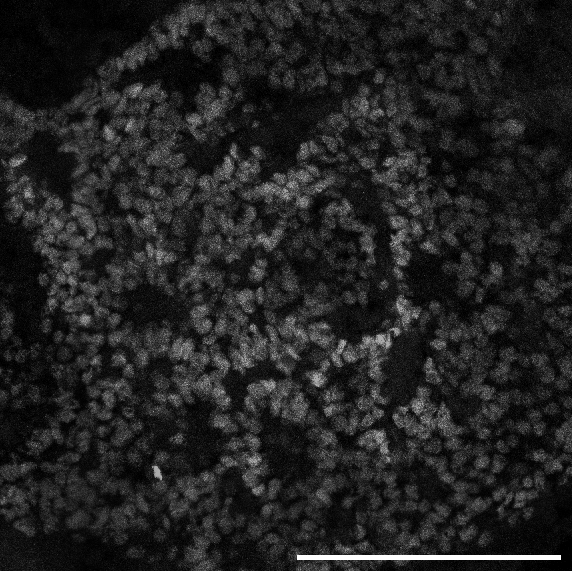

Supplement: Supplementary file 5 — Source data Fig. 4 [file 44320_2025_172_MOESM5_ESM.zip › Figure4/4B/B_TET_BOTTOM/pax6_BW.tiff]

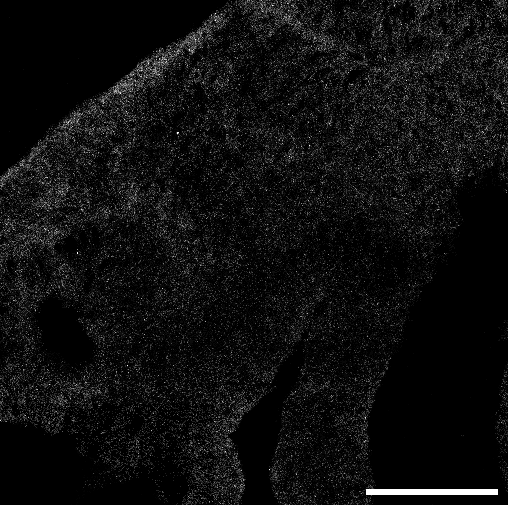

Supplement: Supplementary file 5 — Source data Fig. 4 [file 44320_2025_172_MOESM5_ESM.zip › Figure4/4B/B_CTR_TOP/foxg1_BW.tiff]

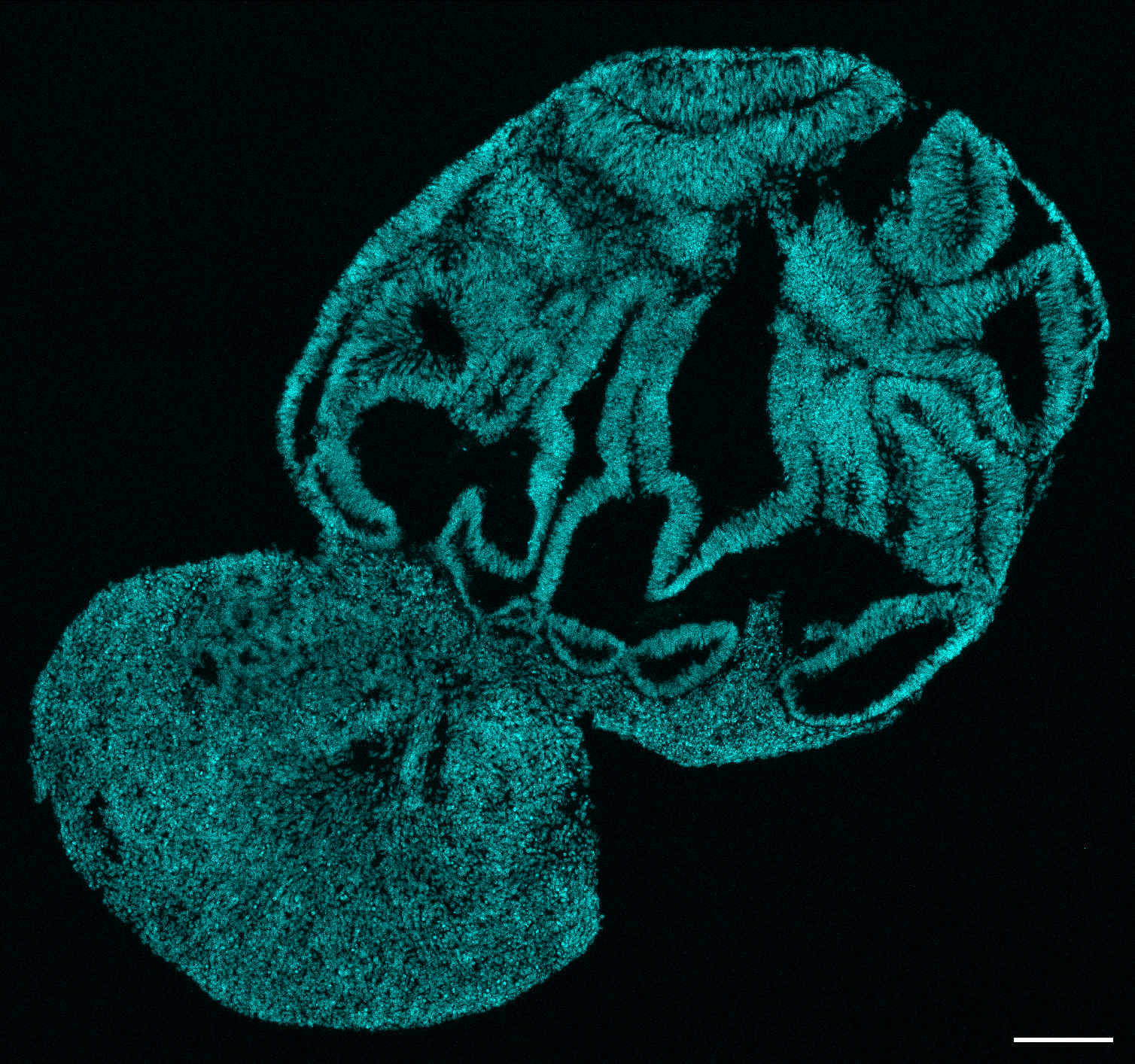

Supplement: Supplementary file 5 — Source data Fig. 4 [file 44320_2025_172_MOESM5_ESM.zip › Figure4/4B/B_CTR_TOP/Holechst_before_chrop.tiff]

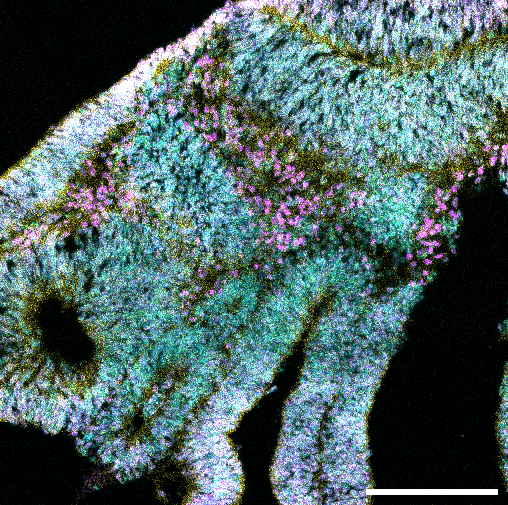

Supplement: Supplementary file 5 — Source data Fig. 4 [file 44320_2025_172_MOESM5_ESM.zip › Figure4/4B/B_CTR_TOP/Merged.tiff]

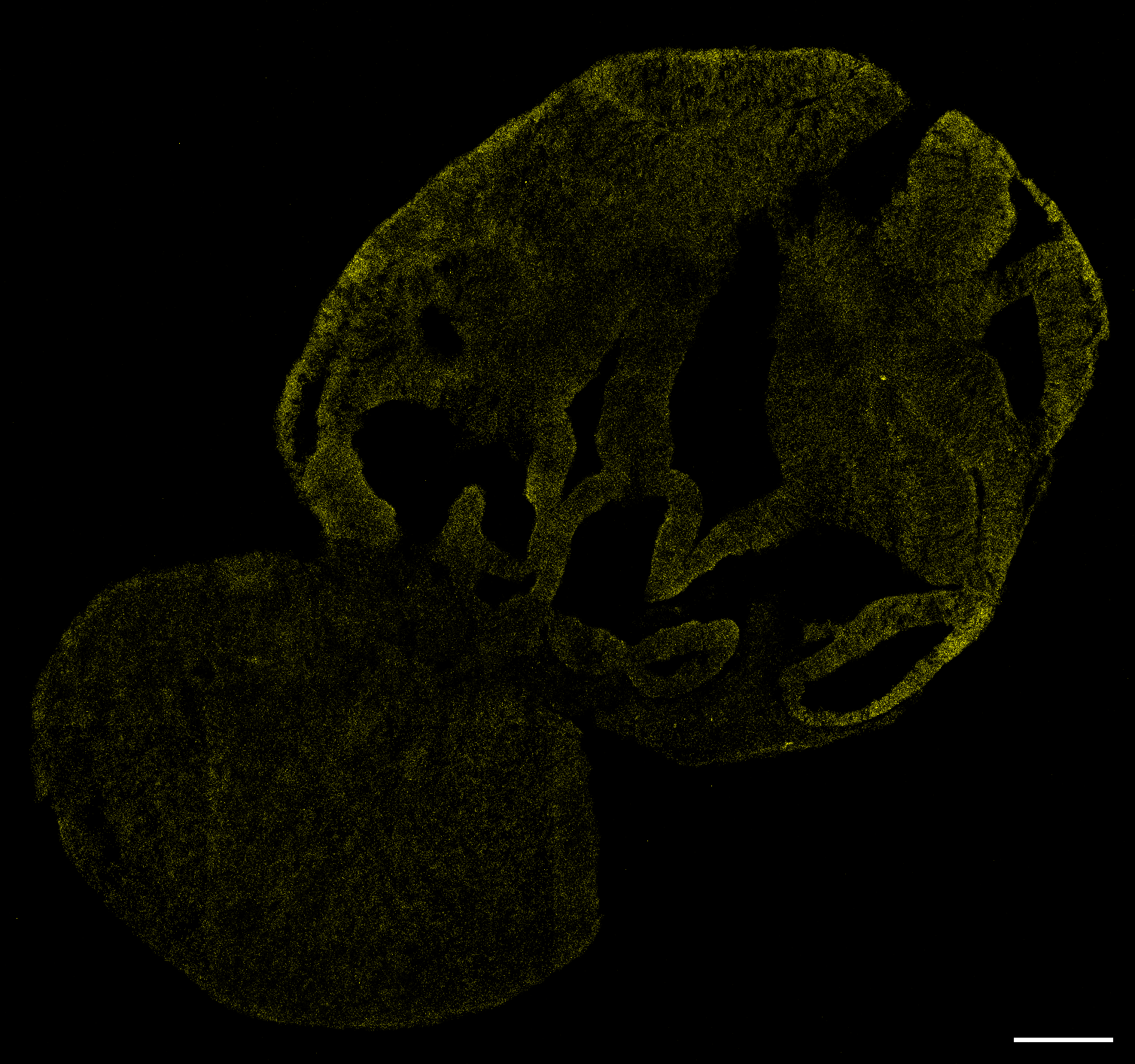

Supplement: Supplementary file 5 — Source data Fig. 4 [file 44320_2025_172_MOESM5_ESM.zip › Figure4/4B/B_CTR_TOP/foxg1_before_chrop.tiff]

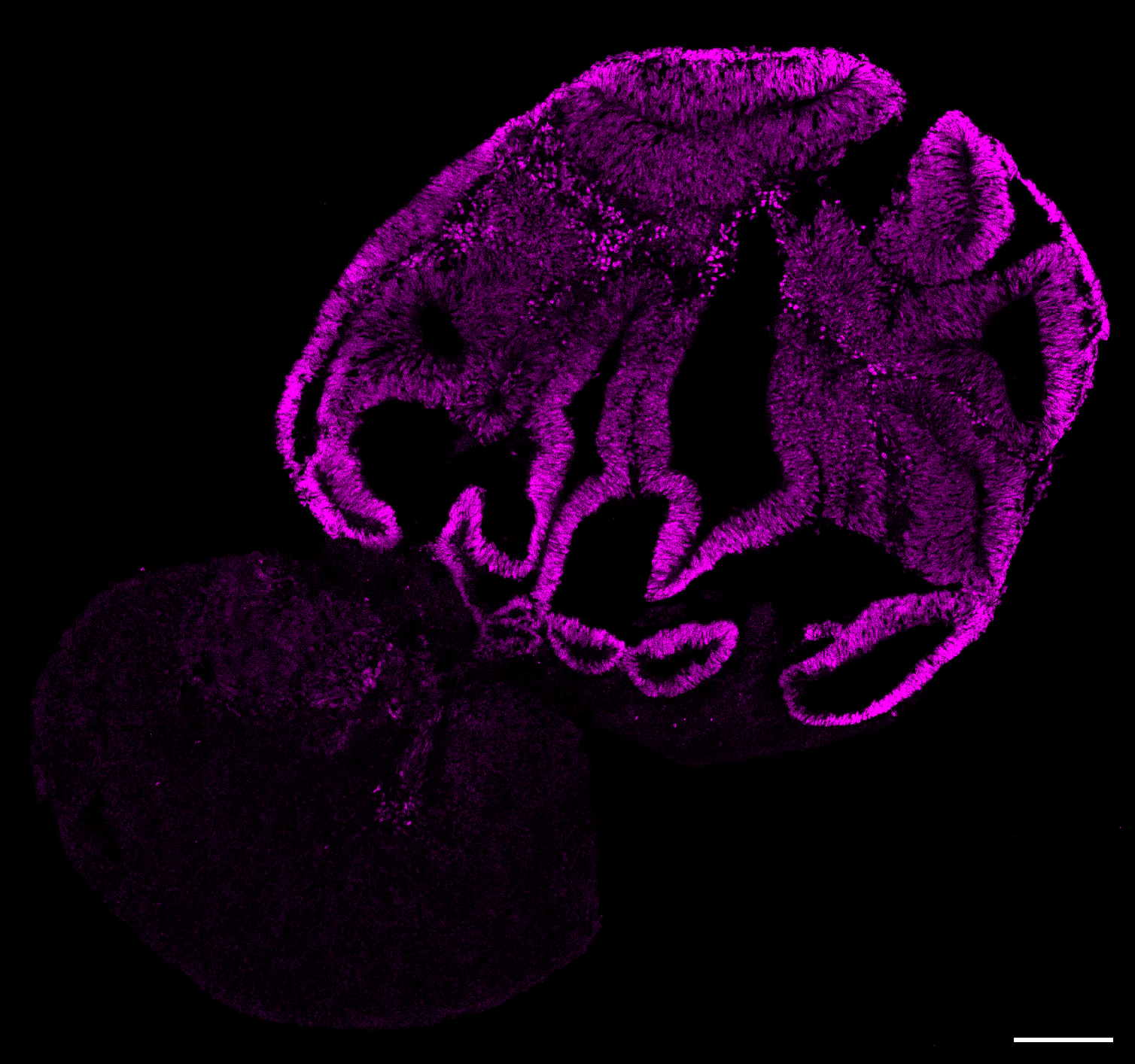

Supplement: Supplementary file 5 — Source data Fig. 4 [file 44320_2025_172_MOESM5_ESM.zip › Figure4/4B/B_CTR_TOP/pax6_before_chrop.tiff]

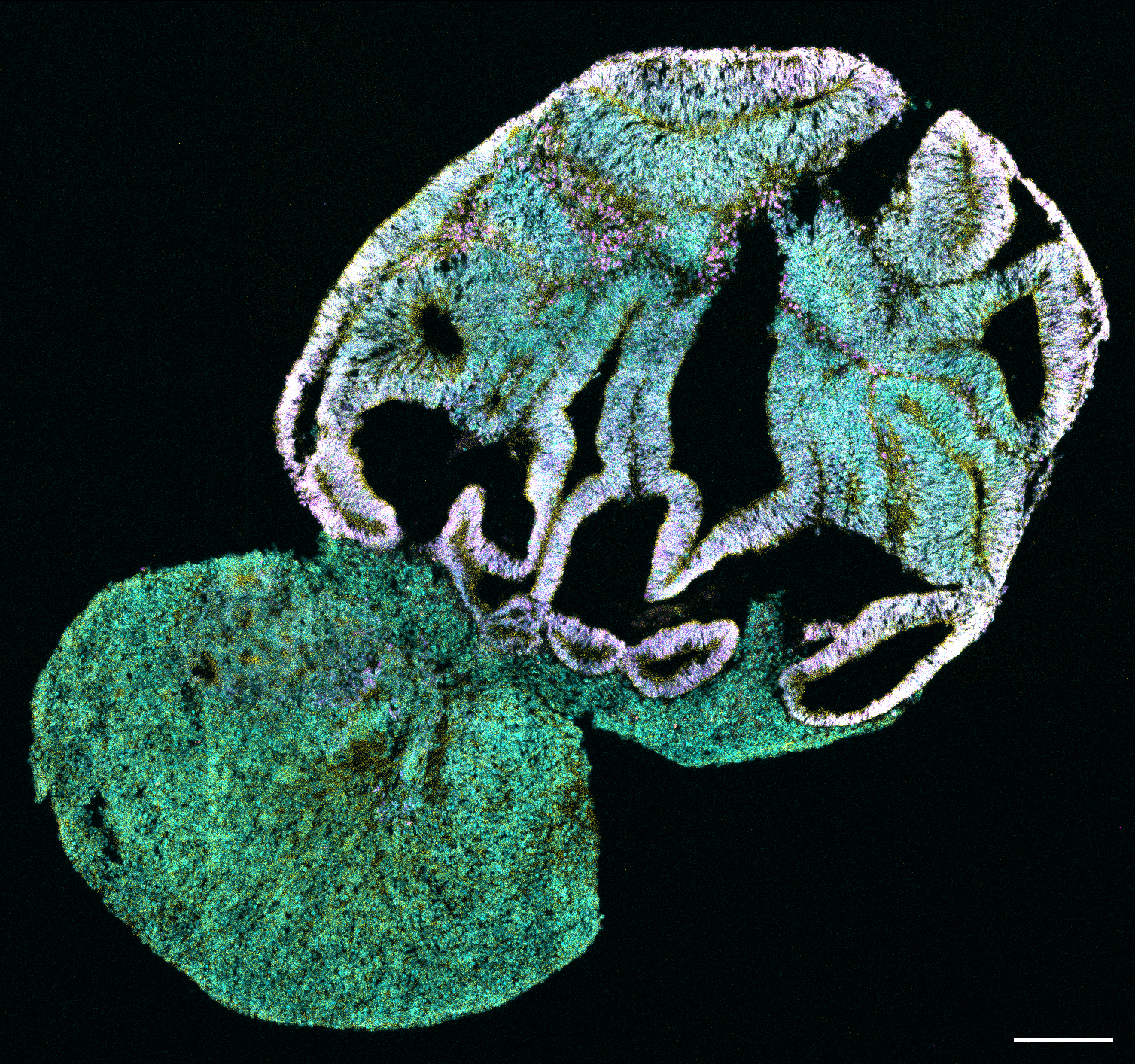

Supplement: Supplementary file 5 — Source data Fig. 4 [file 44320_2025_172_MOESM5_ESM.zip › Figure4/4B/B_CTR_TOP/Merged_main_before_chrop.tiff]

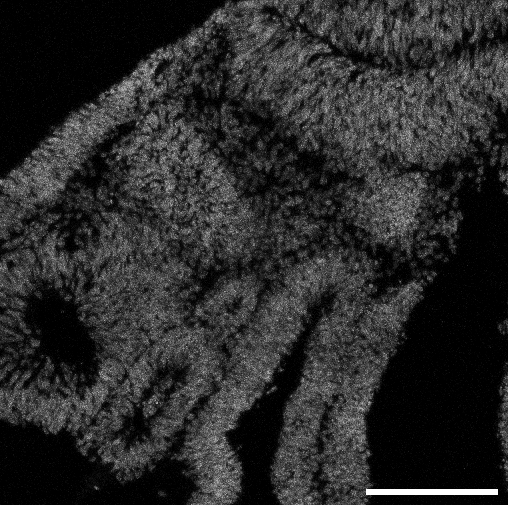

Supplement: Supplementary file 5 — Source data Fig. 4 [file 44320_2025_172_MOESM5_ESM.zip › Figure4/4B/B_CTR_TOP/Holechst_BW.tiff]

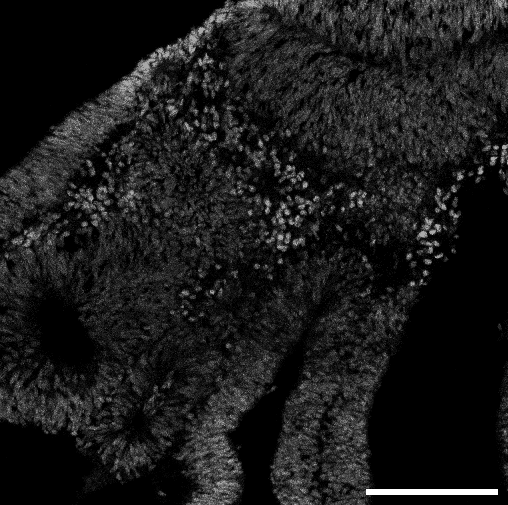

Supplement: Supplementary file 5 — Source data Fig. 4 [file 44320_2025_172_MOESM5_ESM.zip › Figure4/4B/B_CTR_TOP/pax6_BW.tiff]

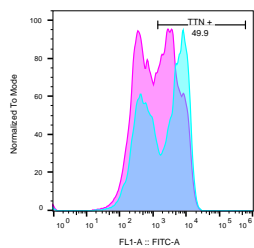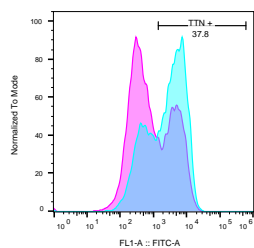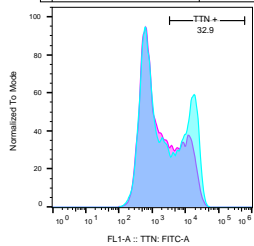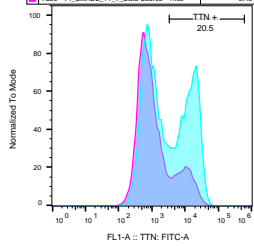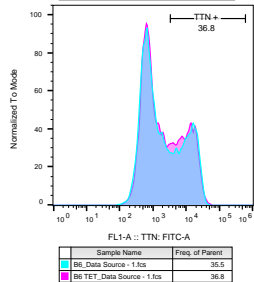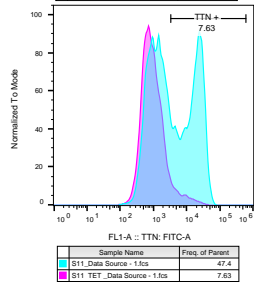

Supplement: Supplementary file 6 — Source data Fig. 6 [file 44320_2025_172_MOESM6_ESM.zip › Figure6/6C_D/HISTOGRAMS_showed_in_6D.pdf]

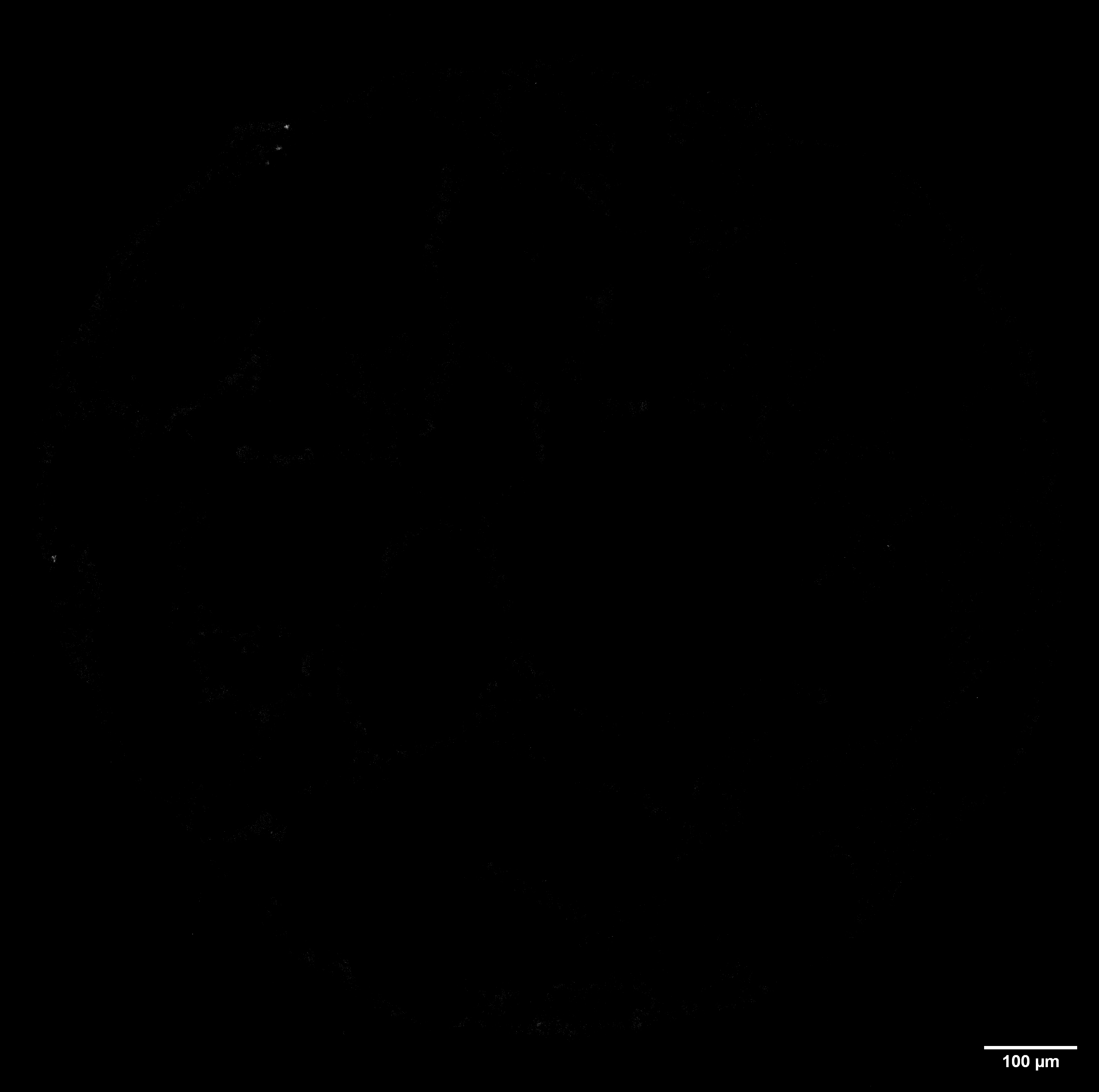

Supplement: Supplementary file 6 — Source data Fig. 6 [file 44320_2025_172_MOESM6_ESM.zip › Figure6/6H/H_SMAD2_CTR_LEFT/top/SMAD_CTR_wt1_BW.tiff]

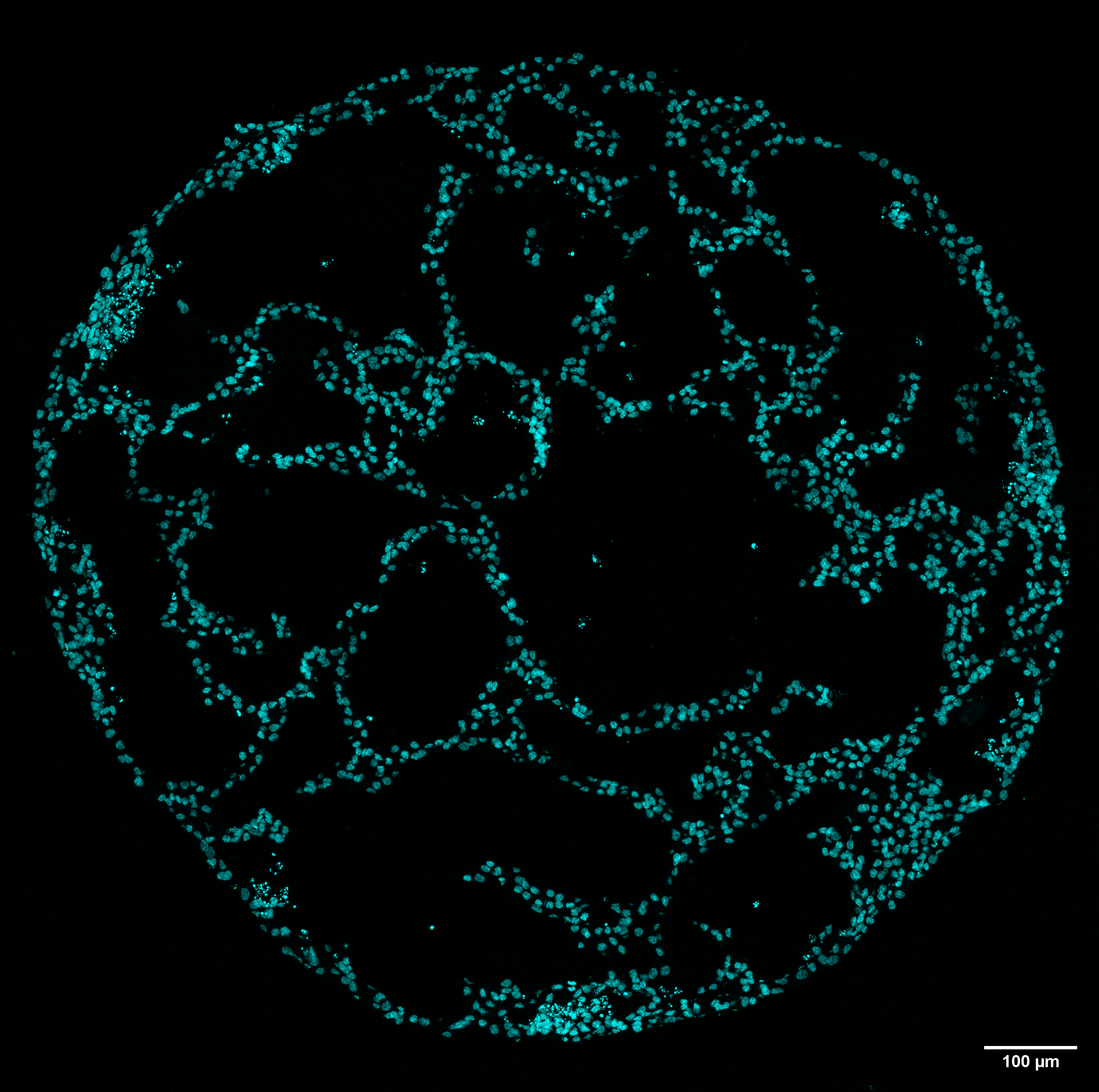

Supplement: Supplementary file 6 — Source data Fig. 6 [file 44320_2025_172_MOESM6_ESM.zip › Figure6/6H/H_SMAD2_CTR_LEFT/top/SMAD_CTR_hoechst.tif]

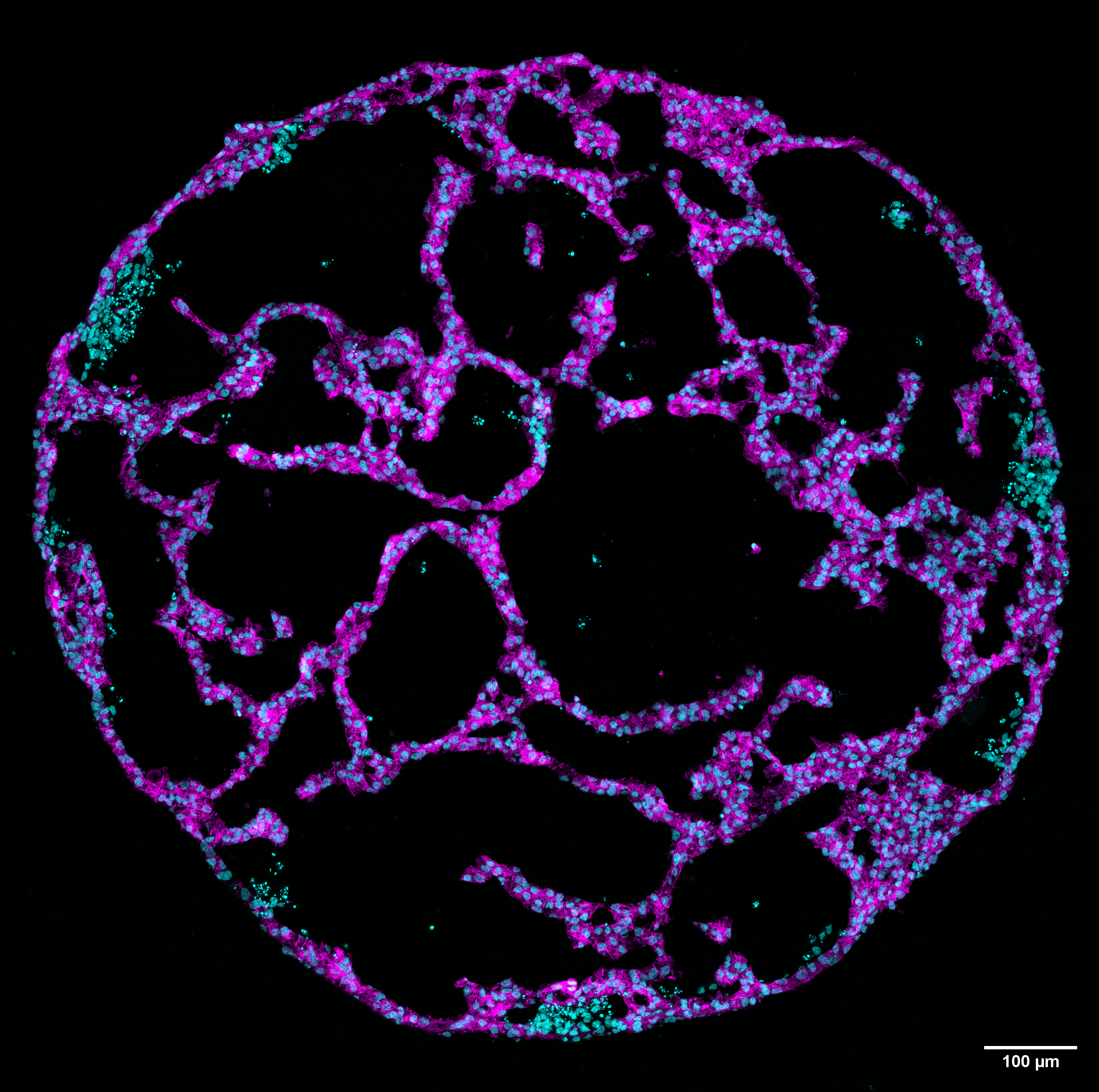

Supplement: Supplementary file 6 — Source data Fig. 6 [file 44320_2025_172_MOESM6_ESM.zip › Figure6/6H/H_SMAD2_CTR_LEFT/top/SMAD_CTR_merged.tif]

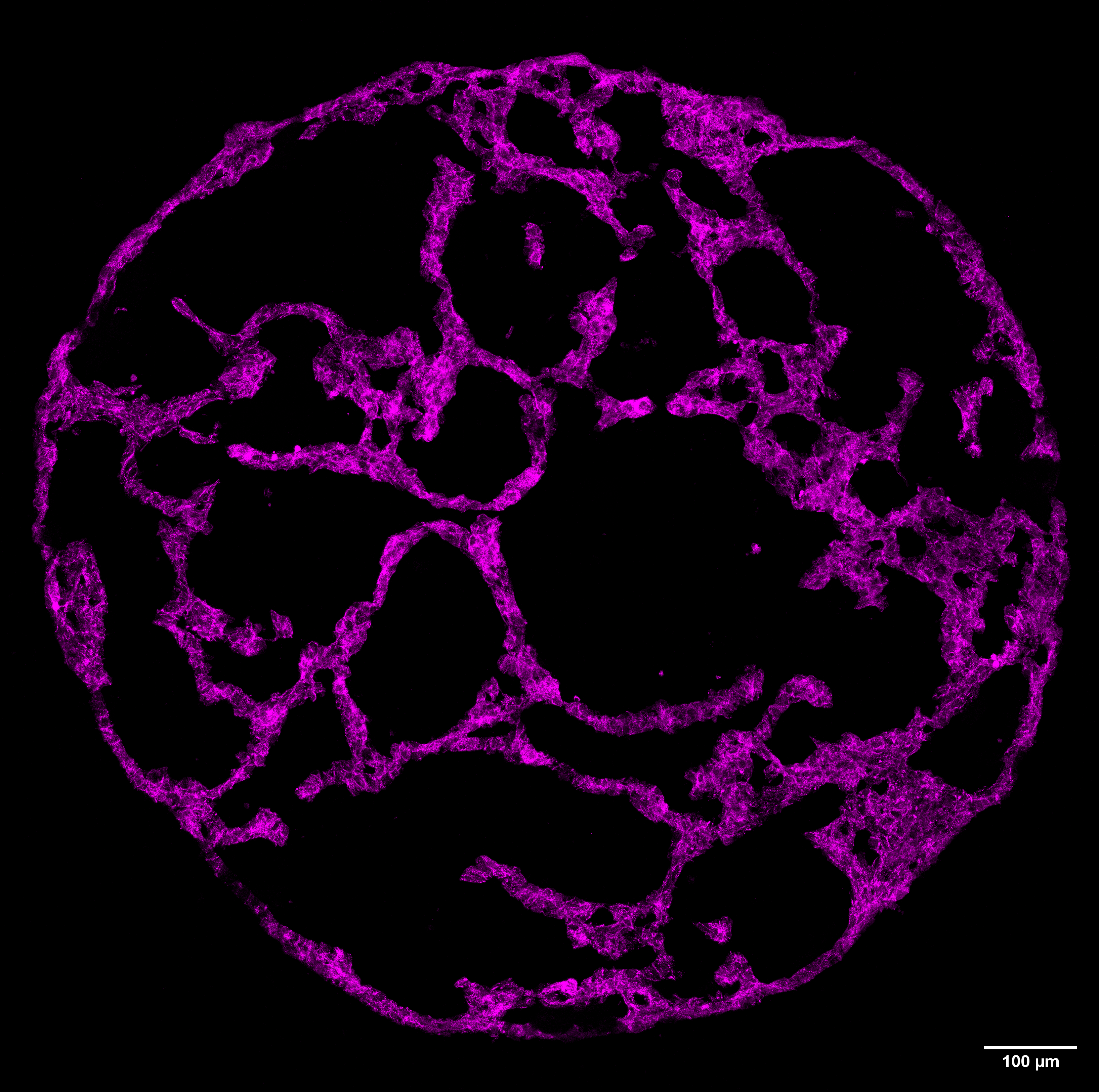

Supplement: Supplementary file 6 — Source data Fig. 6 [file 44320_2025_172_MOESM6_ESM.zip › Figure6/6H/H_SMAD2_CTR_LEFT/top/SMAD_CTR_aAct.tif]

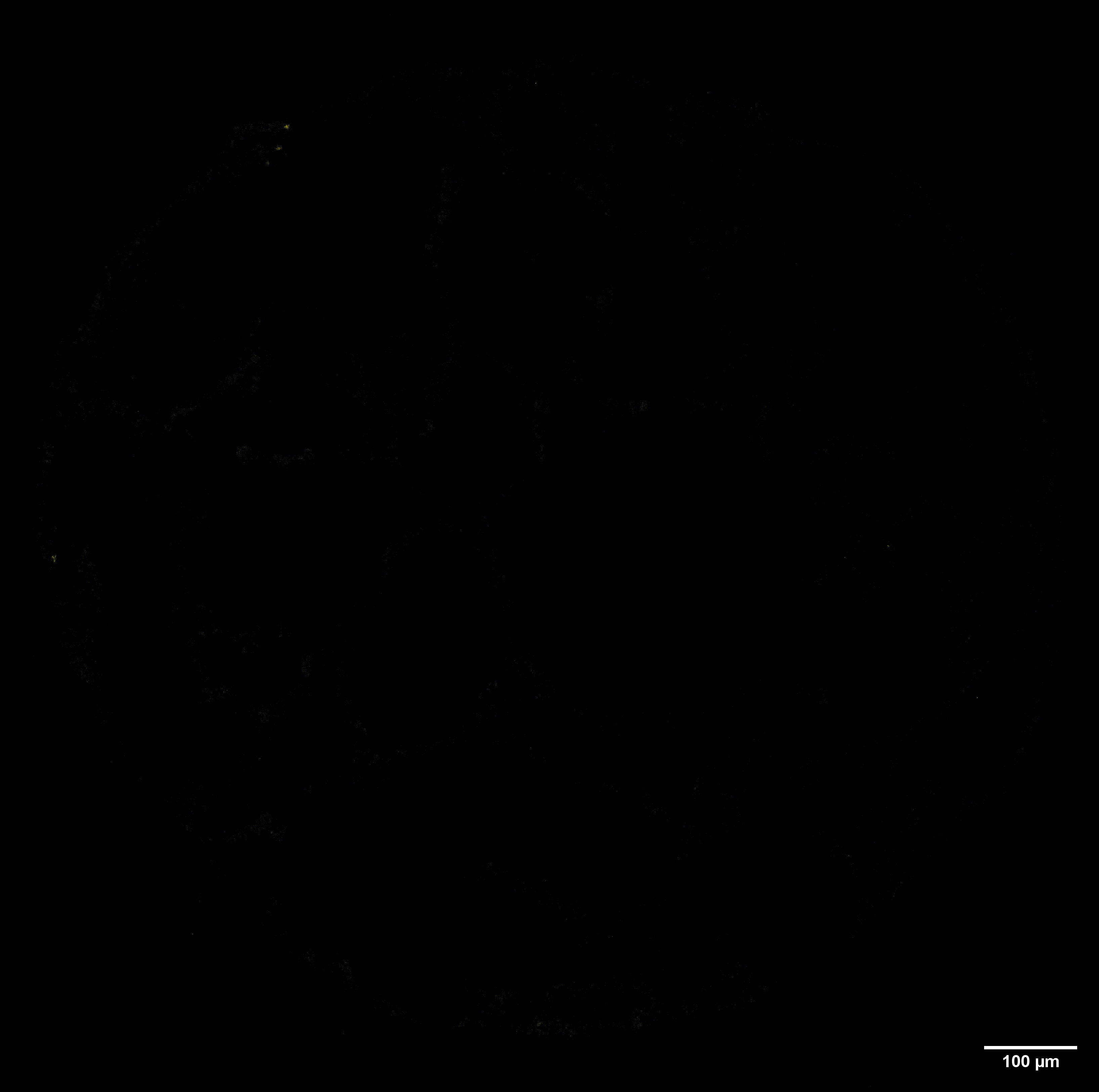

Supplement: Supplementary file 6 — Source data Fig. 6 [file 44320_2025_172_MOESM6_ESM.zip › Figure6/6H/H_SMAD2_CTR_LEFT/top/SMAD_CTR_wt1.tif]

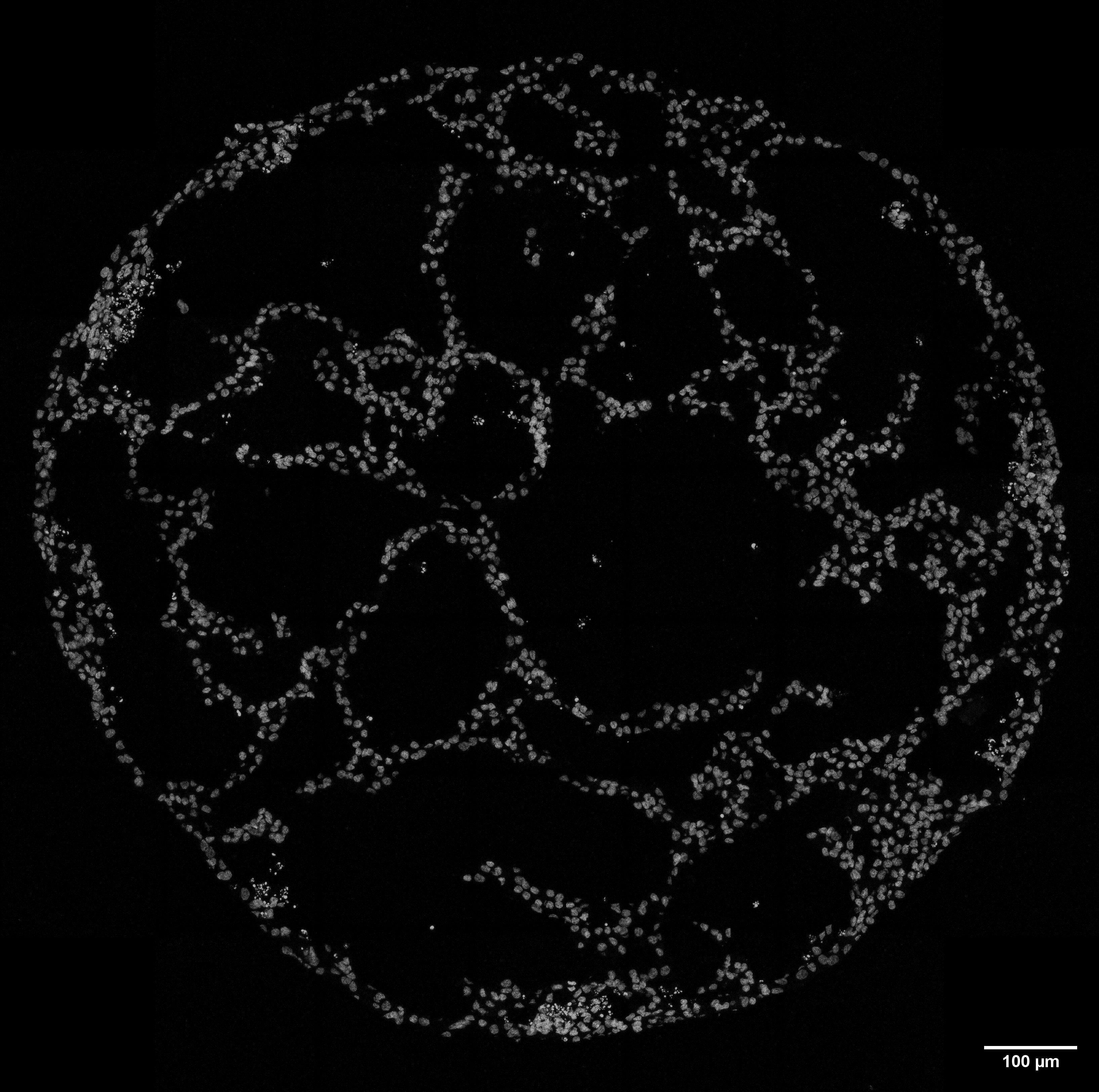

Supplement: Supplementary file 6 — Source data Fig. 6 [file 44320_2025_172_MOESM6_ESM.zip › Figure6/6H/H_SMAD2_CTR_LEFT/top/SMAD_CTR_hoechst_BW.tiff]

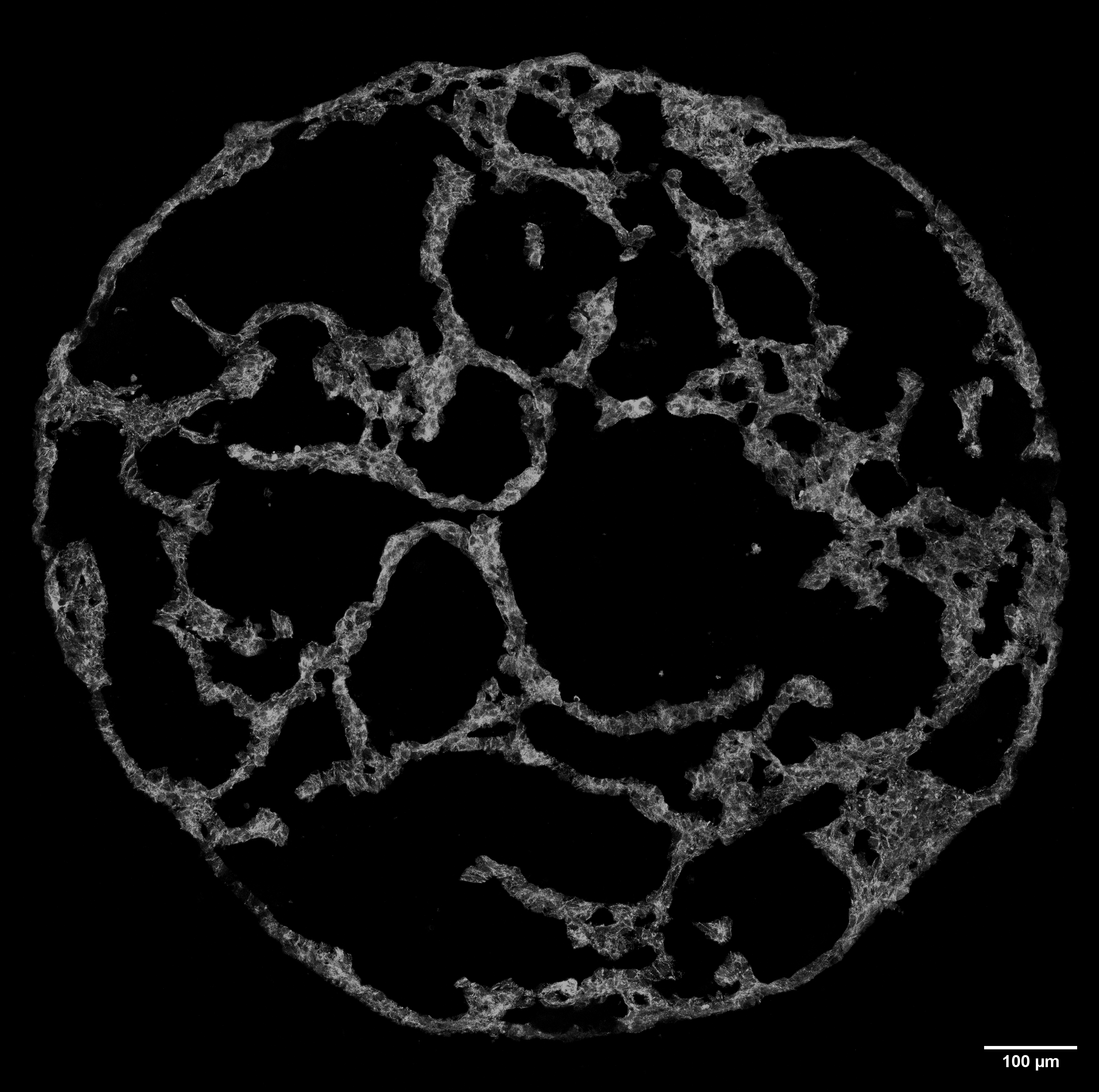

Supplement: Supplementary file 6 — Source data Fig. 6 [file 44320_2025_172_MOESM6_ESM.zip › Figure6/6H/H_SMAD2_CTR_LEFT/top/SMAD_CTR_aAct_BW.tiff]

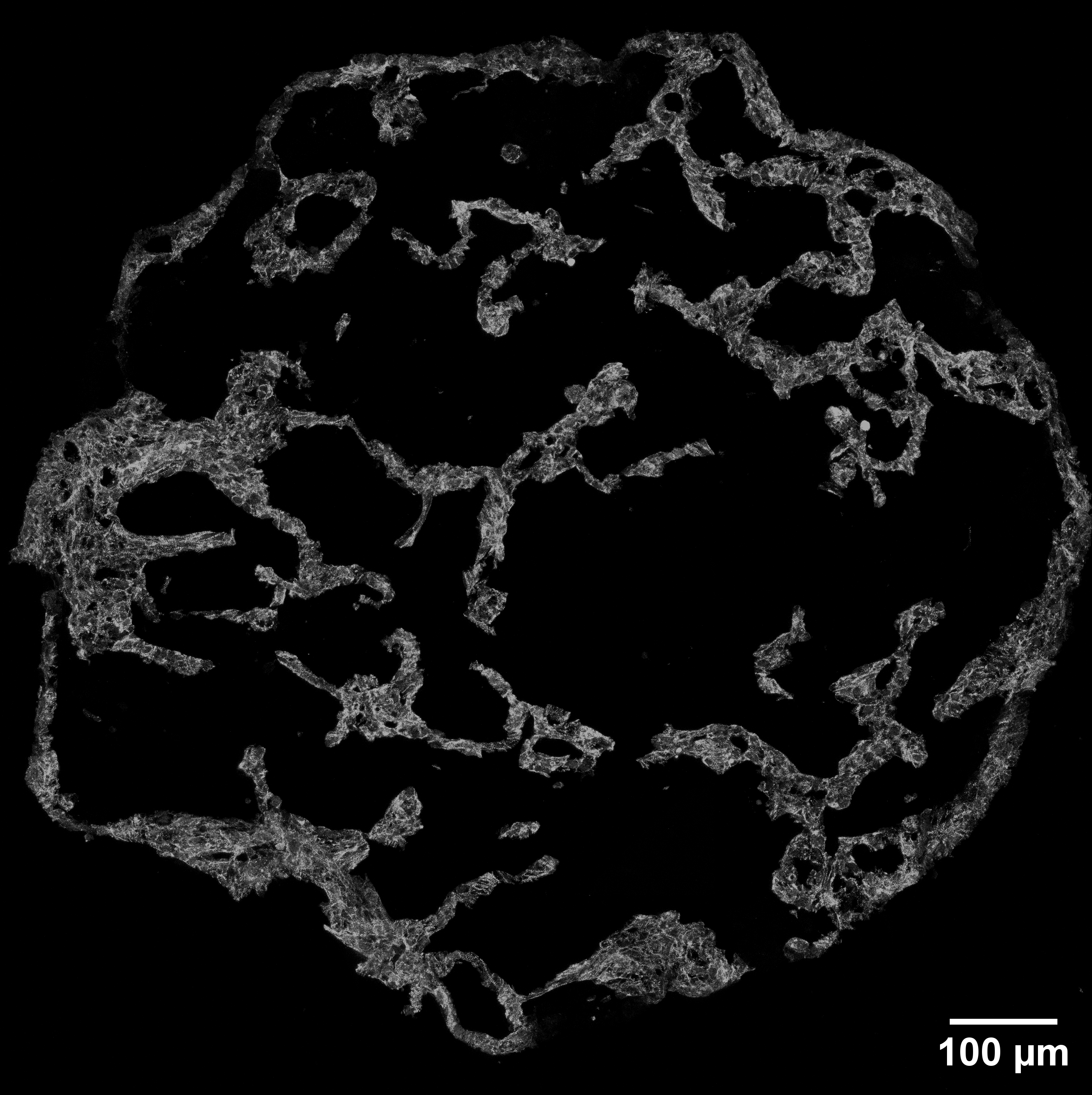

Supplement: Supplementary file 6 — Source data Fig. 6 [file 44320_2025_172_MOESM6_ESM.zip › Figure6/6H/H_SMAD2_CTR_LEFT/bottom/Smad_ctrl_aAct_BW.tiff]

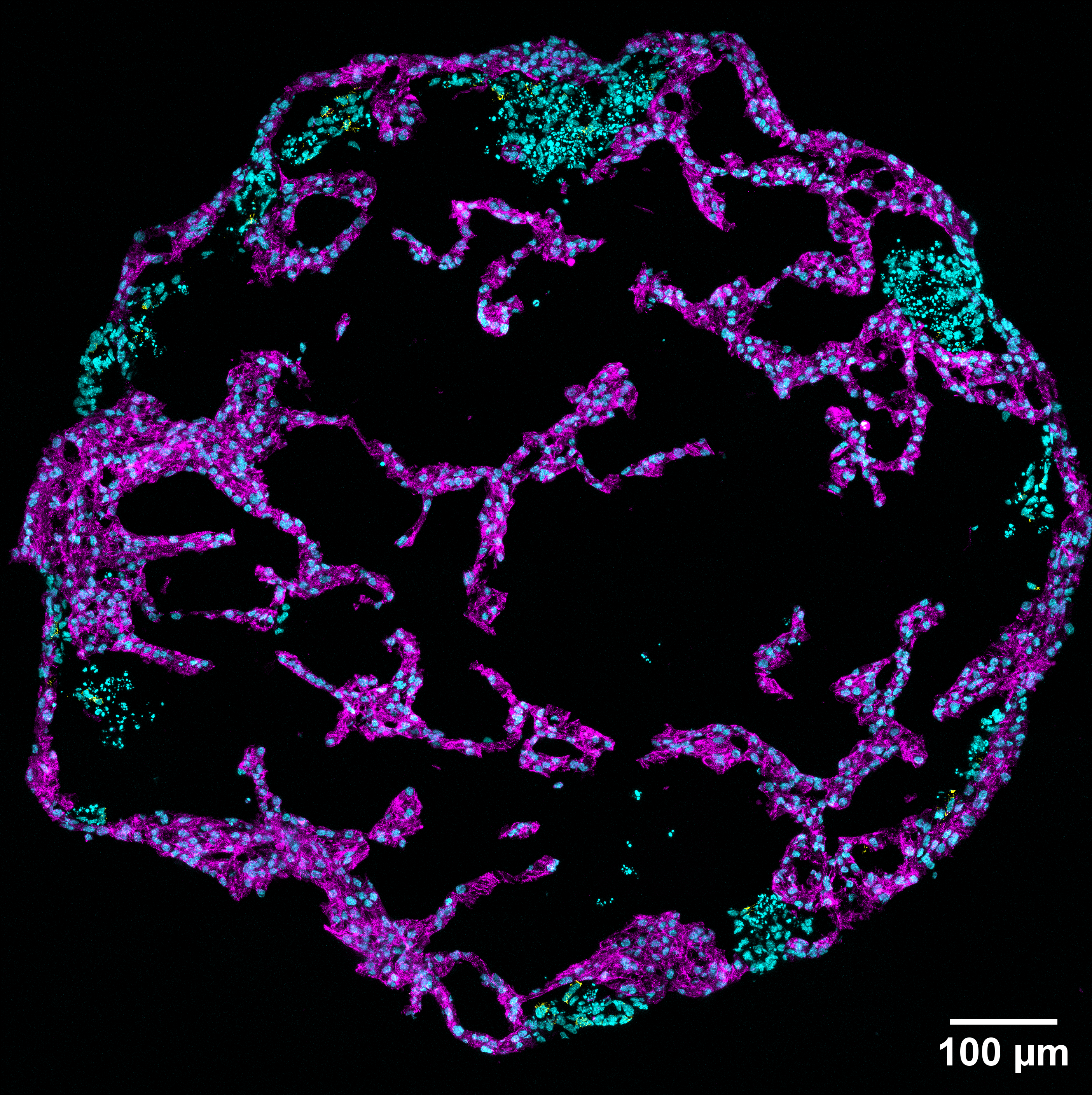

Supplement: Supplementary file 6 — Source data Fig. 6 [file 44320_2025_172_MOESM6_ESM.zip › Figure6/6H/H_SMAD2_CTR_LEFT/bottom/Smad_ctrl_merged.tif]

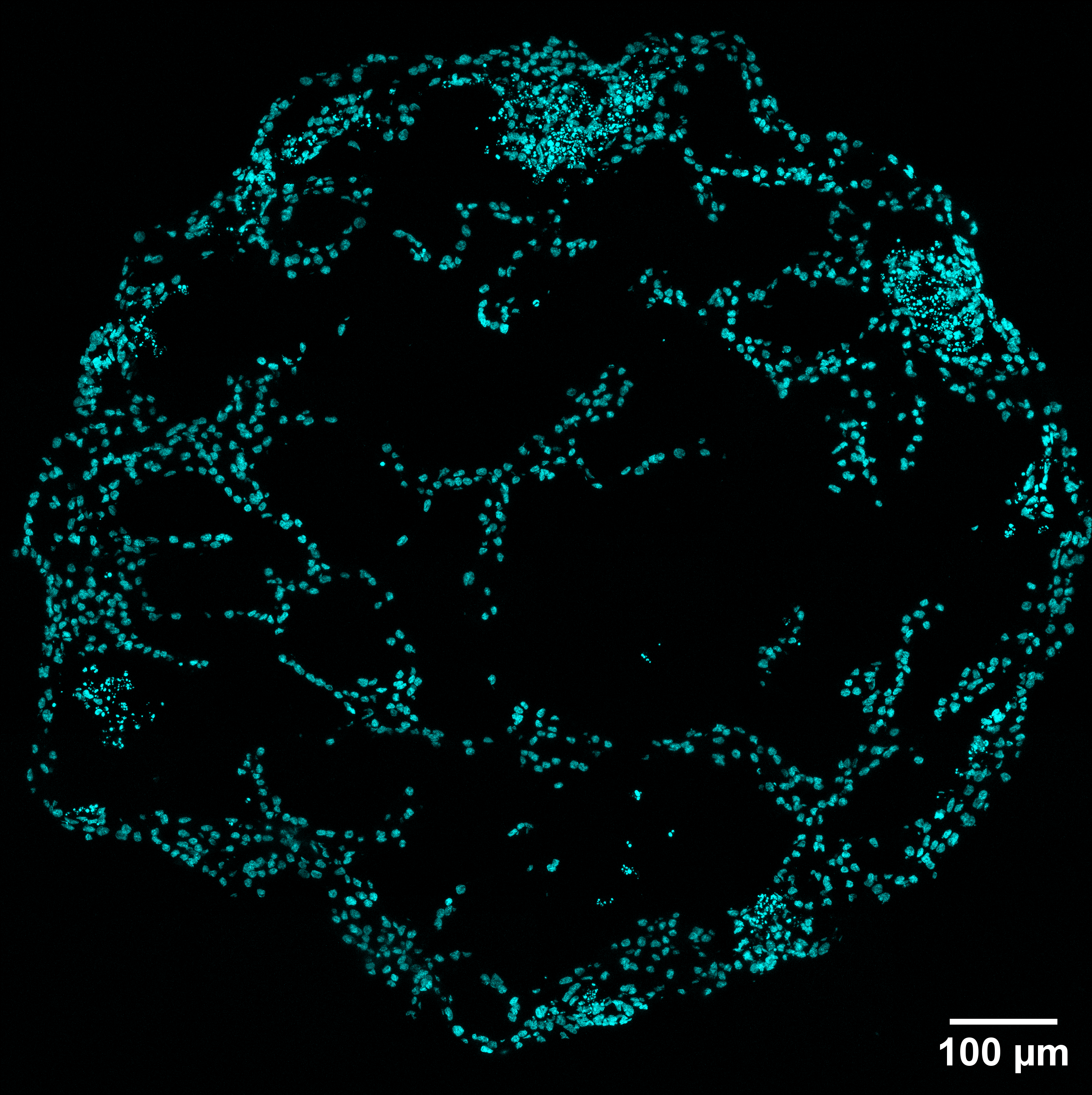

Supplement: Supplementary file 6 — Source data Fig. 6 [file 44320_2025_172_MOESM6_ESM.zip › Figure6/6H/H_SMAD2_CTR_LEFT/bottom/Smad_ctrl_hoechst.tif]

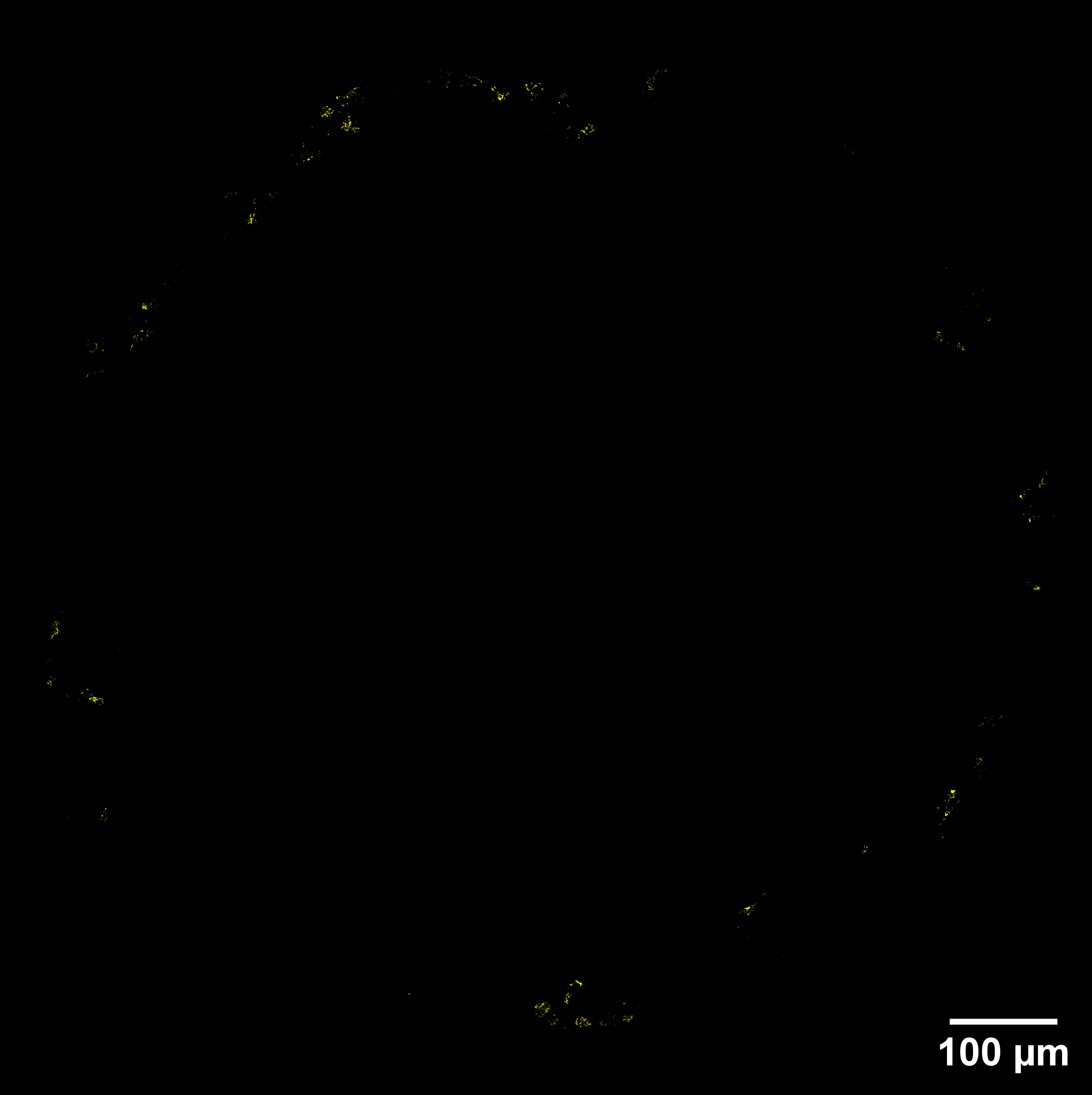

Supplement: Supplementary file 6 — Source data Fig. 6 [file 44320_2025_172_MOESM6_ESM.zip › Figure6/6H/H_SMAD2_CTR_LEFT/bottom/Smad_ctrl_col1a1.tif]

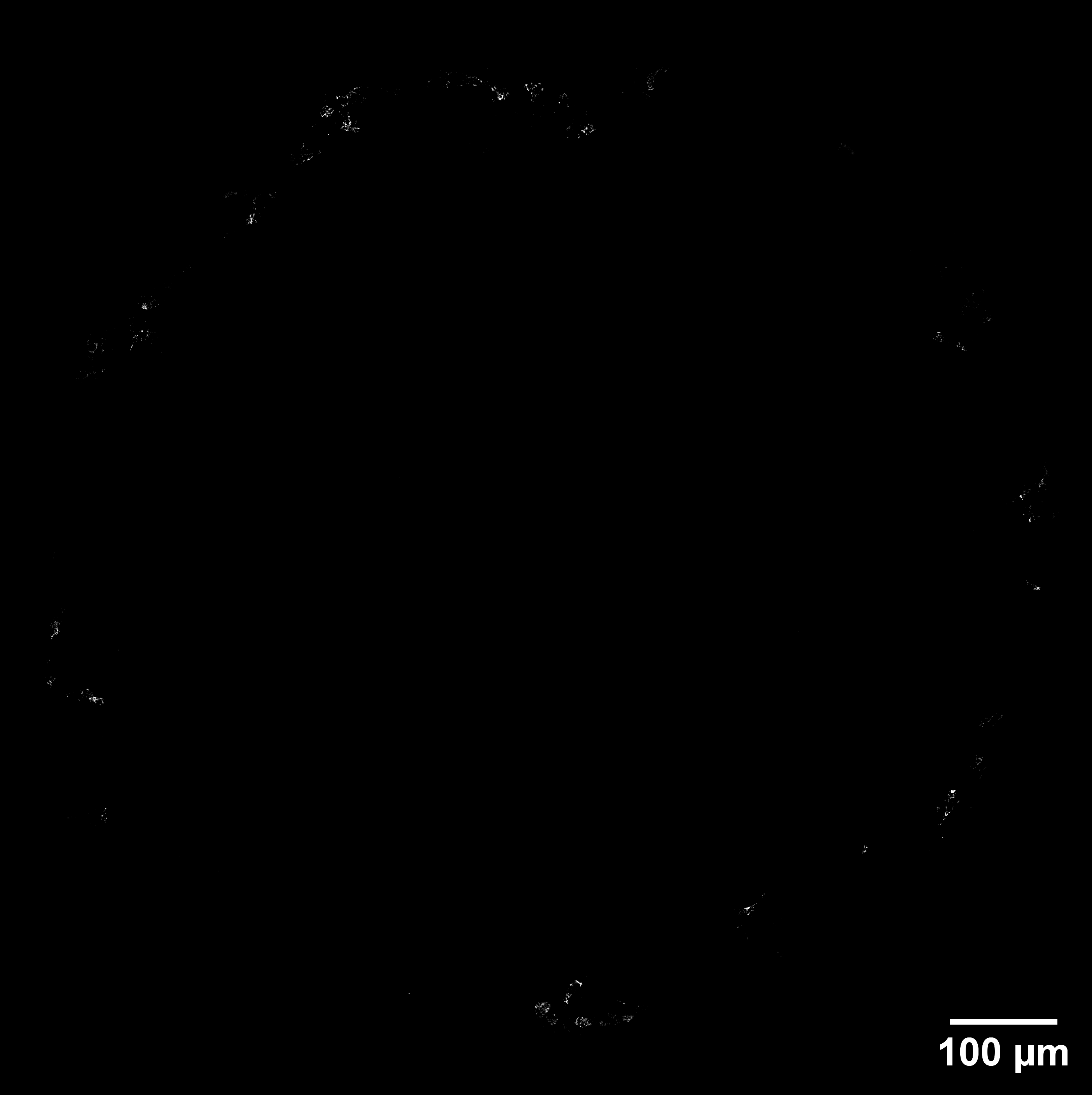

Supplement: Supplementary file 6 — Source data Fig. 6 [file 44320_2025_172_MOESM6_ESM.zip › Figure6/6H/H_SMAD2_CTR_LEFT/bottom/Smad_ctrl_col1a1_BW.tiff]

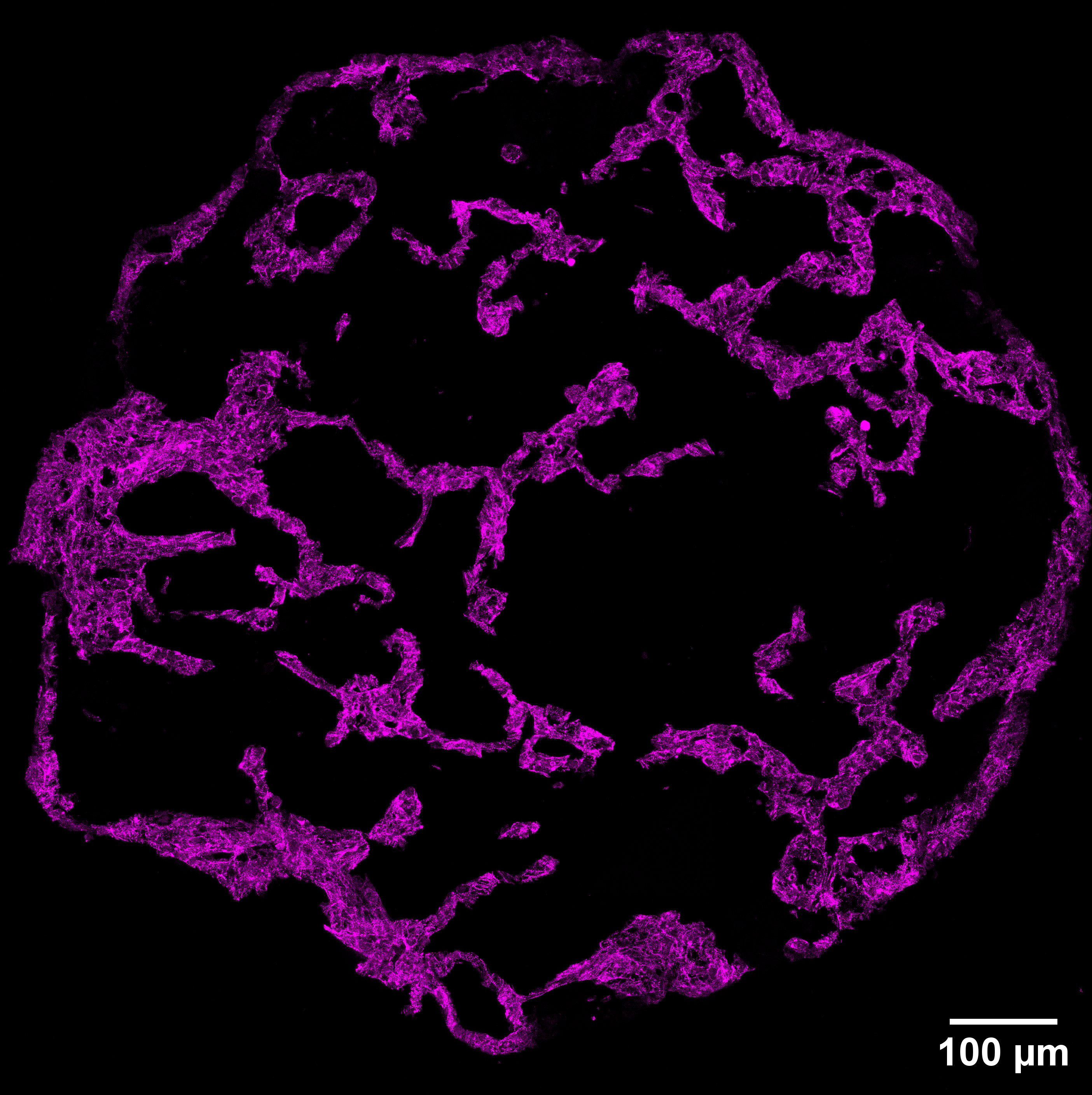

Supplement: Supplementary file 6 — Source data Fig. 6 [file 44320_2025_172_MOESM6_ESM.zip › Figure6/6H/H_SMAD2_CTR_LEFT/bottom/Smad_ctrl_aAct.tif]

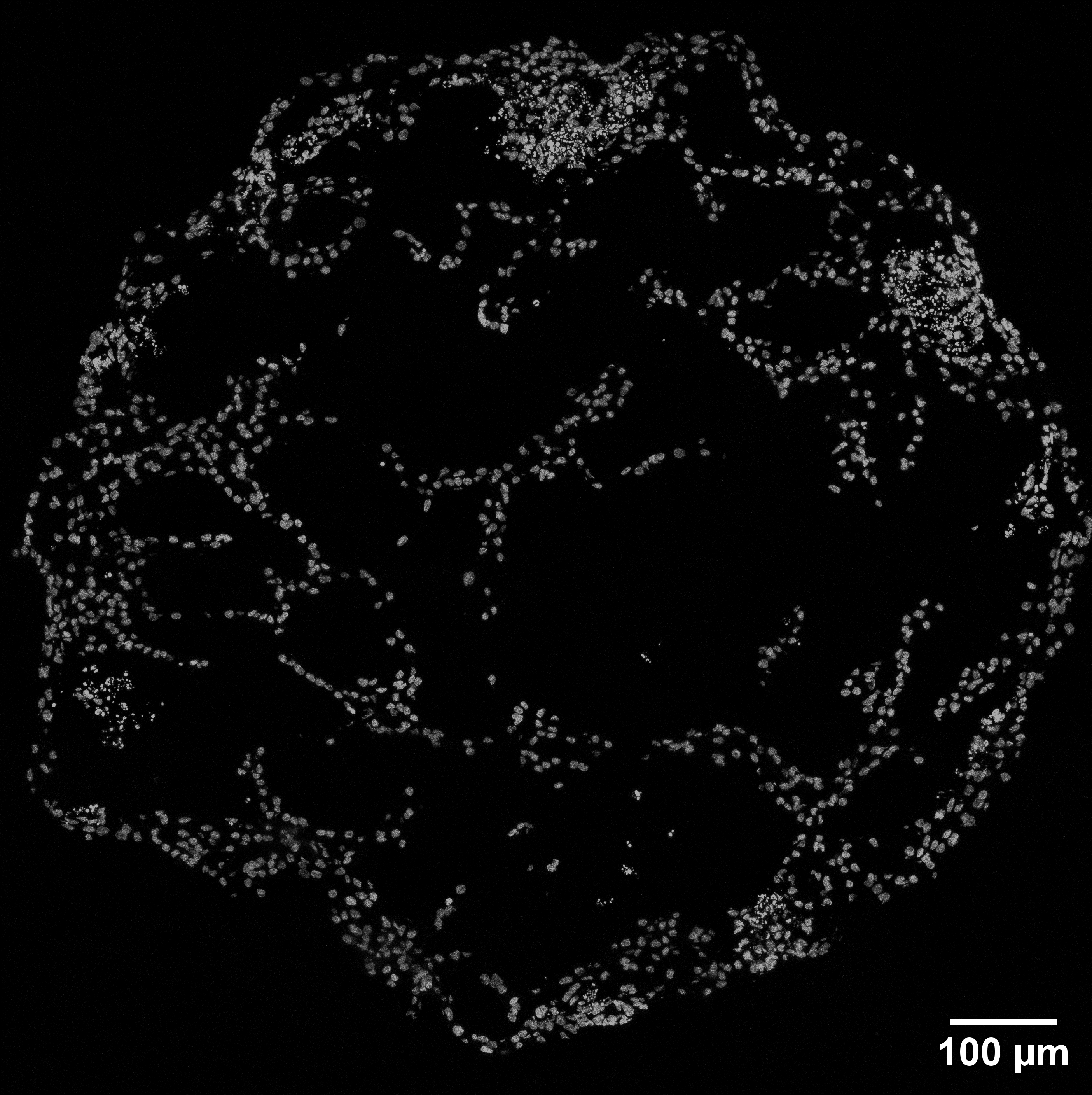

Supplement: Supplementary file 6 — Source data Fig. 6 [file 44320_2025_172_MOESM6_ESM.zip › Figure6/6H/H_SMAD2_CTR_LEFT/bottom/Smad_ctrl_hoechst_BW.tiff]

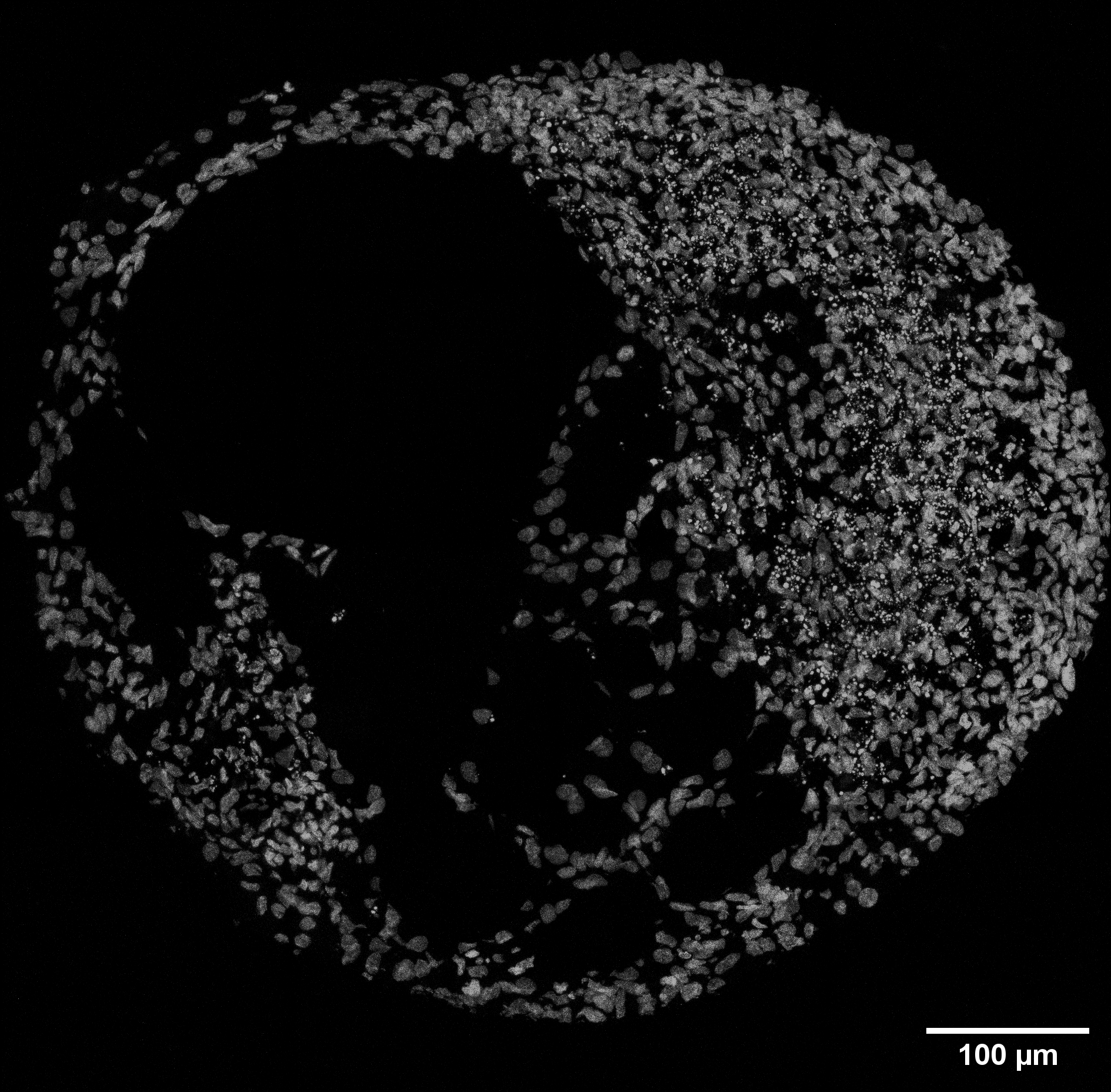

Supplement: Supplementary file 6 — Source data Fig. 6 [file 44320_2025_172_MOESM6_ESM.zip › Figure6/6H/H_SMAD2_TET_RIGHT/top/SMAD_TET_hoechst_BW.tiff]

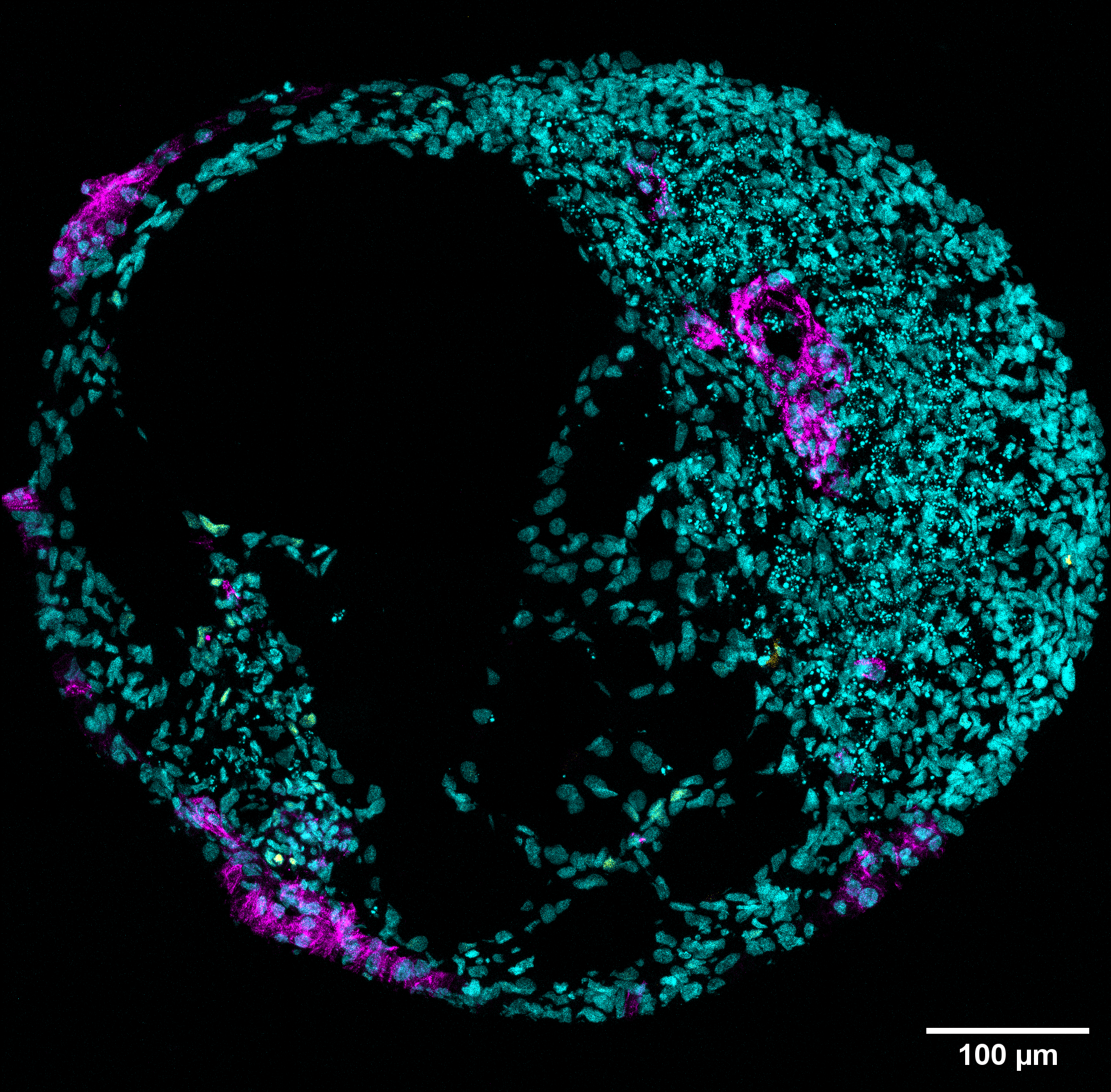

Supplement: Supplementary file 6 — Source data Fig. 6 [file 44320_2025_172_MOESM6_ESM.zip › Figure6/6H/H_SMAD2_TET_RIGHT/top/SMAD_TET_merged.tif]

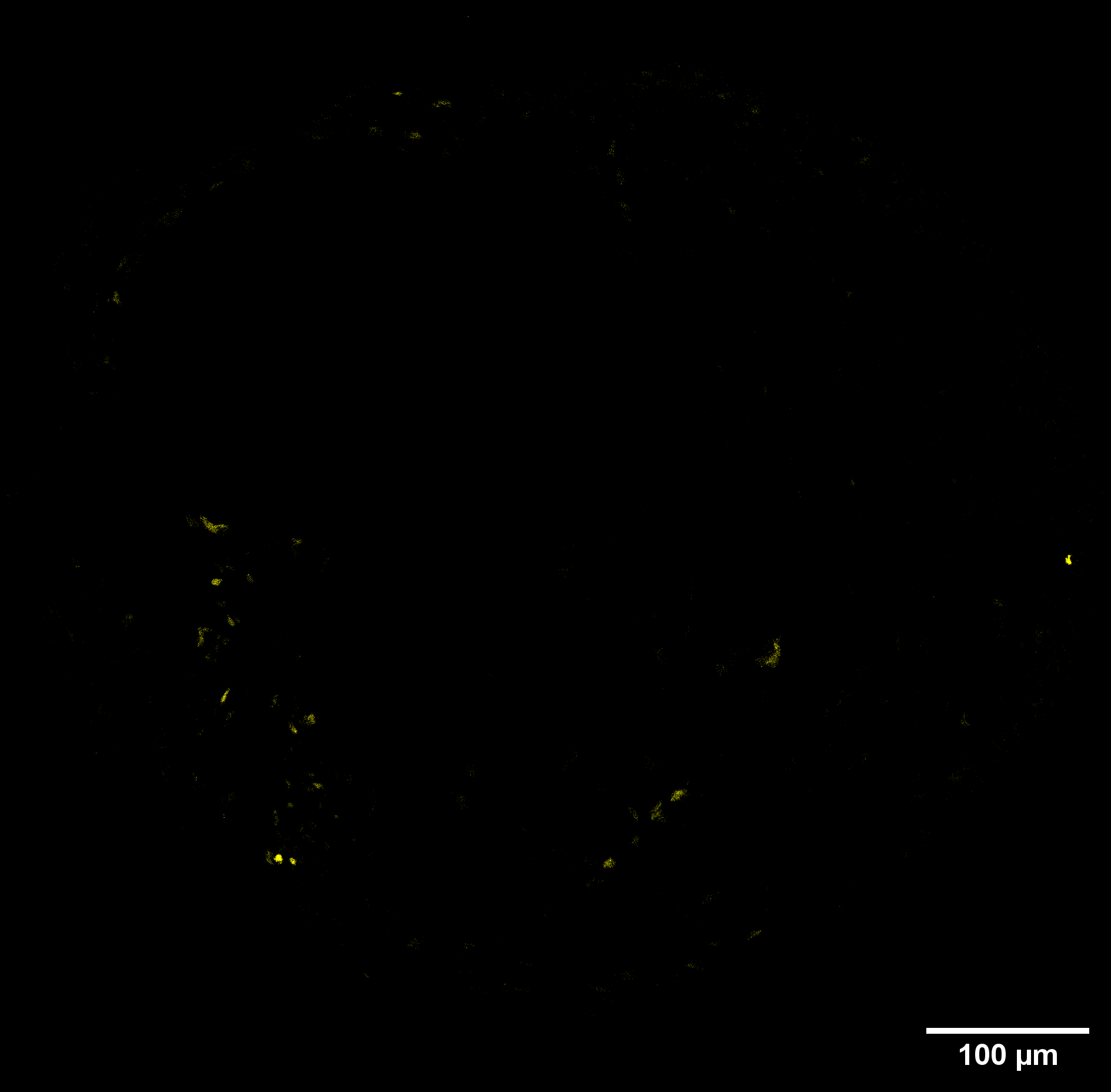

Supplement: Supplementary file 6 — Source data Fig. 6 [file 44320_2025_172_MOESM6_ESM.zip › Figure6/6H/H_SMAD2_TET_RIGHT/top/SMAD_TET_wt1.tif]

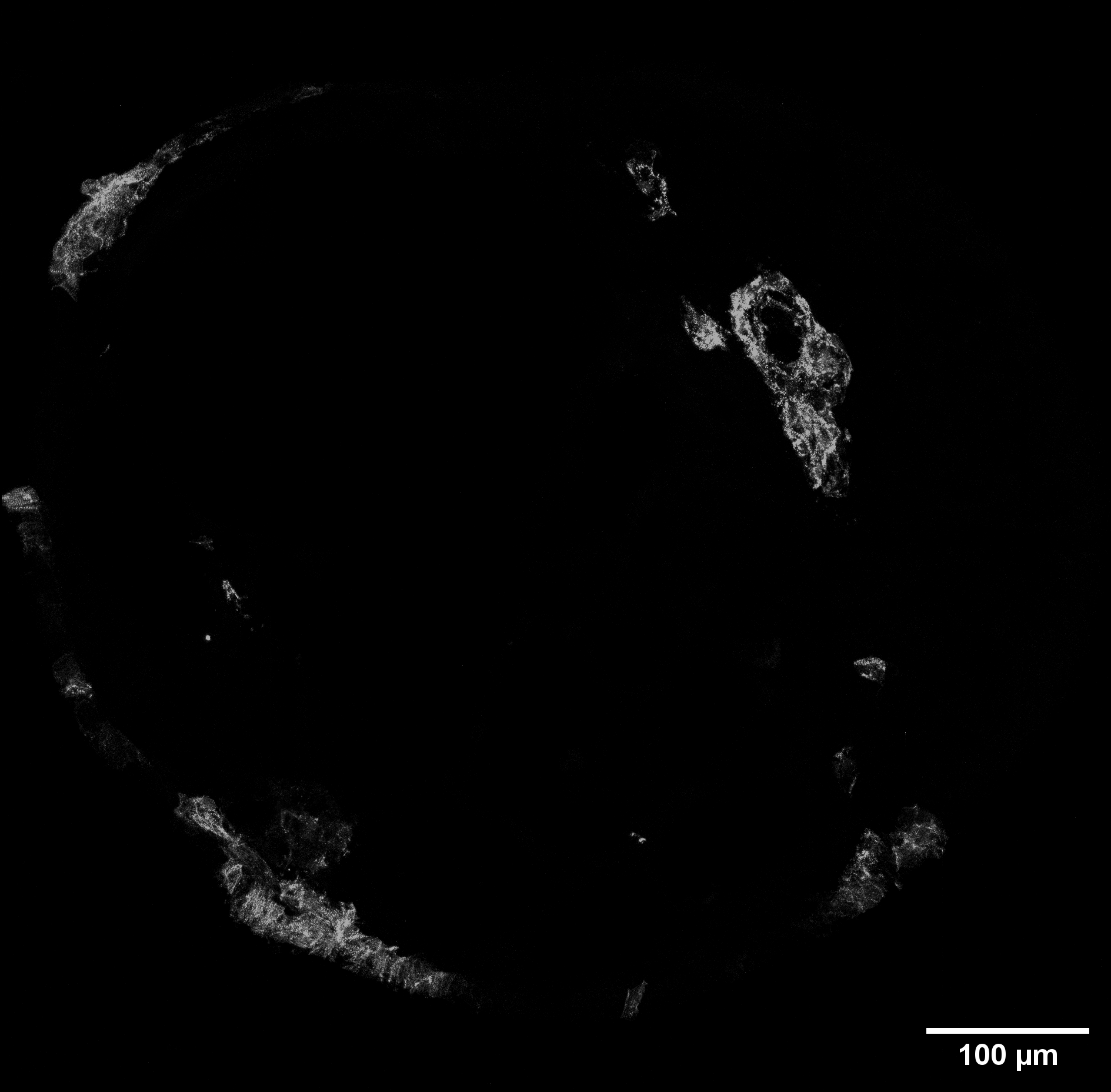

Supplement: Supplementary file 6 — Source data Fig. 6 [file 44320_2025_172_MOESM6_ESM.zip › Figure6/6H/H_SMAD2_TET_RIGHT/top/SMAD_TET_aAct_BW.tiff]

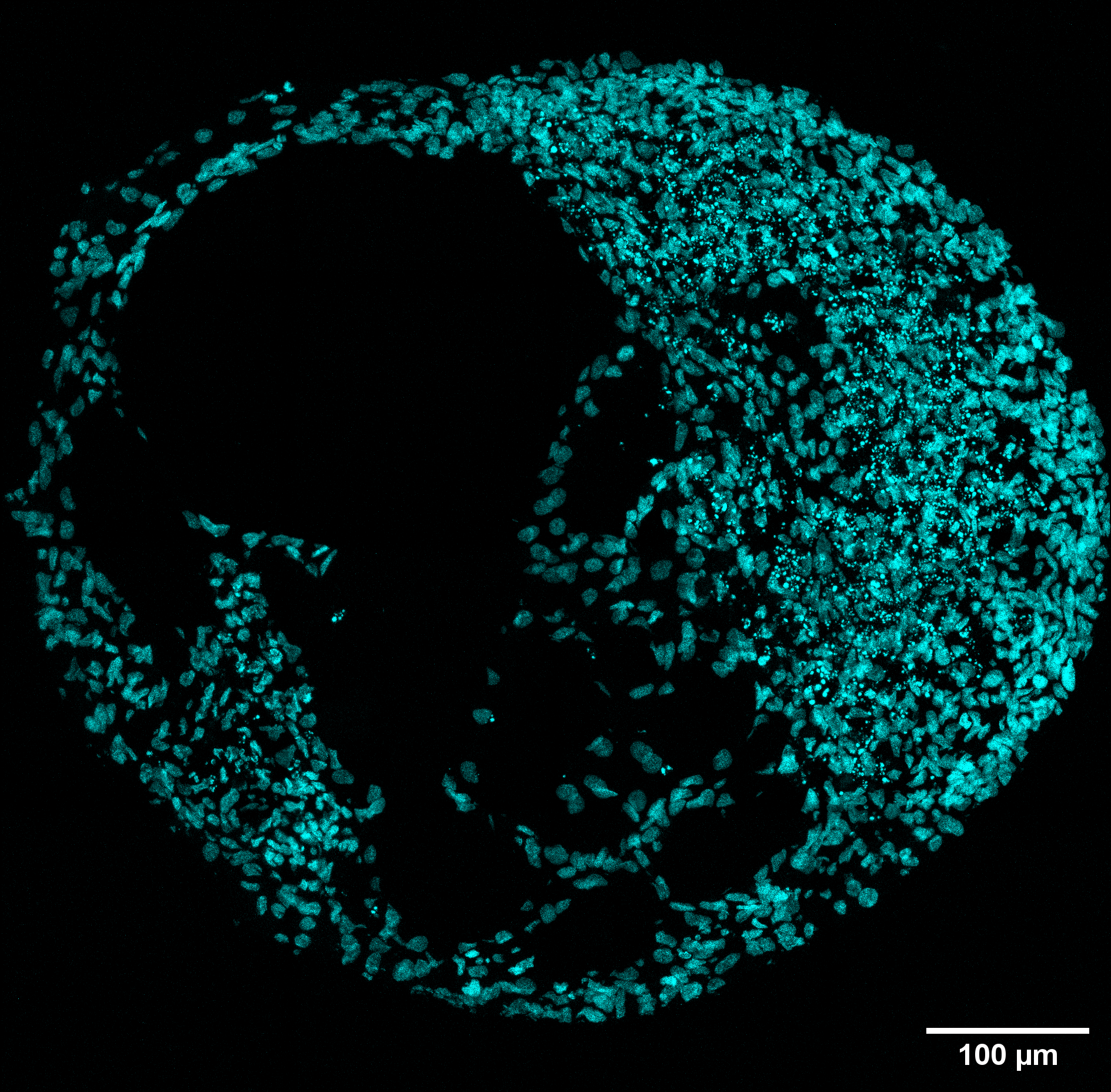

Supplement: Supplementary file 6 — Source data Fig. 6 [file 44320_2025_172_MOESM6_ESM.zip › Figure6/6H/H_SMAD2_TET_RIGHT/top/SMAD_TET_hoechst.tif]

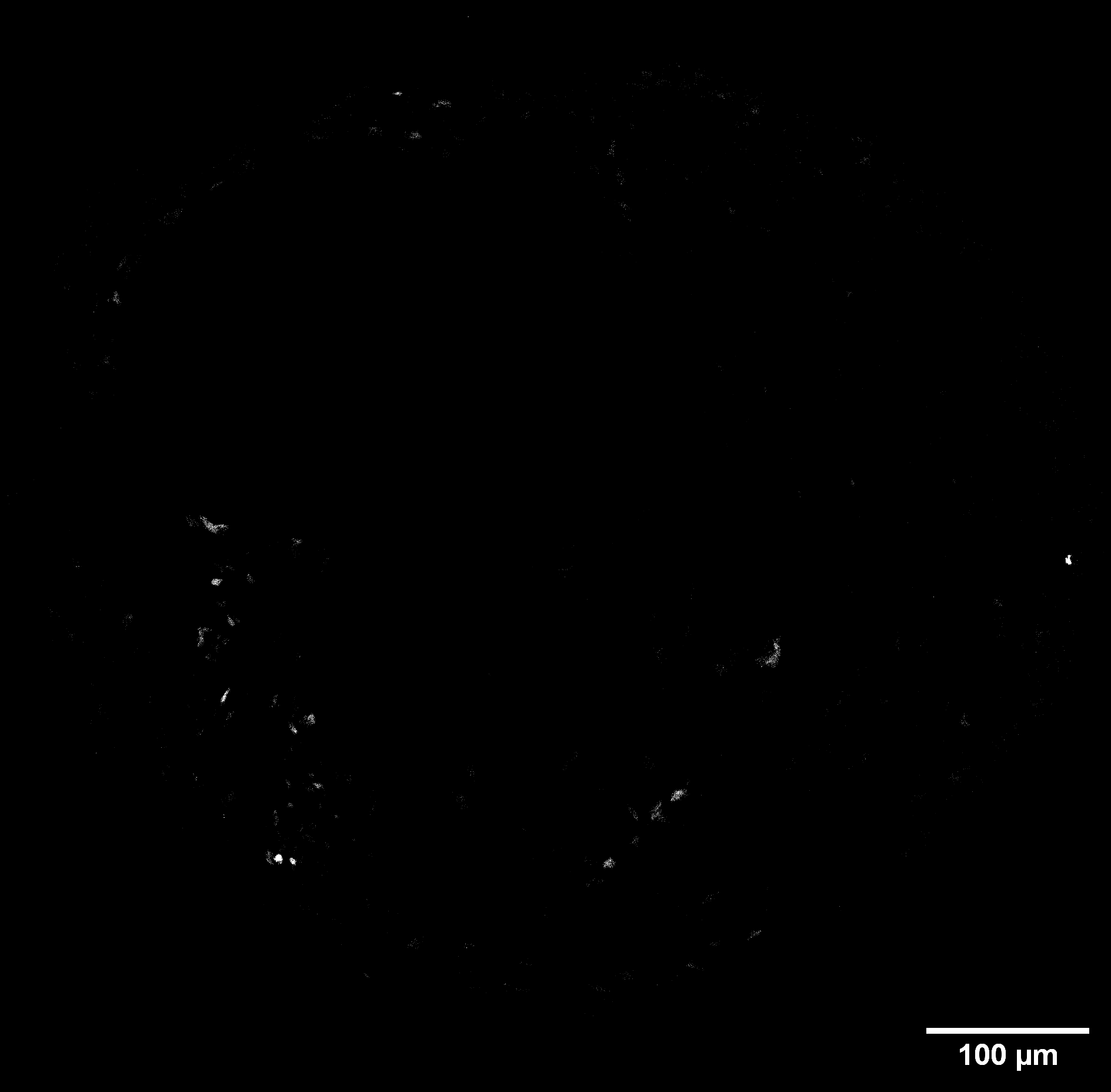

Supplement: Supplementary file 6 — Source data Fig. 6 [file 44320_2025_172_MOESM6_ESM.zip › Figure6/6H/H_SMAD2_TET_RIGHT/top/SMAD_TET_wt1_BW.tif]

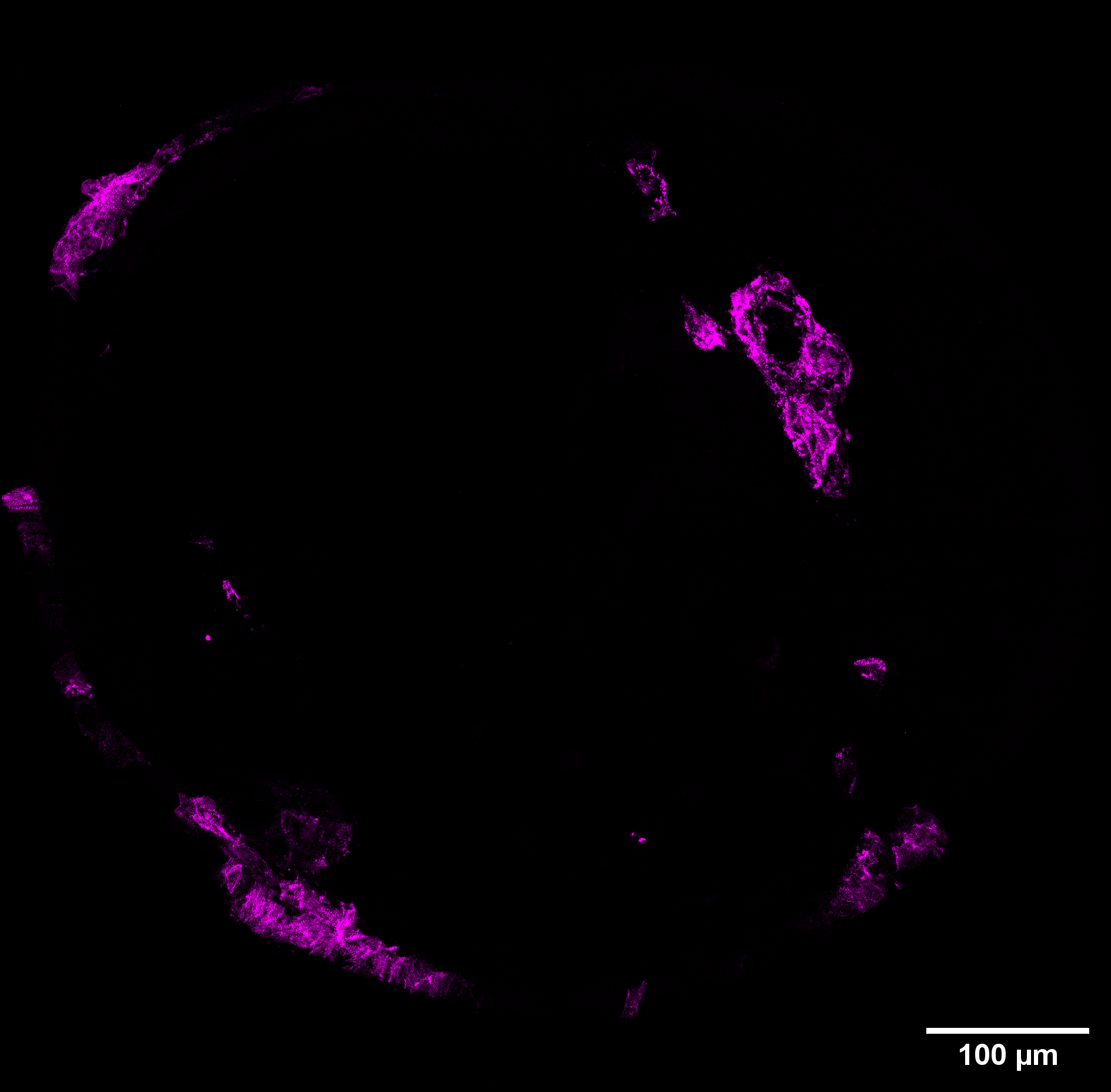

Supplement: Supplementary file 6 — Source data Fig. 6 [file 44320_2025_172_MOESM6_ESM.zip › Figure6/6H/H_SMAD2_TET_RIGHT/top/SMAD_TET_aAct.tif]

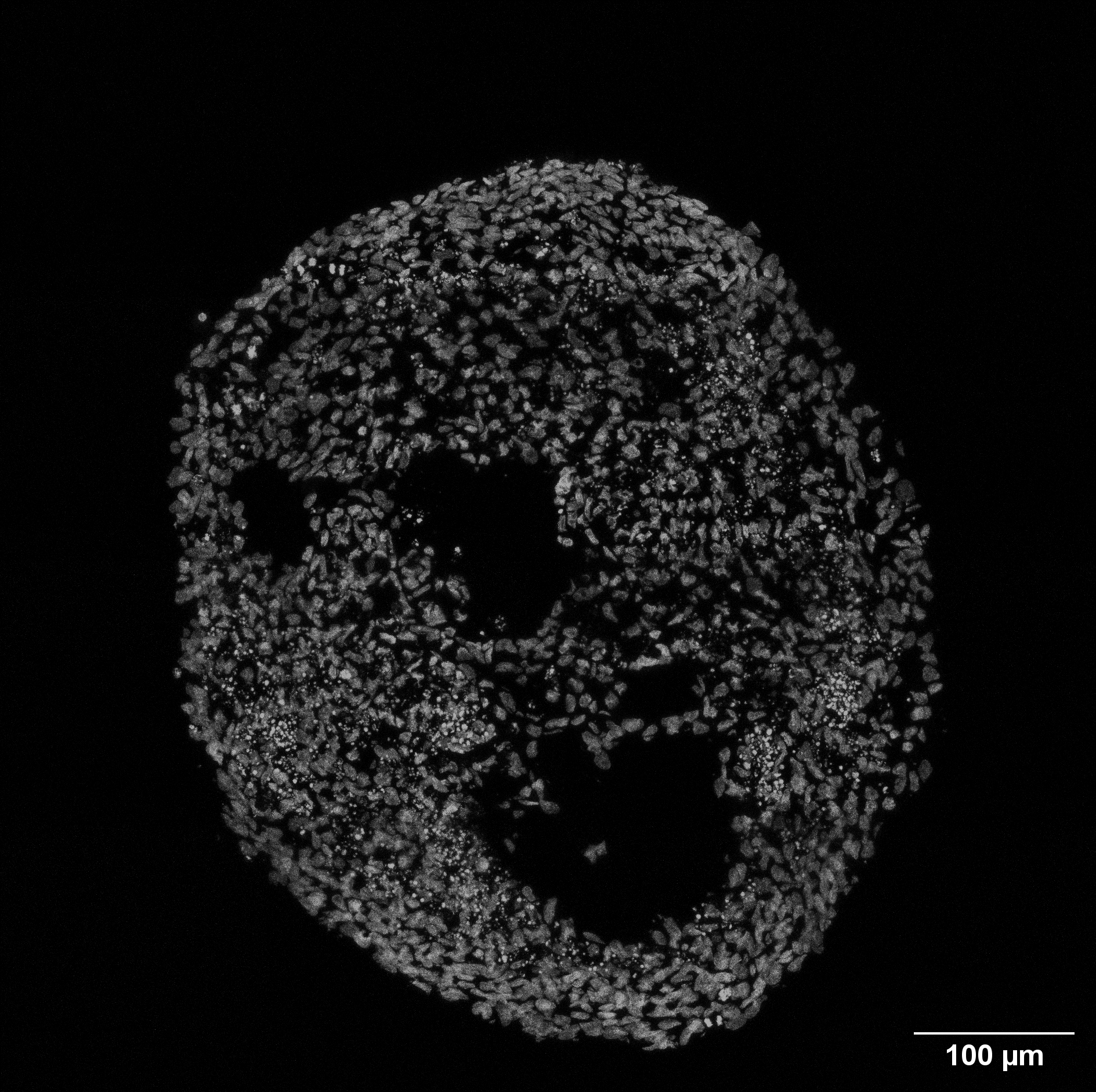

Supplement: Supplementary file 6 — Source data Fig. 6 [file 44320_2025_172_MOESM6_ESM.zip › Figure6/6H/H_SMAD2_TET_RIGHT/bottom/Smad_tet_hoechst_BW.tiff]

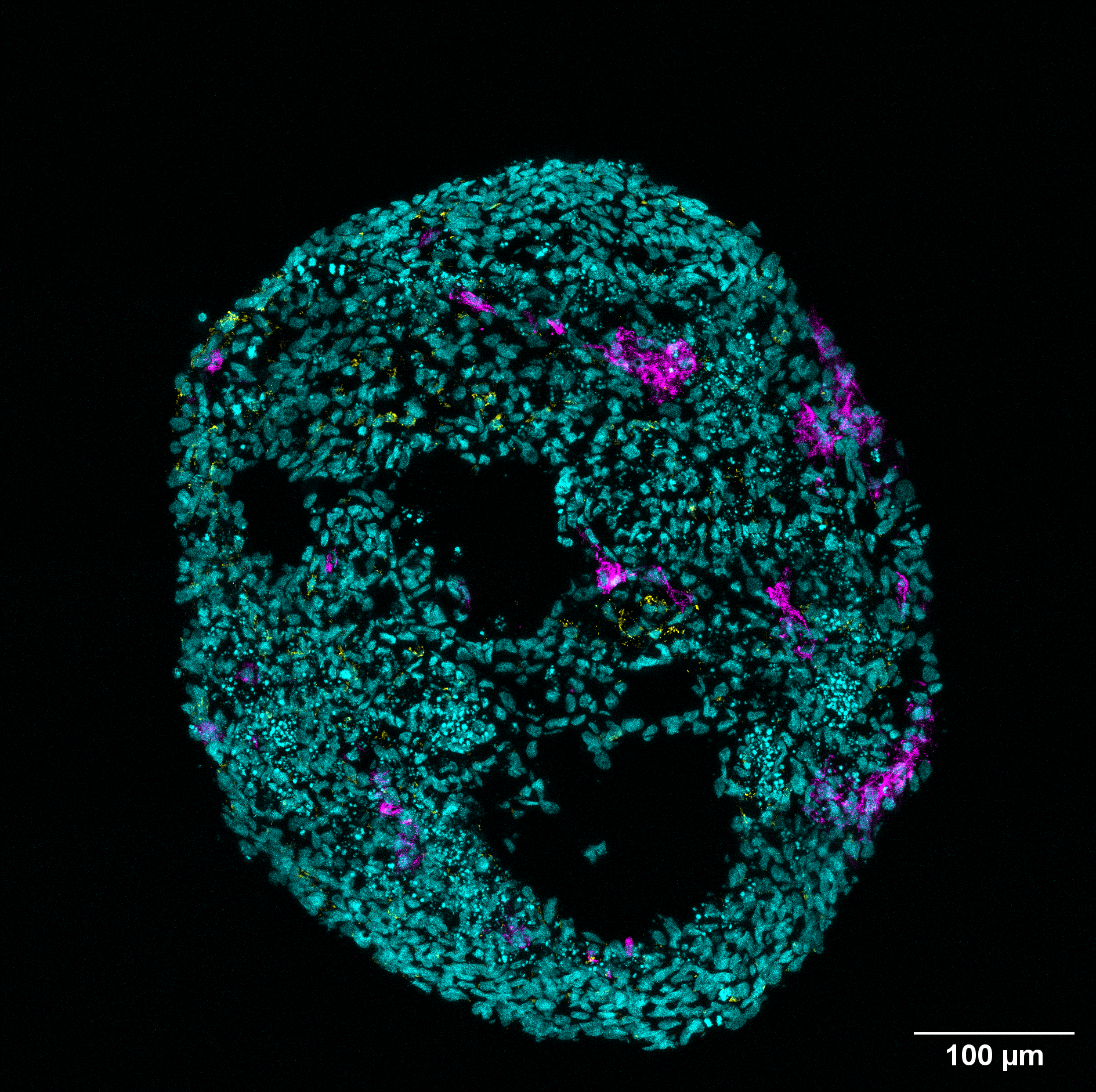

Supplement: Supplementary file 6 — Source data Fig. 6 [file 44320_2025_172_MOESM6_ESM.zip › Figure6/6H/H_SMAD2_TET_RIGHT/bottom/Smad_tet_merged.tif]

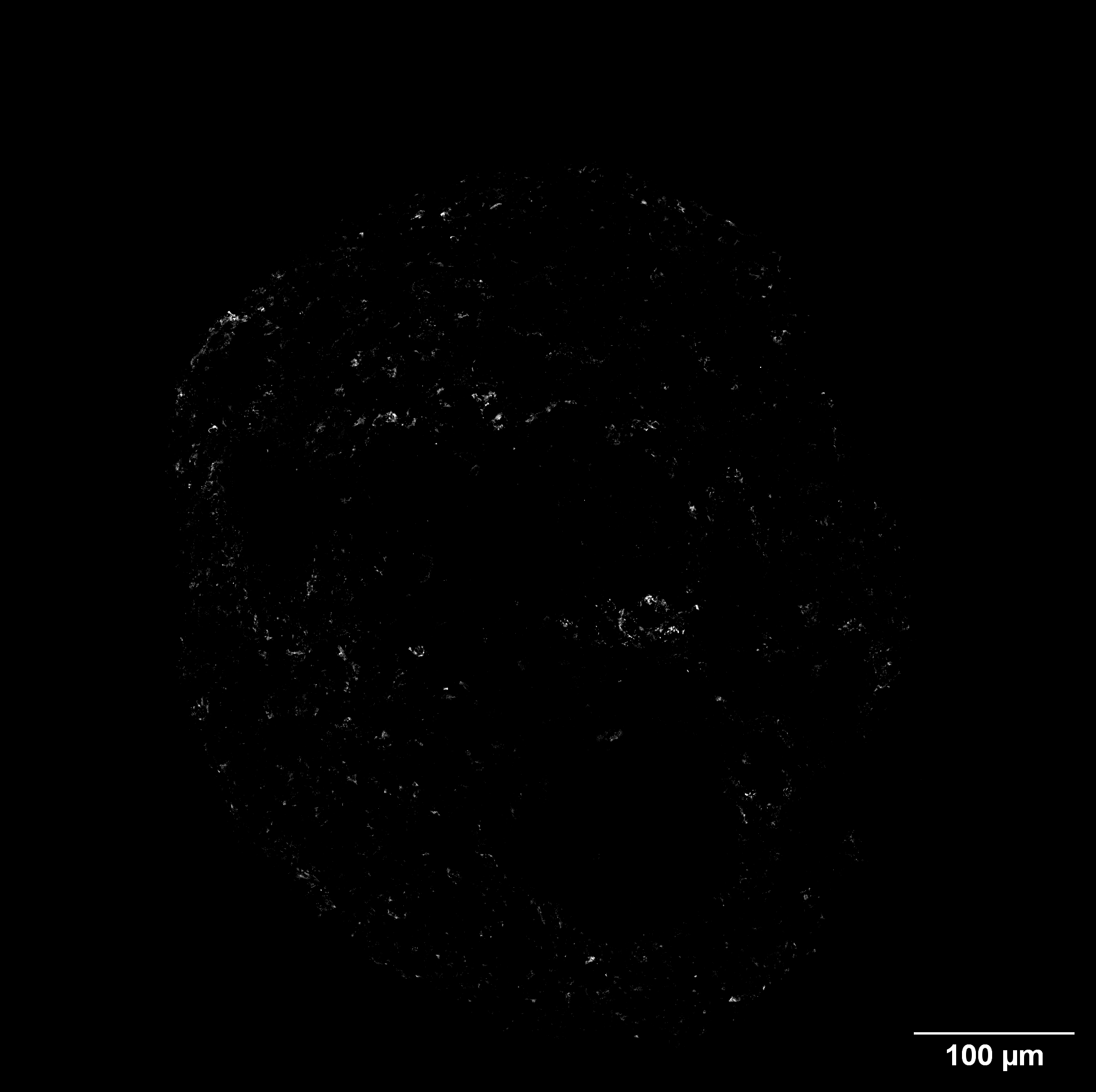

Supplement: Supplementary file 6 — Source data Fig. 6 [file 44320_2025_172_MOESM6_ESM.zip › Figure6/6H/H_SMAD2_TET_RIGHT/bottom/Smad_tet_col1a1_BW.tiff]

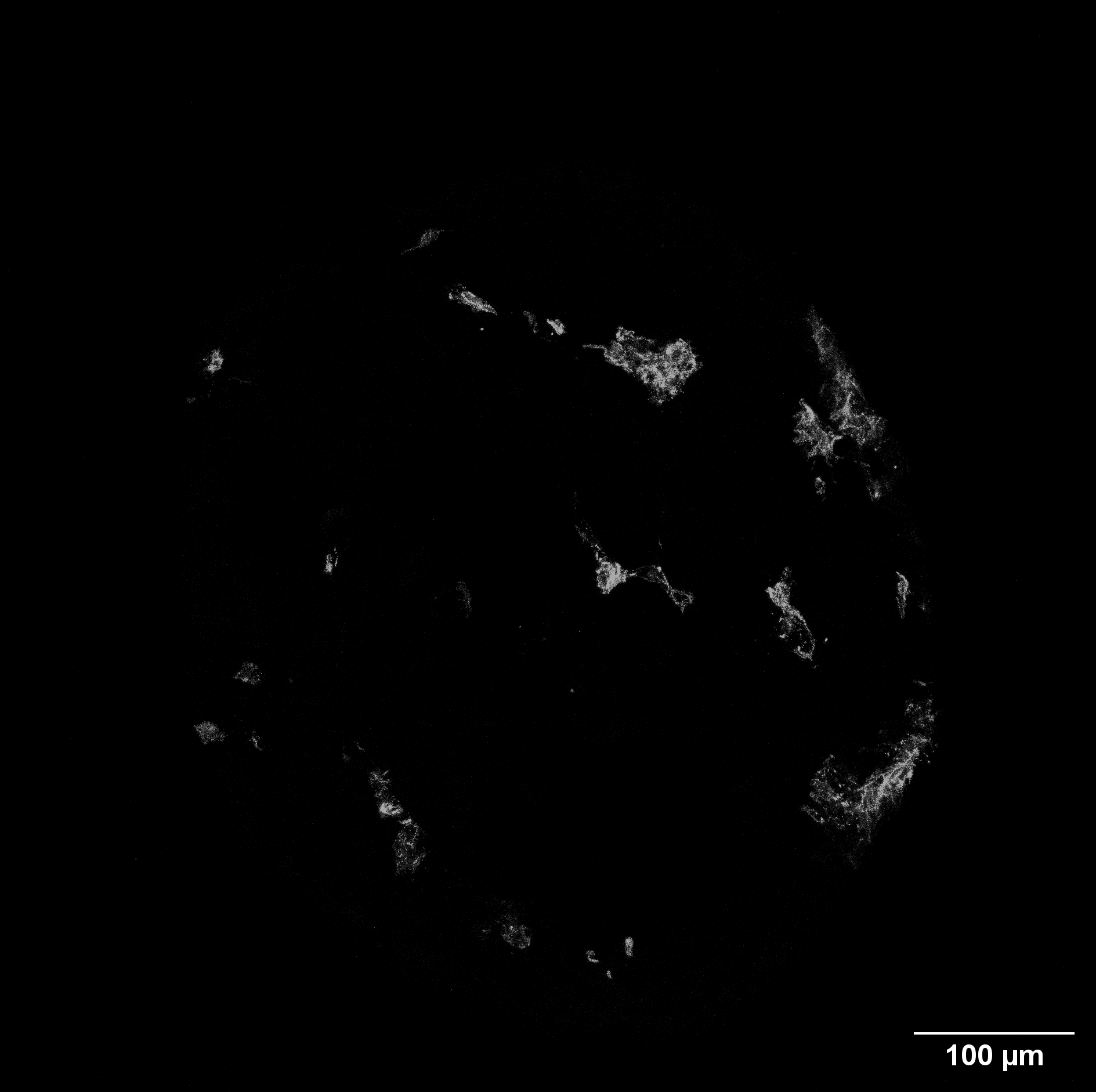

Supplement: Supplementary file 6 — Source data Fig. 6 [file 44320_2025_172_MOESM6_ESM.zip › Figure6/6H/H_SMAD2_TET_RIGHT/bottom/Smad_tet_aAct_BW.tiff]

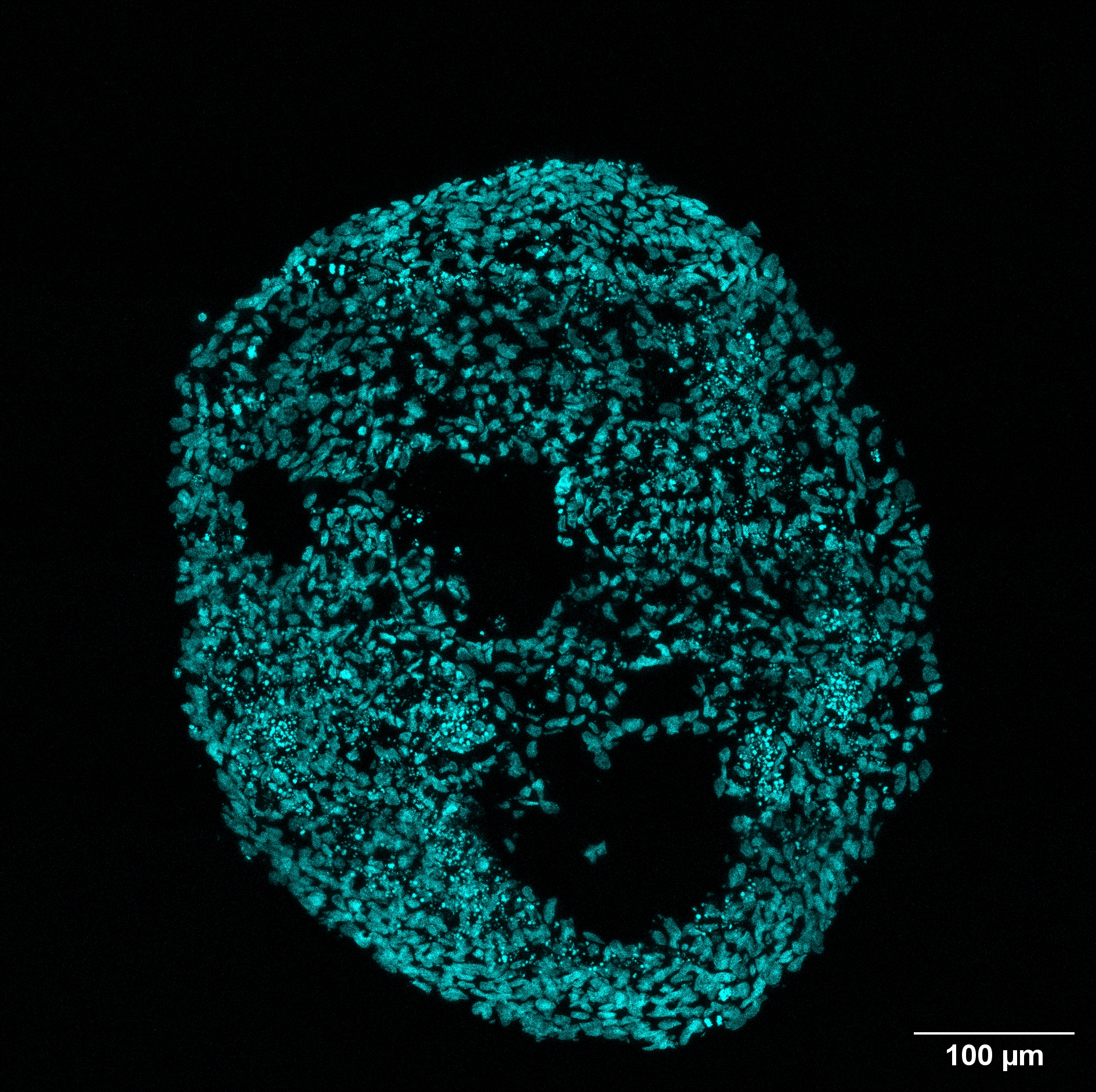

Supplement: Supplementary file 6 — Source data Fig. 6 [file 44320_2025_172_MOESM6_ESM.zip › Figure6/6H/H_SMAD2_TET_RIGHT/bottom/Smad_tet_hoechst.tif]

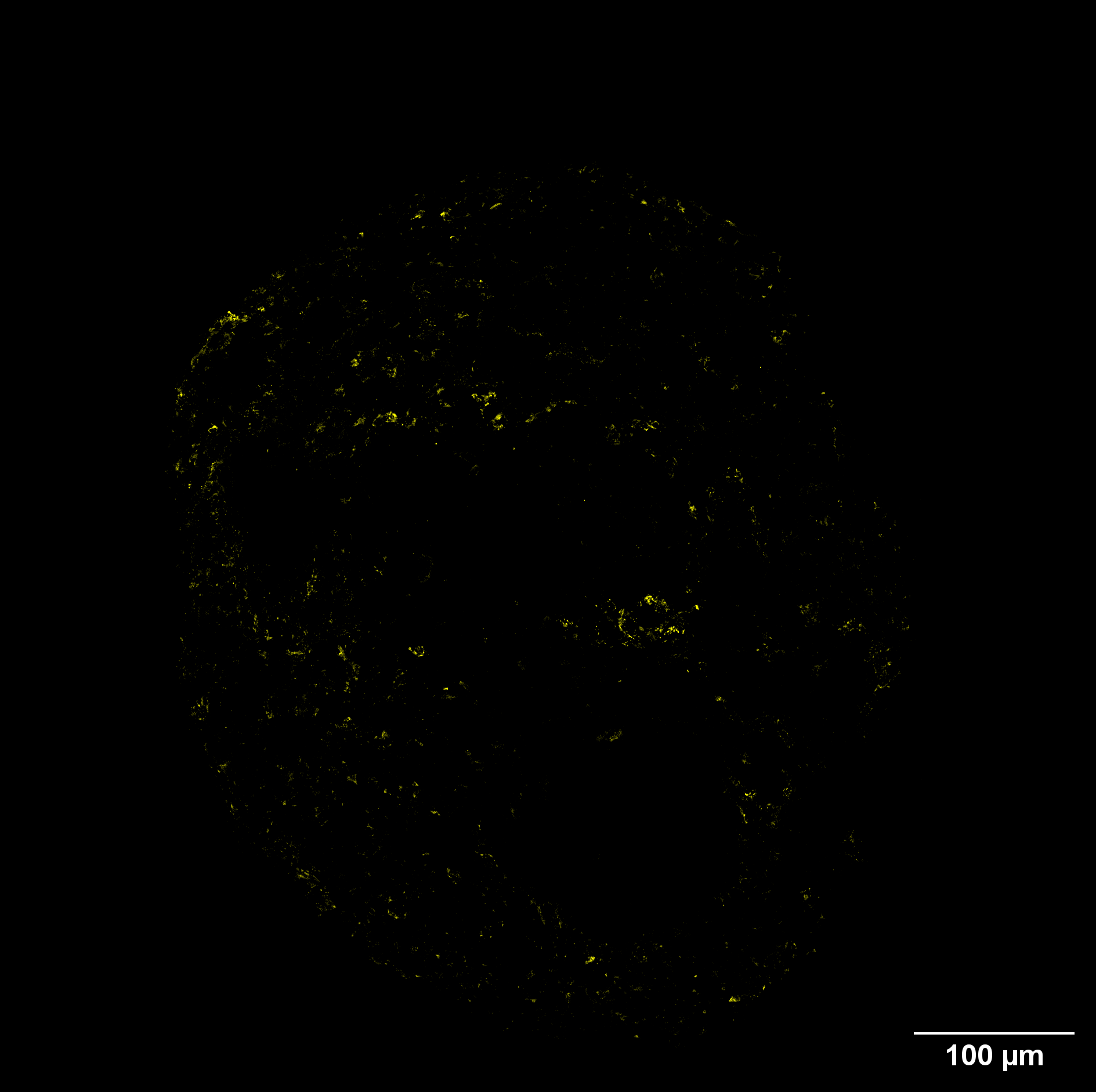

Supplement: Supplementary file 6 — Source data Fig. 6 [file 44320_2025_172_MOESM6_ESM.zip › Figure6/6H/H_SMAD2_TET_RIGHT/bottom/Smad_tet_col1a1.tif]

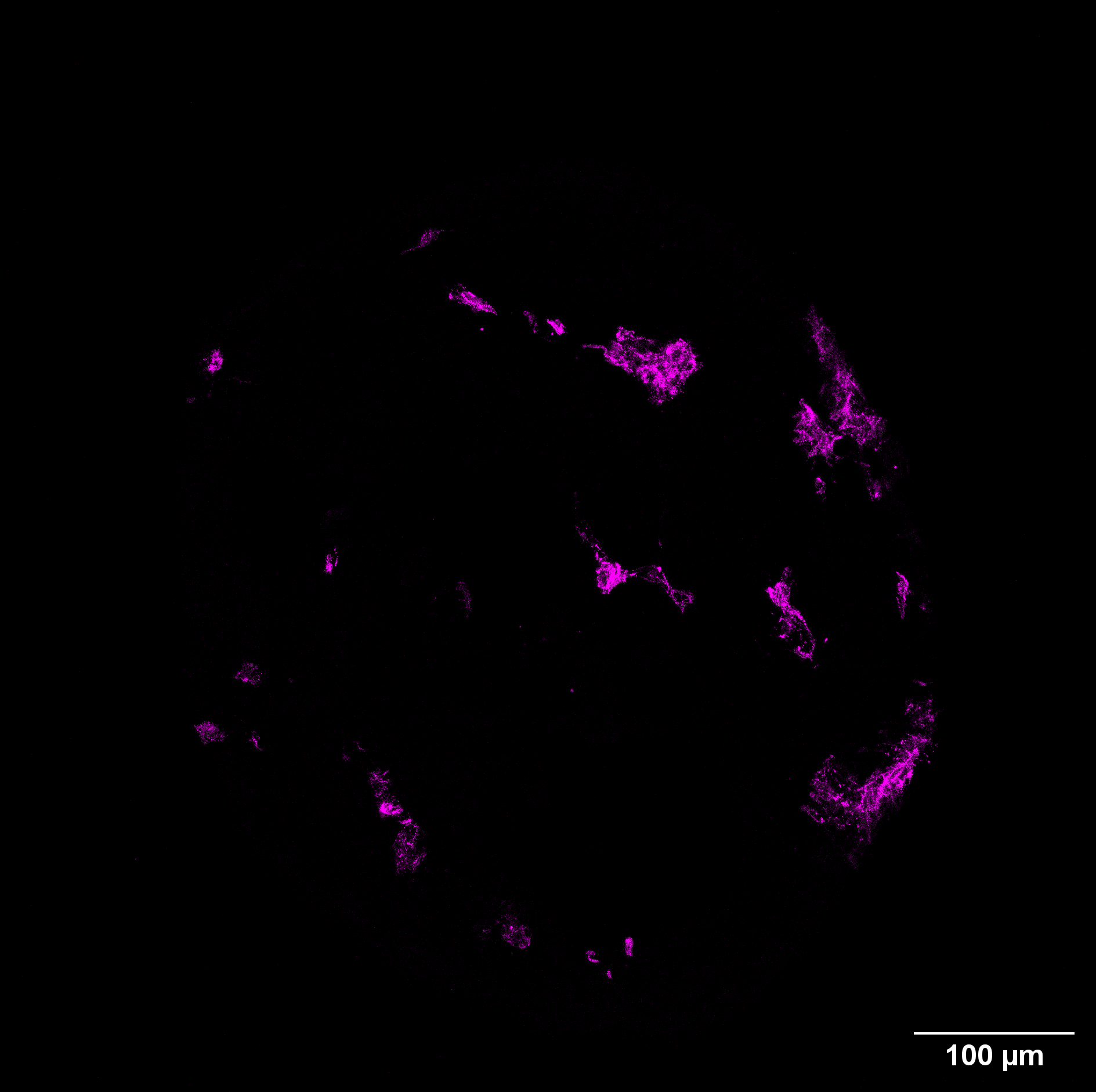

Supplement: Supplementary file 6 — Source data Fig. 6 [file 44320_2025_172_MOESM6_ESM.zip › Figure6/6H/H_SMAD2_TET_RIGHT/bottom/Smad_tet_aAct.tif]

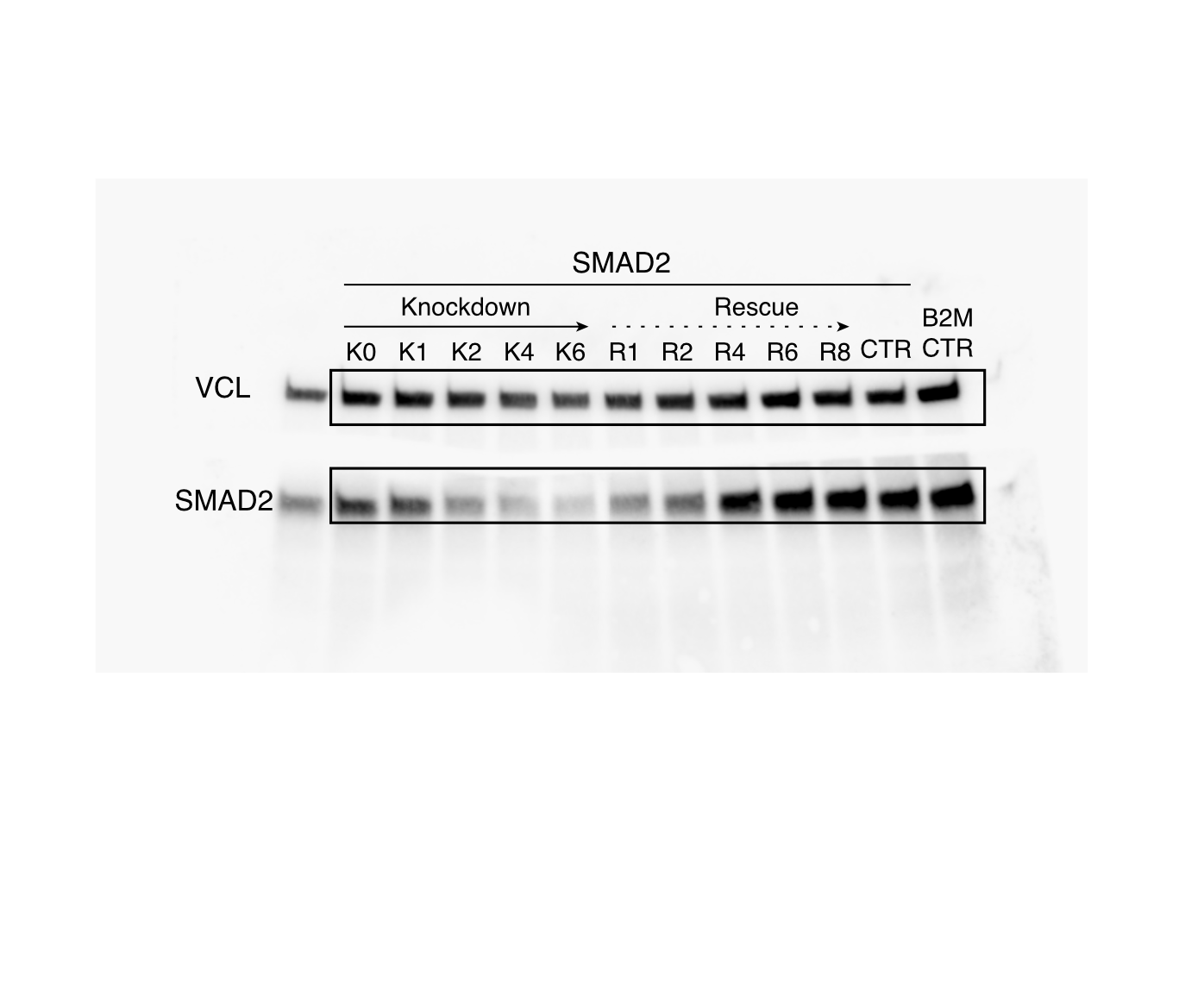

Supplement: Supplementary file 7 — Source data Fig. 7 [file 44320_2025_172_MOESM7_ESM.zip › Figure7/7C/Figure7C western blot.tiff]
