# Supplementary material for: Single cell transcriptional perturbome in pluripotent stem cell models
Source: Mol Syst Biol. 2025 Dec 10;22(2):179–227. doi: 10.1038/s44320-025-00172-8 (PMC12864791; doi:10.1038/s44320-025-00172-8)
Supplement: Supplementary file 6 — Source data Fig. 6 [file 44320_2025_172_MOESM6_ESM.zip › Figure6/6H/README.rtf]

Figures were not processed or manipulated, but only cropped to be placed in the figure panel H. In these folders, the full-size pictures were placed.Panel H SMAD2 CTR (LEFT):in folder H_SMAD2_CTR_LEFTTOP (WT1 staining:epicardial cells)_BW pictures are pictures with channels black and white_all_ch extension are pictures with all channels (ACTC1, WT1, HOLECHST)no extension are pictures with single channels in magenta (ACTC1), yellow (WT1) and blue (HOECHST)BOTTOM (COL1A1 staining:fibroblasts)_BW extension are pictures with channels black and white_all_ch extension are pictures with all channels (ACTC1, COL1A1, HOECHST)no extension are pictures with single channels in magenta (ACTC1), yellow (COL1A1) and blue (HOECHST)Panel H SMAD2 TET (RIGHT):in folder H_SMAD2_TET_RIGHTTOP (WT1 staining:epicardial cells)_BW pictures are pictures with channels black and white_all_ch extension are pictures with all channels (ACTC1, WT1, HOLECHST)no extension are pictures with single channels in magenta (ACTC1), yellow (WT1) and blue (HOECHST)BOTTOM (COL1A1 staining:fibroblasts)_BW extension are pictures with channels black and white_all_ch extension are pictures with all channels (ACTC1, COL1A1, HOECHST)no extension are pictures with single channels in magenta (ACTC1), yellow (COL1A1) and blue (HOECHST)
