# Supplementary material for: Single cell transcriptional perturbome in pluripotent stem cell models
Source: Mol Syst Biol. 2025 Dec 10;22(2):179–227. doi: 10.1038/s44320-025-00172-8 (PMC12864791; doi:10.1038/s44320-025-00172-8)
Supplement: Supplementary file 9 — Expanded View Figures [file 44320_2025_172_MOESM9_ESM.pdf]

Expanded View Figures

Figure EV1. Implementation of the iPS2-sci-seq pipeline.

(**A**) Diagram of the two pooled cloning steps to generate *AAVS1* targeting vectors containing matched shRNAs and UCI-BCs. H1-TO: tetracycline inducible Pol III promoter; CAG: constitutive promoter; HA: homology arm. (**B**) Representative efficiency of pooled cloning step 1 measured in individual bacterial clones through colony PCR. (**C**) As in (**B**) but for bacterial clones resulting from pooled cloning step 2. -ve: parental vector negative control. (**D**) Genotyping of hiPSC clones for the *AAVS1* locus (loss-of-allele implies biallelic targeting), the expected targeting cassette junctions (OPTtetR indicates inducible barcoded shRNA targeting; NeoR signals co-targeting of the second allele), and random shRNA plasmid integrations (which can convey sensitivity to FIAU); the number of shRNAs expressed by each surviving clone (no crosses) can be thus estimated. Refer to Appendix Fig. S1 for details on the optimization steps related to (**B–D**). (**E**) Diagram of the key molecular biology steps tested for and implemented in iPS2-sci-seq. Briefly, during reverse transcription (RT), shRNA UCI-BCs are both captured by the standard pT primer also used for the rest of the transcriptome (exemplified on the right) and specifically enriched by an OPTtetR-specific primer (tetR). Following second-strand synthesis (RT) and tagmentation with Tn5 (which is only needed for the rest of the transcriptome), UCI-BCs are enriched again during PCR. P7A primer is the one ultimately chosen for the final protocol; P7B was tested both in its full length and a truncated version with no overhangs (P7BT). At each step, primers introduce the overhangs required for the subsequent steps (color-coded). The three fragment types are processed in parallel (except for one of the tests described in Appendix Fig. S2), and the same RT indexes and PCR dual indexes (i5 and i7) are inserted in molecules from a given nucleus, out of a large variety of combinations. Following NGS, UCI-BC are matched to single nuclei sharing the same indexes. (**F**) Sequence of the tetR-primed UCI-BC library described in (**E**); the pT-primed UCI-BC library is equivalent except for a larger insert encompassing the ~109 bp bGH polyadenylation site and ~30 bp polyadenosine tail. Primers used for paired-end sequencing on Illumina instruments, with index reads based on the reverse complement workflow. Read 1 is exactly 18 bp, while read 2 is at least 52 bp to support parallel sequencing of the transcriptome. (**G**) Evaluation of hiPSC-CM purity through flow cytometry for cTnT (TNNT2) for the two batches of CMs utilized for the experiments of the rest of this figure and Fig. 1E–K. Gates set on isotype control. (**H**) Filtering of valid nuclei barcodes based on the number of associated UMIs in an iPS2-sci-seq experiment with ~9,600 input hiPSC-CM nuclei (half from each batch from (**G**)). The threshold was set at 500 UMIs. (**I**) Dimensionality reduction and clustering of single nuclei transcriptomes, separated by CM replicate (compare to Fig. 1H). CM1-3: three subsets of CMs; P-CM: proliferating CMs; CF: cardiac fibroblasts. (**J**) Aggregated list of top 5 gene markers per cluster determined with Monocle 3. (**K**) Expression patterns of selected cluster markers: *TNNT2*, pan CM; *NPPB*, early CM; *TNNI1* and *MYH6*, mid CM; *MYH7* and *RYR2*, mature CM; *MKI67*, proliferating cells; and *FBN1*, cardiac fibroblasts. (**L**) Expression of selected hits from scMAGeCK analyses of genes significantly associated with LoF perturbations among those in highly variable gene modules (Fig. 1I–K). Expression presented as log normalized for examples of genes negatively regulated (*CACNA1C*, particularly in *NKX2-5* LoF) or positively regulated (*CTNNA1* and *MYOZ2*, in *KMT2D* and *CHD7* LoF, respectively). Source data are available online for this figure.

 © The Author(s)

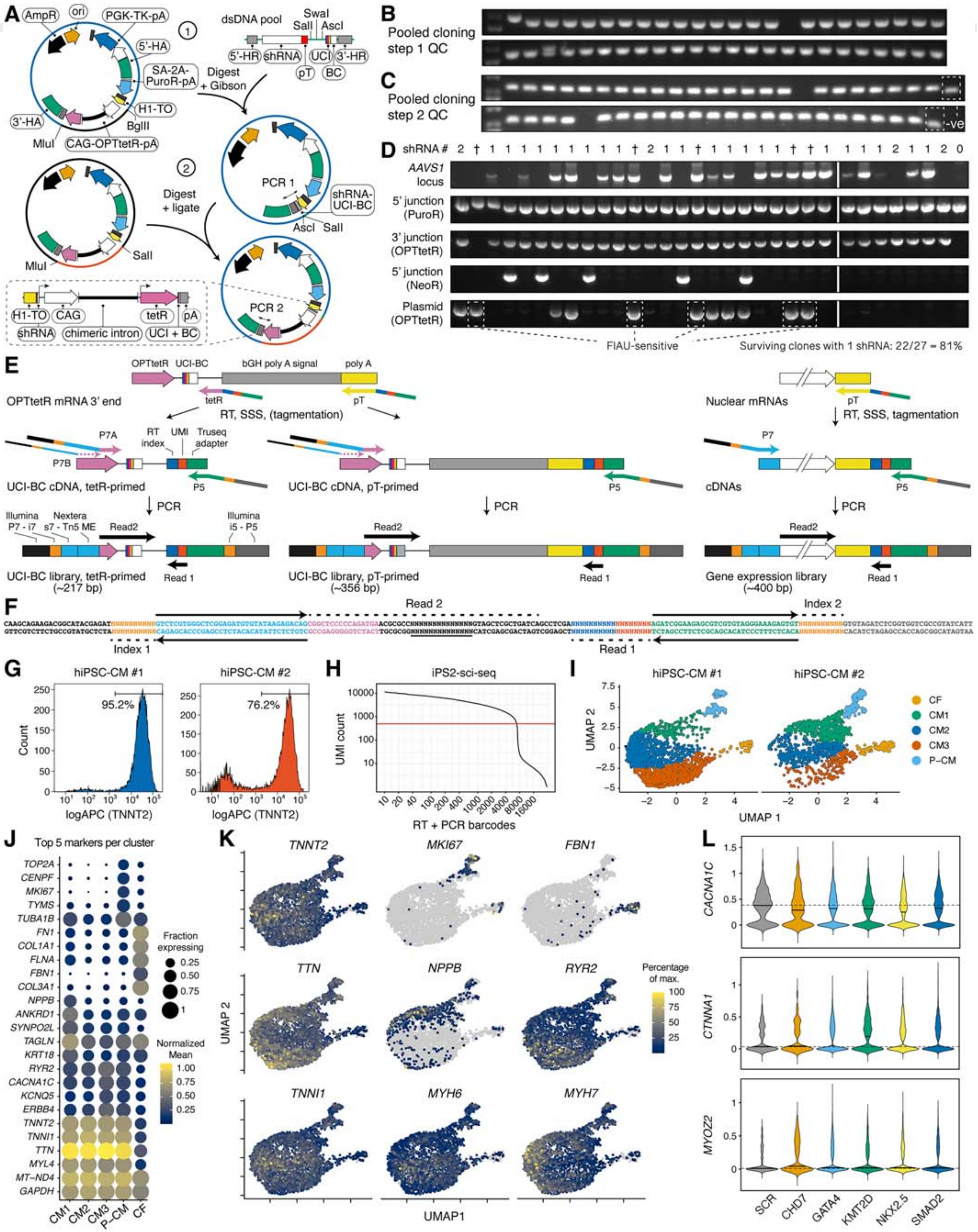

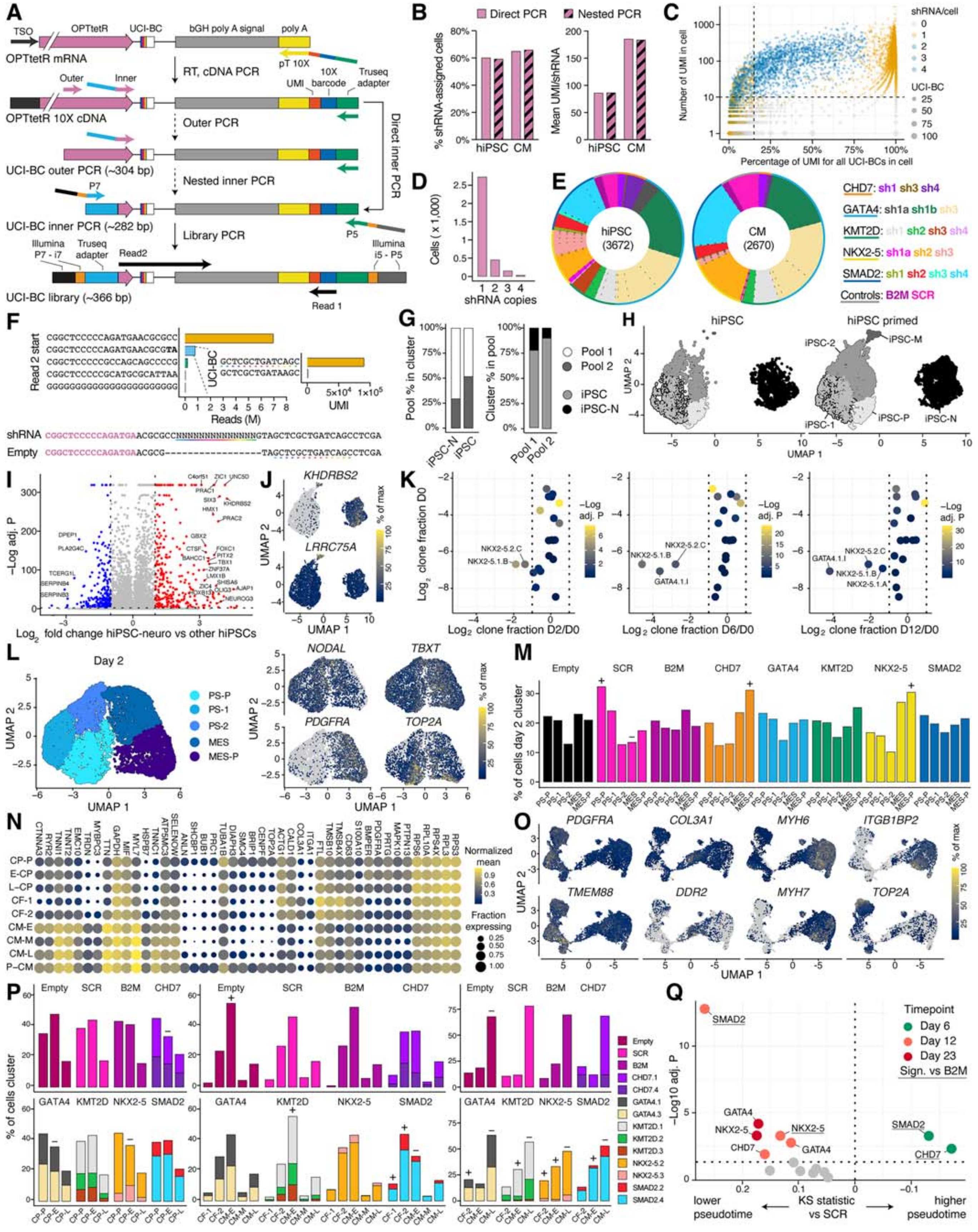
A
TSO
OPTtetR
UCI-BC
bGH poly A signal
poly A
OPTtetR mRNA
RT, cDNA PCR
pT 10X
Outer
Inner
UMI
10X barcode
Truseq adapter
OPTtetR 10X cDNA
Outer PCR
Direct inner PCR
UCI-BC outer PCR (~304 bp)
Nested inner PCR
P7
UCI-BC inner PCR (~282 bp)
Library PCR
P5
Illumina P7 - i7
Truseq adapter
Read2
Illumina i5 - P5
UCI-BC library (~366 bp)
Read 1
B
Direct PCR
Nested PCR
% shRNA-assigned cells
Mean UMI/shRNA
hiPSC
CM
C
Number of UMI in cell
Percentage of UMI for all UCI-BCs in cell
shRNA/cell
UCI-BC
D
Cells (x 1,000)
shRNA copies
E
hiPSC (3672)
CM (2670)
CHD7: sh1 sh3 sh4
GATA4: sh1a sh1b sh3
KMT2D: sh1 sh2 sh3 sh4
NKX2-5: sh1a sh2 sh3
SMAD2: sh1 sh2 sh3 sh4
Controls: B2M SCR
F
Read 2 start
UCI-BC
Reads (M)
UMI
shRNA CGGCTCCCCCAGATGAACGCGCCNNNNNNNNNNNNNNNGTAGCTCGCTGATCAGCCTCGA
Empty CGGCTCCCCCAGATGAACGCG---------------TAGCTCGCTGATCAGCCTCGA
G
Pool % in cluster
Cluster % in pool
Pool 1
Pool 2
iPSC
iPSC-N
H
hiPSC
hiPSC primed
iPSC-2
iPSC-M
iPSC-1
iPSC-P
iPSC-N
UMAP 1
UMAP 2
I
−Log adj. P
Log2 fold change hiPSC-neuro vs other hiPSCs
J
KHDRBS2
LRRC75A
% of max
K
Log2 clone fraction D0
Log2 clone fraction D2/D0
Log2 clone fraction D6/D0
Log2 clone fraction D12/D0
−Log adj. P
L
Day 2
PS-P
PS-1
PS-2
MES
MES-P
NODAL
TBXT
PDGFRA
TOP2A
M
Empty SCR B2M CHD7 GATA4 KMT2D NKX2-5 SMAD2
% of cells day 2 cluster
N
Normalized mean
Fraction expressing
CP-P
E-CP
L-CP
CF-1
CF-2
CM-E
CM-M
CM-L
P-CM
O
PDGFRA
COL3A1
MYH6
ITGB1BP2
TMEM88
DDR2
MYH7
TOP2A
P
% of cells cluster
Q
Timepoint
Day 6
Day 12
Day 23
Sign. vs B2M
−Log10 adj. P
KS statistic vs SCR
lower pseudotime
higher pseudotime

Figure EV2. Optimization and implementation of iPS2-10X-seq.

(**A**) Diagram of the molecular biology protocols tested for and implemented in iPSC-10X-seq. Briefly, an aliquot of 10X Genomics cell-barcoded and pre-amplified cDNA is processed to generate an shRNA UCI-BC library. UCI-BCs are amplified either in three steps ("nested" PCR with outer then inner primers, followed by NGS library prep), or in two steps ("direct" PCR with inner primer followed by NGS library prep). The resulting NGS libraries share the same structure, which is identical to the transcriptome-wide expression library obtained in parallel following the supplier's standard protocol, except that all read 2 sequences start in the same position of the OPTtetR 3' UTR (same sequence described in Fig. EV1F). UCI-BC reads are matched to their originating single cell through the 10X Genomics cell barcodes sequenced as part of read 1. Both strategies are effective, as described in (**B**), but since the direct PCR is more expedient it is our reference protocol. (**B**) Comparison of direct and nested PCR strategies to detect UCI-BCs in the same cDNA samples from hiPSCs and CMs, as assessed by the fraction of cells that could be assigned to individual shRNAs (*catcheR_10Xcatch* with UMI count threshold >10) and the mean UMI count for the relevant UCI-BCs. (**C**) Filtering of UCI-BC counts (UMIs); representative results in day 23 CMs (compare to Fig. 1E). (**D**) Quantification of shRNA expression per cell; representative results in day 23 CMs (compare to Fig. 1F). (**E**) Distribution of clones expressing individual shRNAs in hiPSCs and CMs (compare to Fig. 1C and Fig. 1G). (**F**) Assessment of read 2 types in the UCI-BC NGS library: besides the expected sequence upstream of UCI-BCs (shRNA), the second most common sequence matches the unmodified pAAV-Puro_siKD2.0 plasmid (Empty) and thus identifies integrations lacking an shRNA; representative results from day 23 CMs. (**G**) Relationship between genome editing pool of origin and segregation of hiPSC clones in two main transcriptional clusters (iPSC-N: neuroectoderm-primed hiPSCs, globally depleted during CM differentiation; Fig. 2C). (**H**) Subclustering of hiPSCs (Fig. 2B) based on sample of origin (hiPSCs: standard pluripotency media; hiPSC primed: day 0 of differentiation, following one day of mesoderm priming in pluripotency media supplemented with 1 μM CHIR99021); iPSC-P: proliferating iPSCs; iPSC-M: mesoderm-primed hiPSCs; iPSC-1/2: unbiased hiPSCs. (**I**) Differential gene expression analysis for iPSC-N vs. all other iPSC subclusters (adj. P by negative binomial test with B–H correction, significance threshold of 0.05, and absolute fold-change cutoff of 2). (**J**) Expression patterns of selected markers of hiPSC subclusters: *KHDRBS2*, early neuroectoderm; *LRRC75A*, early mesoderm (also see Fig. 2D). (**K**) Enrichment/depletion analysis for unbiased hiPSC clones (not in the hiPSC-N subcluster); day 23 in Fig. 2E. Adj. P by Fisher test with B–H correction. (**L**) Subclustering of CM differentiation day 2 cells (Fig. 2B) and expression patterns of selected markers; PS-1/2: primitive streak; PS-P: proliferative primitive streak; MES: mesoderm; MES-P: proliferative mesoderm. (**M**) Gene knockdown-associated cell clustering changes at day 2 of differentiation; + or − indicate significantly increased or decreased cluster representation (adj. $P < 0.05$ by Fisher test vs. *B2M* with B–H correction). (**N**) Aggregated list of top 5 gene markers per subcluster in cells from day 6, 12, and 23 of differentiation (Fig. 2G). (**O**) Expression patterns of selected markers of day 6 to day 23 subclusters: *PDGFRA* and *TMEM88*, cardiac progenitors; *COL3A1* and *DDR2*, cardiac fibroblasts; *MYH6*, early CM; *ITGB1BP2*, mid CM; *MYH7*, mature CM; and *TOP2A*, proliferating cells. (**P**) Gene knockdown-associated cell clustering changes at differentiation day 6 (left), 12 (middle), and 23 (right); + or - indicate significantly increased or decreased cluster representation (adj. $P < 0.05$ by Fisher test *vs.* SCR with B–H correction; individual shRNA shown just in reference to Fig. 2H); CP-P/E/L: proliferating/early/late cardiac progenitor; CF-1/2: early/late cardiac fibroblasts; CM-E/M/L: early/mid/late cardiomyocyte. (**Q**) Gene knockdown-associated pseudotime alterations at differentiation day 6, 12, and 23 (refer to Fig. 2I–K); adj. *P* by two-sided Kolmogorov–Smirnov (KS) test of pseudotime cumulative frequency *vs.* SCR with B–H correction and significance threshold of 0.05 (underlined clones also significant *vs. B2M*; *x* axis based on one-sided KS tests).

© The Author(s)

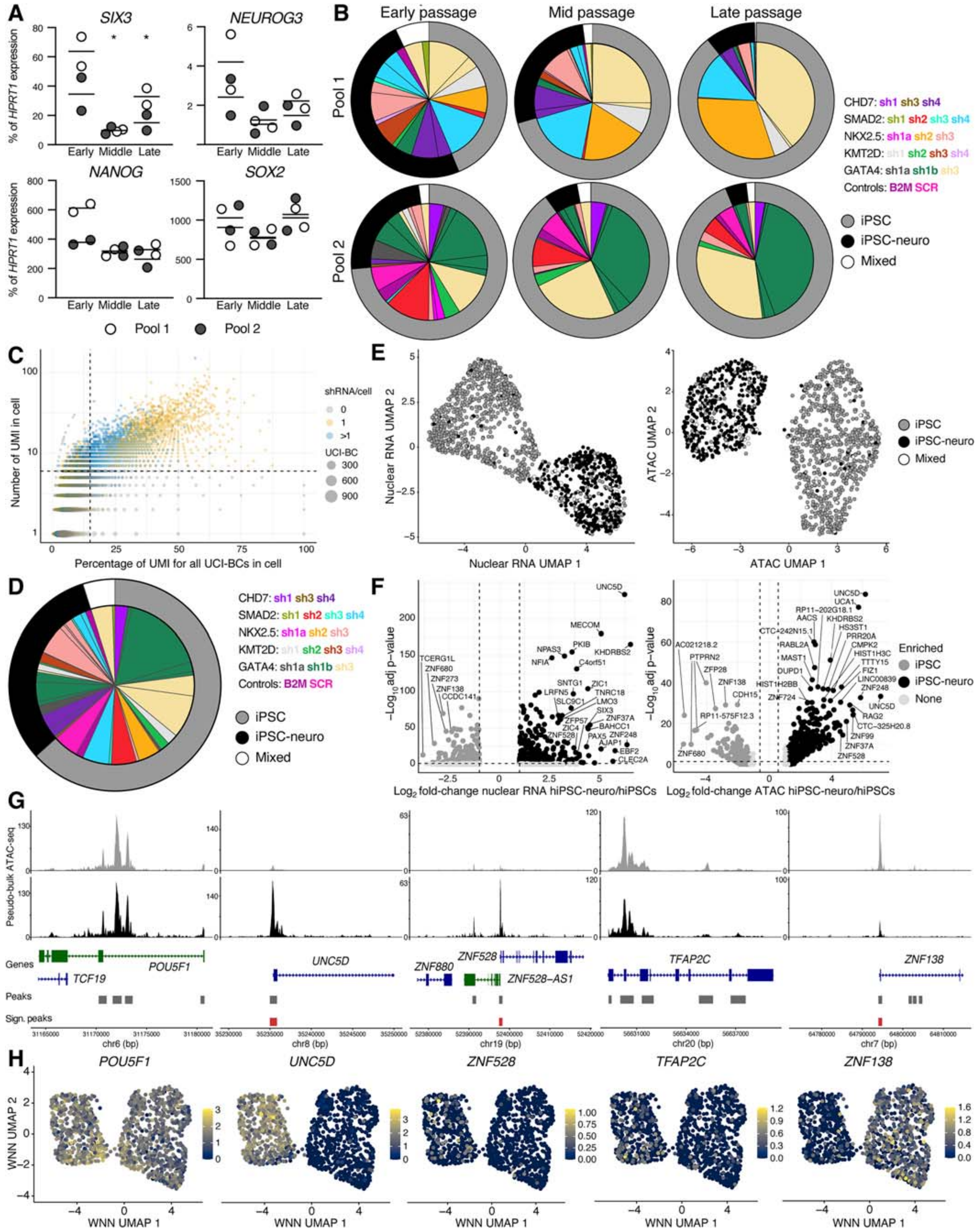
A
SIX3
NEUROG3
NANOG
SOX2
% of HPRT1 expression
Early Middle Late
Pool 1 Pool 2
B
Early passage
Mid passage
Late passage
Pool 1
Pool 2
CHD7: sh1 sh3 sh4
SMAD2: sh1 sh2 sh3 sh4
NKX2.5: sh1a sh2 sh3
KMT2D: sh1 sh2 sh3 sh4
GATA4: sh1a sh1b sh3
Controls: B2M SCR
iPSC
iPSC-neuro
Mixed
C
Number of UMI in cell
Percentage of UMI for all UCI-BCs in cell
shRNA/cell
UCI-BC
D
E
Nuclear RNA UMAP 1
Nuclear RNA UMAP 2
ATAC UMAP 1
ATAC UMAP 2
F
Log2 fold-change nuclear RNA hiPSC-neuro/hiPSCs
Log2 fold-change ATAC hiPSC-neuro/hiPSCs
−Log10 adj p-value
Enriched
None
G
Pseudo-bulk ATAC-seq
Genes
Peaks
Sign. peaks
POU5F1
TCF19
UNC5D
ZNF528
ZNF880
ZNF528-AS1
TFAP2C
ZNF138
chr6 (bp)
chr8 (bp)
chr19 (bp)
chr20 (bp)
chr7 (bp)
H
WNN UMAP 1
WNN UMAP 2

Figure EV3. Dissecting the epigenetic basis of hiPSC clonal biases by iPS2-multi-seq.

(**A**) Additional RT-qPCR of key regulators of neuroectoderm (*SIX3*, *NEUROG3*) and pluripotency (*NANOG*, *SOX2*) at various passages (early - p3, middle - p8, late - p13) of iPS2-seq genome-edited hiPSCs (see Fig. 3B). $N = 2$ cultures (the mean is indicated), * = adj. P of 0.031 and 0.022 vs. early, RM two-way ANOVA with Holm–Šídák's multiple comparisons. (**B**) Clonal composition for the two iPS2-seq hiPSC pools by UCI-BC DNA-seq across passages, plotted separately to show reproducibility of trends in Fig. 3C. Clones are ordered by clonal bias type (Fig. 2C). (**C**) Filtering of UCI-BC counts (UMIs) in the hiPSC pools analyzed at p3 by iPS2-multi-seq (Fig. 3A). (**D**) Clonal composition determined from iPSC-multi-seq, plotted as in (**B**). (**E**) Dimensionality reduction and clustering of matched nuclear RNA-seq (left) and ATAC-seq (right) from iPS2-multi-seq. Cells are color-coded by the clonal bias type (Fig. 2C). iPS-neuro: neuroectoderm-biased hiPSCs. (**F**) Volcano plots showing differential gene expression (left) and chromatin accessibility (right) between hiPSC-neuro and iPSCs from matched nuclear RNA-seq and ATAC-seq, respectively ($N = 1$). Wilcoxon Rank Sum test generated by *FindMarkers* function of Seurat package. (**G**) Aggregated chromatin accessibility tracks at exemplary loci showing no changes (*POU5F1/OCT4*, pluripotency marker; *TFAP2C*, neuroectoderm TF), increased accessibility in iPSC-neuro (*UNC5D*, neural differentiation marker; *ZNF528*, putative neuroectoderm TF), or increased accessibility in iPSC (*ZNF138*, putative iPSC TF). (**H**) Expression of the genes reported in (**G**) projected on WNN of integrated nuclear RNA-seq and ATAC-seq data (Fig. 3D). Source data are available online for this figure.

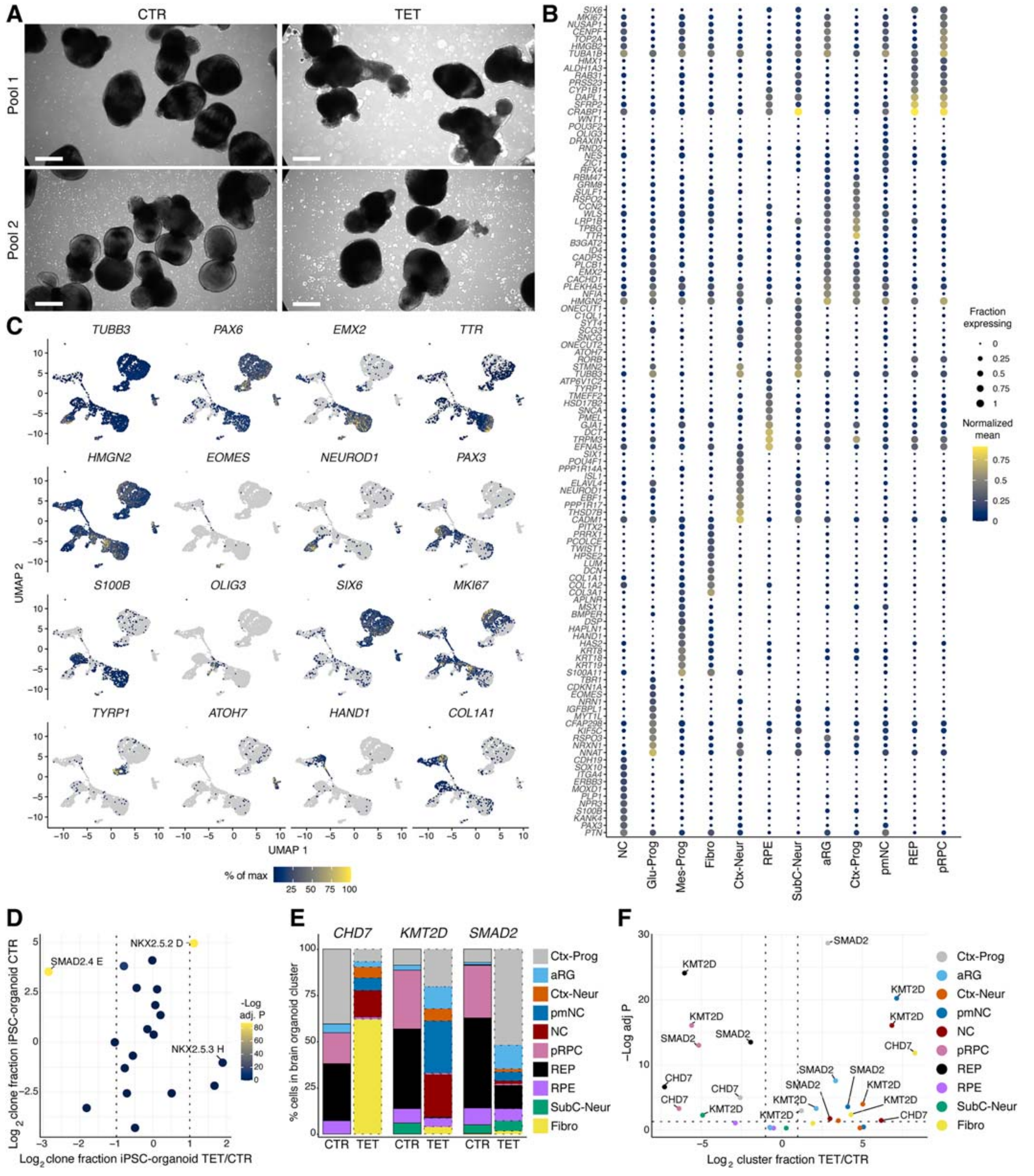
A
CTR
TET
Pool 1
Pool 2
B
SIX6
MKI67
NUSAP1
CENPF
TOP2A
HMGB2
TUBA1B
HMX1
ALDH1A3
RAB31
PRSS23
CYP1B1
DAPL1
SFRP2
CRABP1
WNT1
POU3F2
OLIG3
DRAXIN
RND2
NES
ZIC1
RFX4
RBM47
GRM8
SULF1
RSPO2
WLS
LRP1B
TPBG
TTR
B3GAT2
ID4
CADPS
PLCB1
EMX2
CACHD1
PLEKHA5
NFIA
HMGN2
ONECUT1
C1QL1
SYT4
SCG3
SNCG
ONECUT2
ATOH7
RORB
STMN2
TUBB3
ATP6V1C2
TYRP1
TMEFF2
HSD17B2
SNCA
PMEL
GJA1
DCT
TRPM3
EFNA5
SIX1
POU4F1
PPP1R14A
ISL1
ELAVL4
NEUROD1
EBF1
PPP1R17
THSD7B
CADM1
PITX2
PRRX1
PCOLCE
TWIST1
HPSE2
LUM
DCN
COL1A1
COL1A2
COL3A1
APLNR
MSX1
BMPER
DSP
HAPLN1
HAND1
HAS2
KRT8
KRT18
KRT19
S100A11
TBR1
CDKN1A
EOMES
NRN1
IGFBPL1
MYT1L
CFAP298
KIF5C
RSPO3
NRXN1
NNAT
CDH19
SOX10
ITGA4
ERBB3
MOXD1
PLP1
NPR3
S100B
KANK4
PAX3
PTN
NC
Glu-Prog
Mes-Prog
Fibro
Ctx-Neur
RPE
SubC-Neur
aRG
Ctx-Prog
pmNC
REP
pRPC
Fraction expressing
0
0.25
0.5
0.75
1
Normalized mean
0.75
0.5
0.25
0
C
TUBB3
PAX6
EMX2
TTR
HMGN2
EOMES
NEUROD1
PAX3
S100B
OLIG3
SIX6
MKI67
TYRP1
ATOH7
HAND1
COL1A1
UMAP 2
UMAP 1
% of max
25 50 75 100
D
SMAD2.4 E
NKX2.5.2 D
NKX2.5.3 H
-Log adj. P
80
60
40
20
0
Log2 clone fraction iPSC-organoid CTR
Log2 clone fraction iPSC-organoid TET/CTR
E
CHD7
KMT2D
SMAD2
% cells in brain organoid cluster
CTR TET
Ctx-Prog
aRG
Ctx-Neur
pmNC
NC
pRPC
REP
RPE
SubC-Neur
Fibro
F
−Log adj P
Log2 cluster fraction TET/CTR
SMAD2
KMT2D
CHD7
Ctx-Prog
aRG
Ctx-Neur
pmNC
NC
pRPC
REP
RPE
SubC-Neur
Fibro

◀ **Figure EV4. hiPSC clonal biases alter cell fate in forebrain organoids.**

(**A**) Representative phase contrast images of day 23 forebrain organoids from two iPS2-seq genome-edited hiPSC pools under control and tet-treated conditions, illustrating reproducible morphogenesis. Retinal and cortical regions appear as dark and light multilayered zones, respectively; tet treatment impacts overall structure. Scale bars: 1 mm. (**B**) Aggregated list of top 10 gene markers per cluster from iPS2-10X-seq of day 30 forebrain organoids (Fig. 4C). Ctx-Prog: cortical progenitors; aRG: apical radial glia; Glut-Prog: glutamatergic progenitors; Ctx-Neur: cortical neurons; pmNC: pre-migratory neural crest; NC: neural crest; pRPC: proliferative retinal progenitor cells; REP: retinal epithelial progenitors; RPE: retinal pigmented epithelium; SubC-Neur: sub-cortical neurons; Mes-Prog: mesoderm progenitors; Fibro: fibroblast. (**C**) Expression patterns of selected markers: *TUBB3*, pan-neuronal; *PAX6*, neuroectoderm and retina; *EMX2*, dorsal telencephalon; *TTR*, Ctx-Prog; *HMGN2*, aRG; *EOMES*, Glut-Prog; *NEUROD1*, Ctx-Neur; *PAX3*, pan-NC; *S100B*, NC; *OLIG3*, pmNC; *SIX6*, pan-retinal; *MKI67*, proliferating cells (e.g., pRPC); *TYRP1*, RPE; *ATOH7*, SubC-Neur; *HAND1*, Mes-Prog; *COL1A1*, Fibro. (**D**) Clone-level enrichment/depletion in control *vs*. tet treatment for unbiased hiPSC clones Fig. 4D. Adj. *P* by Fisher test with B–H correction. (**E**) Paired clustering representation changes after tet-induced knockdown of genes expressed in forebrain. (**F**) Statistical analysis of clustering changes in (**E**); adj. *P* by Fisher test comparing tet *vs*. control, B–H correction, significance threshold of 0.05. Source data are available online for this figure.

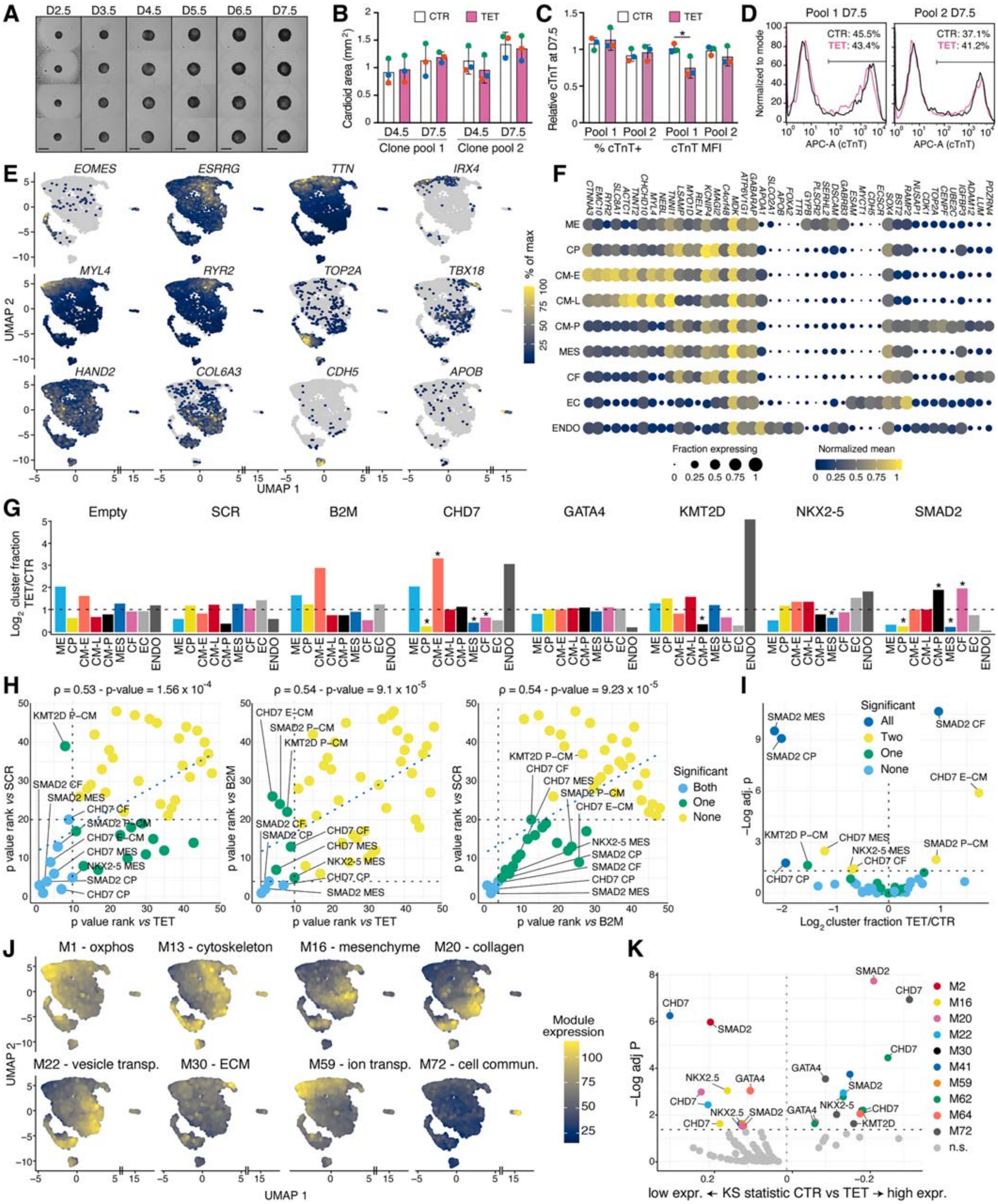
A
D2.5 D3.5 D4.5 D5.5 D6.5 D7.5
B
CTR TET
Cardioid area (mm2)
D4.5 D7.5 D4.5 D7.5
Clone pool 1 Clone pool 2
C
Relative cTnT at D7.5
Pool 1 Pool 2 Pool 1 Pool 2
% cTnT+ cTnT MFI
D
Pool 1 D7.5 Pool 2 D7.5
CTR: 45.5% TET: 43.4%
CTR: 37.1% TET: 41.2%
Normalized to mode
APC-A (cTnT)
E
EOMES ESRRG TTN IRX4
MYL4 RYR2 TOP2A TBX18
HAND2 COL6A3 CDH5 APOB
UMAP 2
UMAP 1
% of max
F
ME CP CM-E CM-L CM-P MES CF EC ENDO
Fraction expressing
Normalized mean
G
Empty SCR B2M CHD7 GATA4 KMT2D NKX2-5 SMAD2
Log2 cluster fraction TET/CTR
H
ρ = 0.53 - p-value = 1.56 x 10-4
ρ = 0.54 - p-value = 9.1 x 10-5
ρ = 0.54 - p-value = 9.23 x 10-5
p value rank vs SCR
p value rank vs TET
p value rank vs B2M
Significant
Both
One
None
I
Significant
All
Two
One
None
−Log adj. p
Log2 cluster fraction TET/CTR
J
M1 - oxphos M13 - cytoskeleton M16 - mesenchyme M20 - collagen
M22 - vesicle transp. M30 - ECM M59 - ion transp. M72 - cell commun.
Module expression
UMAP 2
UMAP 1
K
M2 M16 M20 M22 M30 M41 M59 M62 M64 M72 n.s.
−Log adj P
low expr. ← KS statistic CTR vs TET → high expr.

Figure EV5. Application of iPS2-10X-seq in cardiac organoids.

(**A**) Representative phase contrast images of left ventricular cardiac organoids (cardioids) from 24 h post aggregation to endpoint (Fig. 5A); scale bars: 1 mm. (**B**) Size of cardioids differentiated from two pools of iPS2-seq genome-edited hiPSCs in control conditions or tet-treated; data from 3 differentiations each with $N = 8$ cardioids/condition (color-coded; no significant changes for CTR *vs*. TET). Error bars represent mean ± SD. (**C**) Differentiation efficiency of cardioids from the same experiments as (**B**) (fraction of cTnT+ cells and cTnT median fluorescence intensity, MFI, in cTnT+ cells, both calculated relative to the average values for control conditions in each differentiation, $N = 3$); * = adj. *P* of 0.0284 by RM two-way ANOVA with Holm-Šídák's multiple comparisons. (**D**) cTnT flow cytometry for cardioids from one of the experiments described in (**B**, **C**) that was analyzed by iPS2-10X-seq. (**E**) Expression patterns of selected cluster markers: *EOMES*, primitive streak; *ESRRG*, cardiac progenitors; *TTN*, pan-CMs; *IRX4*, ventricular CMs; *MYL4*, late CMs; *RYR2*, early CMs; *TOP2A*, proliferating cells; *TBX18*, epicardium; *HAND2*, mesoderm; *COL6A3*, cardiac fibroblasts; *CDH5*, endothelial cells; *APOB*, endoderm derivatives. (**F**) Aggregated list of top 5 gene markers per cardioid cluster (Fig. 5B). (**G**) Gene knockdown-associated cell clustering changes; * = adj. $P < 0.05$ by Fisher test for tet vs. control with B–H correction. (**H**) Benchmarking of cell clustering differences by control type: tet-treated cells compared to clone-matched controls or to shRNA controls (SCR or *B2M*). Dots represent individual cluster–shRNA comparisons ranked by Fisher test *P* values; dotted lines mark adj. $P = 0.05$ (B–H corrected). Color coding denotes whether each result was confirmed, missed, or falsely called in the alternative comparison. (**I**) Replot of (**H**) showing significance of tet vs. matched control, with color indicating reproducibility across comparisons using alternative control shRNA (SCR or *B2M*). This design yields the highest proportion of true positives (significant in >2 comparisons) and lowest rate of false positives (significant in only one). Adj. *P* by Fisher test with B–H correction. (**J**) Aggregated expression of a subset of most variant gene modules (Fig. 5H–J). (**K**) Gene knockdown-associated gene module alterations; adj. *P* by two-sided KS test of module expression for tet *vs*. control with B–H correction and significance threshold of 0.05 (*x* axis based on one-sided KS tests). Source data are available online for this figure.

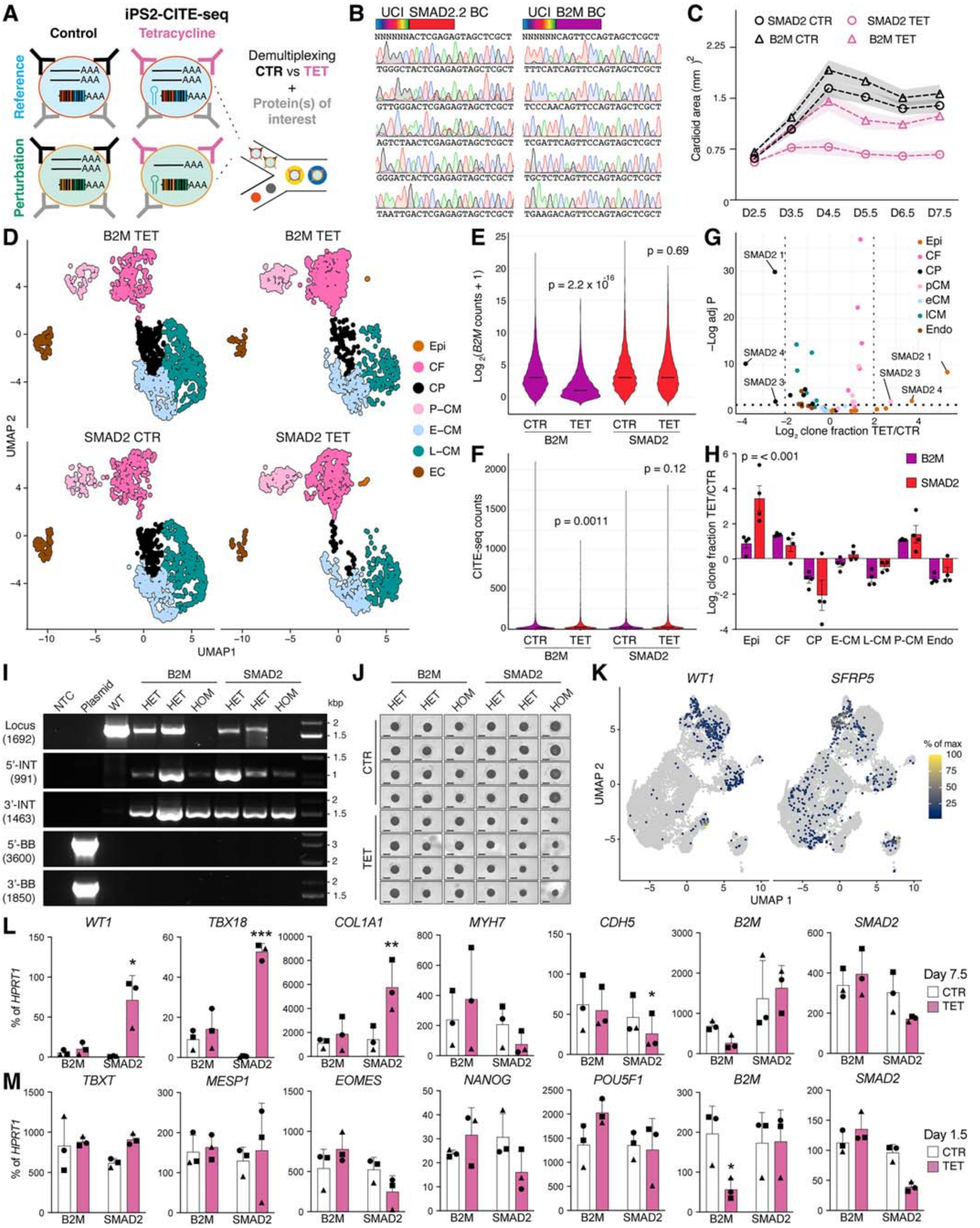
A
iPS2-CITE-seq
Control
Tetracycline
Reference
Perturbation
Demultiplexing
CTR vs TET
+
Protein(s) of interest
B
UCI SMAD2.2 BC
UCI B2M BC
C
SMAD2 CTR
SMAD2 TET
B2M CTR
B2M TET
Cardioid area (mm^2)
D2.5 D3.5 D4.5 D5.5 D6.5 D7.5
D
B2M TET
SMAD2 CTR
SMAD2 TET
UMAP 2
UMAP1
Epi
CF
CP
P–CM
E–CM
L–CM
EC
E
Log$_2$(B2M counts + 1)
p = 2.2 x 10^{-16}
p = 0.69
CTR TET CTR TET
B2M SMAD2
F
CITE-seq counts
p = 0.0011
p = 0.12
G
–Log adj P
Log$_2$ clone fraction TET/CTR
SMAD2 1
SMAD2 4
SMAD2 3
Epi
CF
CP
pCM
eCM
lCM
Endo
H
p = < 0.001
B2M
SMAD2
Log$_2$ clone fraction TET/CTR
Epi CF CP E-CM L-CM P-CM Endo
I
NTC Plasmid WT HET HET HOM HET HET HOM
kbp
Locus (1692)
5'-INT (991)
3'-INT (1463)
5'-BB (3600)
3'-BB (1850)
J
B2M SMAD2
HET HET HOM HET HET HOM
CTR
TET
K
WT1
SFRP5
% of max
UMAP 2
UMAP 1
L
WT1 TBX18 COL1A1 MYH7 CDH5 B2M SMAD2
% of HPRT1
Day 7.5
CTR
TET
M
TBXT MESP1 EOMES NANOG POU5F1 B2M SMAD2
Day 1.5

Figure EV6. ***SMAD2* knockdown impairs cardiac organoid morphodifferentiation.**

(**A**) iPS2-CITE-seq relies on antibodies against a housekeeping cell surface protein (e.g., anti-ATP1B3) barcoded with two different sequences (black and pink), to distinguish control and tet-treated conditions. A reference condition (e.g., *B2M* shRNA) and one or more perturbations of interest (e.g., *SMAD2* shRNA) can be analyzed altogether and distinguished based on UCI-BCs (Fig. 1A). Additional CITE-seq antibodies (e.g., anti-B2M, gray) can be added for multi-omic assessment of protein levels in parallel to perturbation-resolved single-cell transcriptomes. All conditions can be pooled on a single microfluidic channel to provide a cost-saving solution that also eliminates batch effects. (**B**) Sanger sequencing validation of UCI-BCs in pool-cloned plasmids (Fig. EV1A), employed to generate *SMAD2* and *B2M* iPS2-seq genome-edited hiPSCs for the experiments described in (**C–H**). (**C**) Time course analysis of cardioid size from clonal pools in control or tet-treated conditions. Representative data from one differentiation with $N = 24$ cardioids/condition. Day 7.5 cardioids were analyzed in (**D–H**). (**D**) Dimensionality reduction and clustering of control and tet-treated cardioids from clonal pools, shown separately. Epi indicates epicardial cells identified by expression of *TBX18*, *PDPN* (Fig. 6F), *WT1*, and *SFRP5* (**K**). Other cell type acronyms follow Fig. 5B and are annotated based on expression of markers listed in Fig. EV5E,F. (**E**) Violin plots of *B2M* mRNA expression quantified by scRNA-seq. adj. *P* vs. control by Wilcoxon signed-rank test, B–H corrected ($N = 1$ no TET control and $N = 1$ TET treated for each genotype). (**F**) As in (**E**), but for B2M protein expression quantified by CITE-seq ($N = 1$ no TET control and $N = 1$ TET treated for each genotype). (**G**) Quantification of clustering changes in individual clones (1–4); adj. *P* by Fisher test comparing tet vs. control with B–H correction and significance threshold of 0.05. Unlike pooled screens (e.g., Fig. 5E), an additional threshold of effect magnitude was applied for this arrayed counter-screen, as it was sufficiently powered on a per-clone basis (absolute fold change >4). (**H**) Alternative visualization of the data plotted in (**G**), with clones grouped by genotype to enable statistical assessment of intra-clonal reproducible clustering changes. Adj. $P < 0.001$ (***) by two-way ANOVA with Holm-Šídák's multiple comparisons. Error bars represent mean ± SEM. (**I**) Genotyping of *SMAD2* and *B2M* iPS2-seq clones with heterozygous (HET) or homozygous (HOM) integration of the inducible shRNA cassette, monitoring locus (loss-of-allele implies homozygous targeting), the expected targeting cassette junctions (5′/3′ integration, INT), and random shRNA plasmid integrations (5′/3′ backbone, BB). (**J**) Representative images of day 7.5 cardioids derived from the clones described in (**I**). Scale bars: 800 µm. (**K**) Expression of additional epicardial markers in day 7.5 tet-treated cardioids from the homozygous *SMAD2* iPS2-seq clone, projected on the dimensionality reduction from Fig. 6E. (**L**) RT-qPCR quantification of markers of epicardial cells (*WT1* and *TBX18*), cardiac fibroblasts (*COL1A1*), cardiomyocytes (*MYH7*), and endothelial cells (*CDH5*), in day 7.5 cardioids derived from homozygous *SMAD2* and *B2M* iPS2-seq clones. shRNA target expression is also reported. $N = 3$ differentiations (symbols); *, *, **, *** = adj. *P* 0.0103 (*WT1*), 0.0425 (*CDH5*), 0.005, <0.001 vs. control by two-way RM ANOVA with Holm-Šídák's multiple comparisons. Error bars represent mean ± SD. (**M**) As in (**L**), but for day 1.5 of the same differentiation runs (primitive streak stage), quantifying related markers (*TBXT/Brachyury*, *MESP1*, *EOMES*) and pluripotency genes (*NANOG*, *POU5F1/OCT4*), besides shRNA targets. * = adj. *P* 0.0446 vs. control by two-way RM ANOVA with Holm–Šídák's multiple comparisons. Error bars represent mean ± SD. Source data are available online for this figure.

© The Author(s)

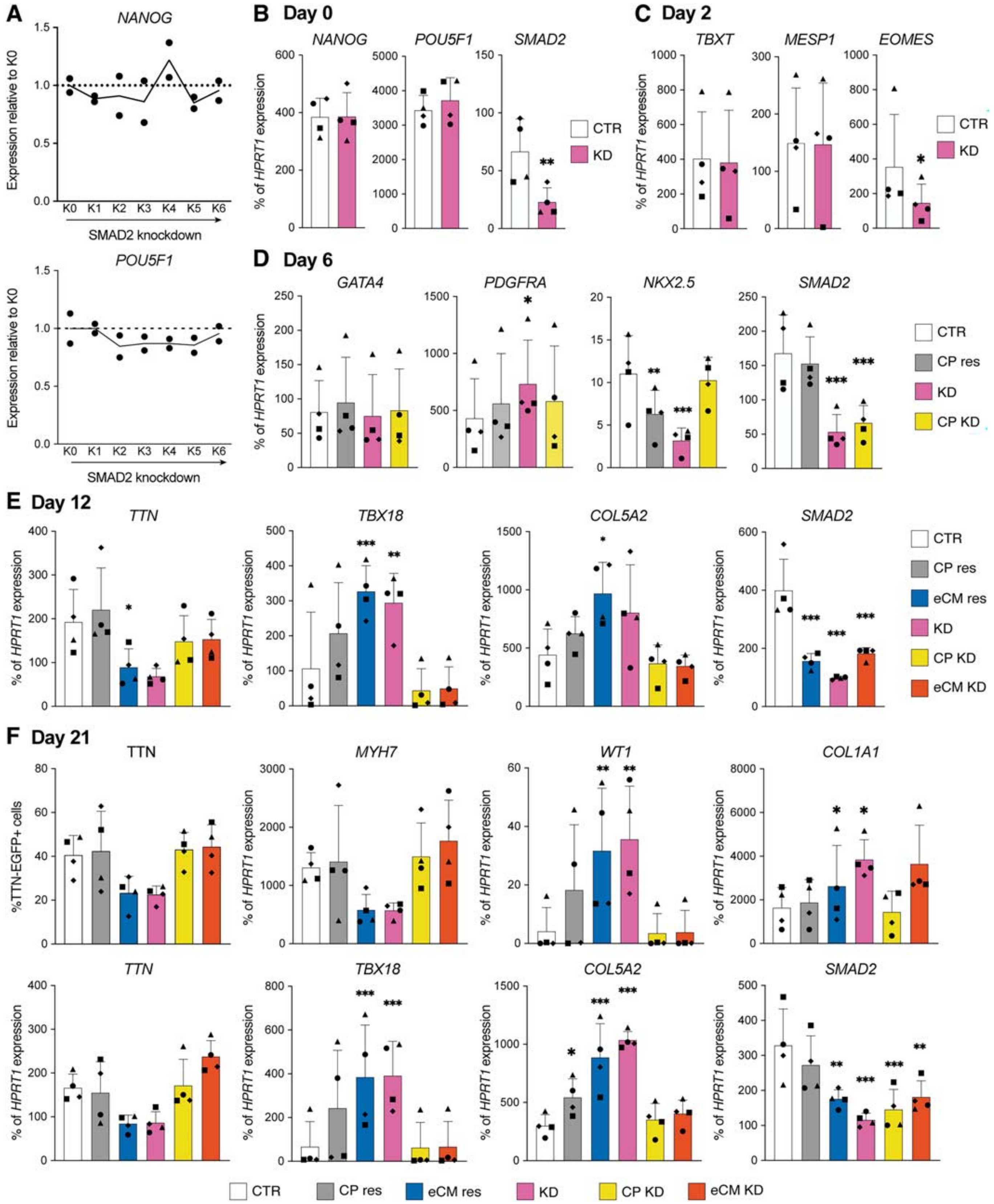
A
NANOG
POU5F1
Expression relative to K0
K0 K1 K2 K3 K4 K5 K6
SMAD2 knockdown
B Day 0
NANOG POU5F1 SMAD2
% of HPRT1 expression
CTR
KD
C Day 2
TBXT MESP1 EOMES
D Day 6
GATA4 PDGFRA NKX2.5 SMAD2
CTR
CP res
KD
CP KD
E Day 12
TTN TBX18 COL5A2 SMAD2
eCM res
eCM KD
F Day 21
TTN MYH7 WT1 COL1A1
%TTN-EGFP+ cells
TTN TBX18 COL5A2 SMAD2
CTR CP res eCM res KD CP KD eCM KD

 © The Author(s)

Figure EV7. *SMAD2* is required for cardiac progenitors patterning and specification.

(**A**) RT-qPCR of pluripotency markers during the time course of *SMAD2* silencing and re-expression in an iPS2-seq homozygous clone examined according to the strategy of Fig. 7A. $N = 2$ cultures. This clone was used throughout this figure. (**B**) RT-qPCR of pluripotency markers and shRNA target at day 0 of hiPSC-CM differentiation in the experiment depicted in Fig. 7D. All other panels in this figure refer to other time points of this same experiment, with $N = 4$ differentiations (symbols); *, **, *** = adj. $P < 0.05$, 0.01, 0.001 vs. control by 1-way RM ANOVA with Dunnett's multiple comparisons. For *SMAD2* P = 0.0017. Error bars represent mean ± SD. (**C**) As in (**B**), for day 2 of differentiation, examining primitive streak markers (*TBXT/Brachyury*, *MESP1*, *EOMES*). P = 0.0435, error bars represent mean ± SD. (**D**) As in (**B**), for day 6 of differentiation, examining cardiac progenitors markers (*GATA4*, *PDGFRA*) and an early cardiac commitment gene (*NKX2-5*). $P = 0.0435$ (*), 0.002 (**) or < 0.001 (***). Error bars represent mean ± SD. (**E**) As in (**B**), for day 12 of differentiation, expanding on data of Fig. 7E for additional markers of cardiomyocytes (*TTN*), epicardial cells (*TBX18*), and cardiac fibroblasts (*COL5A2*). $P = 0.023$ (* *TTN*), 0.015 (* *COL5A2*), 0.001 (**) or < 0.001 (***). Error bars represent mean ± SD. (**F**) As in (**B**), for day 21 of differentiation, examining markers of cardiomyocytes, epicardial cells, and cardiac fibroblasts, plus TTN-mEGFP reporter expression by flow cytometry (top left; compare to Fig. 7E and (**E**)). In order of appearance, $P = 0.005$ or 0.002 (** *WT1*), 0.019 or 0.010 (* *COL1A1*), 0.035 (* *COL5A2*), 0.001 or 0.002 (** *SMAD2*) or < 0.001 (***). Error bars represent mean ± SD. Source data are available online for this figure.

© The Author(s)
