# Supplementary material for: Single cell transcriptional perturbome in pluripotent stem cell models
Source: Mol Syst Biol. 2025 Dec 10;22(2):179–227. doi: 10.1038/s44320-025-00172-8 (PMC12864791; doi:10.1038/s44320-025-00172-8)
Supplement: Supplementary file 8 — EV Figure Source Data [file 44320_2025_172_MOESM8_ESM.zip › EV figures/FigEV1.zip!/FigEV1/EV1G/all_hystograms.pdf]

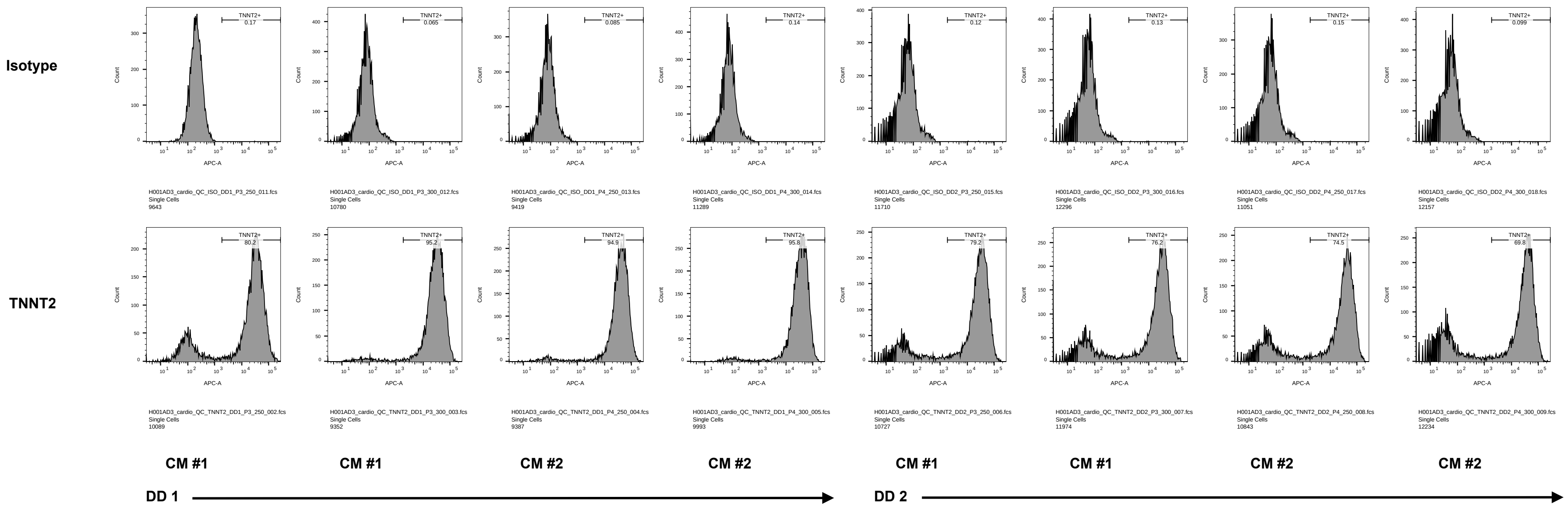
Isotype
TNNT2
TNNT2+
0.17
0.065
0.085
0.14
0.12
0.13
0.15
0.099
80.2
95.2
94.9
95.8
79.2
76.2
74.5
69.8
Count
APC-A
H001AD3_cardio_QC_ISO_DD1_P3_250_011.fcs
Single Cells
9643
H001AD3_cardio_QC_ISO_DD1_P3_300_012.fcs
Single Cells
10780
H001AD3_cardio_QC_ISO_DD1_P4_250_013.fcs
Single Cells
9419
H001AD3_cardio_QC_ISO_DD1_P4_300_014.fcs
Single Cells
11289
H001AD3_cardio_QC_ISO_DD2_P3_250_015.fcs
Single Cells
11710
H001AD3_cardio_QC_ISO_DD2_P3_300_016.fcs
Single Cells
12296
H001AD3_cardio_QC_ISO_DD2_P4_250_017.fcs
Single Cells
11051
H001AD3_cardio_QC_ISO_DD2_P4_300_018.fcs
Single Cells
12157
H001AD3_cardio_QC_TNNT2_DD1_P3_250_002.fcs
Single Cells
10089
H001AD3_cardio_QC_TNNT2_DD1_P3_300_003.fcs
Single Cells
9352
H001AD3_cardio_QC_TNNT2_DD1_P4_250_004.fcs
Single Cells
9387
H001AD3_cardio_QC_TNNT2_DD1_P4_300_005.fcs
Single Cells
9993
H001AD3_cardio_QC_TNNT2_DD2_P3_250_006.fcs
Single Cells
10727
H001AD3_cardio_QC_TNNT2_DD2_P3_300_007.fcs
Single Cells
11974
H001AD3_cardio_QC_TNNT2_DD2_P4_250_008.fcs
Single Cells
10843
H001AD3_cardio_QC_TNNT2_DD2_P4_300_009.fcs
Single Cells
12234
CM #1
CM #1
CM #2
CM #2
CM #1
CM #1
CM #2
CM #2
DD 1
DD 2
