# Supplementary material for: Single cell transcriptional perturbome in pluripotent stem cell models
Source: Mol Syst Biol. 2025 Dec 10;22(2):179–227. doi: 10.1038/s44320-025-00172-8 (PMC12864791; doi:10.1038/s44320-025-00172-8)
Supplement: Supplementary file 8 — EV Figure Source Data [file 44320_2025_172_MOESM8_ESM.zip › EV figures/FigEV6.zip!/FigEV6/EV6B/B2M_Final_pool.pdf]

Sequence: pKS003_pAAV-Puro_siKD_B2M_Final_pool.dna (Circular / 2750 bp)
Enzymes: Unique Cutters (46 of 686 total) Unique Cutters **Bold**
Features: 8 total
Primers: 6 total

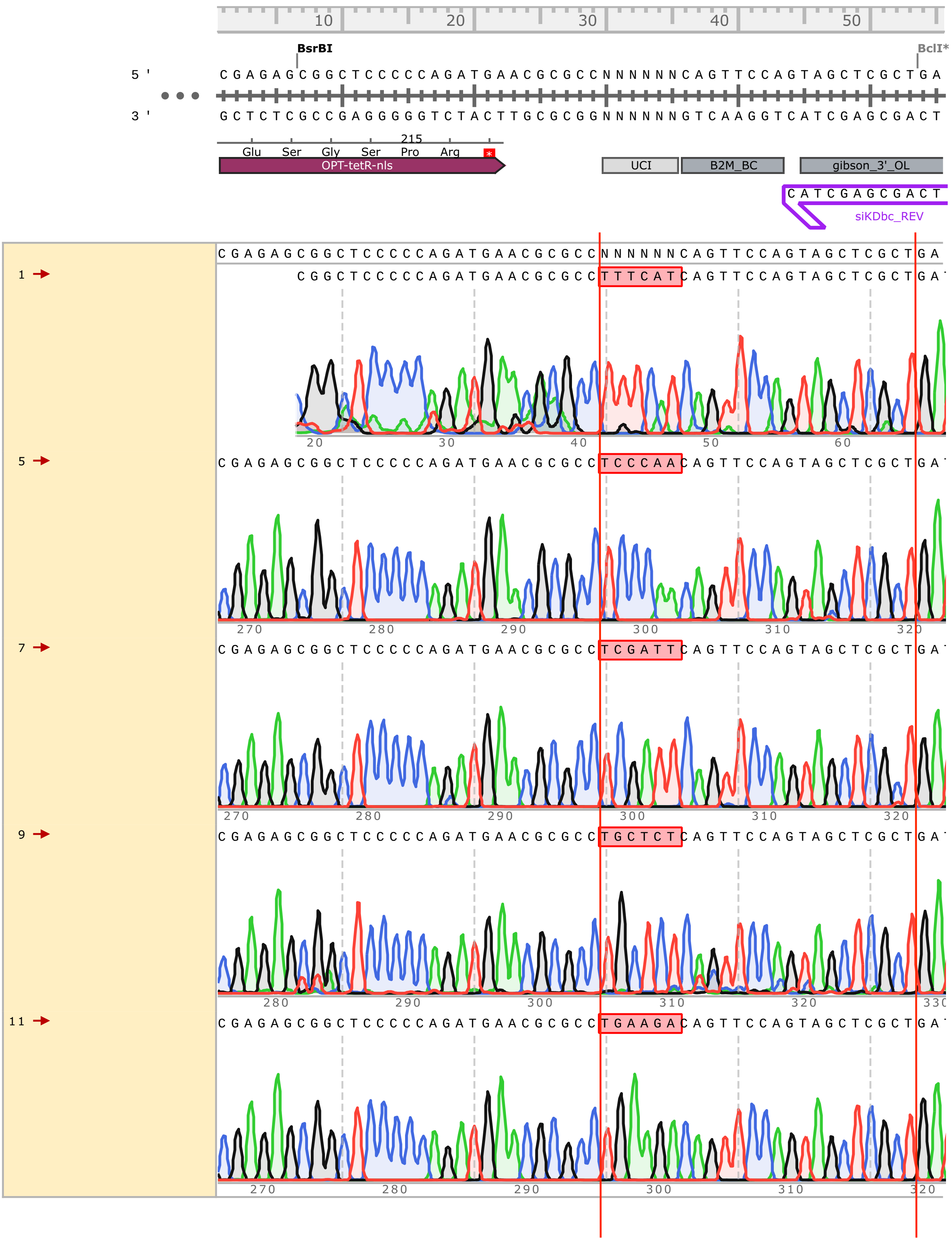

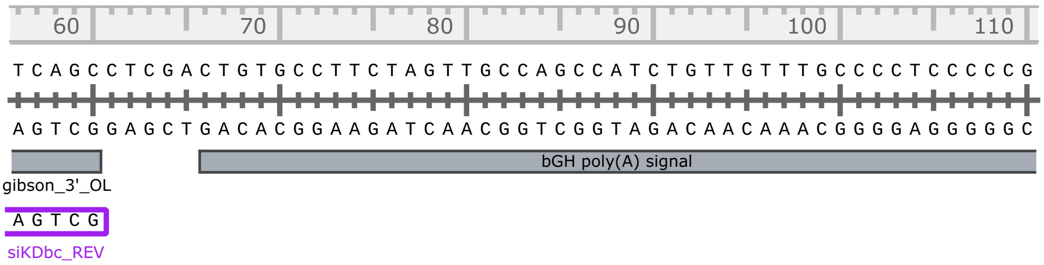
60
70
80
90
100
110
TCAGCCTCGACTGTGCCTTCTAGTTGCCAGCCATCTGTTGTTTGCCCCTCCCCCG
AGTCGGAGCTGACACGGAAGATCAACGGTCGGTAGACAACAAACGGGGAGGGGGC
gibson_3'_OL
bGH poly(A) signal
AGTCG
siKDbc_REV

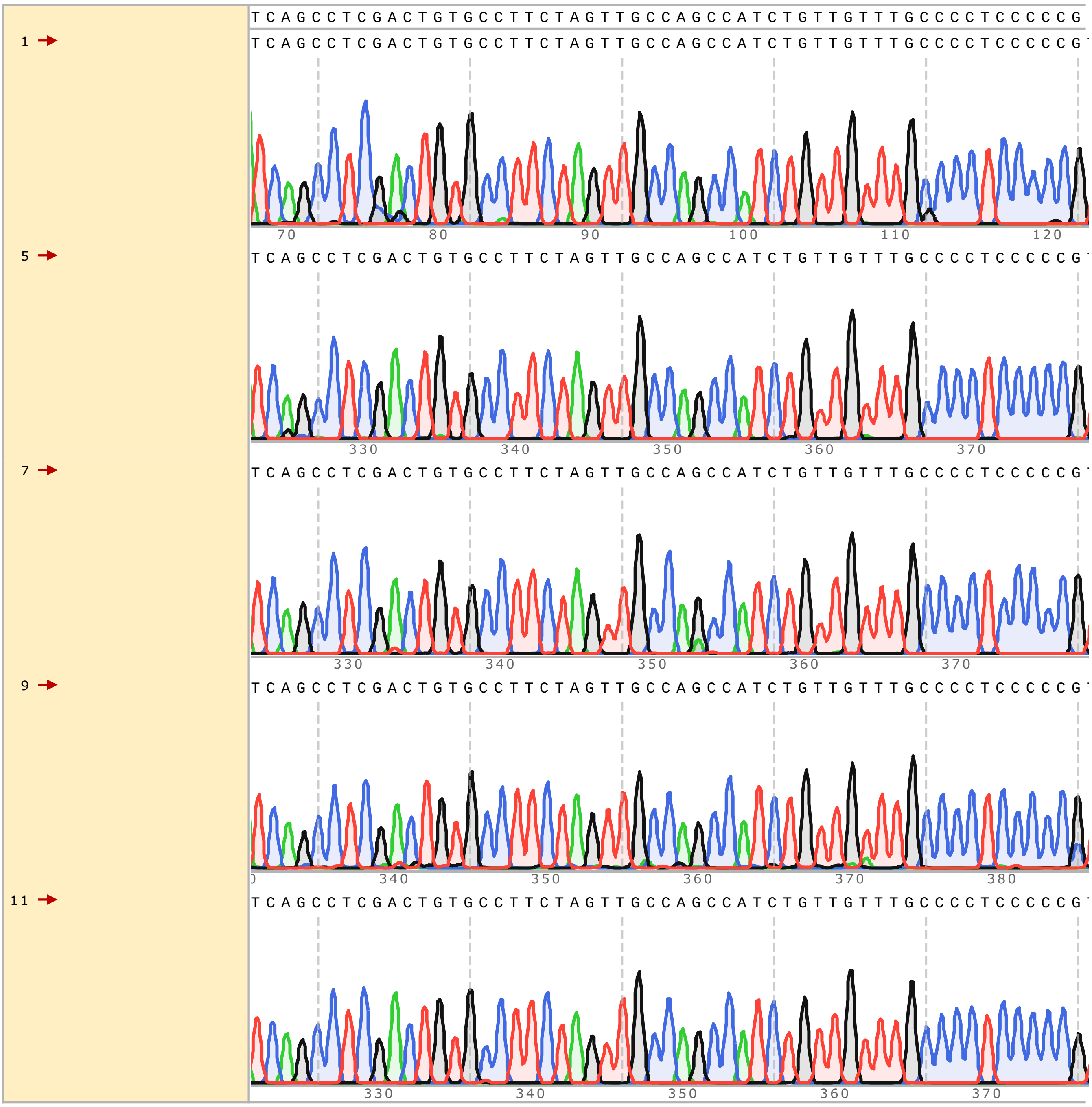
TCAGCCTCGACTGTGCCTTCTAGTTGCCAGCCATCTGTTGTTTGCCCCTCCCCCG
1
TCAGCCTCGACTGTGCCTTCTAGTTGCCAGCCATCTGTTGTTTGCCCCTCCCCCG
70 80 90 100 110 120
5
TCAGCCTCGACTGTGCCTTCTAGTTGCCAGCCATCTGTTGTTTGCCCCTCCCCCG
330 340 350 360 370
7
TCAGCCTCGACTGTGCCTTCTAGTTGCCAGCCATCTGTTGTTTGCCCCTCCCCCG
330 340 350 360 370
9
TCAGCCTCGACTGTGCCTTCTAGTTGCCAGCCATCTGTTGTTTGCCCCTCCCCCG
340 350 360 370 380
11
TCAGCCTCGACTGTGCCTTCTAGTTGCCAGCCATCTGTTGTTTGCCCCTCCCCCG
330 340 350 360 370

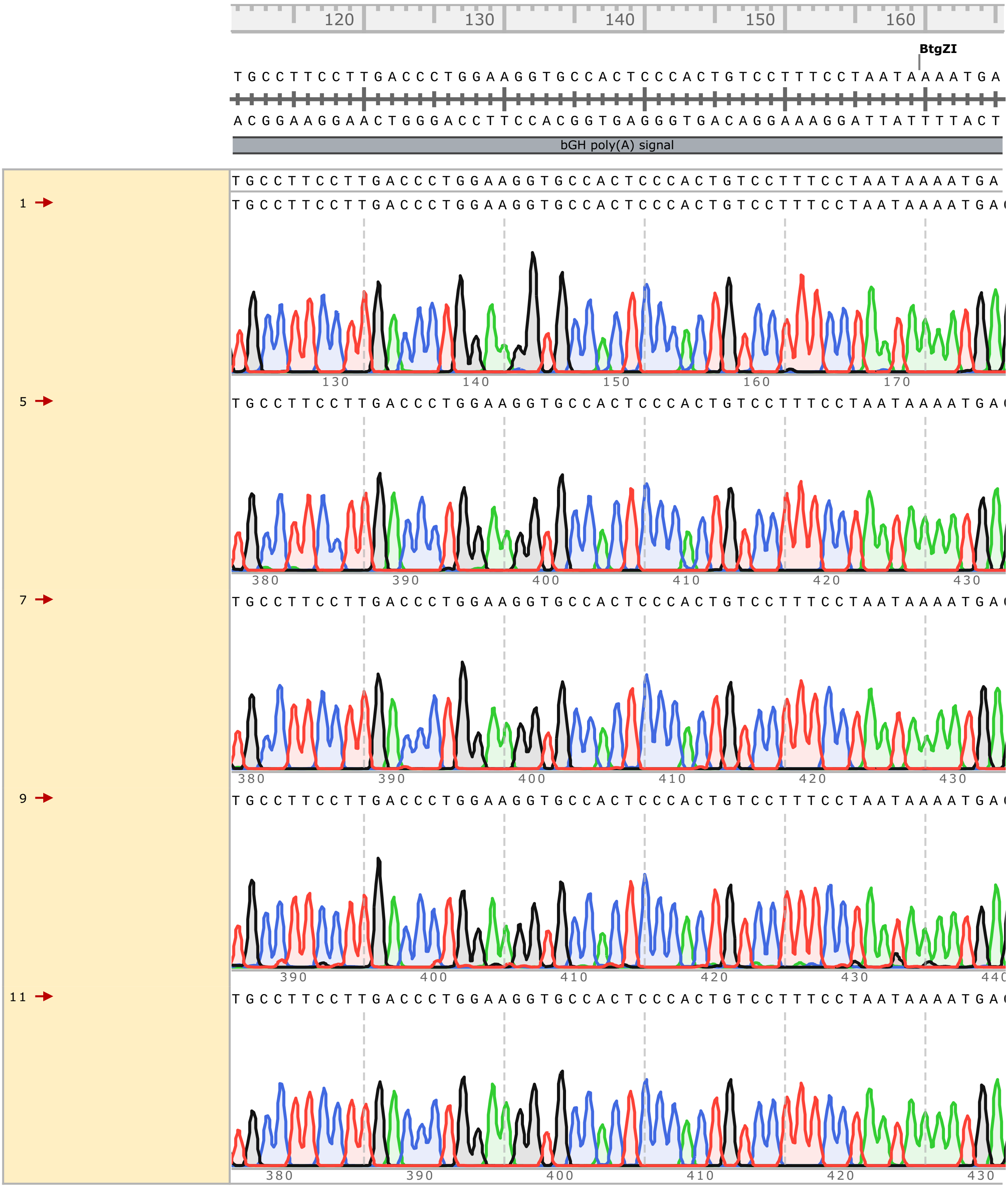
120 130 140 150 160
BtgZI
TGCCTTCCTTGACCCTGGAAGGTGCCACTCCCACTGTCCTTTCCTAATAAAATGA
ACGGAAGGAACTGGGACCTTCCACGGTGAGGGTGACAGGAAAGGATTATTTTACT
bGH poly(A) signal
TGCCTTCCTTGACCCTGGAAGGTGCCACTCCCACTGTCCTTTCCTAATAAAATGA
1
TGCCTTCCTTGACCCTGGAAGGTGCCACTCCCACTGTCCTTTCCTAATAAAATGA
130 140 150 160 170
5
TGCCTTCCTTGACCCTGGAAGGTGCCACTCCCACTGTCCTTTCCTAATAAAATGA
380 390 400 410 420 430
7
TGCCTTCCTTGACCCTGGAAGGTGCCACTCCCACTGTCCTTTCCTAATAAAATGA
380 390 400 410 420 430
9
TGCCTTCCTTGACCCTGGAAGGTGCCACTCCCACTGTCCTTTCCTAATAAAATGA
390 400 410 420 430 440
11
TGCCTTCCTTGACCCTGGAAGGTGCCACTCCCACTGTCCTTTCCTAATAAAATGA
380 390 400 410 420 430

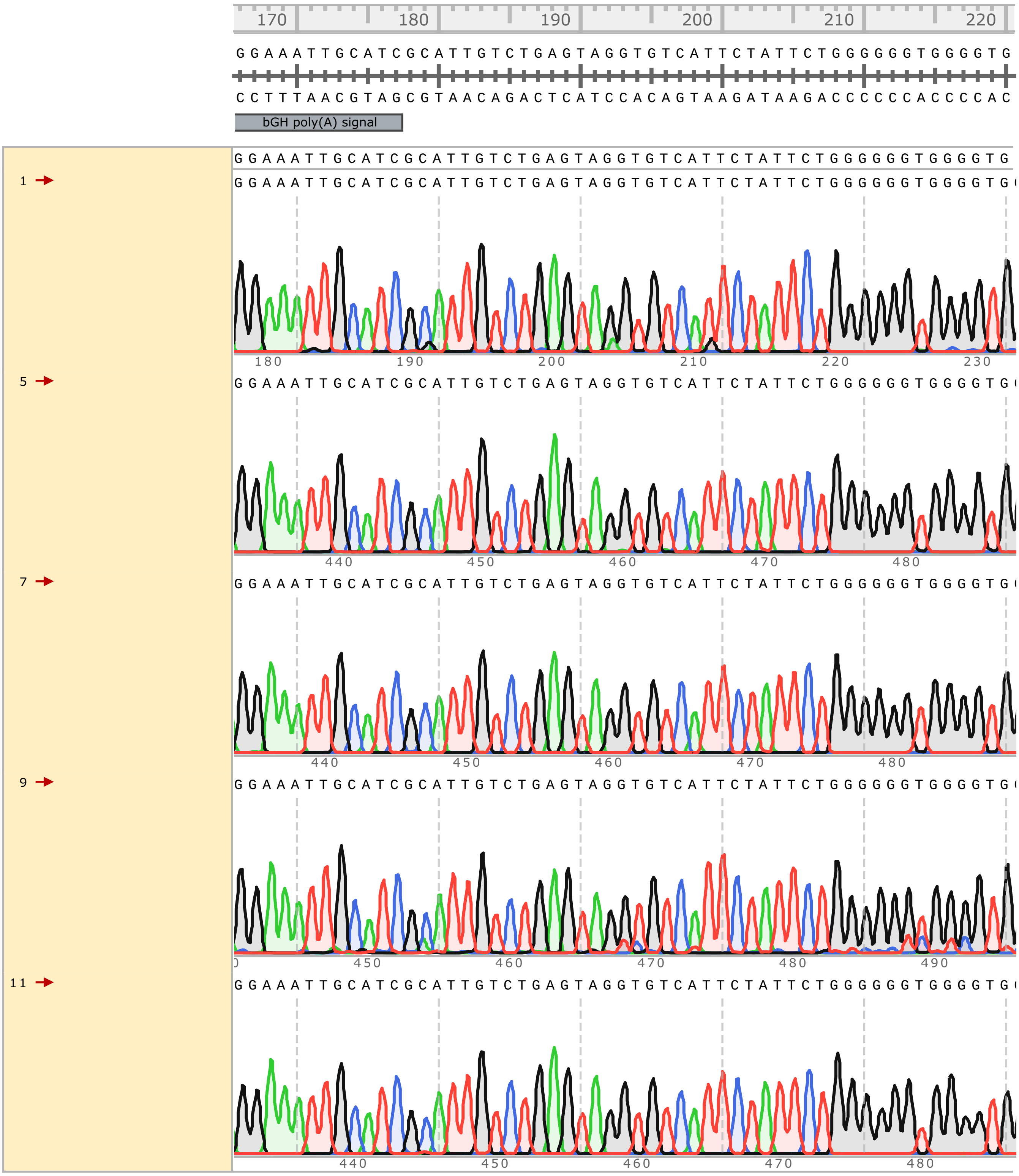
170
180
190
200
210
220
GGAAATTGCATCGCATTGTCTGAGTAGGTGTCATTCTATTCTGGGGGGTGGGGTG
CCTTTAACGTAGCGTAACAGACTCATCCACAGTAAGATAAGACCCCCCACCCCAC
bGH poly(A) signal
GGAAATTGCATCGCATTGTCTGAGTAGGTGTCATTCTATTCTGGGGGGTGGGGTG
1
GGAAATTGCATCGCATTGTCTGAGTAGGTGTCATTCTATTCTGGGGGGTGGGGTG
180 190 200 210 220 230
5
GGAAATTGCATCGCATTGTCTGAGTAGGTGTCATTCTATTCTGGGGGGTGGGGTG
440 450 460 470 480
7
GGAAATTGCATCGCATTGTCTGAGTAGGTGTCATTCTATTCTGGGGGGTGGGGTG
440 450 460 470 480
9
GGAAATTGCATCGCATTGTCTGAGTAGGTGTCATTCTATTCTGGGGGGTGGGGTG
450 460 470 480 490
11
GGAAATTGCATCGCATTGTCTGAGTAGGTGTCATTCTATTCTGGGGGGTGGGGTG
440 450 460 470 480

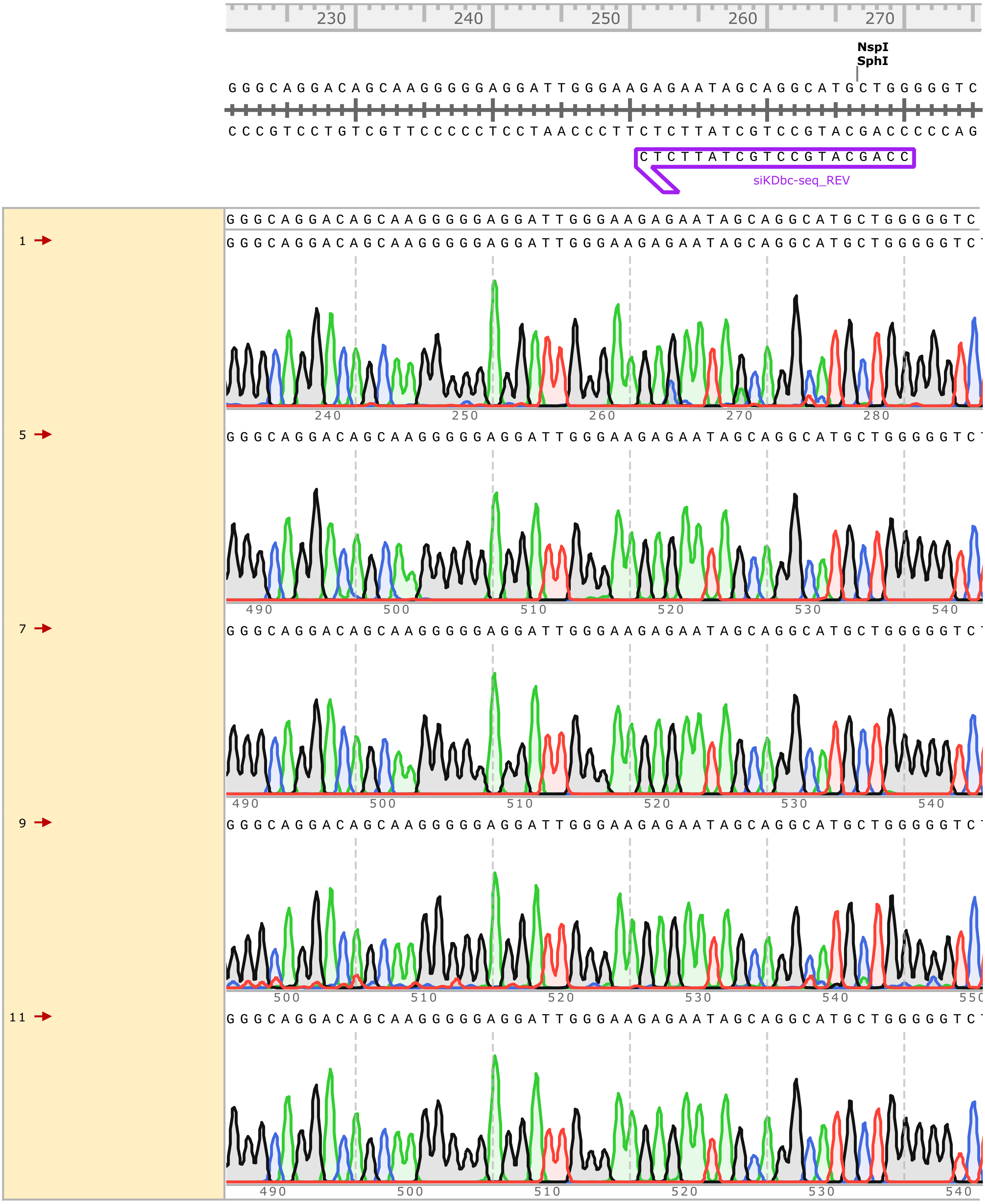
230 240 250 260 270
NspI
SphI
GGGCAGGACAGCAAGGGGGAGGATTGGGAAGAGAATAGCAGGCATGCTGGGGGTC
CCCGTCCTGTCGTTCCCCCTCCTAACCCTTCTCTTATCGTCCGTACGACCCCCAG
CTCTTATCGTCCGTACGACC
siKDbc-seq_REV
GGGCAGGACAGCAAGGGGGAGGATTGGGAAGAGAATAGCAGGCATGCTGGGGGTC
1
GGGCAGGACAGCAAGGGGGAGGATTGGGAAGAGAATAGCAGGCATGCTGGGGGTC
240 250 260 270 280
5
GGGCAGGACAGCAAGGGGGAGGATTGGGAAGAGAATAGCAGGCATGCTGGGGGTC
490 500 510 520 530 540
7
GGGCAGGACAGCAAGGGGGAGGATTGGGAAGAGAATAGCAGGCATGCTGGGGGTC
490 500 510 520 530 540
9
GGGCAGGACAGCAAGGGGGAGGATTGGGAAGAGAATAGCAGGCATGCTGGGGGTC
500 510 520 530 540 550
11
GGGCAGGACAGCAAGGGGGAGGATTGGGAAGAGAATAGCAGGCATGCTGGGGGTC
490 500 510 520 530 540

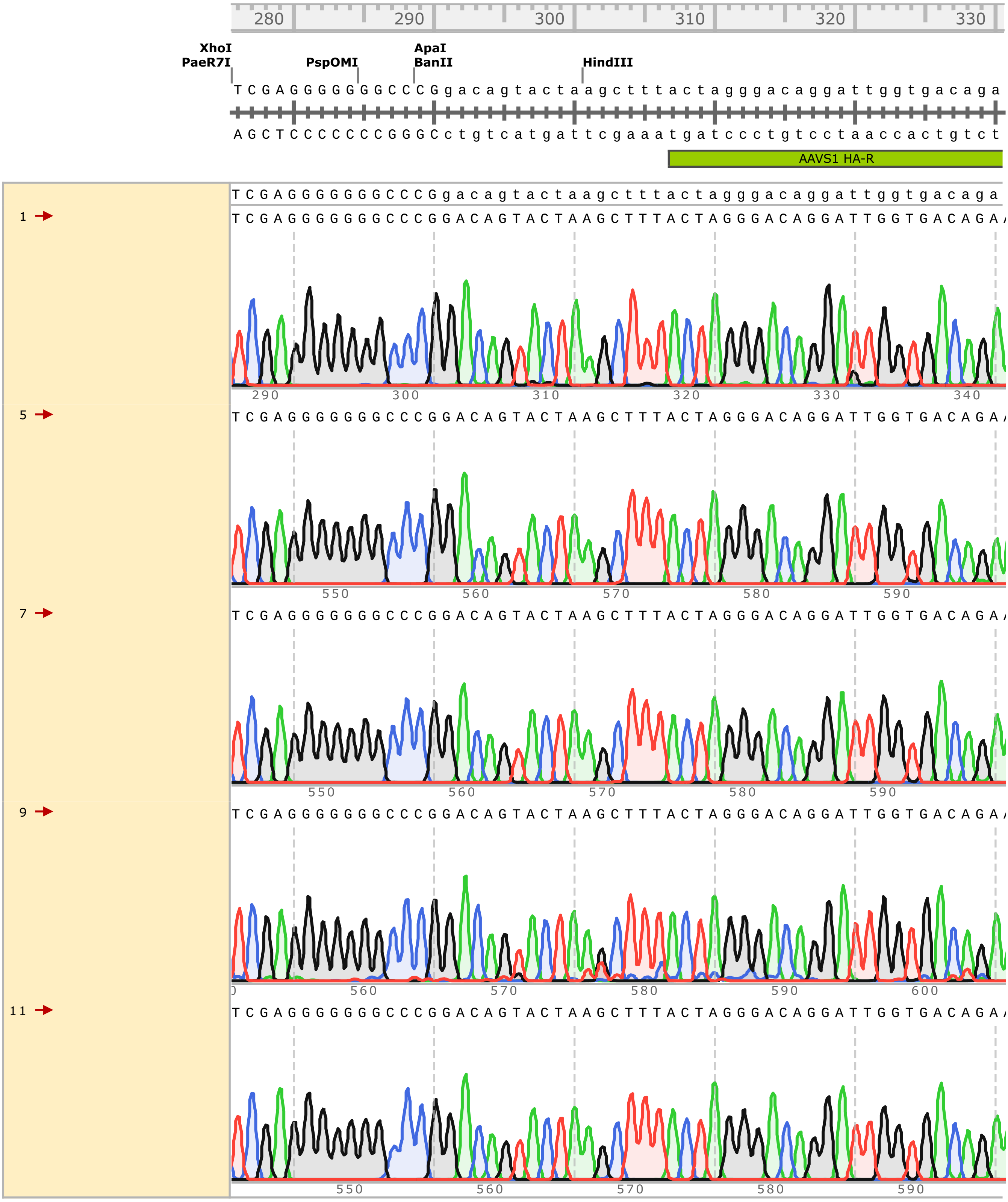
280
290
300
310
320
330
XhoI
PaeR7I
PspOMI
ApaI
BanII
HindIII
TCGAGGGGGGGCCCGgacagtactaagctttactagggacaggattggtgacaga
AGCTCCCCCCCGGGCctgtcatgattcgaaatgatccctgtcctaaccactgtct
AAVS1 HA-R
1
5
7
9
11

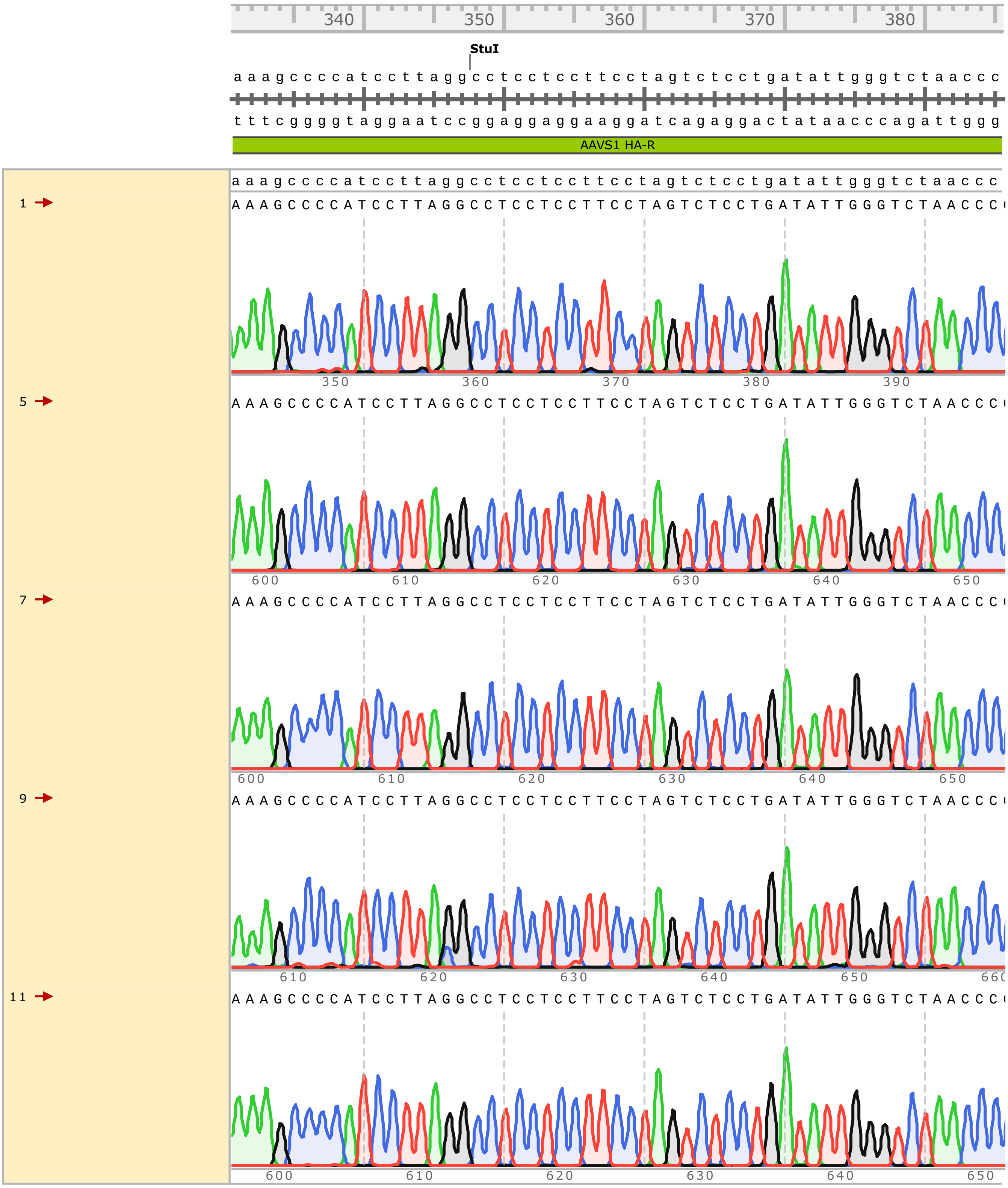
340 350 360 370 380
StuI
aaagccccatccttaggcctcctccttcctagtctcctgatattgggtctaaccc
tttcggggtaggaatccggaggaggaaggatcagaggactataacccagattggg
AAVS1 HA-R
aaagccccatccttaggcctcctccttcctagtctcctgatattgggtctaaccc
1
AAAGCCCCATCCTTAGGCCTCCTCCTTCCTAGTCTCCTGATATTGGGTCTAACCC
350 360 370 380 390
5
AAAGCCCCATCCTTAGGCCTCCTCCTTCCTAGTCTCCTGATATTGGGTCTAACCC
600 610 620 630 640 650
7
AAAGCCCCATCCTTAGGCCTCCTCCTTCCTAGTCTCCTGATATTGGGTCTAACCC
600 610 620 630 640 650
9
AAAGCCCCATCCTTAGGCCTCCTCCTTCCTAGTCTCCTGATATTGGGTCTAACCC
610 620 630 640 650
11
AAAGCCCCATCCTTAGGCCTCCTCCTTCCTAGTCTCCTGATATTGGGTCTAACCC
600 610 620 630 640 650

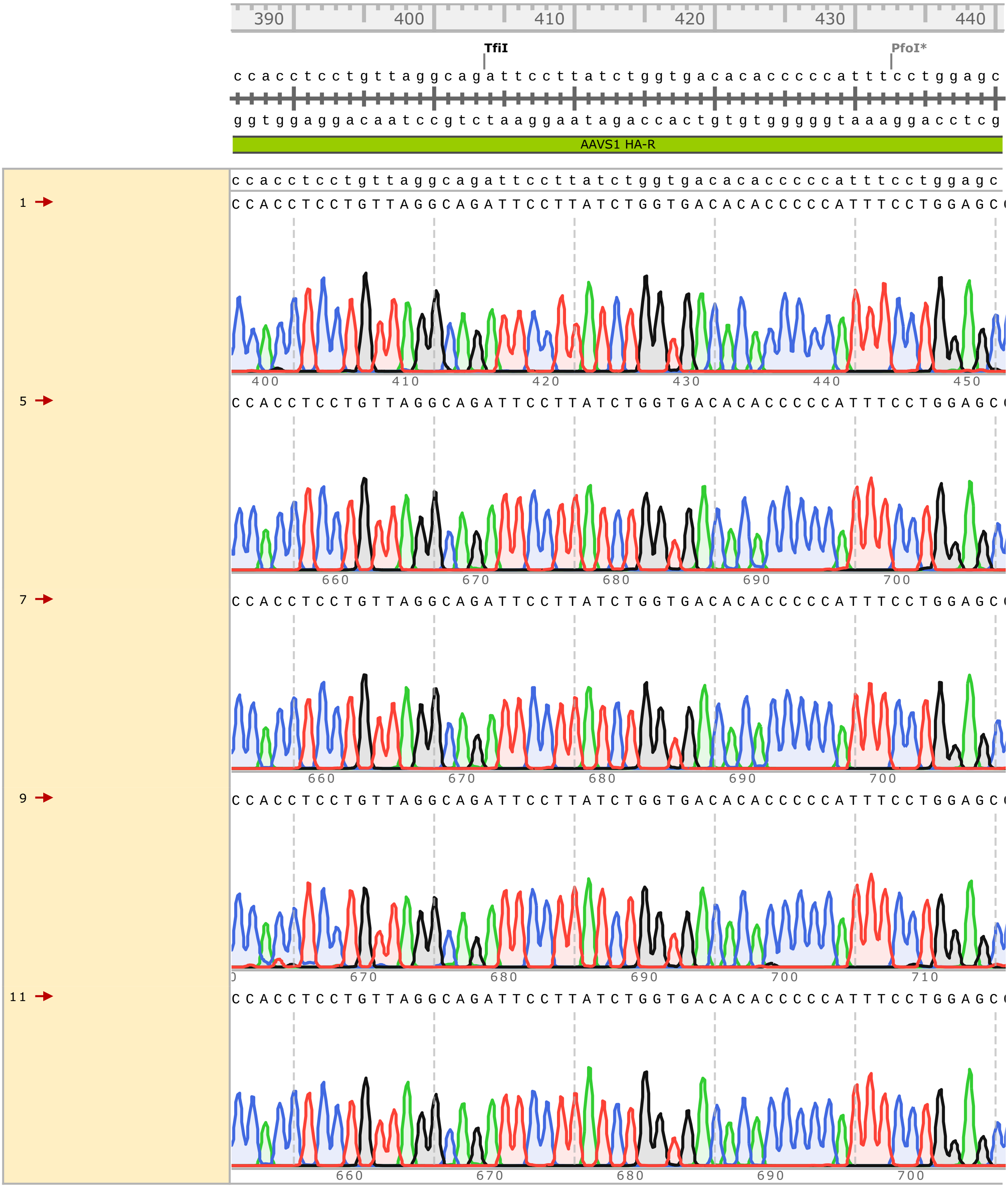
390 400 410 420 430 440
TfiI
PfoI*
ccacctcctgttaggcagattccttatctggtgacacaccccccatttcctggagc
ggtggaggacaatccgtctaaggaatagaccactgtgtggggggtaaaggacctcg
AAVS1 HA-R
ccacctcctgttaggcagattccttatctggtgacacaccccccatttcctggagc
1
CCACCTCCTGTTAGGCAGATTCCTTATCTGGTGACACACCCCCATTTCCTGGAGC
400 410 420 430 440 450
5
CCACCTCCTGTTAGGCAGATTCCTTATCTGGTGACACACCCCCATTTCCTGGAGC
660 670 680 690 700
7
CCACCTCCTGTTAGGCAGATTCCTTATCTGGTGACACACCCCCATTTCCTGGAGC
660 670 680 690 700
9
CCACCTCCTGTTAGGCAGATTCCTTATCTGGTGACACACCCCCATTTCCTGGAGC
670 680 690 700 710
11
CCACCTCCTGTTAGGCAGATTCCTTATCTGGTGACACACCCCCATTTCCTGGAGC
660 670 680 690 700

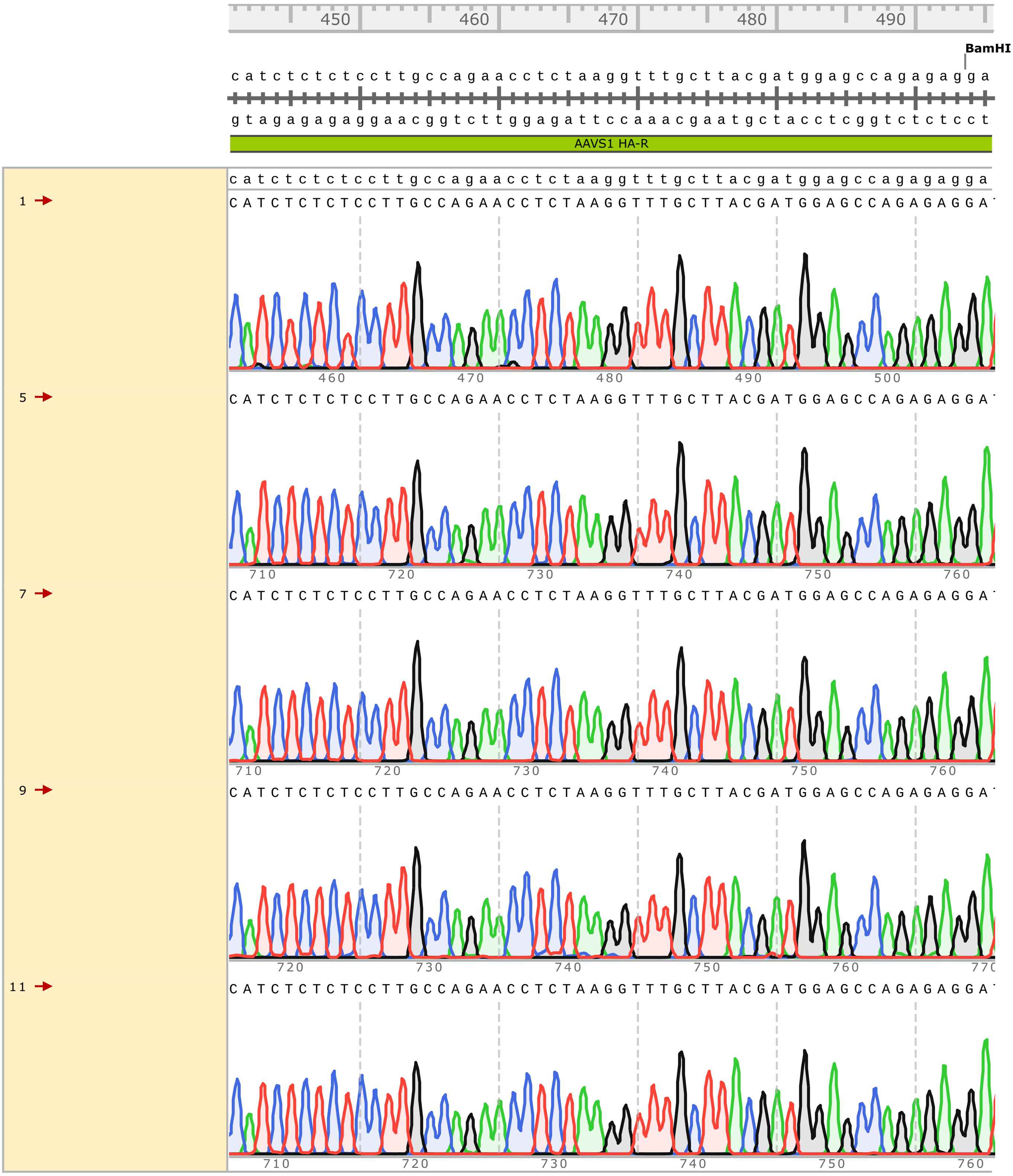
450
460
470
480
490
BamHI
c a t c t c t c t c c t t g c c a g a a c c t c t a a g g t t t g c t t a c g a t g g a g c c a g a g a g g a
g t a g a g a g a g g a a c g g t c t t g g a g a t t c c a a a c g a a t g c t a c c t c g g t c t c t c c t
AAVS1 HA-R
c a t c t c t c t c c t t g c c a g a a c c t c t a a g g t t t g c t t a c g a t g g a g c c a g a g a g g a
1
C A T C T C T C T C C T T G C C A G A A C C T C T A A G G T T T G C T T A C G A T G G A G C C A G A G A G G A
460 470 480 490 500
5
C A T C T C T C T C C T T G C C A G A A C C T C T A A G G T T T G C T T A C G A T G G A G C C A G A G A G G A
710 720 730 740 750 760
7
C A T C T C T C T C C T T G C C A G A A C C T C T A A G G T T T G C T T A C G A T G G A G C C A G A G A G G A
710 720 730 740 750 760
9
C A T C T C T C T C C T T G C C A G A A C C T C T A A G G T T T G C T T A C G A T G G A G C C A G A G A G G A
720 730 740 750 760
11
C A T C T C T C T C C T T G C C A G A A C C T C T A A G G T T T G C T T A C G A T G G A G C C A G A G A G G A
710 720 730 740 750 760

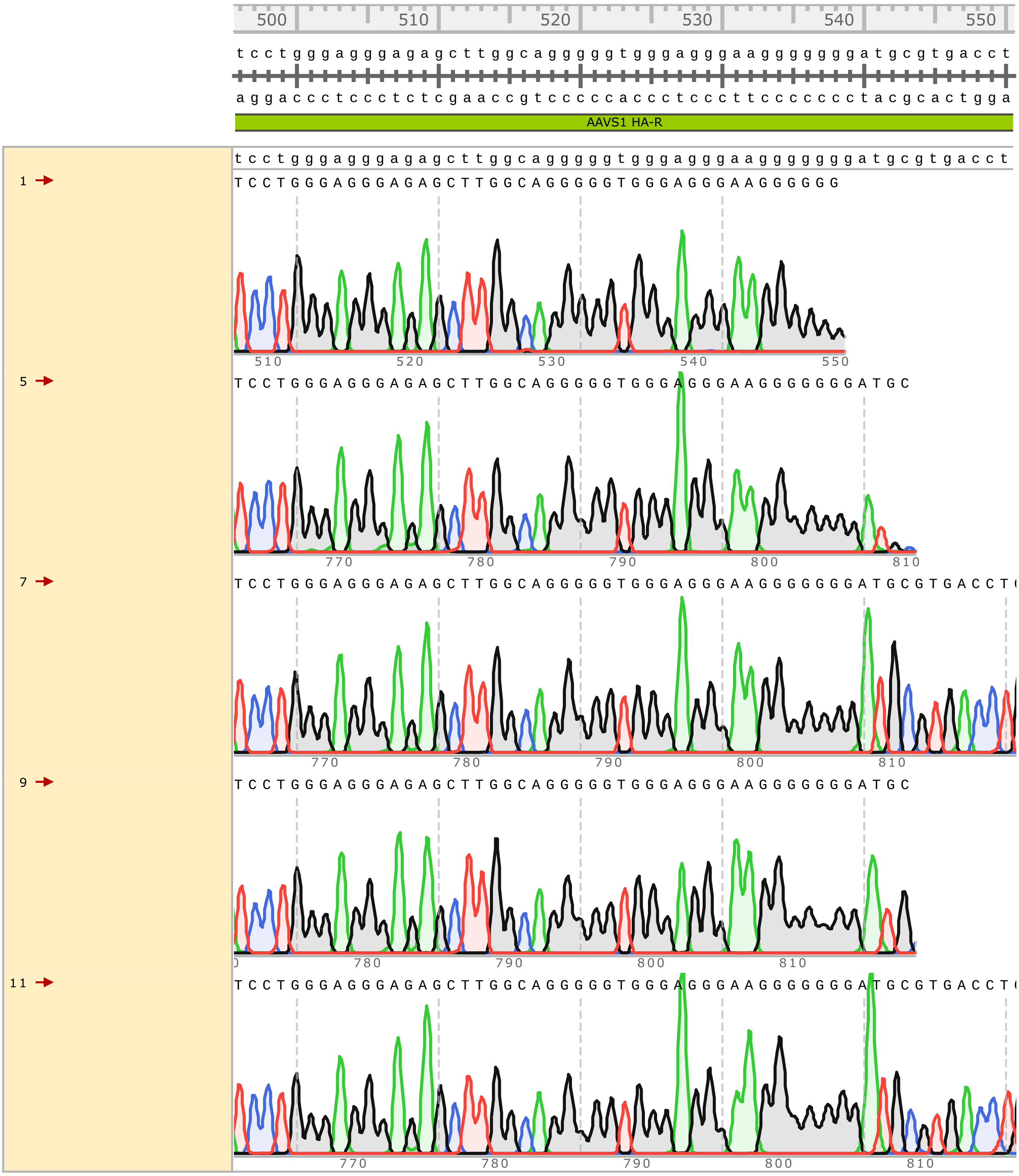
500 510 520 530 540 550
tcctgggagggagagcttggcagggggtgggagggaaggggggggatgcgtgacct
aggaccctccctctcgaaccgtcccccacccctcccttcccccccctacgcactgga
AAVS1 HA-R
tcctgggagggagagcttggcagggggtgggagggaaggggggggatgcgtgacct
1
TCCTGGGAGGGAGAGCTTGGCAGGGGGTGGGAGGGAAGGGGGG
510 520 530 540 550
5
TCCTGGGAGGGAGAGCTTGGCAGGGGGTGGGAGGGAAGGGGGGGATGC
770 780 790 800 810
7
TCCTGGGAGGGAGAGCTTGGCAGGGGGTGGGAGGGAAGGGGGGGATGCGTGACCT
770 780 790 800 810
9
TCCTGGGAGGGAGAGCTTGGCAGGGGGTGGGAGGGAAGGGGGGGATGC
780 790 800 810
11
TCCTGGGAGGGAGAGCTTGGCAGGGGGTGGGAGGGAAGGGGGGGATGCGTGACCT
770 780 790 800 810
