# Supplementary material for: Single cell transcriptional perturbome in pluripotent stem cell models
Source: Mol Syst Biol. 2025 Dec 10;22(2):179–227. doi: 10.1038/s44320-025-00172-8 (PMC12864791; doi:10.1038/s44320-025-00172-8)
Supplement: Supplementary file 8 — EV Figure Source Data [file 44320_2025_172_MOESM8_ESM.zip › EV figures/FigEV6.zip!/FigEV6/EV6B/SMAD2_Final_pool.pdf]

Sequence: pKS004_pAAV-Puro_siKD_SMAD2_Final_pool.dna (Circular / 2750 bp)
Features: 8 total
Primers: 6 total

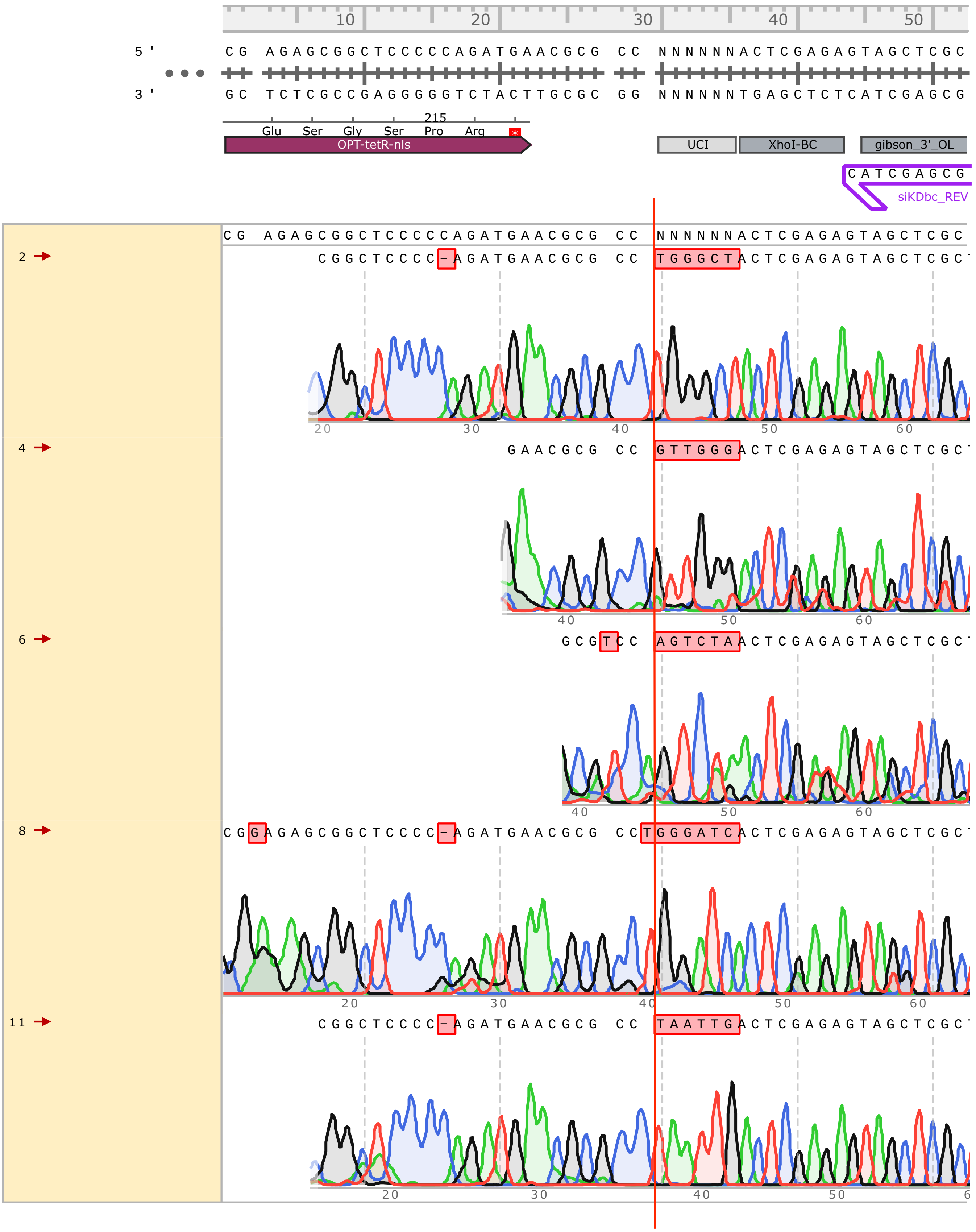

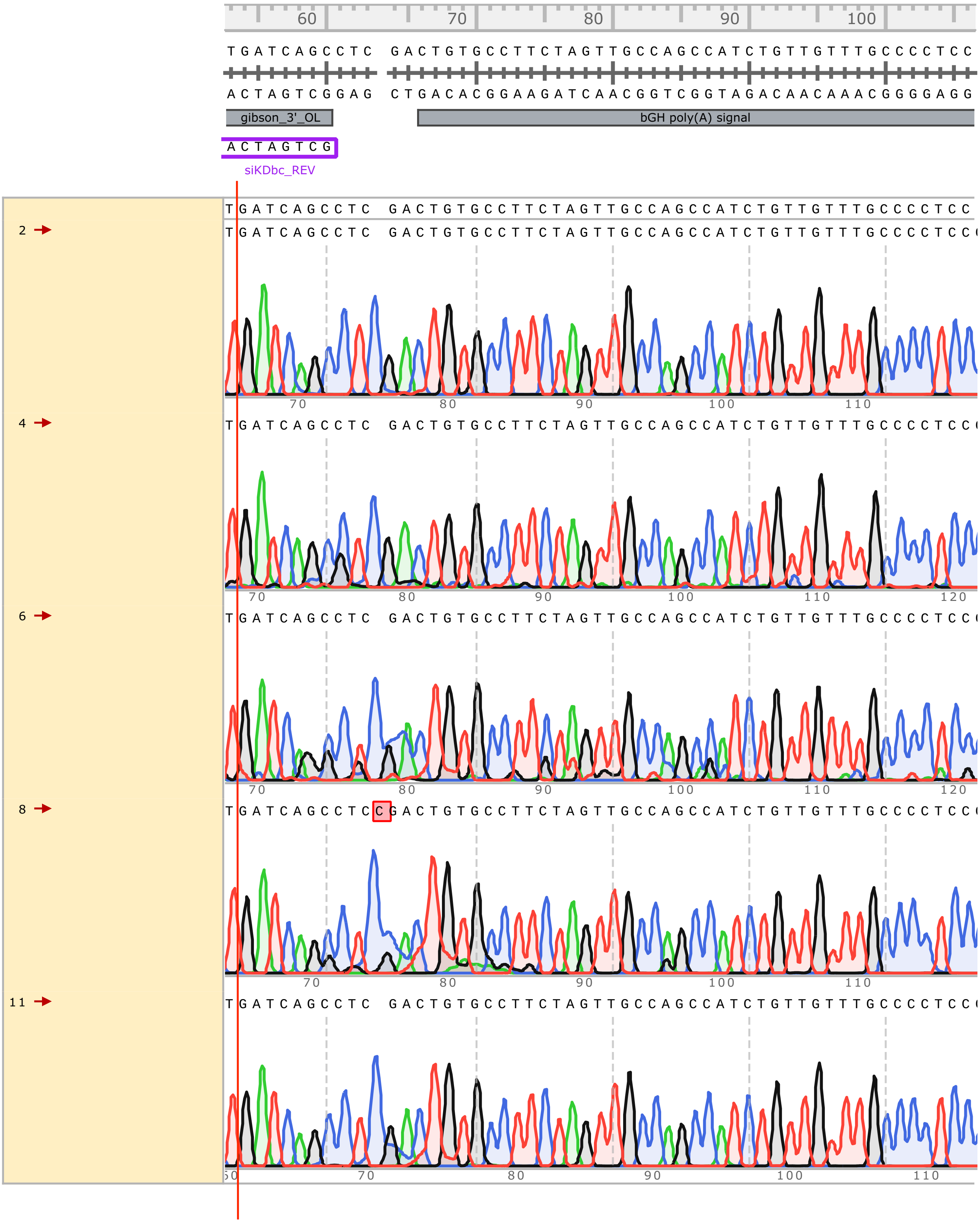
60 70 80 90 100
TGATCAGCCTC GACTGTGCCTTCTAGTTGCCAGCCATCTGTTGTTTGCCCCTCC
ACTAGTCGGAG CTGACACGGAAGATCAACGGTCGGTAGACAACAAACGGGGAGG
gibson_3'_OL
bGH poly(A) signal
ACTAGTCG
siKDbc_REV
2
4
6
8
11

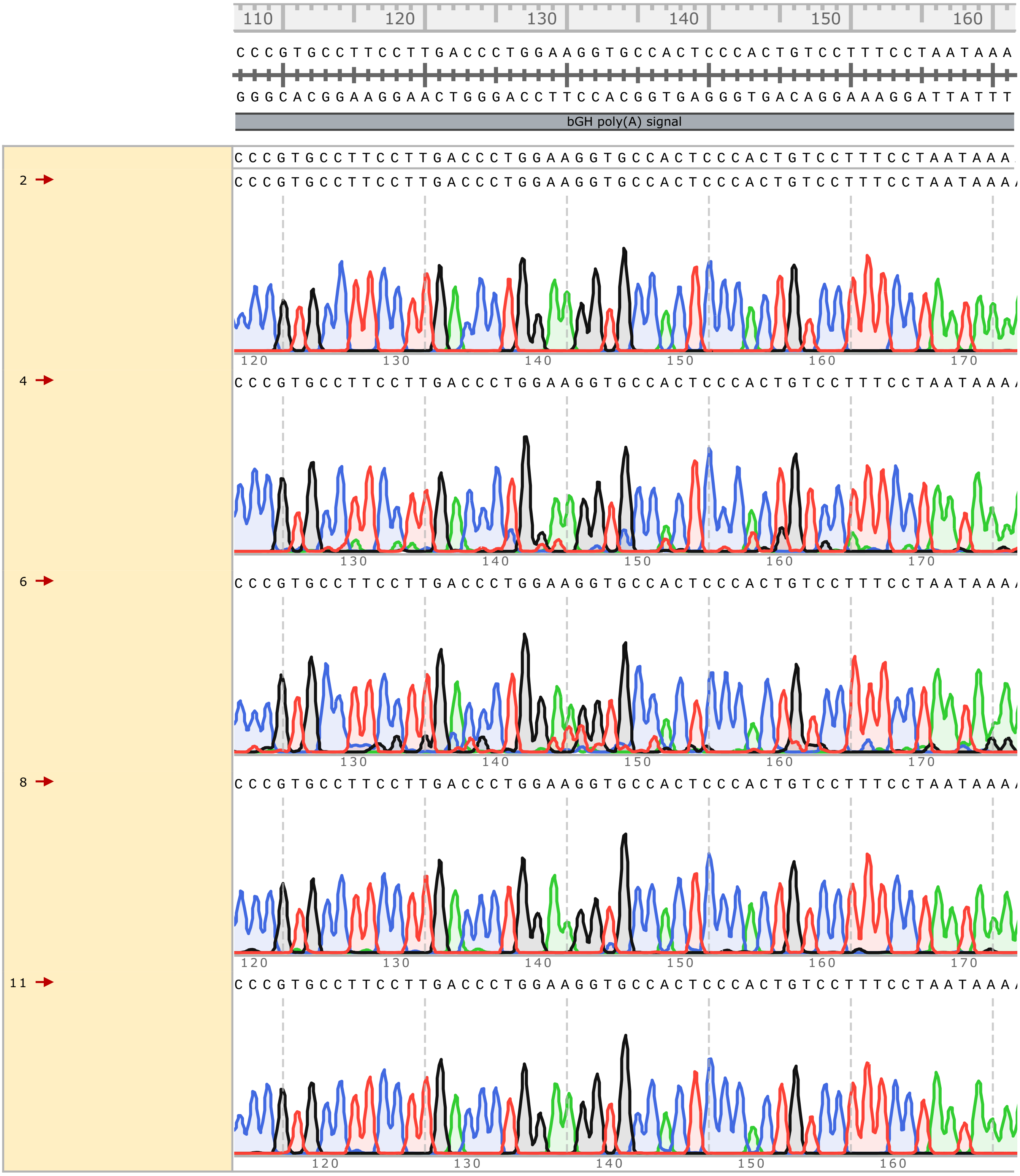
110 120 130 140 150 160
C C C G T G C C T T C C T T G A C C C T G G A A G G T G C C A C T C C C A C T G T C C T T T C C T A A T A A A
G G G C A C G G A A G G A A C T G G G A C C T T C C A C G G T G A G G G T G A C A G G A A A G G A T T A T T T
bGH poly(A) signal
2
4
6
8
11
120 130 140 150 160 170

170 180 190 200 210

ATGAGGAAATTGCATCGCATTGTCTGAGTAGGTGTCATTCTATTCTGGGGGGTGG

TACTCCTTTAACGTAGCGTAACAGACTCATCCACAGTAAGATAAGACCCCCCACC

bGH poly(A) signal

ATGAGGAAATTGCATCGCATTGTCTGAGTAGGTGTCATTCTATTCTGGGGGGTGG

2 → ATGAGGAAATTGCATCGCATTGTCTGAGTAGGTGTCATTCTATTCTGGGGGGTGG

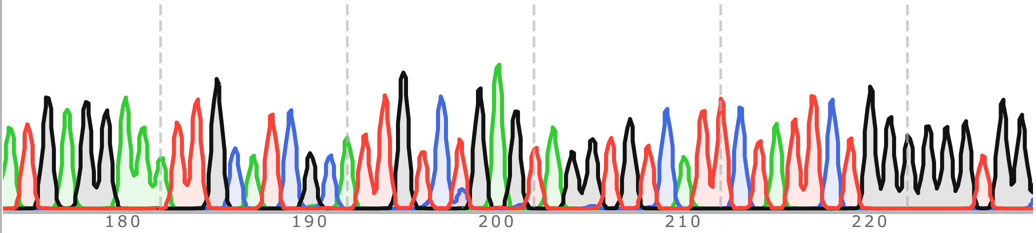


4 → ATGAGGAAATTGCATCGCATTGTCTGAGTAGGTGTCATTCTATTCTGGGGGGTGG

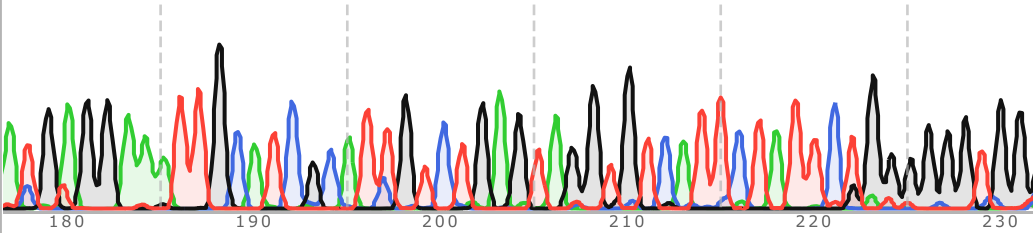


6 → ATGAGGAAATTGCATCGCATTGTCTGAGTAGGTGTCATTCTATTCTGGGGGGTGG

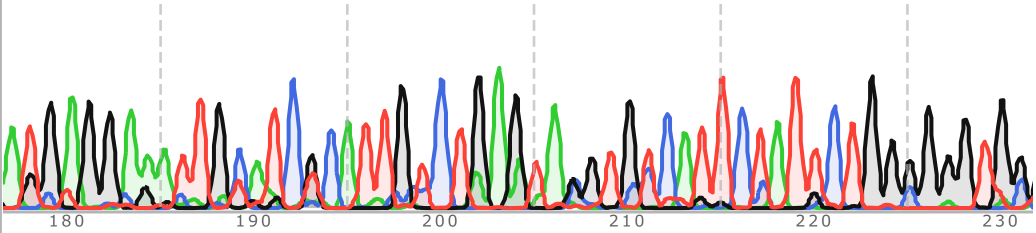


8 → ATGAGGAAATTGCATCGCATTGTCTGAGTAGGTGTCATTCTATTCTGGGGGGTGG

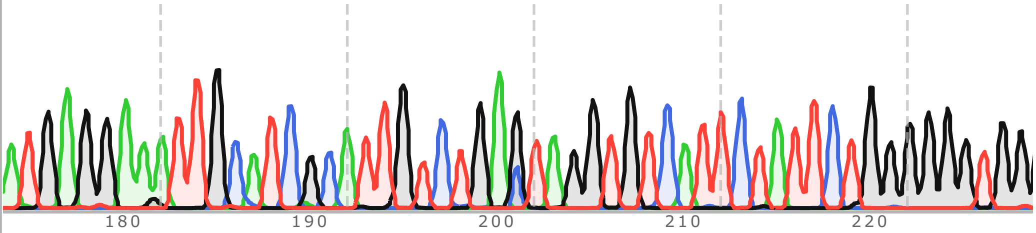


11 → ATGAGGAAATTGCATCGCATTGTCTGAGTAGGTGTCATTCTATTCTGGGGGGTGG

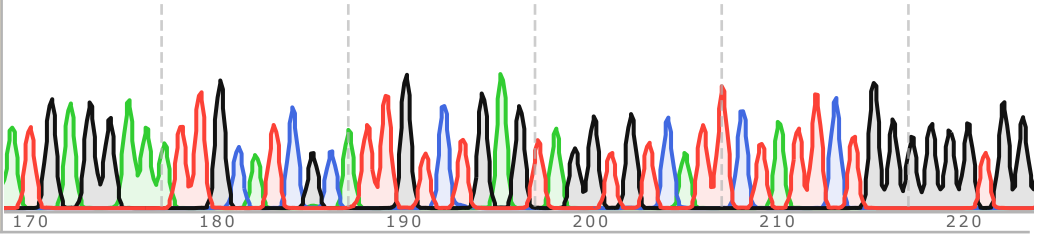

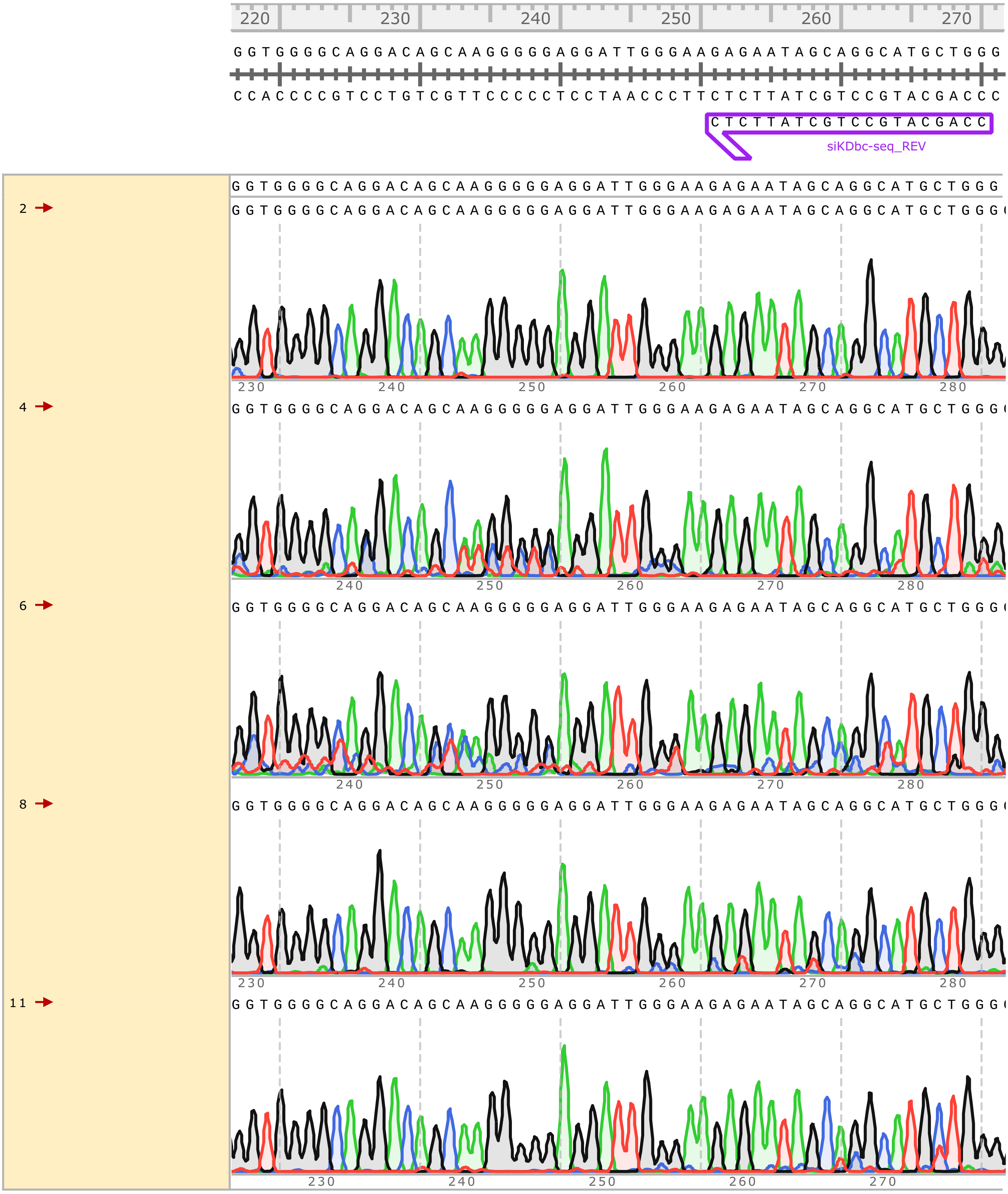
220 230 240 250 260 270
GGTGGGGCAGGACAGCAAGGGGGAGGATTGGGAAGAGAATAGCAGGCATGCTGGG
CCACCCCGTCCTGTCGTTCCCCCTCCTAACCCTTCTCTTATCGTCCGTACGACCC
CTCTTATCGTCCGTACGACC
siKDbc-seq_REV
2
4
6
8
11

280 290 300 310 320

GGTCTCGAGGGGGGGCCCGgacagtactaagctttactagggacaggattggtga

CCAGAGCTCCCCCCCGGGCctgtcatgattcgaaatgatccctgtcctaaccact

AAVS1 HA-R

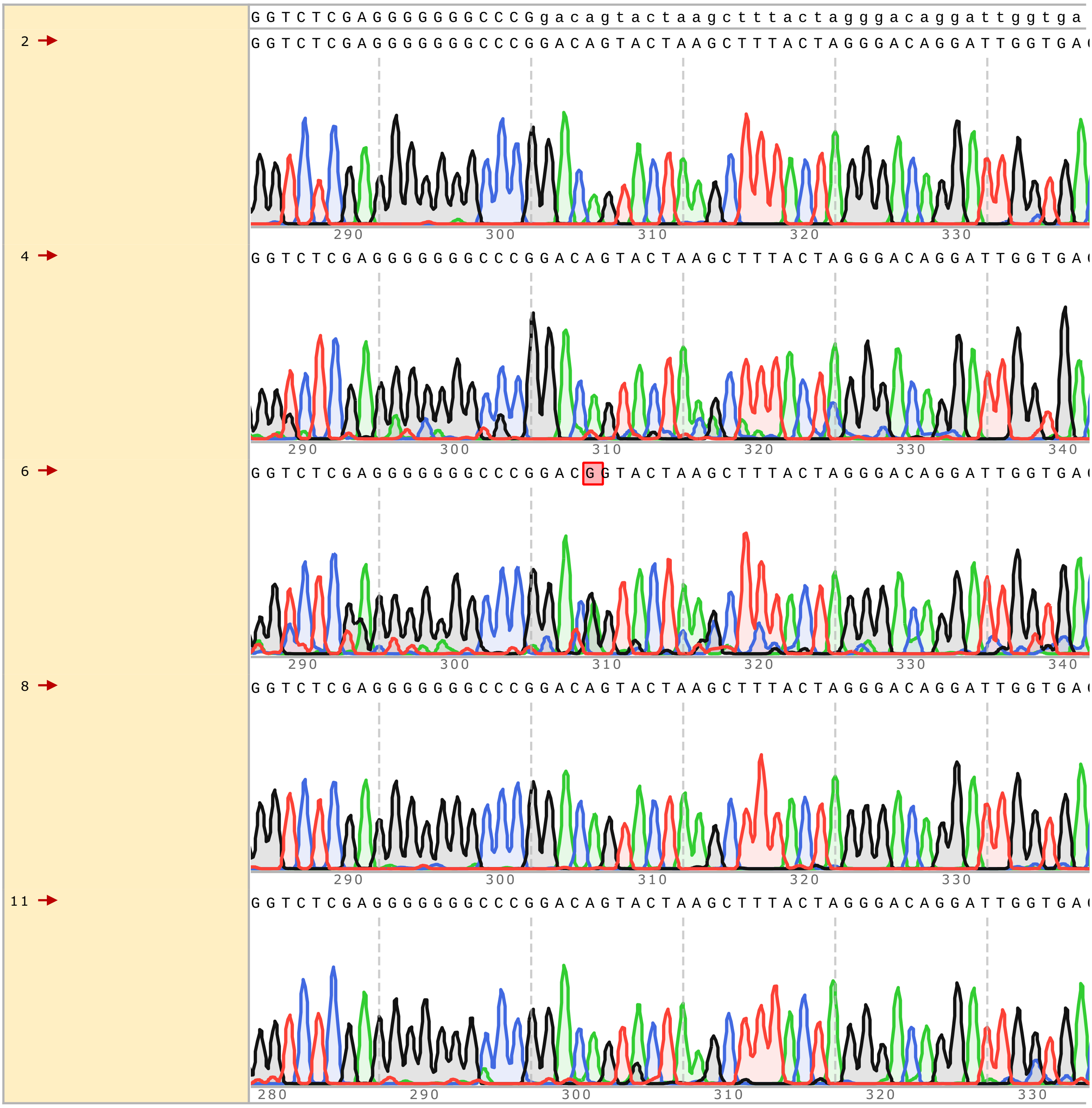

330 340 350 360 370 380

c a g a a a a g c c c c a t c c t t a g g c c t c c t c c t t c c t a g t c t c c t g a t a t t g g g t c t a

g t c t t t t c g g g g t a g g a a t c c g g a g g a g g a a g g a t c a g a g g a c t a t a a c c c a g a t

AAVS1 HA-R

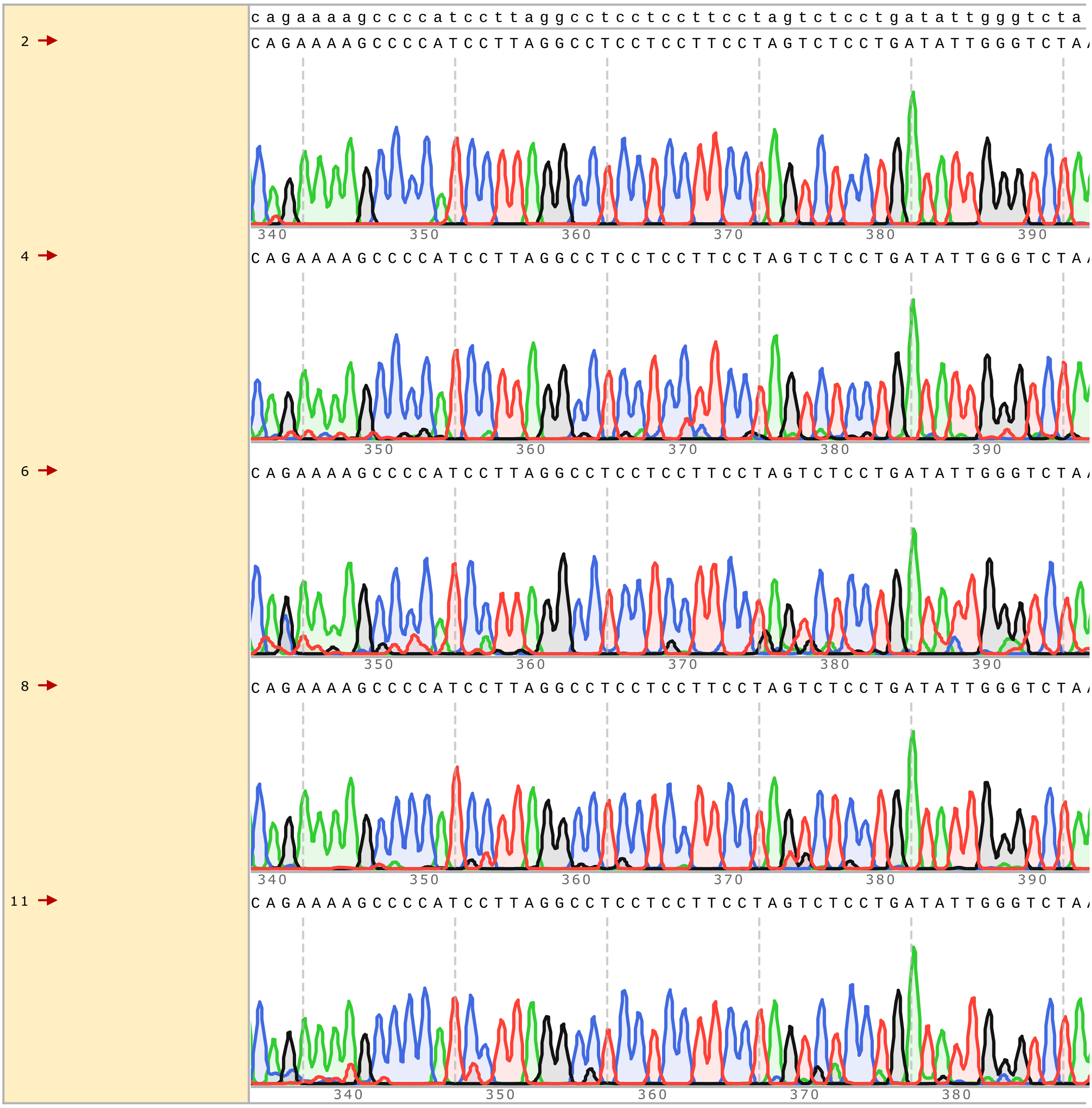

390 400 410 420 430

a c c c c c a c c t c c t g t t a g g c a g a t t c c t t a t c t g g t g a c a c a c c c c c a t t t c c t g

t g g g g g t g g a g g a c a a t c c g t c t a a g g a a t a g a c c a c t g t g t g g g g g t a a a g g a c

AAVS1 HA-R

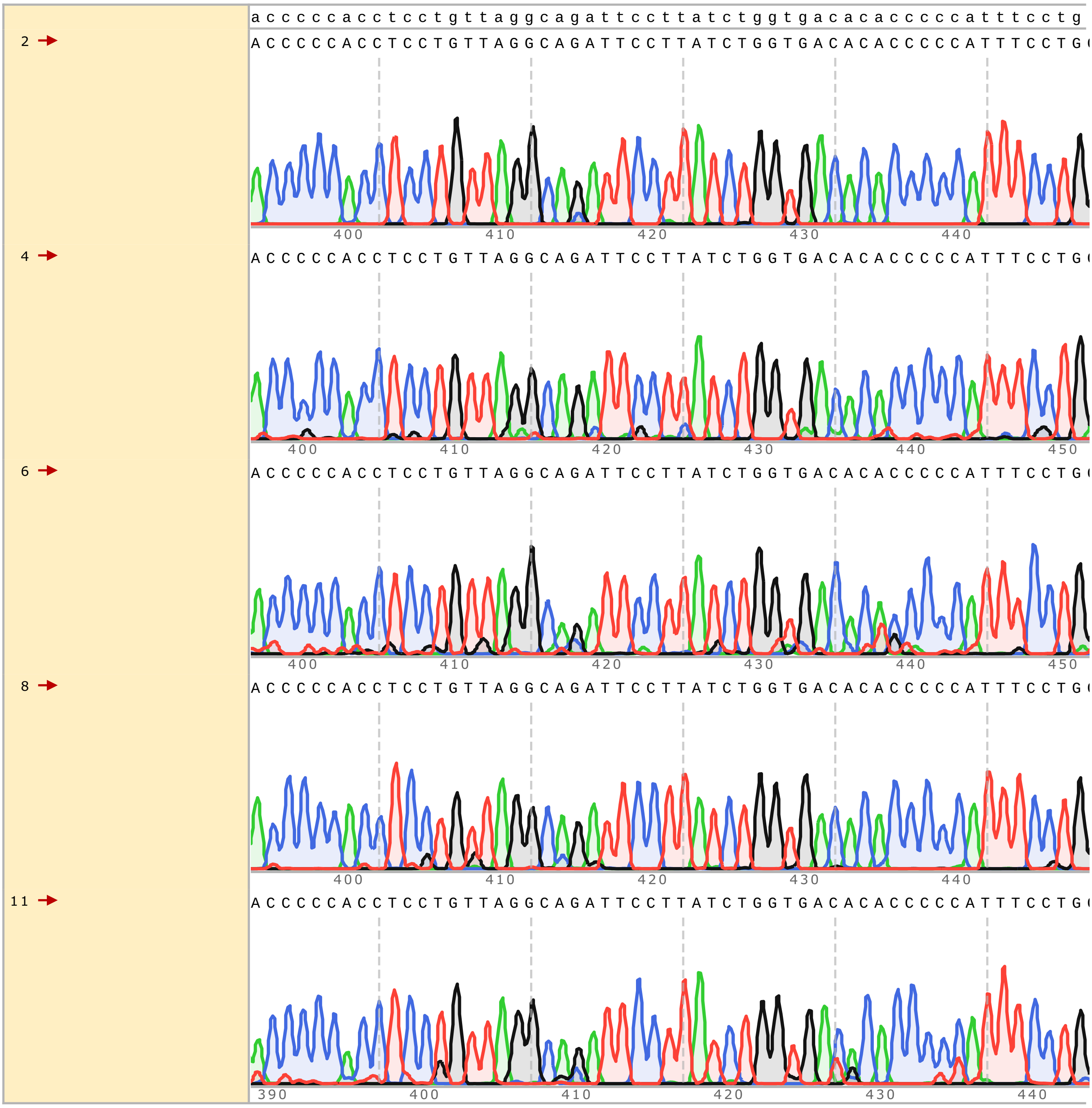

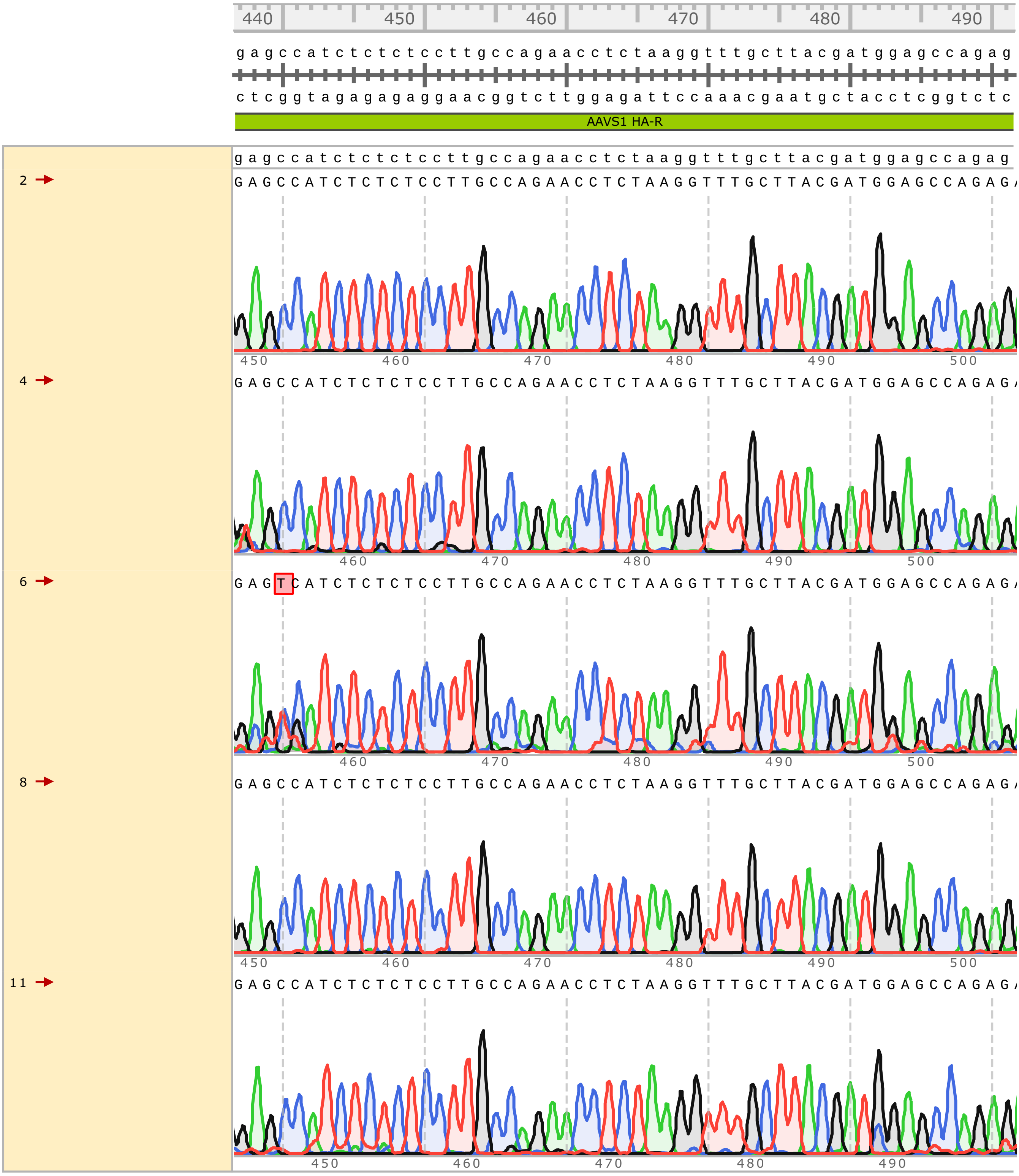

440 450 460 470 480 490
g a g c c a t c t c t c t c c t t g c c a g a a c c t c t a a g g t t t g c t t a c g a t g g a g c c a g a g
c t c g g t a g a g a g a g g a a c g g t c t t g g a g a t t c c a a a c g a a t g c t a c c t c g g t c t c
AAVS1 HA-R
g a g c c a t c t c t c t c c t t g c c a g a a c c t c t a a g g t t t g c t t a c g a t g g a g c c a g a g
2
G A G C C A T C T C T C T C C T T G C C A G A A C C T C T A A G G T T T G C T T A C G A T G G A G C C A G A G
450 460 470 480 490 500
4
G A G C C A T C T C T C T C C T T G C C A G A A C C T C T A A G G T T T G C T T A C G A T G G A G C C A G A G
460 470 480 490 500
6
G A G T C A T C T C T C T C C T T G C C A G A A C C T C T A A G G T T T G C T T A C G A T G G A G C C A G A G
460 470 480 490 500
8
G A G C C A T C T C T C T C C T T G C C A G A A C C T C T A A G G T T T G C T T A C G A T G G A G C C A G A G
450 460 470 480 490 500
11
G A G C C A T C T C T C T C C T T G C C A G A A C C T C T A A G G T T T G C T T A C G A T G G A G C C A G A G
450 460 470 480 490

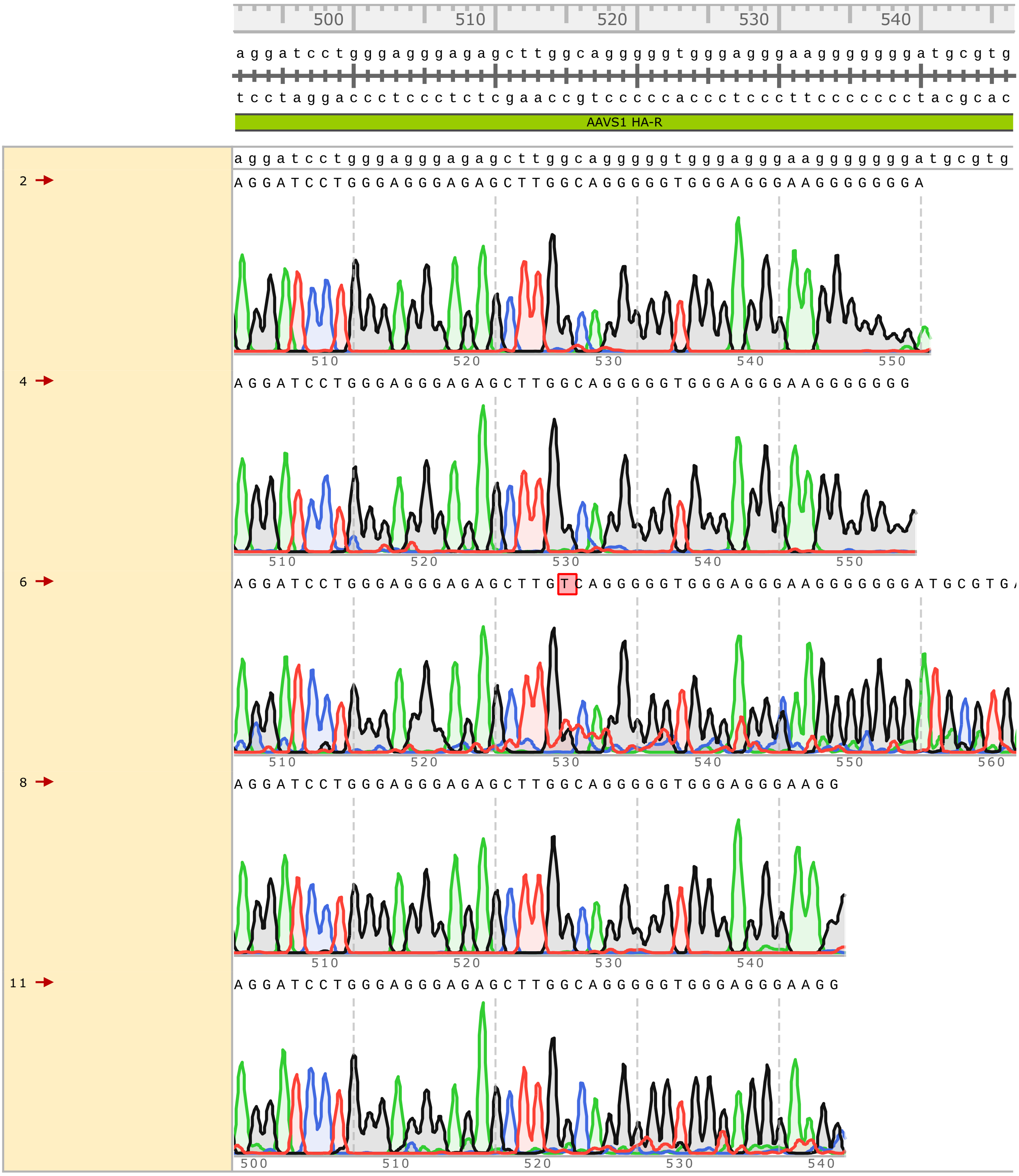
500 510 520 530 540
a g g a t c c t g g g a g g g a g a g c t t g g c a g g g g g t g g g a g g g a a g g g g g g g a t g c g t g
t c c t a g g a c c c t c c c t c t c g a a c c g t c c c c c a c c c t c c c t t c c c c c c c t a c g c a c
AAVS1 HA-R
a g g a t c c t g g g a g g g a g a g c t t g g c a g g g g g t g g g a g g g a a g g g g g g g a t g c g t g
2
A G G A T C C T G G G A G G G A G A G C T T G G C A G G G G G T G G G A G G G A A G G G G G G G A
510 520 530 540 550
4
A G G A T C C T G G G A G G G A G A G C T T G G C A G G G G G T G G G A G G G A A G G G G G G G
510 520 530 540 550
6
A G G A T C C T G G G A G G G A G A G C T T G T C A G G G G G T G G G A G G G A A G G G G G G G A T G C G T G
510 520 530 540 550 560
8
A G G A T C C T G G G A G G G A G A G C T T G G C A G G G G G T G G G A G G G A A G G
510 520 530 540
11
A G G A T C C T G G G A G G G A G A G C T T G G C A G G G G G T G G G A G G G A A G G
500 510 520 530 540
