# Supplementary material for: Single cell transcriptional perturbome in pluripotent stem cell models
Source: Mol Syst Biol. 2025 Dec 10;22(2):179–227. doi: 10.1038/s44320-025-00172-8 (PMC12864791; doi:10.1038/s44320-025-00172-8)
Supplement: Supplementary file 8 — EV Figure Source Data [file 44320_2025_172_MOESM8_ESM.zip › EV figures/FigEV5.zip!/FigEV5/EV5C_D/hystograms.pdf]

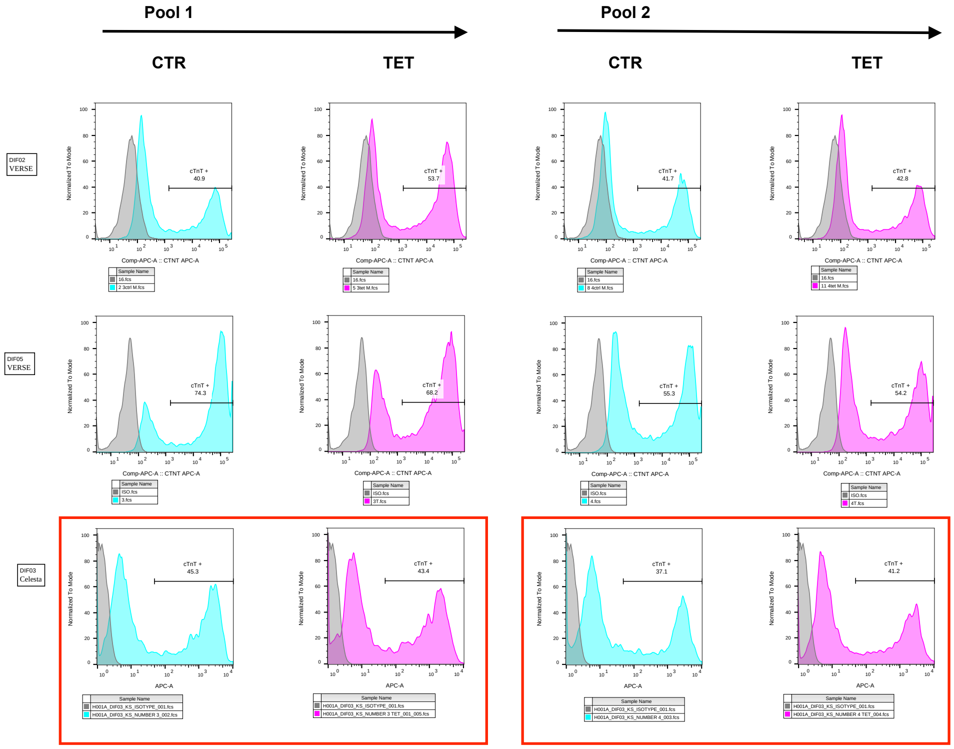
Pool 1
Pool 2
CTR
TET
CTR
TET
DIF02 VERSE
DIF05 VERSE
DIF03 Celesta
Normalized To Mode
Comp-APC-A :: CTNT APC-A
APC-A
Sample Name
cTnT + 40.9
cTnT + 53.7
cTnT + 41.7
cTnT + 42.8
cTnT + 74.3
cTnT + 68.2
cTnT + 55.3
cTnT + 54.2
cTnT + 45.3
cTnT + 43.4
cTnT + 37.1
cTnT + 41.2
