# Supplementary material for: Single cell transcriptional perturbome in pluripotent stem cell models
Source: Mol Syst Biol. 2025 Dec 10;22(2):179–227. doi: 10.1038/s44320-025-00172-8 (PMC12864791; doi:10.1038/s44320-025-00172-8)
Supplement: Supplementary file 8 — EV Figure Source Data [file 44320_2025_172_MOESM8_ESM.zip › EV figures/FigEV5.zip!/FigEV5/EV5A/README.rtf]

Incucite figures were exported without manipulations, and then pictures were cropped, as shown by the red box, and combined.
